# Supplementary material for: Marine furanocembranoids-inspired macrocycles enabled by Pd-catalyzed unactivated C(sp3)-H olefination mediated by donor/donor carbenes
Source: Nat Commun. 2021 Feb 26;12:1304. doi: 10.1038/s41467-021-21484-x (PMC7910576; doi:10.1038/s41467-021-21484-x)
Supplement: Supplementary file 1 — Supplementary Information [file 41467_2021_21484_MOESM1_ESM.pdf]

Marine Furanocembranoids-Inspired Macrocycles Enabled by Pd-catalyzed Unactivated C(sp^3)-H Olefination Mediated by Donor/Donor Carbenes

Jiping Hao,[1+] Xueying Guo,[2+] Shijun He,[1,3+] Zhongliang Xu,[1,3] Lu Chen,[1,3] Zhongyu Li,[1] Bichao Song,[1,3] Jianping Zuo,[1,3] Zhenyang Lin[2]* and Weibo Yang[1,3,4]*

1 Chinese Academy of Sciences Key Laboratory of Receptor Research, Shanghai Institute of Materia Medica (SIMM), Chinese Academy of Sciences, Shanghai, China

2 Department of Chemistry, The Hong Kong University of Science and Technology, Clear Water Bay, Kowloon, Hong Kong, China

3 University of Chinese Academy of Sciences, Beijing 100049, China

4 School of Pharmaceutical Science and Technology, Hangzhou Institute for Advanced Study, University of Chinese Academy of Sciences, Hangzhou 310024, China.

Supplementary Methods

General comments

NMR spectra were recorded at room temperature on the following spectrometers: Bruker Avance III 400 Spectrometer (400 MHz) and Bruker Avance III 500 (Cryo) Spectrometer (500 MHz). Chemical shifts are given in ppm and coupling constants in Hz. ^{1}H spectra were calibrated in relation to the reference measurement of TMS (0.00 ppm). ^{13}C spectra were calibrated in relation to deuterated solvents. The following abbreviations were used for ^{1}H NMR spectra to indicate the signal multiplicity: s (singlet), d (doublet), t (triplet), q (quartet) and m (multiplet) as well as combinations of them. For HRMS data, the ESI-positive method was applied on the Agilent G6520 Q-TOF. Chemicals were purchased from commercial suppliers. Unless stated otherwise, all the substrates and solvents were purified and dried according to standard methods prior to use.

1. General Procedure for the Synthesis of the starting materials

1.1 Synthesis of 1-Bromo-2-*tert*-butylbenzenes

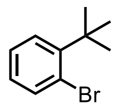

1a

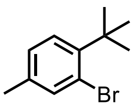

1b

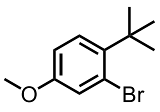

1c

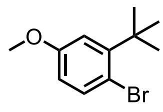

1d

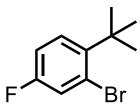

1e

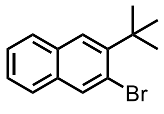

1f

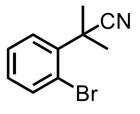

1g

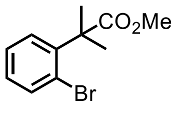

1h

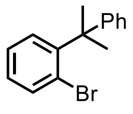

1i

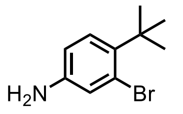

ij

1a was prepared by following method A[1]. **1b**, **1c**, **1d**, **1e**, **1f**, and **1i** were prepared by following method B[2]. **1g** and **1i** have commercial sources. **1h** were prepared by following method C[3].

Method A:

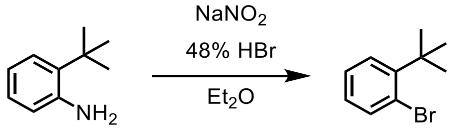

2-*tert*-Butylaniline (2.50 g, 16.7 mmol) was added to 13 mL 48% w/w hydrobromic acid. The thus formed pale pink suspension was cooled to -56 °C. Then, sodium nitrite (1.68 g, 24.4 mmol) was added portion-wise. After stirring for 1 hour, 20 mL Et_2O was added slowly. The temperature was adjusted to -8 °C over 2 hours and kept at -8 °C for an additional 2 hours. Next, the system was cooled to -40 °C again. 3.06 g Na_2CO_3 was added. The reaction was then warming up slowly to room temperature over 3 hours and stirred overnight. The reaction was diluted with water and extracted with EtOAc. The organic layer was washed with water, $NaHCO_3$ (aq.), brine, dried over Na_2SO_4, concentrated in vacuo, and purified by silica gel column chromatography with petroleum ether to afford 1-bromo-2-(*tert*-butyl)benzene as a colorless oil.(1.49 g, 42% yield)

Method B:

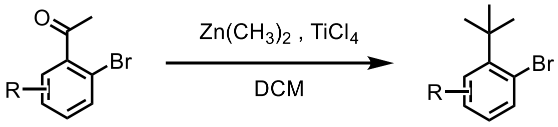

Under nitrogen atmosphere, $TiCl_4$ (1 M in CH_2Cl_2, 2.2 equiv.) was added to anhydrous CH_2Cl_2 (0.3 M) and cooled to - 40 °C. Then, a solution of Me_2Zn (1 M in hexane, 2.2 equiv.) was added dropwise. After stirring for 15 minutes, a solution of aryl ketone in anhydrous CH_2Cl_2 (1 M) was added dropwise. The reaction was warm to 0 °C slowly (over 3 hours), stirred at 0 °C overnight, and poured into ice water. The aqueous phase was extracted with CH_2Cl_2. The combined organic layers were washed with $NaHCO_3$ (aq.), brine, dried over Na_2SO_4, filtered and concentrated in vacuo. (If necessary, the crude product was dissolved in CH_2Cl_2, treated with 3-chloroperoxybenzoic acid (*m*-CPMA) and stirred at room temperature to remove the

aryl propene, a by-product of the reaction, which is not easy to be separated from the desired product by silica gel column chromatography). The crude product was purified by silica gel column chromatography with petroleum ether/ethyl acetate to afford the corresponding aryl bromide. (45-67% yield)

Method C:

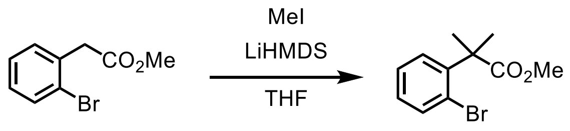

To a stirred solution of HMDS (4.25 mL, 20.0 mmol) in THF (36.8 mL) was added *n*-BuLi (1.60 M in hexane, 12.5 mL, 20.0 mmol) at 0 °C. After the mixture was stirred for 1.5 h, a solution of methyl 2-(2-bromophenyl)acetate (1.2 g, 5.0 mmol) in THF (25.0 mL) was added, and the mixture was stirred for 2 h at room temperature. Then CH_3I (1.20 mL, 20.0 mmol) was added at 0 °C, and the mixture was stirred for 12 h at room temperature. Then the reaction was quenched by addition of 1 M HCl at 0 °C. The crude mixture was extracted with EtOAc (x4) and the combined organic extracts were washed with brine, dried (Na_2SO_4) and concentrated in vacuo. The crude product was purified by silica gel column chromatography with petroleum ether/ethyl acetate to afford the corresponding product as a colorless oil. (1g, 78% yield)

1.2 Synthesis of complex 1-Bromo-2-*tert*-butylbenzenes from drug molecules (1k ~ 1p)

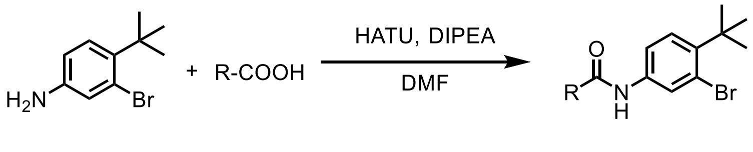

3-bromo-4-(tert-butyl)aniline(70 mg, 0.3 mmol), and the corresponding complex acids(0.36 mmol), and HATU(171 mg, 0.45 mmol), DIPEA(156 mg, 1.2 mmol) were in 5 mL DMF. The mixture was stirred at rt for 12 h. The reaction was diluted with water and extracted with EtOAc. The organic layer was washed with brine, dried over

Na_2SO_4, concentrated in vacuo, and purified by silica gel column chromatography with PE and EA (PE = petroleumether, EA = ethyl acetate) to afford corresponding products.

1.3 Synthesis of Ene-Yne-Ketones

The ene-yne-ketones used in this study were prepared by two steps starting from terminal alkynes and 1,3-dicarbonyl compounds.

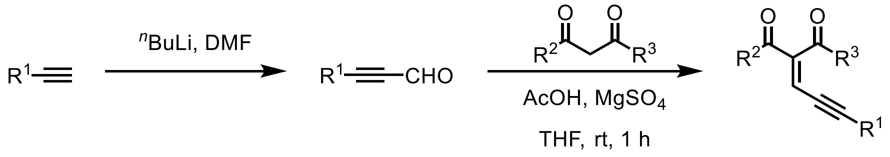

The alkyne (10 mmol) was dissolved in dry THF (15 mL) and the solution was cooled to -40 °C under nitrogen, n-butyllithium (1.6 M in hexanes, 6.8 mL, 11 mmol) was added dropwise over 2 minutes while maintaining the temperature between -35 and -40 °C. After completion of the addition, anhydrous DMF (1.55 mL, 20 mmol) was added in one portion and the cold bath was removed. The reaction mixture was warm to room temperature and aged for 30 minutes. The THF solution was poured into a vigorously stirred biphasic solution prepared from aqueous solution of KH_2PO_4 (50 mL, 30 mmol) and Et_2O (30 mL) cooled over ice to about 5 °C. Layers were separated and the organic extract was washed with water (2 x 30 mL). Combined aqueous layers were back extracted with Et_2O (30 mL). Combined organic layers were dried over Na_2SO_4 and filtered. Then solvent was removed in vacuo carefully under 0 °C to leave a crude acetylenic aldehyde.

The crude product was then dissolved in THF (8 mL), and 1,3-dicarbonyl compounds (10 mmol) was added into the solution. Then AcOH (2 mmol) and $MgSO_4$ (2 mmol) was added to the reaction mixture. The mixture was stirred at room temperature for about one hour. When the reaction was completed as monitored by TLC, filtration through celite and removal of the solvent by rotary evaporation gave the crude product. The ene-yne-ketones was purified by chromatography on silica gel with the appropriate

mixture of PE and EA (PE = petroleum ether, EA = ethyl acetate) with about 50-70% yields (two steps). [4]

1.4 Synthesis of Allenes (4a ~ 4h)

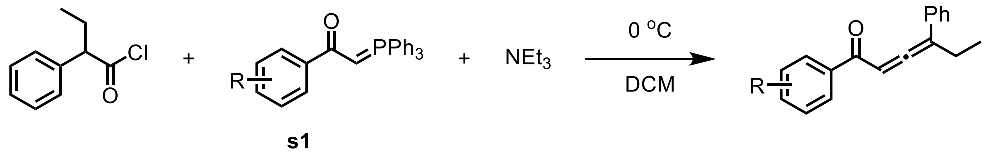

To an ice-cooled (0°C) solution of **s1**(7.89 mmol) and triethylamine (1.1 ml, 7.89 mmol) in 23 ml of dry dichloromethane stirred under argon was added dropwise a solution of 2-phenylbutanoyl chloride (2.02 g, 7.89 mmol) in 8 ml of dry dichloromethane. The resulting bright yellow solution was stirred for 2 h. After removal of half of the solvent, the residue was diluted with diethyl ether to precipitate triphenylphosphine oxide. After filtration, silica gel was added to adsorb the reaction products and the solvent was evaporated in vacuo. Flash Silica column chromatography (1:10 EtOAc/hexanes) gave the corresponding products[5].(21-50% yield)

2. Procedure for the Gram-Scale reaction for 3ba and the transformation

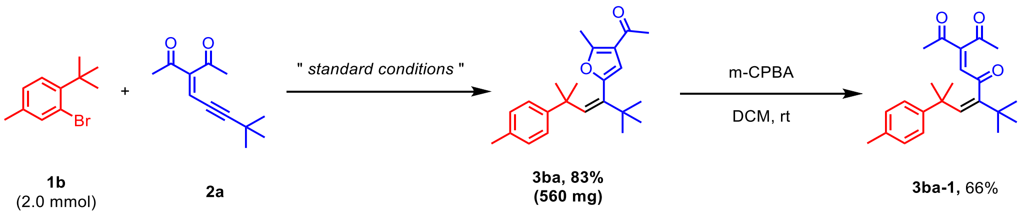

A screw-capped vial was charged with 2-bromo-1-(*tert*-butyl)-4-methylbenzene **1b** (2.0 mmol, 454 mg, 1.0 equiv.), the respective 3-(4,4-dimethylpent-2-yn-1-ylidene)pentane-2,4-dione **2a** (4.0 mmol, 770 mg, 2.0 equiv.), [Pd(Cl(allyl)]$_2$ (5 mol%), ^{t}BuXphos (30 mol%), NaOAc (3.0 equiv.), DMF (5.0 mL). The reaction mixture was stirred at 100 °C under N_2 for 4 h, and then quenched with saturated aqueous NaCl and

extracted with ethyl acetate. After drying over Na_2SO_4 for 30 min, the combined organic phase was concentrated, and the residue was purified by silica gel column chromatography with petroleum ether/ethyl acetate to afford the product **3ba** as a yellow oil. (560 mg, 83% yield)

The product **3ba** (0.1 mmol, 34 mg, 1.0 equiv.) was dissolved in DCM (2.0 mL), and *m*-CPBA (0.2 mmol, 35 mg, 2.0 equiv.) was added into the solution. The mixture was stirred at rt. When the reaction was completed as monitored by TLC, filtration through celite and removal of the solvent by rotary evaporation gave the crude product. The residue was purified by chromatography on silica gel with the appropriate mixture of PE and EA (PE = petroleumether, EA = ethyl acetate) to afford the product **3ba-1** as a colorless oil. (23.5 mg, 66% yield)

3. The preparation of the macrolactams

3.1 Synthesis of 6a

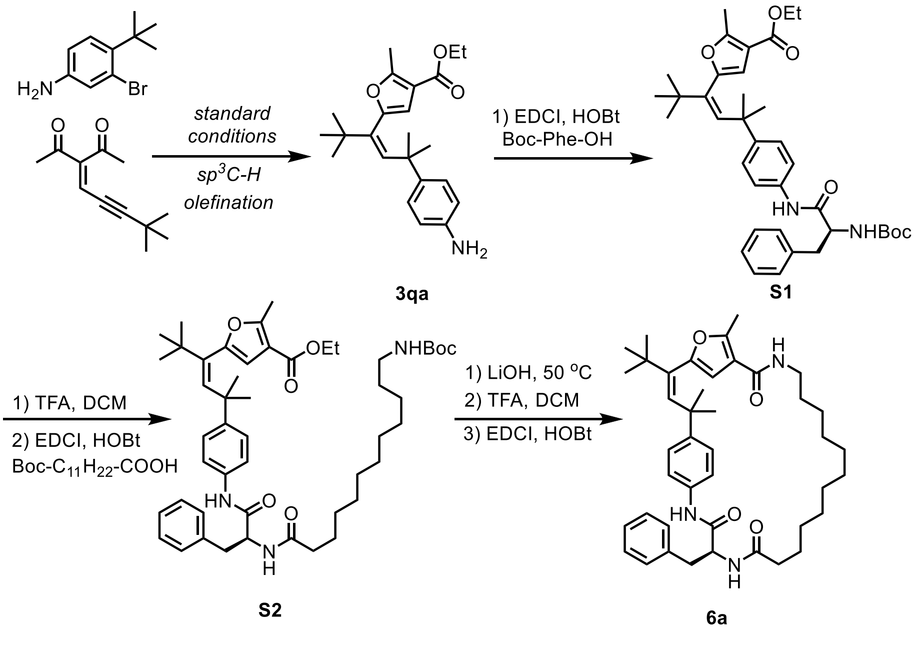

Synthesis of **3qa**:

Please refer to **3ba**

From **3qa** to **S1**:

To a solution of **3qa** (50 mg, 0.14 mmol) in DCM (2 mL) were added EDCI (51.5 mg, 0.27 mmol), HOBt (36.6 mg, 0.27 mmol), Boc-Phe-OH (42.4 mg, 0.16 mmol). The stirring of the solution was continued at r.t. for 18 h. Then the solution was washed with water, and the organic phase was evaporated under reduced pressure and the residue was purified by FCC to get the desired product **S1** (54.9 mg, yield: 66%).

From **S1** to **S2**:

The **S1** (54.9 mg, 0.09 mmol) was dissolved in DCM (2 mL) and TFA (0.2 mL) were added slowly. The stirring of the solution was continued at r.t. for 4 h. Then the solution was evaporating under reduced pressure. And the residue was used in the next step without further purification.

The residue was dissolved in DCM (5 mL). And EDCI (51.5 mg, 0.27 mmol), HOBt (36.6 mg, 0.27 mmol), Boc-NH-$(CH_2)_{11}$-COOH (56.7 mg, 0.18 mmol) were added. The stirring of the solution was continued at r.t. for 24 h. Then the solution was washed with water, and the organic phase was evaporated under reduced pressure and the residue was purified by FCC to get the desired product **S2** (54.2 mg, 74% yield in two steps from **S1**).

From **S2** to **6a**:

The **S2** (54.2 mg, 0.07 mmol) was dissolved in DCM (2 mL) and TFA (0.2 mL) were added slowly. The stirring of the solution was continued at r.t. for 4 h. Then the solution was evaporating under reduced pressure. And the residue was used in the next step without further purification.

The residue was dissolved in MeOH (2 mL) and the solution of NaOH (100 mg) in H_2O were added slowly. The stirring of the solution was continued at 50 ^{o}C for 4 h. Then the solution was acidified by HCl, followed by evaporating under reduced

pressure. And the residue was used in the next step without further purification.

The residue was dissolved in DCM (40 mL). And EDCI (76.4 mg, 0.4 mmol), HOBt (54.0 mg, 0.4 mmol) were added. The stirring of the solution was continued at r.t. for 24 h. Then the solution was washed with water, and the organic phase was evaporated under reduced pressure and the residue was purified by FCC to get the desired product **6a** as a white solid (11.8 mg, 27.2% yield in three steps from **S2**).

3.2 Synthesis of 6b

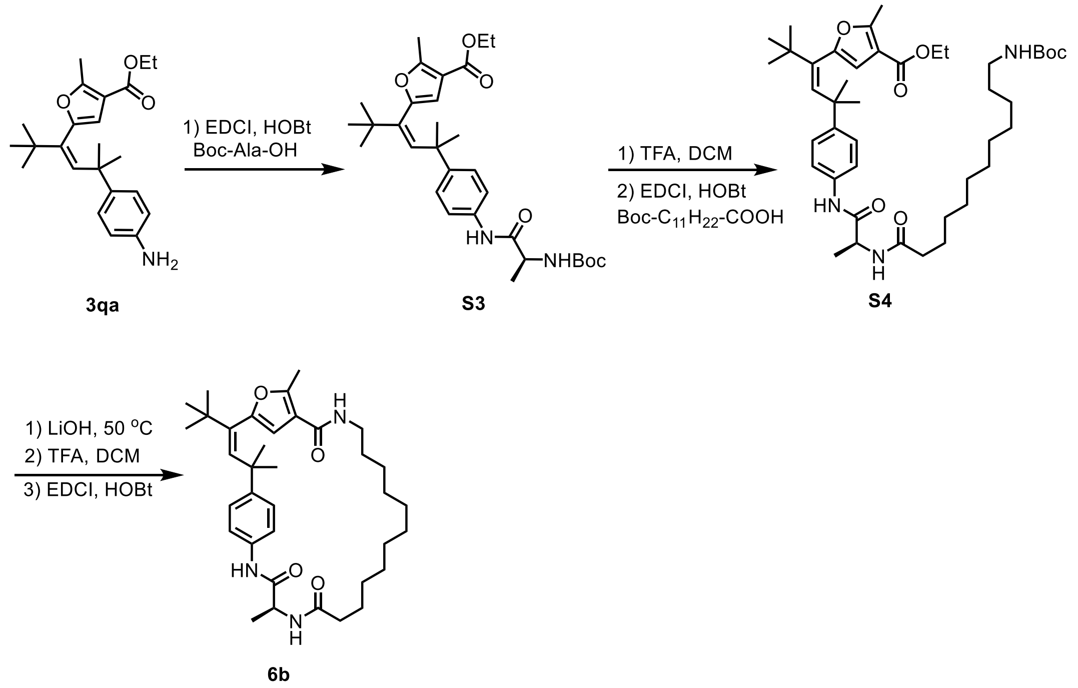

Synthesis of **6b**:

This compound was prepared form **3qa** and Boc-Ala-OH using the procedure described for **6b** and was isolated as a white solid, 2.5 mg, 2.9% yield in six steps from **3qa**.

3.3 Synthesis of 6c

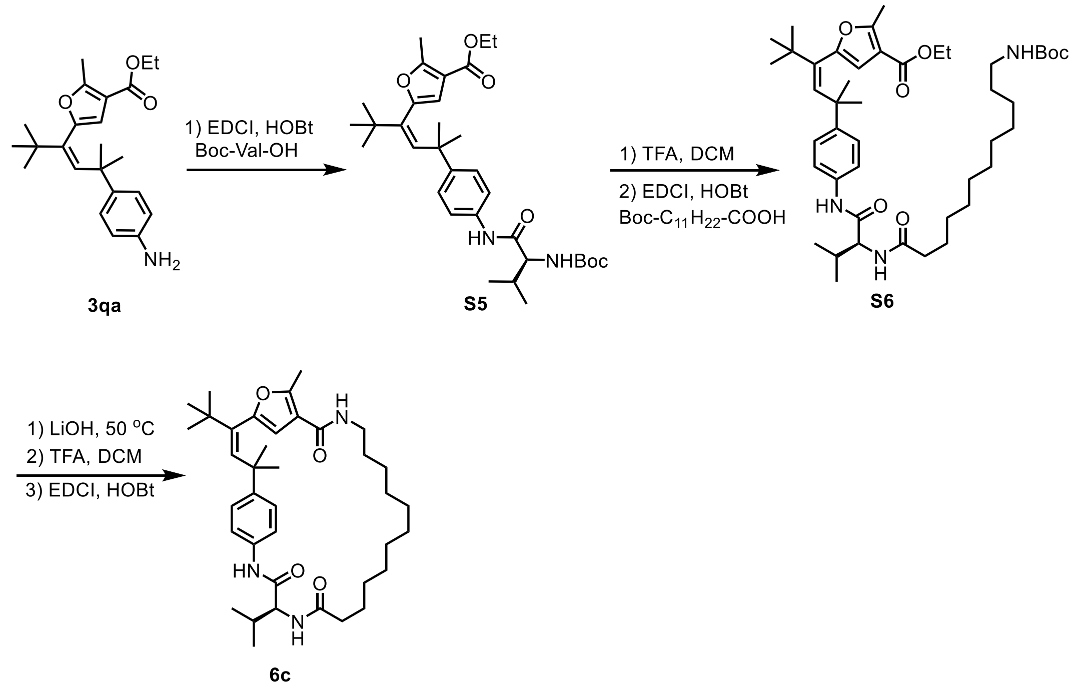

Synthesis of **6c**:

This compound was prepared form **3qa** and Boc-Val-OH using the procedure described for **6c** and was isolated as a white solid, 4.6 mg, 5.5% yield in six steps from **3qa**.

3.4 Synthesis of 6d

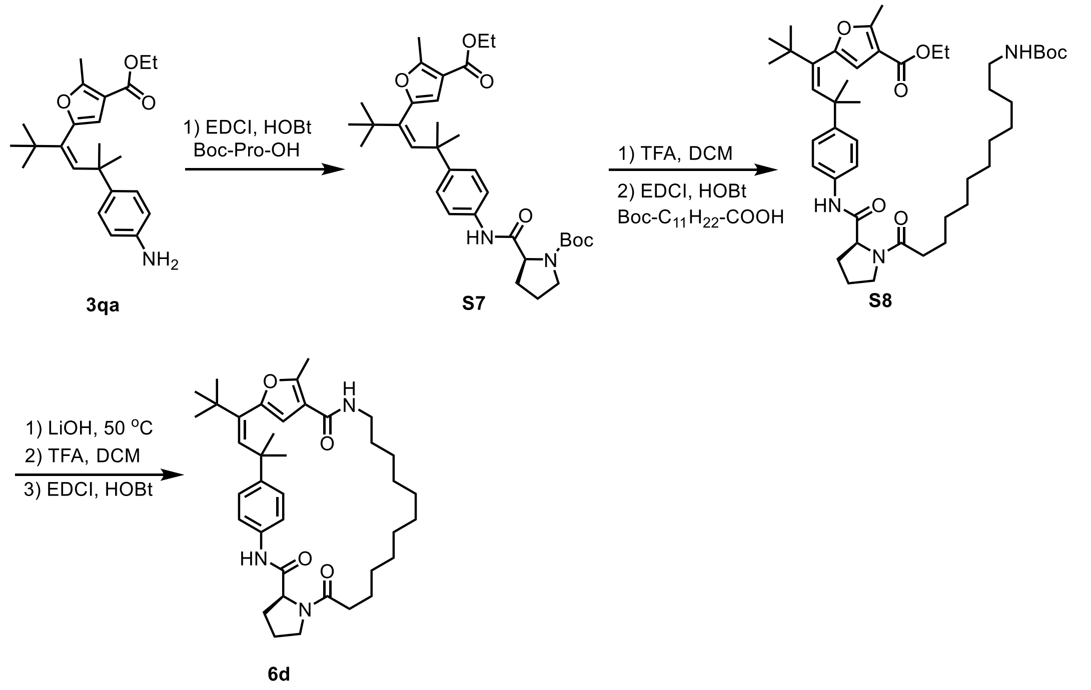

Synthesis of **6d**:

This compound was prepared form **3qa** and Boc-Pro-OH using the procedure described for **6d** and was isolated as a white solid, 9.1 mg, 10.9% yield in six steps from **3qa**.

3.5 Synthesis of 6e

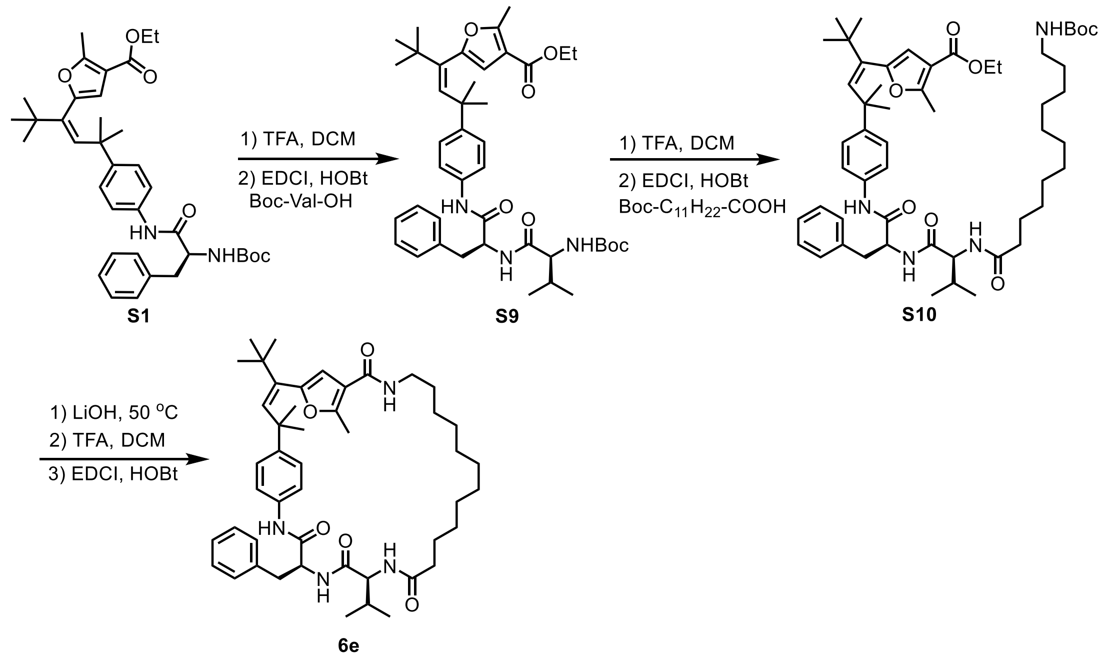

Synthesis of **6e**:

This compound was prepared form **S1** and Boc-Val-OH using the procedure described for **6e** and was isolated as a white solid, 4.1 mg, 6.1% yield in seven steps from **S1**.

3.6 Synthesis of 6f

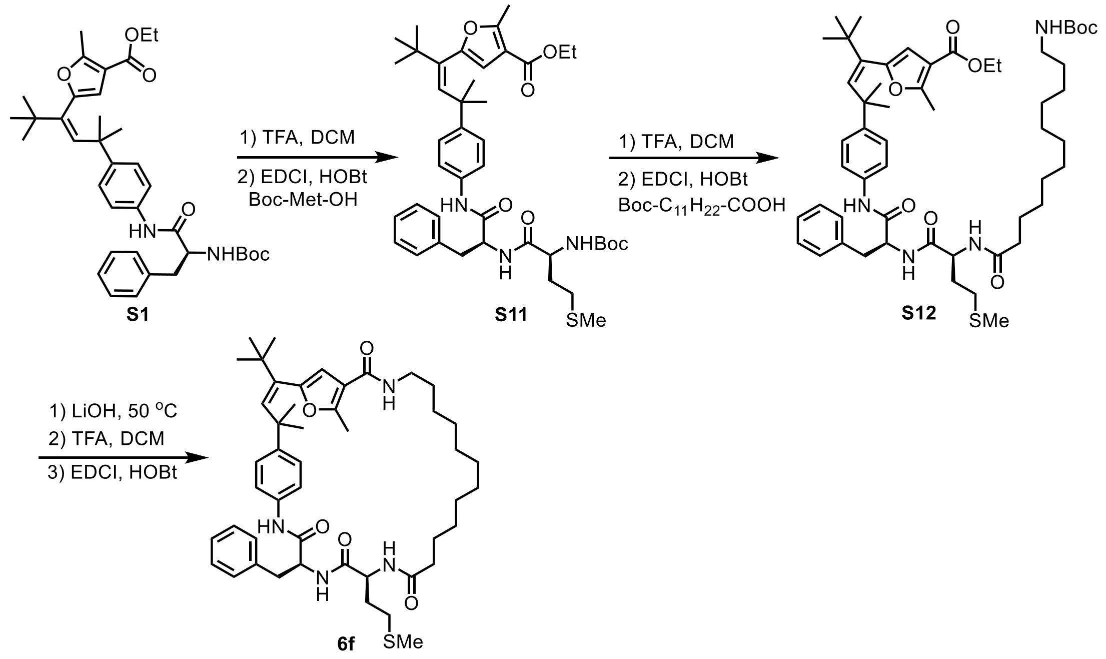

Synthesis of **6f**:

This compound was prepared form **S1** and Boc-Met-OH using the procedure described for **6f** and was isolated as a white solid, 2.8 mg, 3.6% yield in seven steps from **S1**.

3.7 Synthesis of 6g

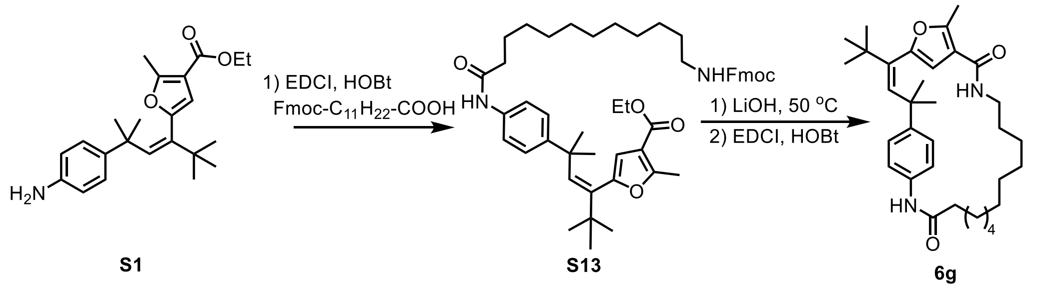

From **S1** to **S13**:

To a solution of **S1** (100 mg, 0.27 mmol) in DCM (2 mL) were added EDCI (103 mg, 0.54 mmol), HOBt (73.2 mg, 0.54 mmol), Fmoc-NH-$(CH_2)_{11}$-COOH (141.6 mg, 0.32 mmol). The stirring of the solution was continued at r.t. for 18 h. Then the solution was washed with water, and the organic phase was evaporated under reduced pressure and the residue was purified by FCC to get the desired product **S13** (131.2 mg, yield:

64%).

From **S13** to **6g**:

The **S13** (100 mg) was dissolved in MeOH (2 mL) and the solution of NaOH (100 mg) in H_2O were added slowly. The stirring of the solution was continued at 50 °C for 4 h. Then the solution was acidified by HCl, followed by evaporating under reduced pressure. And the residue was used in the next step without further purification.

The residue was dissolved in DCM (40 mL). And EDCI (95.5 mg, 0.5 mmol), HOBt (67.5 mg, 0.5 mmol) were added. The stirring of the solution was continued at r.t. for 24 h. Then the solution was washed with water, and the organic phase was evaporated under reduced pressure and the residue was purified by FCC to get the desired product **6g** (17.8 mg, yield: 27%).

3.8 Synthesis of 6h

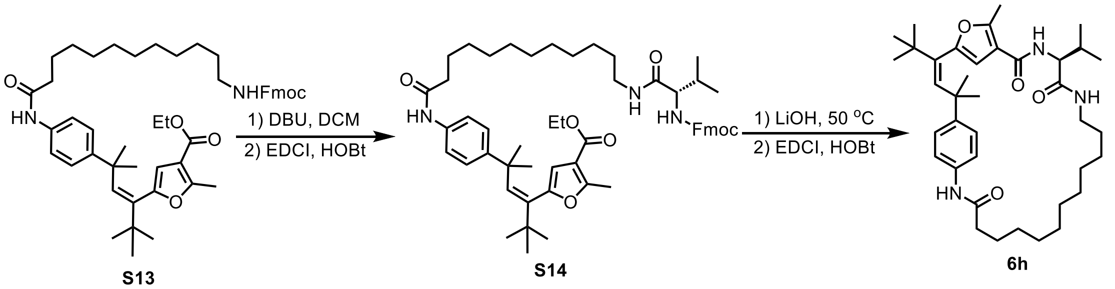

From **S13** to **S14**:

To a solution of **S13** in DCM were added DBU (10 eq) slowly and stirring of the solution was continued at r.t. for 1 h. Then the solution was washed with water and extracted by EtOAc. Then the organic phase was evaporated under reduced pressure and the residue was purified by FCC (DCM/EtOH = 2:1).

The product purified was dissolved in DCM. And EDCI (3 eq), HOBt (3 eq) were added followed by (((9H-fluoren-9-yl)methoxy)carbonyl)valine. The stirring of the solution was continued at r.t. for 18 h. Then the solution was washed with water, and

the organic phase was evaporated under reduced pressure and the residue was purified by FCC (DCM/MeOH = 1% - 5%) to get the desired product **S14**.

From **S14** to **6h**:

The **S14** was dissolved in MeOH and the solution of 10% LiOH in H_2O were added. The stirring of the solution was continued at 50 °C for 4 h. Then the pH of solution was adjusted to 7-8. Then the solvent was evaporated under reduced pressure. And the residue was used in the next step without further purification.

The residue was dissolved in DCM. And EDCI (3 eq), HOBt (3 eq) were added. The stirring of the solution was continued at r.t. for 24 h. Then the solution was washed with water and extracted by EtOAc. Then the organic phase was evaporated under reduced pressure and the residue was purified by FCC to get the desired product **6h**.

4. The DFT calculated details

Density functional theory (DFT) calculations were carried out at the M06 level of theory.[6]All the molecular geometries were fully optimized without constraints in DMF (n, n-dimethylformamide) solvent by employing the SMD model.[7] Frequency calculations at the same level of theory have also been performed to identify all the stationary points as minima (zero imaginary frequencies) or transition states (one imaginary frequency) and to provide free energies at 298.15K, which include entropic contributions. Transition states were located using the Berny algorithm. Intrinsic reaction coordinates (IRC) analysis[8,9] were calculated at the same level of theory as geometry optimization to confirm these transition states indeed connect two relevant minima. The Stuttgart-Dresden-Bonn basis set[10-15]was used to describe Pd and P atoms, with polarization functions for Pd (ζ_f = 1.472), and P (ζ_d = 0.387) being added.[16] The 6-31G**

basis set was used for the other atoms. All calculations were performed using Gaussian 09 package.[17]

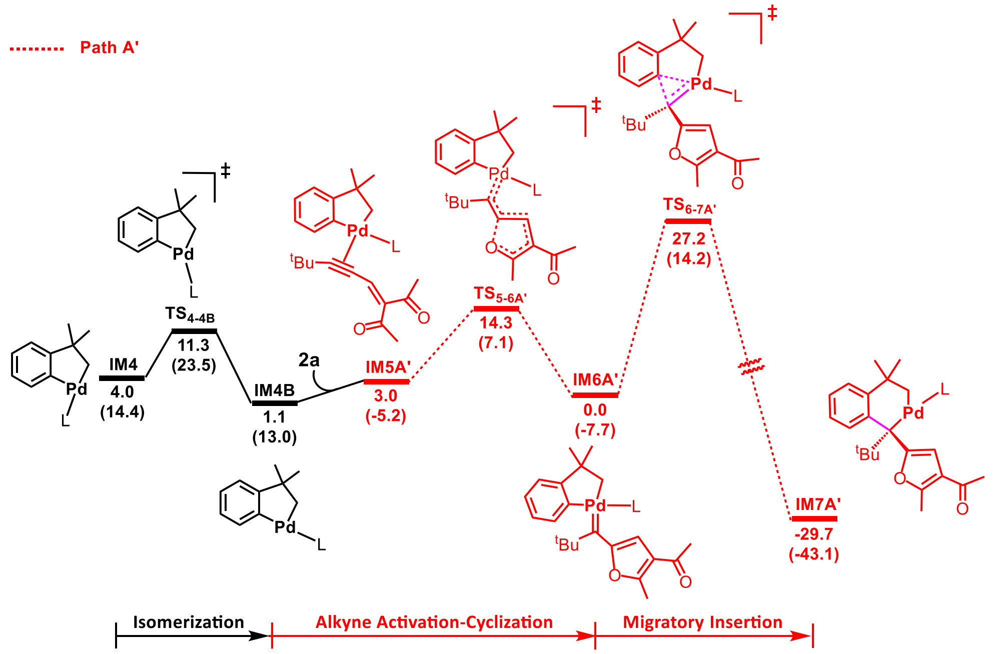

Supplementary Figure 1. Energy profiles calculated for the path A′ which is similar with Path A starting from **IM4B**. The solvation-corrected relative free energies and electronic energies (in parentheses) are given in kcal/mol.

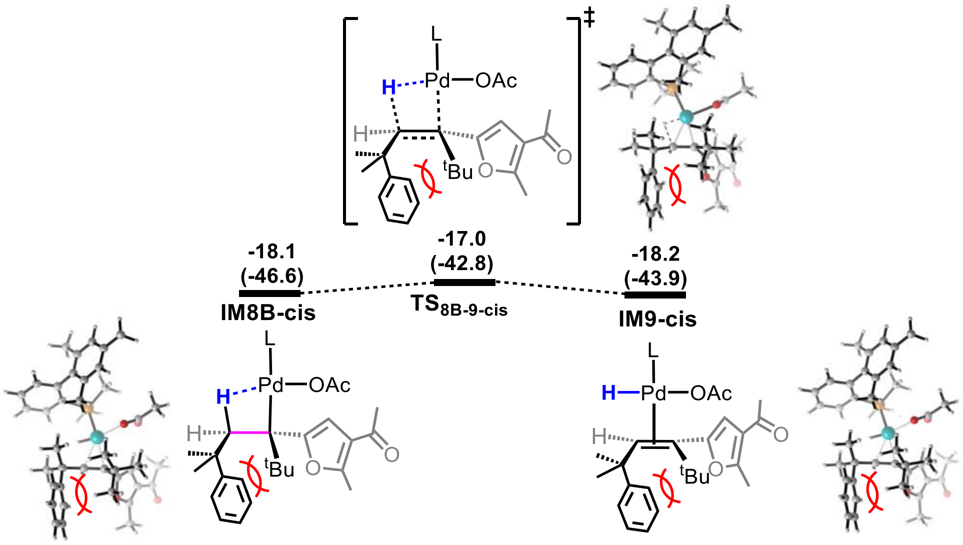

Supplementary Figure 2. Energy profile calculated for the *β*-Hydride elimination from a highly unstable isomeric agostic species (*cf.* **IM8B** in Figure 3(c) in the main text). The solvation-corrected relative free energies and electronic energies (in parentheses) are given in kcal/mol.

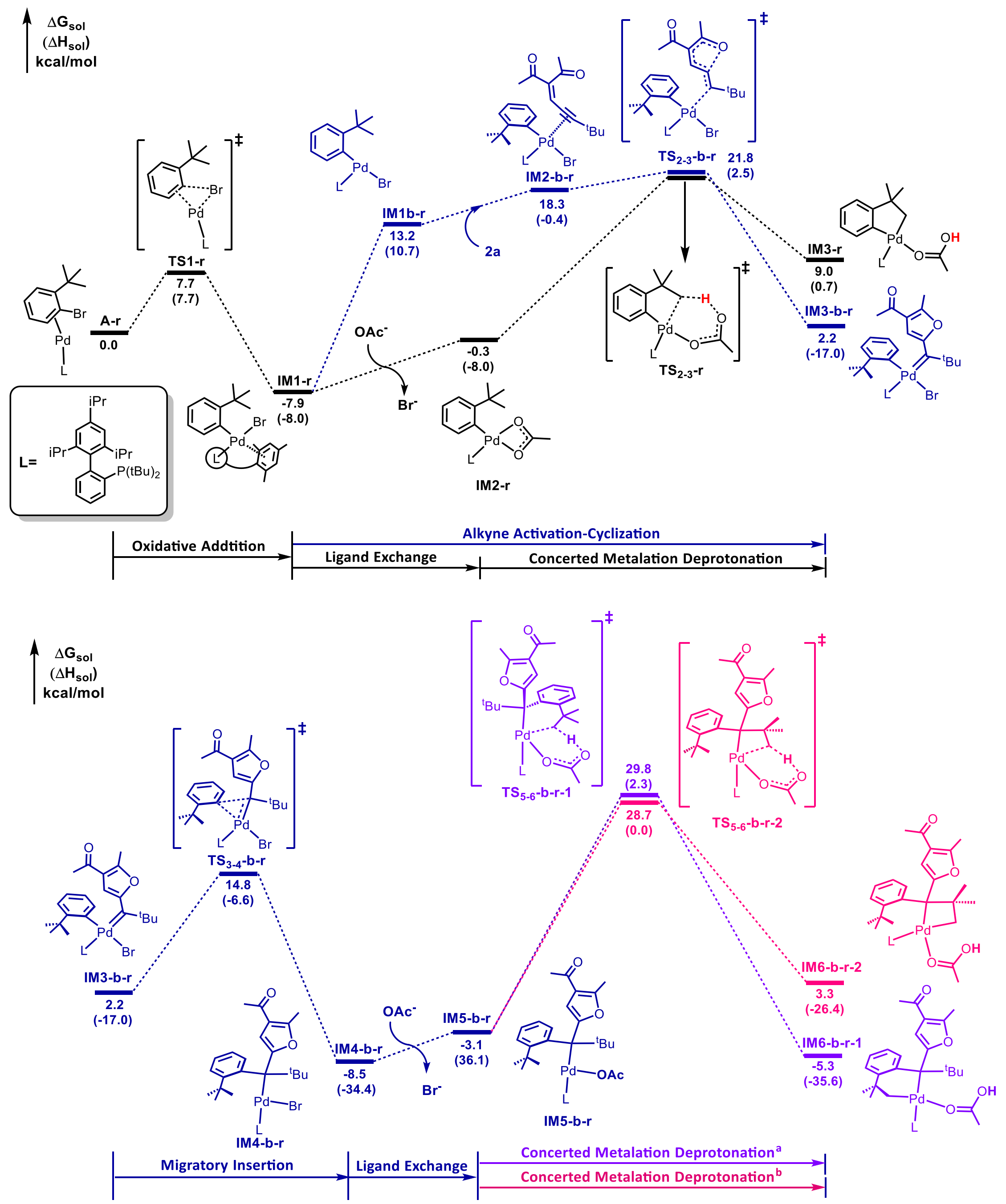

Supplementary Figure 3. Energy profiles calculated (using the experimentally employed ligand) for an alternative pathway considering an alkyne activation cyclization immediately after oxidative addition. The results here indicate that such an alkyne activation cyclization is reversible in view of the inaccessibly high lying **TS$_{5\text{-}6}$-b-r-1** and **TS$_{5\text{-}6}$-b-r-2**. The solvation-corrected relative free energies and electronic energies (in parentheses) are given in kcal/mol.

[a] through 6-membered ring

[b] through 4-membered ring

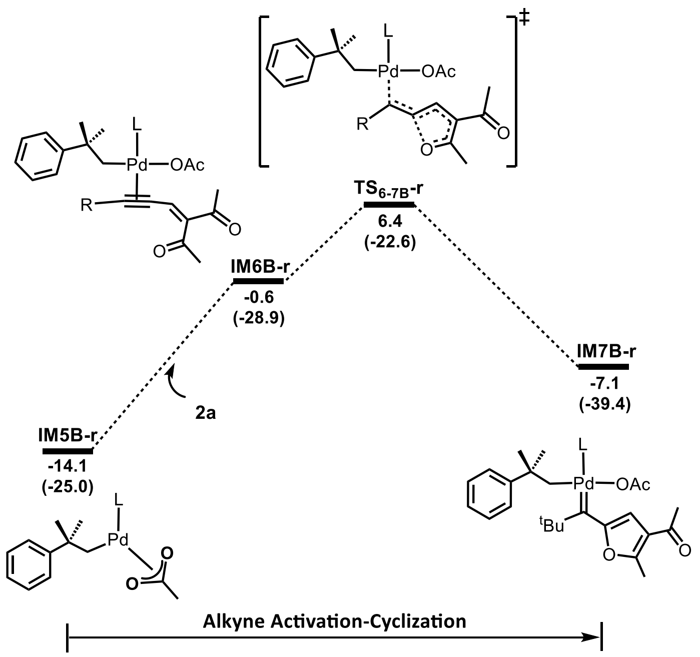

Supplementary Figure 4. Energy profile calculated for alkyne activation cyclization using the experimentally employed ligand. The barrier is compatible with that obtained by using the model ligand (*cf.* Figure 3(b) in the main text), but is much smaller than the corresponding step shown in Figure 3 (from **IM1-r** to **TS$_{2\text{-}3}$-b-r**). The solvation-corrected relative free energies and electronic energies (in parentheses) are given in kcal/mol.

Supplementary Table 1 Cytotoxicity (CC_{50}, μM) and anti-inflammatory effects (IC_{50}, μM) of selected compounds.

Compds	IC_{50}(μM)			CC_{50}(μM)
	TNF-α	IL-6	IL-1β	----
**6a**	>250	53.59	12.84	152.2
**6b**	2.15	7.28	3.40	39.67
**6c**	11.5	10.52	4.38	154
**6d**	107.1	195.7	17.55	27.67
**6e**	>250	80.78	3.05	>250
**6f**	10.03	29.46	8.61	214.5
**6g**	0.45	1.59	0.59	>250
**6h**	56.43	0.95	0.0041	56.43
**DEX**	0.008	10.54	0.0004	152.2

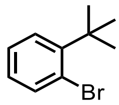

1a

1-bromo-2-(tert-butyl)benzene (1a): Colorless oil.

^{1}H NMR (400 MHz, $CDCl_3$) δ 7.58 (d, *J* = 7.9 Hz, 1H), 7.43 (d, *J* = 8.1 Hz, 1H), 7.23 (t, *J* = 7.6 Hz, 1H), 7.01 (t, *J* = 7.6 Hz, 1H), 1.51 (s, 9H).

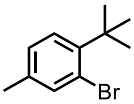

1b

2-bromo-1-(tert-butyl)-4-methylbenzene (1b): Colorless oil.

^{1}H NMR (400 MHz, $CDCl_3$) δ 7.45 (s, 1H), 7.34 (d, *J* = 8.0 Hz, 1H), 7.06 (d, *J* = 8.0 Hz, 1H), 2.30 (s, 3H), 1.51 (s, 9H).

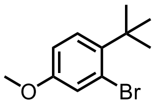

1c

2-bromo-1-(tert-butyl)-4-methoxybenzene (1c): Colorless oil.

^{1}H NMR (400 MHz, $CDCl_3$) δ 7.35 (d, *J* = 8.8 Hz, 1H), 7.17 (d, *J* = 2.7 Hz, 1H), 6.79 (dd, *J* = 8.8, 2.7 Hz, 1H), 3.78 (s, 3H), 1.49 (s, 9H).

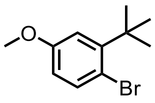

1d

1-bromo-2-(tert-butyl)-4-methoxybenzene (1d): Colorless oil.

^{1}H NMR (500 MHz, $CDCl_3$) δ 7.47 (d, *J* = 8.6 Hz, 1H), 7.01 (d, *J* = 3.1 Hz, 1H), 6.59 (dd, *J* = 8.7, 3.1 Hz, 1H), 3.78 (s, 3H), 1.49 (s, 9H).

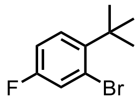

1e

2-bromo-1-(tert-butyl)-4-fluorobenzene (1e): Colorless oil.

^{1}H NMR (400 MHz, $CDCl_3$) δ 7.40 (dd, *J* = 9.0, 6.3 Hz, 1H), 7.34 (dd, *J* = 8.3, 2.7 Hz, 1H), 6.96 (ddd, *J* = 9.0, 7.8, 2.7 Hz, 1H), 1.50 (s, 9H).

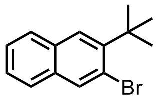

1f

2-bromo-3-(tert-butyl)naphthalene (1f): Colorless oil.

^{1}H NMR (400 MHz, $CDCl_3$) δ 8.55 (dt, *J* = 8.7, 0.9 Hz, 1H), 7.79 (dd, *J* = 10.0, 8.2 Hz, 2H), 7.67 (d, *J* = 8.8 Hz, 1H), 7.59 (ddd, *J* = 8.5, 6.8, 1.4 Hz, 1H), 7.50 (ddd, *J* = 7.9, 6.8, 1.2 Hz, 1H), 1.69 (s, 9H).

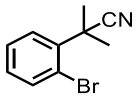

1g

2-(2-bromophenyl)-2-methylpropanenitrile (1g): Yellow oil.

^{1}H NMR (300 MHz, $CDCl_3$) δ 7.67 (dd, J_1 = 8.1 Hz, J_2 = 1.5 Hz, 1H), 7.48 (dd, J_1 = 7.8 Hz, J_2 = 1.5 Hz, 1H), 7.35 (td, J_1 = 7.5 Hz, J_2 = 1.2 Hz, 1H), 7.19 (td, J_1 = 7.8 Hz, J_2 = 1.8 Hz, 1H), 1.90 (s, 6H).

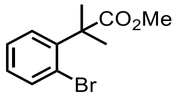

1h

methyl 2-(2-bromophenyl)-2-methylpropanoate (1h): Colorless oil.

^{1}H NMR (500 MHz, $CDCl_3$) δ 7.56 (dd, *J* = 7.9, 1.4 Hz, 1H), 7.42 (dd, *J* = 7.8, 1.7 Hz, 1H), 7.32 (td, *J* = 7.6, 1.4 Hz, 1H), 7.12 (td, *J* = 7.6, 1.7 Hz, 1H), 3.68 (s, 3H), 1.64 (s, 6H).

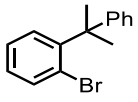

1i

1-bromo-2-(2-phenylpropan-2-yl)benzene (1i): Colorless oil.

^{1}H NMR (400 MHz, $CDCl_3$) δ 7.70 (dd, *J* = 7.9, 1.6 Hz, 1H), 7.54 (dd, *J* = 7.9, 1.4 Hz, 1H), 7.39 (td, *J* = 7.6, 1.4 Hz, 1H), 7.29 (td, *J* = 7.1, 6.3, 1.3 Hz, 2H), 7.24 – 7.19 (m, 1H), 7.19 – 7.10 (m, 3H), 1.80 (s, 6H).

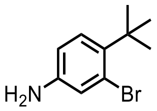

ij

3-bromo-4-(tert-butyl)aniline (ij): Colorless oil.

^{1}H NMR (400 MHz, $CDCl_3$) δ 7. 16 (d, *J*= 8.6 Hz, 1H), 6.97 (d, *J*= 3.0 Hz, 1H), 6.59 (dd, *J*= 8.4, 2.8 Hz, 1H), 1.43 (s, 9H).

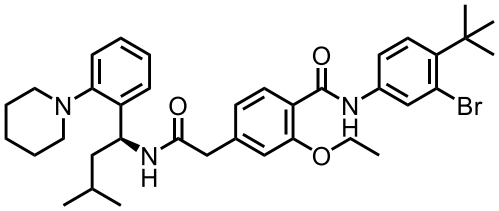

1k

(*S*)-N-(3-bromo-4-(tert-butyl)phenyl)-2-ethoxy-4-(2-((3-methyl-1-(2-(piperidin-1-yl)phenyl)butyl)amino)-2-oxoethyl)benzamide (1k): White solid.

^{1}H NMR (500 MHz, $CDCl_3$) δ 10.00 (s, 1H), 8.18 (d, *J* = 8.0 Hz, 1H), 7.95 (d, *J* = 2.4 Hz, 1H), 7.51 (dd, *J* = 8.7, 2.4 Hz, 1H), 7.39 (d, *J* = 8.7 Hz, 1H), 7.24 – 7.15 (m, 2H), 7.11 (dd, *J* = 7.6, 1.5 Hz, 1H), 7.04 (ddd, *J* = 7.9, 6.4, 2.0 Hz, 1H), 7.00 – 6.88 (m, 3H), 5.38 (td, *J* = 8.7, 6.4 Hz, 1H), 4.22 – 4.03 (m, 2H), 3.54 (s, 2H), 2.94 (t, *J* = 9.0 Hz, 2H), 2.61 (t, *J* = 9.1 Hz, 2H), 1.72 (q, *J* = 6.9, 6.4 Hz, 2H), 1.64 – 1.52 (m, 9H), 1.50 (s, 9H), 1.44 (dt, *J* = 13.4, 6.7 Hz, 1H), 0.92 (dd, *J* = 6.5, 2.1 Hz, 6H).

^{13}C NMR (126 MHz, $CDCl_3$) δ 168.49, 162.73, 156.58, 152.29, 143.07, 141.14, 138.46, 137.02, 132.50, 127.90, 127.68, 127.55, 126.51, 124.85, 122.60, 122.34, 122.02, 119.77, 118.32, 112.85, 64.86, 49.65, 46.43, 43.76, 36.04, 35.50, 29.85, 29.59, 26.54, 25.11, 23.89, 22.55, 22.30, 14.59.

HRMS (ESI): $C_{37}H_{49}BrN3O_3^{+}$, calcd. 662.2952, found 662.2961.

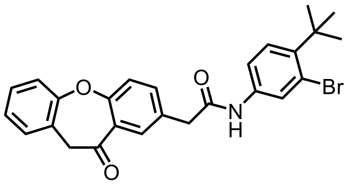

1l

N-(3-bromo-4-(tert-butyl)phenyl)-2-(11-oxo-10,11-dihydrodibenzo[b,f]oxepin-2-yl)acetamide (1l): White solid.

^{1}H NMR (500 MHz, $CDCl_3$) δ 8.16 (d, *J* = 2.4 Hz, 1H), 7.87 (dd, *J* = 7.8, 1.3 Hz, 1H), 7.65 (d, *J* = 2.6 Hz, 2H), 7.56 (td, *J* = 7.5, 1.4 Hz, 1H), 7.52 – 7.43 (m, 2H), 7.41 – 7.34 (m, 2H), 7.29 (d, *J* = 8.6 Hz, 1H), 7.05 (d, *J* = 8.4 Hz, 1H), 5.18 (s, 2H), 3.72 (s, 2H), 1.44 (s, 9H).

^{13}C NMR (126 MHz, $CDCl_3$) δ 191.01, 169.09, 160.79, 143.78, 140.31, 136.43, 135.53, 132.97, 132.49, 129.50, 129.36, 128.23, 128.07, 127.92, 126.69, 125.30, 122.36, 121.71, 118.63, 73.65, 43.49, 36.26, 29.76.

HRMS (ESI): $C_{26}H_{25}BrNO_3^+$, calcd. 478.1012, found 478.1016.

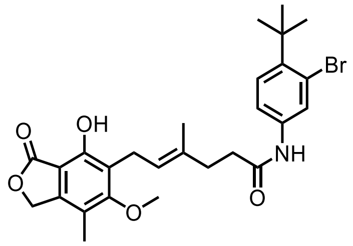

1m

(*E*)-N-(3-bromo-4-(tert-butyl)phenyl)-6-(4-hydroxy-6-methoxy-7-methyl-3-oxo-1,3-dihydroisobenzofuran-5-yl)-4-methylhex-4-enamide (1m): White solid.

^{1}H NMR (500 MHz, $CDCl_3$) δ 7.67 (s, 1H), 7.63 (t, *J* = 1.3 Hz, 1H), 7.38 (d, *J* = 11.0 Hz, 1H), 7.30 (d, *J* = 1.3 Hz, 2H), 5.40 – 5.25 (m, 1H), 5.17 (s, 2H), 3.74 (s, 3H), 3.41 (d, *J* = 6.9 Hz, 2H), 2.51 – 2.32 (m, 4H), 2.11 (s, 3H), 1.88 – 1.73 (m, 3H), 1.47 (s, 9H).

^{13}C NMR (126 MHz, $CDCl_3$) δ 172.94, 171.07, 163.65, 153.54, 144.13, 143.48, 136.55, 134.44, 128.00, 126.66, 123.42, 122.32, 121.91, 118.61, 116.85, 106.40, 70.09, 61.04, 36.26, 35.84, 34.98, 29.81, 29.79, 22.67, 16.23, 11.57.

HRMS (ESI): $C_{27}H_{33}BrNO_5^+$, calcd. 530.1537, found 530.1539.

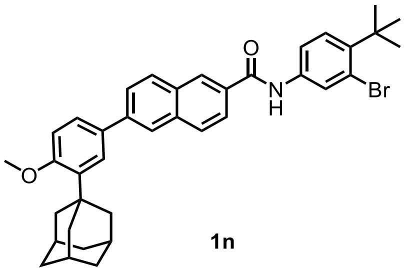

1n

6-(3-((3r,5r,7r)-adamantan-1-yl)-4-methoxyphenyl)-N-(3-bromo-4-(tert-butyl)phenyl)-2-naphthamide (1n): White solid.

^{1}H NMR (500 MHz, $CDCl_3$) δ 8.39 – 8.30 (m, 2H), 7.98 (dd, *J* = 8.7, 2.0 Hz, 2H), 7.92 – 7.85 (m, 3H), 7.78 (dd, *J* = 8.5, 1.8 Hz, 1H), 7.69 (dd, *J* = 8.6, 2.4 Hz, 1H), 7.63 (d, *J* = 2.4 Hz, 1H), 7.53 (dd, *J* = 8.3, 2.3 Hz, 1H), 7.43 (d, *J* = 8.7, 1H), 7.01 (d, *J* = 8.5 Hz, 1H), 3.93 (s, 3H), 2.23 (d, J = 2.9 Hz, 6H), 2.20 – 2.12 (m, 3H), 1.86 (d, J = 3.1 Hz, 6H), 1.55 (s, 9H).

^{13}C NMR (126 MHz, $CDCl_3$) δ 166.11, 158.93, 143.85, 141.05, 139.02, 136.86, 135.32, 132.49, 131.27, 131.23, 129.36, 128.74, 128.22, 127.53, 127.18, 126.67, 125.94, 125.74, 124.67, 123.90, 122.54, 119.10, 112.15, 55.20, 40.66, 37.24, 37.18, 36.36, 29.87, 29.16.

HRMS (ESI): $C_{38}H_{41}BrNO_2^+$, calcd. 622.2315, found 622.2308.

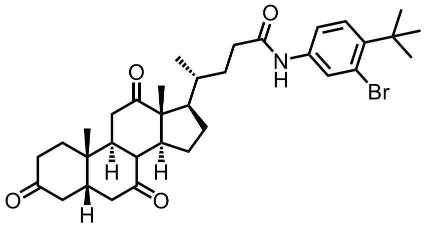

1o

(4*R*)-N-(3-bromo-4-(tert-butyl)phenyl)-4-((5*S*,9*S*,10*S*,13*R*,14*S*,17*R*)-10,13-dimethyl-3,7,12-trioxohexadecahydro-1H-cyclopenta[a]phenanthren-17-yl)pentanamide (1l): White solid.

^{1}H NMR (500 MHz, $CDCl_3$) δ 8.43 (s, 1H), 7.75 (d, J = 2.3 Hz, 1H), 7.47 (dd, J = 8.6, 2.4 Hz, 1H), 7.32 (d, J = 8.8 Hz, 1H), 3.01 – 2.79 (m, 3H), 2.44 (ddd, J = 14.1, 10.1, 4.5 Hz, 2H), 2.38 – 2.23 (m, 4H), 2.24 – 2.16 (m, 3H), 2.10 (dd, J = 12.4, 5.0 Hz, 1H), 2.06 – 1.88 (m, 5H), 1.82 (td, J = 11.3, 7.1 Hz, 1H), 1.60 (td, J = 14.6, 4.4 Hz, 1H), 1.46 (s, 9H), 1.39 (s, 3H), 1.37 – 1.21 (m, 4H), 1.05 (s, 3H), 0.83 (d, J = 6.6 Hz, 3H).

13C NMR (126 MHz, CDCl3) δ 212.28, 209.41, 209.17, 172.05, 143.21, 137.04, 128.07, 126.51, 122.34, 118.48, 56.92, 51.79, 48.99, 46.81, 45.43, 45.35, 45.02, 42.81, 38.69, 36.46, 36.22, 36.01, 35.28, 35.25, 34.02, 30.76, 29.82, 27.63, 25.16, 21.85, 18.76, 11.88.

HRMS (ESI): $C_{34}H_{47}BrNO_4^+$, calcd. 612.2683, found 612.2682.

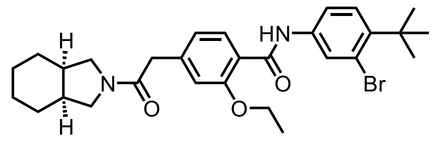

1p

N-(3-bromo-4-(tert-butyl)phenyl)-2-ethoxy-4-(2-((3aR,7aS)-octahydro-2H-isoindol-2-yl)-2-oxoethyl)benzamide (1p): Yellow solid.

^{1}H NMR (500 MHz, $CDCl_3$) δ 10.04 (s, 1H), 8.21 (d, *J* = 8.0 Hz, 1H), 7.94 (d, *J* = 2.4 Hz, 1H), 7.54 (dd, *J* = 8.6, 2.3 Hz, 1H), 7.40 (d, *J* = 8.7 Hz, 1H), 7.05 (d, *J* = 1.6 Hz, 1H), 6.98 (dd, *J* = 8.0, 1.6 Hz, 1H), 4.29 (q, *J* = 7.0 Hz, 2H), 3.67 (s, 2H), 3.53 – 3.34 (m, 3H), 3.31 (dd, J = 9.9, 5.9 Hz, 1H), 2.30 – 2.11 (m, 2H), 1.61 (t, J = 7.1 Hz, 7H), 1.51 (s, 9H), 1.42 – 1.33 (m, 4H).

^{13}C NMR (126 MHz, $CDCl_3$) δ 169.29, 163.10, 156.85, 143.28, 140.96, 137.34, 132.61, 128.15, 126.79, 122.59, 122.42, 119.99, 118.60, 113.14, 65.22, 51.05, 49.84, 41.98, 37.68, 36.30, 35.92, 29.86, 25.72, 22.74, 22.53, 14.91.

HRMS (ESI): $C_{29}H_{38}BrN_2O_3^+$, calcd.541.2060, found.541.2053

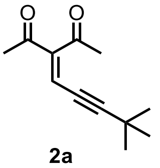

2a

3-(4,4-dimethylpent-2-yn-1-ylidene)pentane-2,4-dione (2a): Yellow oil.
^{1} **H NMR** (400 MHz, CDCl3) δ 6.72 (s, 1H), 2.49 (s, 3H), 2.31 (s, 3H), 1.27 (s, 9H).

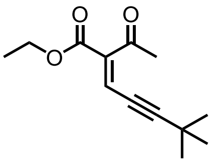

2b

ethyl (E)-2-acetyl-6,6-dimethylhept-2-en-4-ynoate (2b): Colorless oil.
^{1}**H NMR** (400 MHz, $CDCl_3$) δ 6.81 (s, 1H), 4.34 (q, J = 7.1, 2H), 2.46 (s, 3H), 1.38 (t, J = 7.1, 3H), 1.29 (s, 9H).

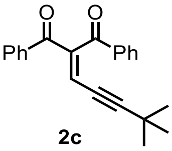

2c

2-(4,4-dimethylpent-2-yn-1-ylidene)-1,3-diphenylpropane-1,3-dione (2c): White solid.
^{1}**H NMR** (400 MHz, $CDCl_3$) δ 8.05 – 7.97 (m, 2H), 7.84 – 7.77 (m, 2H), 7.67 – 7.40 (m, 6H), 6.76 (s, 1H), 0.97 (s, 9H).

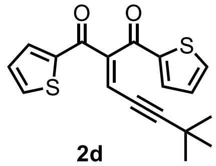

2d

2-(4,4-dimethylpent-2-yn-1-ylidene)-1,3-di(thiophen-2-yl)propane-1,3-dione (2d): Yellow solid.
^{1}**H NMR** (500 MHz, $CDCl_3$) δ 7.77 – 7.72 (m, 2H), 7.68 – 7.64 (m, 2H), 7.15 (dd, J = 4.8, 4.0 Hz, 1H), 7.08 (dd, J = 4.9, 3.9 Hz, 1H), 6.89 (s, 1H), 1.04 (s, 9H).

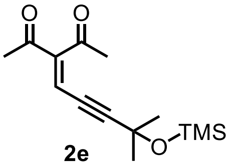

2e

3-(4-methyl-4-((trimethylsilyl)oxy)pent-2-yn-1-ylidene)pentane-2,4-dione (2e): Colorless oil.
^{1}**H NMR** (400 MHz, $CDCl_3$) δ 6.73 (s, 1H), 2.49 (s, 3H), 2.34 (s, 3H), 1.54 (s, 6H), 0.19 (s, 9H).

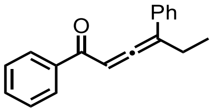

4a

1,4-diphenylhexa-2,3-dien-1-one (4a): Yellow oil.
^{1}H NMR (500 MHz, $CDCl_3$) δ 7.91 – 7.82 (m, 2H), 7.54 – 7.45 (m, 1H), 7.44 – 7.32 (m, 6H), 7.31 – 7.26 (m, 1H), 6.71 (t, *J* = 3.3 Hz, 1H), 2.64 – 2.52 (m, 2H), 1.15 (t, *J* = 7.4 Hz, 3H).

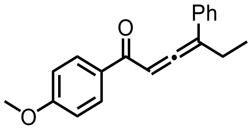

4b

1-(4-methoxyphenyl)-4-phenylhexa-2,3-dien-1-one (4b): Yellow oil.
^{1}H NMR (400 MHz, $CDCl_3$) δ 7.96 – 7.92 (m, 2H), 7.47 – 7.29 (m, 6H), 6.91 – 6.86 (m, 2H), 6.73 (t, *J* = 3.4 Hz, 1H), 3.86 (s, 3H), 2.65 – 2.56 (m, 2H), 1.20 (t, *J* = 7.3 Hz, 3H).

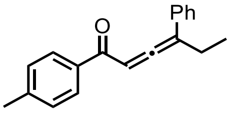

4c

4-phenyl-1-(p-tolyl)hexa-2,3-dien-1-one (4c): Yellow oil.
^{1}H NMR (400 MHz, $CDCl_3$) δ 7.96 – 7.92 (m, 2H), 7.47 – 7.29 (m, 6H), 6.91 – 6.86 (m, 2H), 6.73 (t, *J* = 3.4 Hz, 1H), 2.65 – 2.56 (m, 2H), 2.38 (s, 3H), 1.20 (t, *J* = 7.3 Hz, 3H).

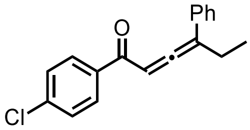

4d

1-(4-chlorophenyl)-4-phenylhexa-2,3-dien-1-one (4d): Yellow oil.
^{1}H NMR (400 MHz, $CDCl_3$) δ 7.88 – 7.74 (m, 2H), 7.42 – 7.38 (m, 3H), 7.37 (d, *J* = 2.1 Hz, 1H), 7.35 – 7.29 (m, 3H), 6.68 (t, *J* = 3.4 Hz, 1H), 2.65 – 2.56 (m, 2H), 1.17 (t, *J* = 7.3 Hz, 3H).

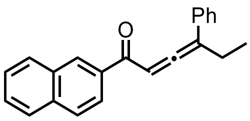

4e

1-(naphthalen-2-yl)-4-phenylhexa-2,3-dien-1-one (4e): Yellow oil.

^{1}H NMR (400 MHz, $CDCl_3$) δ 8.39 (t, *J* = 1.1 Hz, 1H), 7.97 (dd, *J* = 8.6, 1.8 Hz, 1H), 7.86 (d, *J* = 8.5 Hz, 2H), 7.64 (d, *J* = 8.1 Hz, 1H), 7.58 (ddd, *J* = 8.2, 6.8, 1.3 Hz, 1H), 7.52 – 7.39 (m, 5H), 7.38 – 7.31 (m, 1H), 6.80 (t, *J* = 3.3 Hz,1H), 2.65 – 2.56 (m, 2H),1.18 (t, *J* = 7.3 Hz, 3H).

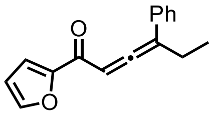

4f

1-(furan-2-yl)-4-phenylhexa-2,3-dien-1-one (4f): Yellow oil.
^{1}H NMR (400 MHz, $CDCl_3$) δ 7.61 (dd, *J* = 1.7, 0.8 Hz, 1H), 7.48 – 7.44 (m, 2H), 7.42 – 7.36 (m, 2H), 7.36 – 7.29 (m, 1H), 7.23 (dd, *J* = 3.6, 0.8 Hz, 1H), 6.69 (t, *J* = 3.5 Hz, 1H), 6.51 (dd, *J* = 3.6, 1.7 Hz, 1H), 2.65 – 2.56 (m, 2H), 1.23 (t, *J* = 7.3 Hz,3H).

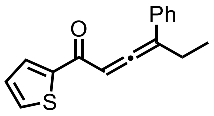

4g

4-phenyl-1-(thiophen-2-yl)hexa-2,3-dien-1-one (4g): Yellow oil.
^{1}H NMR (400 MHz, $CDCl_3$) δ 7.85 (dd, *J* = 3.8, 1.2 Hz, 1H), 7.60 (dd, *J* = 5.0, 1.2 Hz, 1H), 7.51 – 7.46 (m, 2H), 7.44 – 7.36 (m, 2H), 7.36 – 7.28 (m, 1H), 7.09 (dd, *J* = 5.0, 3.8 Hz, 1H), 6.65 (t, *J* = 3.6 Hz, 1H), 2.65 – 2.56 (m, 2H),1.24 (t, *J* = 7.3 Hz, 3H).

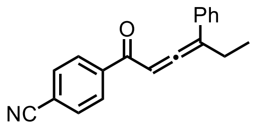

4h

4-(4-phenylhexa-2,3-dienoyl)benzonitrile (4h): Yellow oil.
^{1}H NMR (400 MHz, $CDCl_3$) δ 7.92 – 7.86 (m, 2H), 7.69 – 7.64 (m, 2H), 7.47 – 7.30 (m, 5H), 6.66 (t, *J* = 3.4 Hz, 1H), 2.64 – 2.52 (m, 2H), 1.14 (t, *J* = 7.3 Hz, 3H).

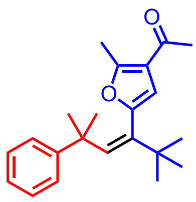

3aa

(Z)-1-(2-methyl-5-(2,2,5-trimethyl-5-phenylhex-3-en-3-yl)furan-3-yl)ethan-1-one (3aa): Yellow oil, 24.6 mg.

¹H NMR (400 MHz, $CDCl_3$) δ 7.22 – 7.06 (m,5H), 6.12 (s, 1H), 5.64 (s, 1H), 2.44 (s, 3H), 2.22 (s, 3H), 1.32 (s, 6H), 1.07 (s, 9H).

¹³C NMR (101 MHz, $CDCl_3$) δ 194.34, 156.32, 150.13, 148.68, 141.68, 138.89, 127.58, 126.17, 125.03, 121.59, 109.99, 40.19, 36.38, 30.91, 29.62, 28.92, 14.29

HRMS (ESI): $C_{22}H_{29}O_2^+$, calcd. 325.2162, found 325.2171.

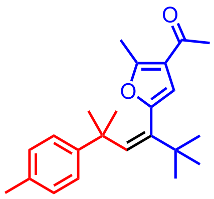

3ba

(Z)-1-(2-methyl-5-(2,2,5-trimethyl-5-(p-tolyl)hex-3-en-3-yl)furan-3-yl)ethan-1-one (3ba) Yellow oil, 27.7 mg.

¹H NMR (400 MHz, $CDCl_3$) δ 7.02 (q, J = 8.3 Hz, 4H), 6.09 (s, 1H), 5.65 (s, 1H), 2.45 (s,3H), 2.29 (s, 3H), 2.23 (s, 3H), 1.30 (s, 6H), 1.06 (s, 9H)

¹³C NMR (126 MHz, $CDCl_3$) δ 194.41, 156.42, 148.74, 147.18, 141.77, 138.61, 134.51, 128.30, 126.10, 121.57, 109.97, 39.90, 36.38, 30.85, 29.62, 28.86, 20.84, 14.29

HRMS (ESI): $C_{23}H_{31}O_2^+$, calcd. 339.2319, found 339.2321.

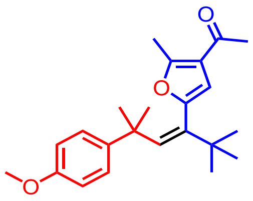

3ca

(Z)-1-(5-(5-(4-methoxyphenyl)-2,2,5-trimethylhex-3-en-3-yl)-2-methylfuran-3-yl)ethan-1-one (3ca) Yellow solid, 32.9 mg.

¹H NMR (400 MHz, $CDCl_3$) δ 7.05 (d, J = 8.8 Hz, 2H), 6.72 (d, J = 8.8 Hz, 2H), 6.07 (s, 1H), 5.68 (s, 1H), 3.76 (s, 3H), 2.46 (s, 3H), 2.24 (s, 3H), 1.30 (s, 6H), 1.06 (s, 9H).

¹³C NMR (126 MHz, $CDCl_3$) δ 194.40, 157.21, 156.37, 148.76, 142.15, 141.84, 138.57, 127.14, 121.68, 112.87, 109.82, 55.14, 39.62, 36.36, 30.95, 29.60, 28.88, 14.29

HRMS (ESI): $C_{23}H_{31}O_3^+$, calcd. 355.2268, found 355.227.

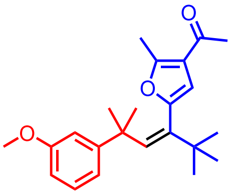

3da

(Z)-1-(5-(5-(3-methoxyphenyl)-2,2,5-trimethylhex-3-en-3-yl)-2-methylfuran-3-yl)ethan-1-one (3da) Yellow solid, 34.3 mg.

^{1}H NMR (400 MHz, $CDCl_3$) δ 7.10 (t, J = 7.9 Hz, 1H), 6.76 (ddd, J = 7.8, 1.7, 0.9 Hz, 1H), 6.69 (t, J = 2.2 Hz, 1H), 6.66 – 6.61 (m, 1H), 6.08 (s, 1H), 5.68 (s, 1H), 3.76 (s, 3H), 2.45 (s, 3H), 2.23 (s, 3H), 1.31 (s, 6H), 1.07 (s, 9H).

^{13}C NMR (101 MHz, $CDCl_3$) δ 194.28, 159.00, 156.36, 151.70, 148.65, 141.46, 138.81, 128.44, 121.61, 118.89, 112.73, 110.05, 109.74, 55.00, 40.29, 36.37, 30.84, 29.62, 28.86, 14.27

HRMS (ESI): $C_{23}H_{31}O_3^+$, calcd. 355.2268, found 355.2274.

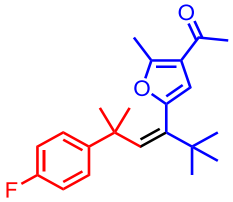

3ea

(Z)-1-(5-(5-(4-fluorophenyl)-2,2,5-trimethylhex-3-en-3-yl)-2-methylfuran-3-yl)ethan-1-one (3ea) Yellow solid, 30.4 mg.

^{1}H NMR (400 MHz, $CDCl_3$) δ 7.08 (dd, J = 8.8, 5.4 Hz, 2H), 6.85 (t, J = 8.8 Hz, 2H), 6.08 (s, 1H), 5.69 (s, 1H), 2.44 (s, 3H), 2.25 (s, 3H), 1.31 (s, 6H), 1.05 (s, 9H).

^{13}C NMR (126 MHz, $CDCl_3$) δ 194.25, 160.74 (J_{C-F} = 256.4 Hz), 156.42, 148.60, 145.68 (J_{C-F} = 2.8 Hz), 141.50, 139.21, 127.65 (J_{C-F} = 8.1 Hz), 121.66, 114.12(J_{C-F} = 21.2 Hz), 109.82, 39.78, 36.40, 31.09, 29.55, 28.88, 14.26

HRMS (ESI): $C_{22}H_{28}FO_2^+$, calcd. 343.2068, found 343.2074.

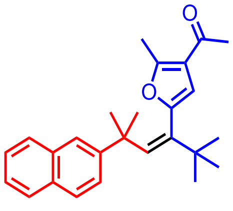

3fa

(Z)-1-(2-methyl-5-(2,2,5-trimethyl-5-(naphthalen-2-yl)hex-3-en-3-yl)furan-3-yl)ethan-1-one (3fa) Yellow solid, 19.8 mg.

^{1}H NMR (400 MHz, $CDCl_3$) δ 7.79 – 7.72 (m, 1H), 7.67 (dd, J = 8.5, 5.3 Hz, 2H), 7.45 – 7.32 (m, 4H), 6.16 (s, 1H), 5.50 (s, 1H), 2.17 (s, 3H), 1.79 (s, 3H), 1.47 (s, 6H), 1.06 (s, 9H).

^{13}C NMR (126 MHz, $CDCl_3$) δ 193.95, 156.18, 148.31, 146.90, 141.55, 139.68, 133.13, 131.45, 127.50, 127.18, 126.90, 126.51, 125.78, 125.19, 123.33, 121.50, 109.81, 40.22, 36.35, 30.92, 29.53, 28.32, 13.98

HRMS (ESI): $C_{26}H_{31}O_2^+$, calcd. 375.2319, found 375.2311.

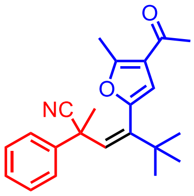

3ga

(Z)-4-(4-acetyl-5-methylfuran-2-yl)-2,5,5-trimethyl-2-phenylhex-3-enenitrile (3ga) White oil, 17.42 mg.

^{1}H NMR (400 MHz, $CDCl_3$) δ 7.36 – 7.23 (m, 5H), 5.98 (d, J = 6.7 Hz, 2H), 2.45 (s, 3H), 2.25 (s, 3H), 1.81 (s, 3H), 1.14 (s, 9H)

^{13}C NMR (126 MHz, $CDCl_3$) δ 194.10, 157.83, 146.43, 145.25, 142.63, 131.53, 128.65, 127.22, 125.56, 122.06, 121.53, 112.08, 41.62, 36.77, 31.97, 29.35, 28.99, 14.23

HRMS (ESI): $C_{22}H_{26}NO_2^+$, calcd. 336.1958, found 336.195.

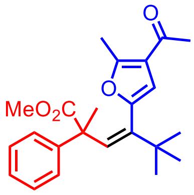

3ha

Methyl-(Z)-4-(4-acetyl-5-methylfuran-2-yl)-2,5,5-trimethyl-2-phenylhex-3-enoate (3ha) White solid, 19.2 mg.

^{1}H NMR (400 MHz, $CDCl_3$) δ 7.23 (d, J = 2.7 Hz, 5H), 6.56 (s, 1H), 5.84 (s, 1H), 3.53 (s, 3H), 2.48 (s, 3H), 2.27 (s, 3H), 1.59 (s, 3H), 1.11 (s, 9H).

^{13}C NMR (101 MHz, $CDCl_3$) δ 194.17, 175.47, 156.82, 148.19, 143.99, 140.56,

135.68, 127.97, 126.87, 126.41, 121.67, 110.27, 52.30, 51.98, 36.84, 29.58, 28.99, 24.62, 14.31.

HRMS (ESI): $C_{23}H_{29}O_4^+$, calcd. 369.206, found 369.2062.

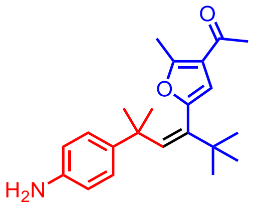

3ja

(Z)-1-(5-(5-(4-aminophenyl)-2,2,5-trimethylhex-3-en-3-yl)-2-methylfuran-3-yl)ethan-1-one (3ja) Yellow solid, 21.0 mg.

^{1}H NMR (400 MHz, $CDCl_3$) δ 6.93 (d, *J* = 8.5 Hz, 2H), 6.53 (d, *J* = 8.5 Hz, 2H), 6.05 (s, 1H), 5.73 (s, 1H), 2.49 (s, 3H), 2.29 (s, 3H), 1.25 (s, 6H), 1.05 (s, 9H).

^{13}C NMR (126 MHz, $CDCl_3$) δ 194.71, 156.50, 148.90, 143.71, 141.97, 140.40, 138.17, 126.96, 121.65, 114.49, 109.82, 39.54, 36.36, 30.78, 29.62, 29.00, 14.36.

HRMS (ESI): $C_{22}H_{30}NO_2^+$, calcd. 339.2198, found 339.2194.

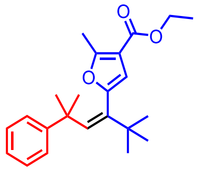

3ab

Ethyl-(Z)-2-methyl-5-(2,2,5-trimethyl-5-phenylhex-3-en-3-yl)furan-3-carboxylate (3ab) Yellow solid, 27.3 mg.

^{1}H NMR (400 MHz, $CDCl_3$) δ 7.19 (d, *J* = 6.1 Hz, 4H), 7.11 (ddd, *J* = 6.1, 5.1, 2.6 Hz, 1H), 6.06 (s, 1H), 5.92 (s, 1H), 4.26 (q, *J* = 7.1 Hz, 2H), 2.42 (s, 3H), 1.35 (t, *J* = 7.1 Hz, 3H), 1.31 (s, 6H), 1.07 (s, 9H).

^{13}C NMR (126 MHz, $CDCl_3$) δ 164.37, 157.12, 149.93, 148.80, 141.22, 138.45, 127.64, 126.13, 125.09, 113.61, 110.11, 59.83, 40.46, 36.46, 30.52, 29.62, 14.40, 13.70.

HRMS (ESI): $C_{23}H_{31}O_3^+$, calcd. 355.2268, found 355.2267.

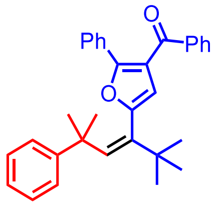

3ac

(Z)-phenyl(2-phenyl-5-(2,2,5-trimethyl-5-phenylhex-3-en-3-yl)furan-3-yl)methanone (3ac) Yellow solid, 38.1 mg.

^{1}H NMR (400 MHz, $CDCl_3$) δ 7.75 – 7.69 (m, 2H), 7.62 – 7.57 (m, 2H), 7.55 – 7.48 (m, 1H), 7.38 (t, *J* = 7.7 Hz, 2H), 7.31 – 7.26 (m, 5H), 7.21 (dd, *J* = 8.5, 6.9 Hz, 2H), 7.11 – 7.04 (m, 1H), 6.18 (s, 1H), 5.99 (s, 1H), 1.41 (s, 6H), 1.16 (s, 9H).

^{13}C NMR (101 MHz, $CDCl_3$) δ 191.67, 154.34, 150.10, 149.85, 141.48, 138.41, 138.27, 132.51, 130.00, 129.64, 128.56, 128.16, 128.12, 127.83, 127.41, 126.12, 125.37, 121.16, 113.34, 40.57, 36.71, 31.08, 29.75.

HRMS (ESI): $C_{32}H_{33}O_2^+$, calcd. 449.2475, found 449.2474.

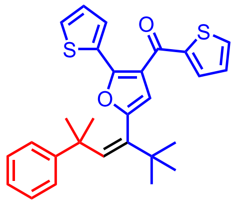

3ad

(Z)-thiophen-2-yl(2-(thiophen-2-yl)-5-(2,2,5-trimethyl-5-phenylhex-3-en-3-yl)furan-3-yl)methanone (3ad) Yellow solid, 41.8 mg.

^{1}H NMR (400 MHz, $CDCl_3$) δ 7.83 (dd, *J* = 3.8, 1.2 Hz, 1H), 7.65 (dd, *J* = 4.9, 1.1 Hz, 1H), 7.39 (dd, *J* = 5.0, 1.2 Hz, 1H), 7.35 (dd, *J* = 3.8, 1.2 Hz, 1H), 7.30 – 7.25 (m, 2H), 7.21 (dd, *J* = 8.6, 6.9 Hz, 2H), 7.12 – 7.04 (m, 3H), 6.22 (s, 1H), 6.10 (s, 1H), 1.42 (s, 6H), 1.17 (s, 9H).

^{13}C NMR (126 MHz, $CDCl_3$) δ 181.49, 150.89, 149.93, 149.65, 144.94, 141.98, 138.37, 133.43, 133.34, 132.05, 127.95, 127.89, 127.78, 127.41, 127.37, 126.16, 125.45, 119.43, 112.52, 40.51, 36.65, 31.26, 29.73.

HRMS (ESI): $C_{28}H_{29}O_2S_2^+$, calcd. 461.1603, found 461.1603.

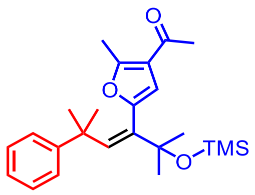

3ae

(E)-1-(5-(2,5-dimethyl-5-phenyl-2-((trimethylsilyl)oxy)hex-3-en-3-yl)-2-methylfuran-3-yl)ethan-1-one (3ae) Yellow oil, 21.1 mg.

^{1}H NMR (500 MHz, $CDCl_3$) δ 7.17 – 7.12 (m, 4H), 7.07 (ddd, *J* = 6.1, 5.1, 2.6 Hz, 1H), 6.36 (s, 1H), 5.71 (s, 1H), 2.40 (s, 3H), 2.19 (s, 3H), 1.33 (s, 6H), 1.31 (s, 6H), 0.15 (s, 9H).

^{13}C NMR (126 MHz, $CDCl_3$) δ 194.33, 156.65, 149.75, 147.94, 142.06, 137.15, 127.59, 126.21, 125.12, 121.70, 110.49, 75.96, 40.05, 30.77, 30.40, 28.92, 14.29, 2.54.

HRMS (ESI): $C_{24}H_{35}O_3Si^+$, calcd. 399.235, found 399.2349.

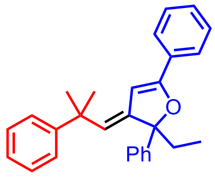

5aa

(E)-2-ethyl-3-(2-methyl-2-phenylpropylidene)-2,5-diphenyl-2,3-dihydrofuran (5aa) Yellow oil, 18.24 mg.

^{1}H NMR (400 MHz, $CDCl_3$) δ 7.58 – 7.53 (m, 2H), 7.49 (dd, *J* = 7.7, 1.7 Hz, 2H), 7.39 – 7.29 (m, 10H), 7.23 – 7.17 (m, 1H), 5.50 (s, 1H), 5.17 (d, *J* = 1.0 Hz, 1H), 2.30 (dq, *J* = 14.3, 7.2 Hz, 1H), 2.09 (dq, *J* = 14.3, 7.2 Hz, 1H), 1.53 (s, 3H), 1.48 (s, 3H), 0.97 (t, *J* = 7.2 Hz, 3H).

^{13}C NMR (126 MHz, $CDCl_3$) δ 160.09, 150.36, 145.73, 144.82, 130.59, 128.92, 128.32, 128.20, 128.11, 127.17, 126.53, 125.58, 125.48, 125.41, 125.02, 98.83, 92.84, 40.84, 33.58, 31.36, 31.15, 7.92.

HRMS (ESI): $C_{28}H_{29}O^+$, calcd. 381.2213, found 381.2226.

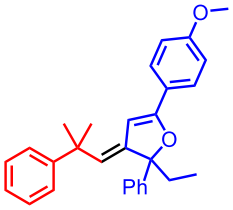

5ab

(E)-2-ethyl-5-(4-methoxyphenyl)-3-(2-methyl-2-phenylpropylidene)-2-phenyl-2,3-dihydrofuran (5ab) Yellow solid, 27.9 mg.

^{1}H NMR (400 MHz, $CDCl_3$) δ 7.54 – 7.47 (m, 3H), 7.38 (ddt, *J* = 8.0, 6.0, 1.5 Hz, 3H), 7.34 – 7.29 (m, 3H), 7.24 – 7.18 (m, 1H), 6.88 (d, *J* = 8.8 Hz, 2H), 5.40 (d, *J* = 0.9 Hz, 1H), 5.13 (d, *J* = 1.0 Hz, 1H), 3.84 (s, 3H), 2.30 (dq, *J* = 14.4, 7.2 Hz, 1H), 2.10 (dq, *J* = 14.4, 7.2 Hz, 1H), 1.54 (s, 3H), 1.49 (s, 3H), 0.98 (t, *J* = 7.2 Hz, 3H).

^{13}C NMR (101 MHz, $CDCl_3$) δ 160.28, 160.07, 150.49, 145.94, 144.94, 128.16, 128.06, 127.10, 126.99, 126.53, 125.50, 125.02, 124.22, 123.40, 113.77, 97.18, 92.78, 55.34, 40.76, 33.52, 31.38, 31.15, 7.92.

HRMS (ESI): $C_{29}H_{31}O_2^+$, calcd. 411.2319, found 411.233.

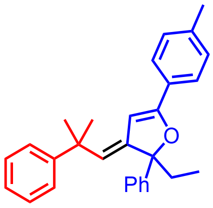

5ac

(E)-2-ethyl-3-(2-methyl-2-phenylpropylidene)-2-phenyl-5-(p-tolyl)-2,3-dihydrofuran Yellow solid, 27.6 mg.

^{1}H NMR (400 MHz, $CDCl_3$) δ 7.50 (ddd, *J* = 11.2, 7.7, 1.7 Hz, 4H), 7.42 – 7.36 (m, 4H), 7.32 (ddt, *J* = 9.0, 7.3, 2.4 Hz, 3H), 7.25 – 7.15 (m, 3H), 5.49 (d, *J* = 0.9 Hz, 1H), 5.17 (d, *J* = 1.0 Hz, 1H), 2.39 (s, 3H), 2.31 (dq, *J* = 14.3, 7.2 Hz, 1H), 2.10 (dq, *J* = 14.3, 7.2 Hz, 1H), 1.55 (s, 3H), 1.50 (s, 3H), 0.99 (t, *J* = 7.2 Hz, 3H).

^{13}C NMR (101 MHz, $CDCl_3$) δ 160.32, 150.44, 145.89, 144.93, 139.04, 129.03, 128.17, 128.09, 127.85, 127.12, 126.52, 125.54, 125.45, 125.01, 124.76, 98.10, 92.76, 40.81, 33.56, 31.38, 31.17, 21.44, 7.93.

HRMS (ESI): $C_{29}H_{31}O^+$, calcd. 395.2369, found 395.2381.

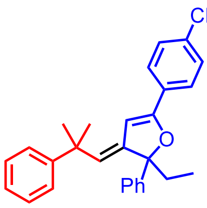

5ad

(E)-5-(4-chlorophenyl)-2-ethyl-3-(2-methyl-2-phenylpropylidene)-2-phenyl-2,3-dihydrofuran (5ad) Yellow oil, 10.4 mg.

^{1}H NMR (400 MHz, $CDCl_3$) δ 7.48 – 7.43 (m, 4H), 7.40 – 7.29 (m, 9H), 7.23 – 7.18 (m, 1H), 5.45 (d, *J* = 1.0 Hz, 1H), 5.19 (d, *J* = 1.1 Hz, 1H), 2.28 (dp, *J* = 13.1, 6.9 Hz, 1H), 2.03 (dp, *J* = 13.1, 6.9 Hz, 1H), 1.53 (s, 3H), 1.48 (s, 3H), 0.95 (t, *J* = 7.2 Hz, 3H).

^{13}C NMR (126 MHz, $CDCl_3$) δ 158.96, 150.21, 145.41, 144.59, 134.63, 129.06, 128.54, 128.24, 128.14, 127.27, 126.71, 126.52, 126.11, 125.64, 124.98, 99.23, 93.08, 40.88, 33.57, 31.31, 31.12, 7.90.

HRMS (ESI): $C_{28}H_{28}ClO^+$, calcd. 415.1823, found 415.1828.

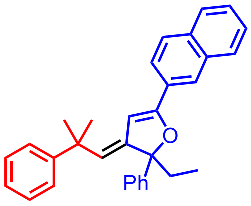

5ae

(E)-2-ethyl-3-(2-methyl-2-phenylpropylidene)-5-(naphthalen-2-yl)-2-phenyl-2,3-dihydrofuran (5ae) Yellow solid, 24.5 mg.

^{1}H NMR (400 MHz, $CDCl_3$) δ 8.16 (s, 1H), 7.91 – 7.84 (m, 1H), 7.84 – 7.80 (m, 1H), 7.77 (d, *J* = 8.6 Hz, 1H), 7.57 – 7.47 (m, 5H), 7.40 (dt, *J* = 7.7, 3.7 Hz, 4H), 7.34 (t, *J* = 7.5 Hz, 3H), 7.24 (t, *J* = 7.2 Hz, 1H), 5.66 (s, 1H), 5.23 (s, 1H), 2.37 (dq, *J* = 14.2, 6.8 Hz, 1H), 2.16 (dq, *J* = 14.4, 7.3 Hz, 1H), 1.58 (s, 3H), 1.52 (s, 3H), 1.03 (t, *J* = 7.2 Hz, 3H).

^{13}C NMR (101 MHz, $CDCl_3$) δ 160.13, 150.35, 145.75, 144.85, 133.52, 133.19, 128.53, 128.25, 128.15, 127.86, 127.77, 127.66, 127.23, 126.55, 126.51, 126.43, 125.77, 125.63, 125.05, 124.46, 123.39, 99.69, 92.95, 40.93, 33.55, 31.38, 31.16, 7.96.

HRMS (ESI): $C_{32}H_{31}O^+$, calcd. 431.2369, found 431.2364.

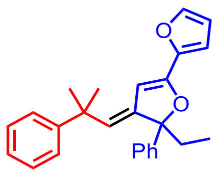

5af

(E)-5-ethyl-4-(2-methyl-2-phenylpropylidene)-5-phenyl-4,5-dihydro-2,2'-bifuran (5af) Yellow oil, 7.5 mg.

^{1}H NMR (400 MHz, $CDCl_3$) δ 7.49 – 7.43 (m, 2H), 7.43 – 7.26 (m, 8H), 7.22 – 7.16 (m, 1H), 6.63 (d, *J* = 3.4 Hz, 1H), 6.43 (dd, *J* = 3.4, 1.8 Hz, 1H), 5.51 (d, *J* = 1.0 Hz, 1H), 5.15 (d, *J* = 1.1 Hz, 1H), 2.27 (dq, *J* = 14.4, 7.3 Hz, 1H), 2.07 (dq, *J* = 14.4, 7.3 Hz, 1H), 1.52 (s, 3H), 1.47 (s, 3H), 0.96 (t, *J* = 7.2 Hz, 3H).

^{13}C NMR (126 MHz, $CDCl_3$) δ 152.15, 150.38, 146.50, 144.89, 144.56, 143.35, 128.21, 128.11, 127.29, 126.39, 125.80, 125.57, 125.07, 111.46, 109.04, 98.59, 93.34, 40.81, 33.31, 31.36, 30.98, 7.83.

HRMS (ESI): $C_{26}H_{27}O_2^+$, calcd. 371.2006, found 371.1996.

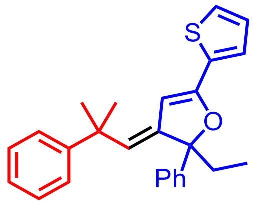

5ag

(E)-2-ethyl-3-(2-methyl-2-phenylpropylidene)-2-phenyl-5-(thiophen-2-yl)-2,3-dihydrofuran (5ag) Yellow oil, 12.0 mg.

^{1}H NMR (400 MHz, $CDCl_3$) δ 7.51 – 7.45 (m, 2H), 7.40 – 7.27 (m, 8H),7.25 (dd, *J* = 3.7, 1.2 Hz, 1H), 7.23 – 7.18 (m, 1H), 7.02 (dd, *J* = 5.0, 3.7 Hz, 1H), 5.36 (d, *J* = 1.0 Hz, 1H), 5.15 (d, *J* = 1.1 Hz, 1H), 2.27 (dq, *J* = 14.4, 7.3 Hz, 1H), 2.09 (dq, *J* = 14.4, 7.3 Hz, 1H), 1.52 (s, 3H), 1.47 (s, 3H), 0.97 (t, *J* = 7.2 Hz, 3H).

^{13}C NMR (126 MHz, $CDCl_3$) δ 155.34, 150.23, 145.26, 144.59, 133.81, 128.17, 128.08, 127.54, 127.21, 126.46, 126.24, 125.57, 125.40, 125.35, 125.00, 98.50, 93.40, 40.82, 33.44, 31.26, 31.04, 7.86.

HRMS (ESI): $C_{26}H_{27}OS^+$, calcd. 386.1704, found 386.1710.

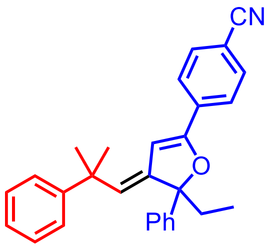

5ah

(E)-4-(5-ethyl-4-(2-methyl-2-phenylpropylidene)-5-phenyl-4,5-dihydrofuran-2-yl)benzonitrile (5ah) Yellow solid, 7.3 mg.

^{1}H NMR (400 MHz, $CDCl_3$) δ 7.64 – 7.54 (m, 4H), 7.47 – 7.42 (m, 2H), 7.40 – 7.29 (m, 7H), 7.26 – 7.20 (m, 1H), 5.53 (d, J = 1.1 Hz, 1H), 5.29 (d, J = 1.2 Hz, 1H), 2.37 – 2.22 (m, 1H), 2.15 – 2.00 (m, 1H), 1.53 (s, 3H), 1.48 (s, 3H), 0.95 (t, J = 7.2 Hz, 3H).

^{13}C NMR (126 MHz, $CDCl_3$) δ 157.84, 149.86, 144.87, 144.19, 134.61, 132.08, 128.42, 128.30, 128.21, 127.44, 126.52, 125.78, 125.73, 124.93, 118.82, 111.69, 101.93, 93.38, 41.03, 33.60, 31.24, 31.08, 7.87.

HRMS (ESI): $C_{29}H_{28}NO^{+}$, calcd. 405.2093, found 405.2090.

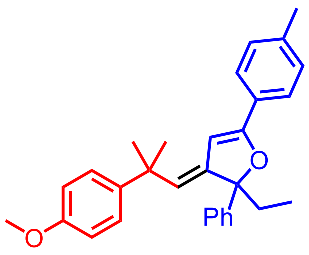

5cc

(E)-2-ethyl-3-(2-(4-methoxyphenyl)-2-methylpropylidene)-2-phenyl-5-(p-tolyl)-2,3-dihydrofuran (5cc) Yellow solid, 21.6mg.

^{1}H NMR (400 MHz, $CDCl_3$) δ 7.48 (dd, J = 8.4, 1.7 Hz, 4H), 7.43 – 7.30 (m, 3H), 7.28 – 7.24 (m, 2H), 7.16 (d, J = 8.0 Hz, 2H), 6.87 – 6.81 (m, 2H), 5.53 (d, J = 1.1 Hz, 1H), 5.13 (d, J = 1.0 Hz, 1H), 3.82 (s, 3H), 2.38 (s, 3H), 2.28 (dq, J = 14.3, 7.2 Hz, 1H), 2.09 (dq, J = 14.4, 7.2 Hz, 1H), 1.50 (s, 3H), 1.45 (s, 3H), 0.97 (t, J = 7.2 Hz, 3H).

^{13}C NMR (126 MHz, $CDCl_3$) δ 160.20, 157.34, 145.67, 144.94, 142.62, 139.00, 129.00, 128.14, 127.83, 127.43, 127.07, 125.43, 124.96, 124.93, 113.34, 98.13, 92.70, 55.21, 40.13, 33.51, 31.57, 31.26, 21.40, 7.89.

HRMS (ESI): $C_{30}H_{33}O_2^{+}$, calcd. 425.2475, found 425.2473.

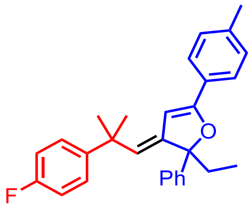

5ec

(E)-2-ethyl-3-(2-(4-fluorophenyl)-2-methylpropylidene)-2-phenyl-5-(p-tolyl)-2,3-dihydrofuran (5ec) Yellow solid, 27.2 mg.

^{1}H NMR (400 MHz, $CDCl_3$) δ 7.51 – 7.45 (m, 4H), 7.40 – 7.34 (m, 2H), 7.33 – 7.26 (m, 3H), 7.17 (d, *J* = 8.0 Hz, 2H), 7.01 – 6.94 (m, 2H), 5.46 (d, *J* = 1.0 Hz, 1H), 5.12 (d, *J* = 1.0 Hz, 1H), 2.38 (s, 3H), 2.30 (dq, *J* = 14.4, 7.2 Hz, 1H), 2.10 (dq, *J* = 14.4, 7.2 Hz, 1H), 1.52 (s, 3H), 1.45 (s, 3H), 0.97 (t, *J* = 7.2 Hz, 3H).

^{13}C NMR (126 MHz, $CDCl_3$) δ 160.92 (J_{C-F} = 244.4 Hz), 160.65, 159.95, 146.23, 146.19, 144.83, 139.24, 129.09, 127.97 (J_{C-F} = 8.1 Hz), 127.94, 127.71, 127.18, 125.49, 124.93, 124.30, 114.69 (J_{C-F} = 20.5 Hz), 97.84, 92.77, 40.35, 33.48, 31.66, 31.28, 21.45, 7.91.

HRMS (ESI): $C_{29}H_{30}FO^+$, calcd. 413.2275, found 413.2262.

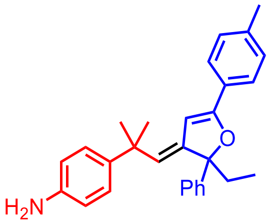

5jc

(E)-4-(1-(2-ethyl-2-phenyl-5-(p-tolyl)furan-3(2H)-ylidene)-2-methylpropan-2-yl)aniline (5jc) Yellow oil, 15.6 mg.

^{1}H NMR (400 MHz, $CDCl_3$) δ 7.47 (td, *J* = 5.9, 2.7 Hz, 4H), 7.38 – 7.31 (m, 2H), 7.30 – 7.24 (m, 1H), 7.19 – 7.12 (m, 4H), 6.69 (d, *J* = 8.5 Hz, 2H), 5.54 (d, *J* = 1.0 Hz, 1H), 5.10 (d, *J* = 1.1 Hz, 1H), 2.36 (s, 3H), 2.26 (dq, *J* = 14.4, 7.2 Hz, 1H), 2.05 (dq, *J* = 14.4, 7.2 Hz, 1H), 1.47 (s, 3H), 1.42 (s, 3H), 0.95 (t, *J* = 7.2 Hz, 3H).

^{13}C NMR (126 MHz, $CDCl_3$) δ 160.19, 145.59, 144.97, 139.02, 129.06, 128.15, 127.86, 127.38, 127.08, 125.47, 125.06, 124.98, 116.15, 98.18, 92.71, 40.15, 33.56, 31.52, 31.20, 21.43, 7.91.

HRMS (ESI): $C_{29}H_{32}NO^+$, calcd. 410.2478, found 410.2484.

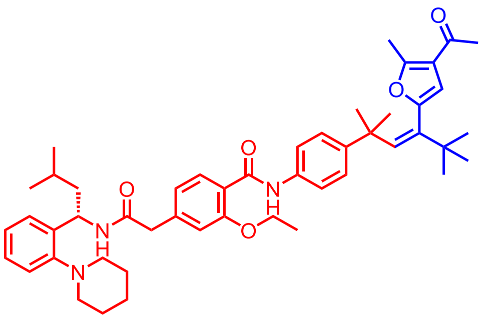

3ka

(S,Z)-N-(4-(4-(4-acetyl-5-methylfuran-2-yl)-2,5,5-trimethylhex-3-en-2-yl)phenyl)-2-ethoxy-4-(2-((3-methyl-1-(2-(piperidin-1-yl)phenyl)butyl)amino)-2-oxoethyl)benzamide (3ka) Yellow solid, 71.2 mg.

^{1}H NMR (400 MHz, $CDCl_3$) δ 10.00 (s, 1H), 8.21 (d, *J* = 7.9 Hz, 1H), 7.50 – 7.45 (m, 2H), 7.25 – 7.17 (m, 2H), 7.13 (td, *J* = 6.5, 6.0, 3.3 Hz, 3H), 7.06 (ddd, *J* = 7.9, 6.3, 2.2 Hz, 1H), 6.96 (ddd, *J* = 11.6, 6.4, 3.0 Hz, 3H), 6.08 (s, 1H), 5.73 (s, 1H), 5.40 (td, *J* = 8.7, 6.4 Hz, 1H), 4.23 – 4.07 (m, 2H), 3.57 (s, 2H), 2.92 (d, *J* = 28.4 Hz, 2H), 2.61 (q, *J* = 11.0, 9.9 Hz, 2H), 2.45 (s, 3H), 2.21 (s, 3H), 1.99 (s, 2H), 1.78 – 1.68 (m, 2H), 1.67 – 1.58 (m, 4H), 1.57 – 1.41 (m, 4H), 1.31 (s, 6H), 1.05 (s, 9H), 0.93 (dd, *J* = 6.5, 2.0 Hz, 6H).

^{13}C NMR (126 MHz, $CDCl_3$) δ 192.63, 167.00, 160.93, 154.95, 154.55, 150.65, 146.84, 143.98, 139.73, 139.24, 136.99, 136.90, 133.95, 130.86, 126.04, 125.84, 124.92, 123.17, 120.89, 120.33, 119.85, 118.48, 117.35, 111.17, 108.08, 63.18, 47.90, 44.81, 42.13, 38.04, 34.52, 28.97, 27.71, 27.07, 24.91, 23.49, 22.29, 20.96, 20.67, 12.99, 12.47.

HRMS (ESI): $C_{49}H_{64}N_3O_5^+$, calcd. 774.484, found 774.4839.

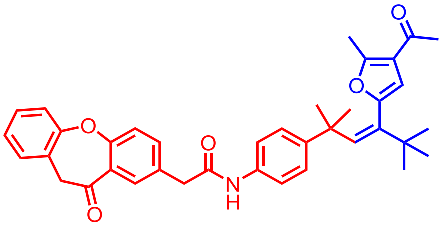

3la

(Z)-N-(4-(4-(4-acetyl-5-methylfuran-2-yl)-2,5,5-trimethylhex-3-en-2-yl)phenyl)-2-(11-oxo-10,11-dihydrodibenzo[b,f]oxepin-2-yl)acetamide (3la) Yellow solid, 49 mg.

^{1}H NMR (400 MHz, $CDCl_3$) δ 8.17 (d, *J* = 2.4 Hz, 1H), 7.89 (dd, *J* = 7.7, 1.3 Hz, 1H), 7.74 (s, 1H), 7.60 – 7.45 (m, 3H),7.38 (dd, *J* = 7.5, 1.2 Hz, 1H), 7.31 (d, *J* = 2.0 Hz, 1H), 7.10 – 7.00 (m, 3H), 6.03 (s, 1H), 5.68 (s, 1H), 5.20 (s, 2H), 3.71 (s, 2H), 2.42 (s, 3H), 2.20 (s, 3H), 2.10 (s, 1H), 1.27 (s, 6H), 1.03 (s, 9H).

^{13}C NMR (126 MHz, $CDCl_3$) δ 194.70, 190.93, 168.86, 160.68, 156.48, 148.69, 146.06, 141.48, 140.34, 138.73, 136.40, 135.56, 135.15, 132.92, 132.40, 129.50,

129.33, 128.63, 127.90, 126.64, 125.27, 121.76, 121.57, 119.16, 109.91, 73.65, 43.52, 39.91, 36.37, 30.78, 29.56, 28.98, 14.37.

HRMS (ESI): $C_{38}H_{40}NO_5^+$, calcd. 590.2901, found 590.2905.

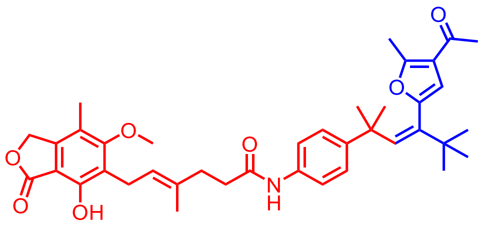

3ma

(E)-N-(4-((Z)-4-(4-acetyl-5-methylfuran-2-yl)-2,5,5-trimethylhex-3-en-2-yl)phenyl)-6-(4-hydroxy-6-methoxy-7-methyl-3-oxo-1,3-dihydroisobenzofuran-5-yl)-4-methylhex-4-enamide (3ma) Yellow solid, 33.3 mg.

^{1}H NMR (400 MHz, $CDCl_3$) δ 7.72 (s, 1H), 7.38 (s, 1H), 7.24 (d, J = 8.5 Hz, 2H), 7.07 – 7.00 (m, 2H), 6.04 (s, 1H), 5.70 (s, 1H), 5.36 – 5.27 (m, 1H), 5.20 (s, 2H), 3.76 (s, 3H), 3.42 (d, J = 6.9 Hz, 2H), 2.47 – 2.38 (m, 7H), 2.22 (s, 3H), 2.14 (s, 3H), 1.85 (s, 3H), 1.28 (s, 6H), 1.04 (s, 9H).

^{13}C NMR (101 MHz, $CDCl_3$) δ 194.57, 172.89, 170.80, 163.64, 156.41, 153.57, 148.66, 145.70, 144.07, 141.44, 138.66, 135.24, 134.55, 126.58, 123.08, 122.04, 121.71, 119.02, 116.79, 109.91, 106.38, 70.05, 61.02, 39.91, 36.36, 36.07, 35.14, 30.78, 29.56, 28.95, 22.65, 16.24, 14.33, 11.57.

HRMS (ESI): $C_{39}H_{48}NO_7^+$, calcd. 642.3425, found 642.3409.

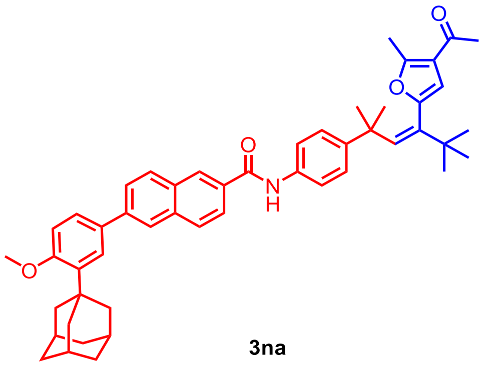

3na

N-(4-((Z)-4-(4-acetyl-5-methylfuran-2-yl)-2,5,5-trimethylhex-3-en-2-yl)phenyl)-6-(3-((3r,5r,7r)-adamantan-1-yl)-4-methoxyphenyl)-2-naphthamide (3na) Yellow solid, 50.6 mg.

^{1}H NMR (400 MHz, $CDCl_3$) δ 8.33 (d, J = 39.0 Hz, 2H), 8.04 – 8.01 (m, 1H), 7.99 – 7.92 (m, 3H), 7.81 (dd, J = 8.6, 1.8 Hz, 1H), 7.63 (d, J = 2.3 Hz, 1H), 7.58 – 7.52 (m, 3H), 7.16 (d, J = 8.6 Hz, 2H), 7.01 (d, J = 8.5 Hz, 1H), 6.10 (s, 1H), 5.76 (s, 1H), 3.92 (s, 3H), 2.49 (s, 3H), 2.26 (s, 3H), 2.22 (d, J = 2.9 Hz, 6H), 2.14 (t, J = 3.4 Hz, 3H), 1.84 (d, J = 3.0 Hz, 6H), 1.35 (s, 6H), 1.08 (s, 9H).

^{13}C NMR (101 MHz, $CDCl_3$) δ 194.68, 165.77, 158.89, 156.47, 148.73, 146.10, 141.54, 140.95, 139.00, 138.80, 135.47, 135.25, 132.53, 131.75, 131.36, 129.34, 128.73, 127.32, 126.83, 126.68, 125.94, 125.71, 124.71, 123.93, 121.85, 119.48, 112.13, 109.98, 55.18, 40.63, 40.00, 37.23, 37.15, 36.40, 30.88, 29.59, 29.13, 29.02, 14.43.

HRMS (ESI): $C_{50}H_{56}NO_4^+$, calcd. 734.4204, found 734.4192.

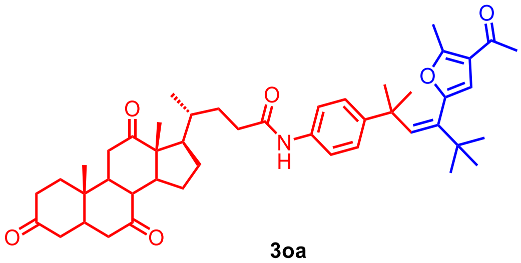

3oa

(4R)-N-(4-((Z)-4-(4-acetyl-5-methylfuran-2-yl)-2,5,5-trimethylhex-3-en-2-yl)phenyl)-4-((10S,13R,17R)-10,13-dimethyl-3,7,12-trioxohexadecahydro-1H-cyclopenta[a]phenanthren-17-yl)pentanamide (3oa) Yellow solid, 65.8 mg.

^{1}H NMR (400 MHz, Chloroform-*d*) δ 7.68 (s, 1H), 7.31 (d, *J* = 8.6 Hz, 2H), 7.07 – 7.00 (m, 2H), 6.03 (s, 1H), 5.68 (s, 1H), 2.97 – 2.80 (m, 3H), 2.48 – 2.43 (m, 1H), 2.42 (s, 3H), 2.38 – 2.22 (m, 6H), 2.21 (s, 3H), 2.17 – 2.09 (m, 3H), 2.06 – 2.02 (m, 3H), 1.99 – 1.89 (m, 2H), 1.84 (td, *J* = 11.4, 7.1 Hz, 1H), 1.61 (td, *J* = 14.5, 4.4 Hz, 1H), 1.48 (dtd, *J* = 14.2, 9.1, 5.3 Hz, 1H), 1.40 (s, 3H), 1.38 – 1.30 (m, 3H), 1.27 (s, 6H), 1.07 (s, 3H), 1.02 (s, 9H), 0.86 (d, *J* = 6.6 Hz, 3H).

^{13}C NMR (126 MHz, $CDCl_3$) δ 210.34, 207.43, 207.08, 192.72, 169.68, 154.52, 146.79, 143.68, 139.63, 136.78, 133.58, 124.73, 119.78, 117.02, 107.98, 55.01, 49.88, 47.07, 44.91, 43.61, 43.51, 43.09, 40.88, 37.96, 36.76, 34.56, 34.44, 34.10, 33.48, 33.34, 32.39, 29.04, 28.95, 28.87, 27.64, 27.06, 25.73, 23.25, 19.96, 16.85, 12.43, 9.98.

HRMS (ESI): $C_{46}H_{60}NO_6^-$, calcd. 722.4426, found 722.4422.

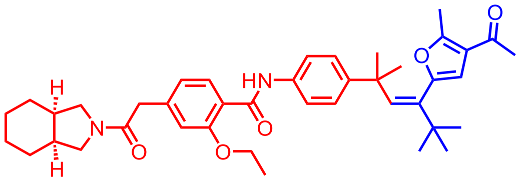

3pa

N-(4-((Z)-4-(4-acetyl-5-methylfuran-2-yl)-2,5,5-trimethylhex-3-en-2-yl)phenyl)-2-ethoxy-4-(2-((3aR,7aS)-octahydro-2H-isoindol-2-yl)-2-oxoethyl)benzamide (3pa) Yellow solid, 49.6 mg.

^{1}H NMR (400 MHz, $CDCl_3$) δ 10.02 (s, 1H), 8.22 (d, *J* = 8.0 Hz, 1H), 7.47 (d, *J* = 8.6 Hz, 2H), 7.14 – 7.08 (m, 2H), 7.04 (d, *J* = 1.5 Hz, 1H), 6.98 (dd, *J* = 8.0, 1.5 Hz, 1H), 6.07 (s, 1H), 5.71 (s, 1H), 4.29 (q, *J* = 7.0 Hz, 2H), 3.68 (s, 2H), 3.51 – 3.37 (m,

3H), 3.32 (dd, J = 9.9, 5.8 Hz, 1H), 2.44 (s, 3H), 2.26 (td, J = 14.0, 12.8, 6.7 Hz, 2H), 2.20 (s, 3H), 2.20 – 2.14 (m, 1H), 1.62 (t, J = 6.9 Hz, 3H), 1.58 – 1.34 (m, 8H), 1.31 (s, 6H), 1.04 (s, 9H).

^{13}C NMR (101 MHz, $CDCl_3$) δ 194.49, 169.39, 162.88, 156.81, 156.39, 148.68, 145.75, 141.60, 140.63, 138.84, 135.88, 132.52, 126.76, 122.26, 121.71, 120.27, 119.20, 113.04, 109.95, 65.13, 51.04, 49.83, 41.98, 39.89, 37.65, 36.37, 35.89, 30.83, 29.57, 28.93, 25.70, 22.72, 22.52, 14.89, 14.33.

HRMS (ESI): $C_{41}H_{51}N_2O_5^-$, calcd. 651.3803, found 651.3798.

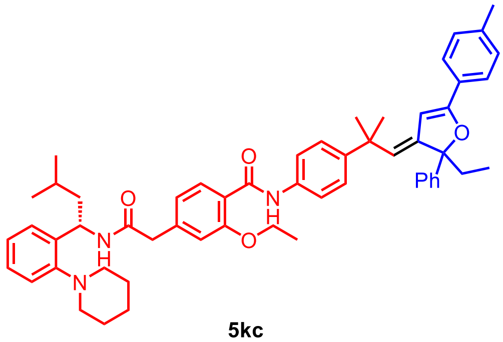

5kc

2-ethoxy-N-(4-((E)-1-(2-ethyl-2-phenyl-5-(p-tolyl)furan-3(2H)-ylidene)-2-methylpropan-2-yl)phenyl)-4-(2-(((S)-3-methyl-1-(2-(piperidin-1-yl)phenyl)butyl)amino)-2-oxoethyl)benzamide (5kc) Yellow solid, 49.7 mg.

^{1}H NMR (400 MHz, $CDCl_3$) δ 10.06 (s, 1H), 8.26 (d, J = 8.0 Hz, 1H), 7.61 – 7.56 (m, 2H), 7.48 (d, J = 8.0 Hz, 4H), 7.40 – 7.30 (m, 5H), 7.23 (dd, J = 6.5, 1.9 Hz, 2H), 7.17 – 7.06 (m, 4H), 7.01 – 6.93 (m, 2H), 6.87 (d, J = 8.8 Hz, 1H), 5.58 (s, 1H), 5.44 – 5.35 (m, 1H), 5.14 (d, J = 1.0 Hz, 1H), 4.25 – 4.07 (m, 2H), 3.59 (s, 2H), 2.96 (d, J = 11.4 Hz, 2H), 2.64 (d, J = 9.9 Hz, 2H), 2.35 (s, 3H), 2.28 (dt, J = 14.0, 7.0 Hz, 1H), 2.10 (dt, J = 14.1, 7.1 Hz, 1H), 1.74 (s, 3H), 1.67 – 1.55 (m, 9H), 1.52 (s, 3H), 1.46 (s, 3H), 1.01 – 0.91 (m, 9H).

^{13}C NMR (126 MHz, $CDCl_3$) δ 168.85, 162.83, 160.45, 156.86, 152.57, 146.41, 146.08, 144.92, 140.91, 139.07, 138.65, 136.09, 132.87, 129.03, 128.19, 127.97, 127.88, 127.79, 127.12, 127.05, 125.51, 125.15, 124.98, 124.55, 122.97, 122.23, 120.56, 119.67, 113.04, 98.14, 92.74, 65.08, 50.08, 46.67, 44.11, 40.43, 33.55, 31.59, 31.17, 26.80, 25.37, 24.15, 22.80, 22.58, 21.42, 14.90, 7.93.

HRMS (ESI): $C_{56}H_{65}ClN_3O_4^-$, calcd. 878.4669, found 878.4663.

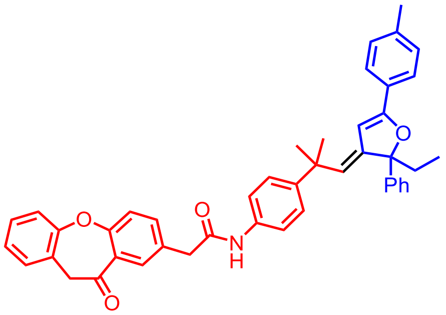

5lc

(E)-N-(4-(1-(2-ethyl-2-phenyl-5-(p-tolyl)furan-3(2H)-ylidene)-2-methylpropan-2-yl)phenyl)-2-(11-oxo-10,11-dihydrodibenzo[b,f]oxepin-2-yl)acetamide (5lc) Yellow solid, 48.8 mg.

^{1}H NMR (400 MHz, $CDCl_3$) δ 8.19 (d, *J* = 2.4 Hz, 1H), 7.92 (dd, *J* = 7.7, 1.4 Hz, 1H), 7.60 (td, *J* = 7.4, 1.4 Hz, 1H), 7.52 (ddd, *J* = 7.6, 6.1, 1.9 Hz, 2H), 7.49 – 7.42 (m, 4H), 7.40 (d, *J* = 7.4 Hz, 1H), 7.38 – 7.30 (m, 4H), 7.25 (t, *J* = 7.5 Hz, 3H), 7.18 (s, 1H), 7.12 (dd, *J* = 15.6, 8.2 Hz, 3H), 5.51 (s, 1H), 5.23 (s, 2H), 5.10 (s, 1H), 3.75 (s, 2H), 2.36 (s, 3H), 2.26 (dq, *J* = 14.3, 7.2 Hz, 1H), 2.07 (dq, *J* = 14.2, 7.1 Hz, 1H), 1.47 (s, 3H), 1.41 (s, 3H), 0.94 (t, *J* = 7.2 Hz, 3H).

^{13}C NMR (101 MHz, $CDCl_3$) δ 190.83, 168.71, 160.74, 160.51, 147.04, 146.19, 144.84, 140.37, 139.09, 136.42, 135.47, 134.98, 132.92, 132.56, 129.52, 129.34, 129.01, 128.31, 128.16, 127.88, 127.72, 127.10, 126.92, 125.49, 125.36, 124.90, 124.34, 121.75, 119.67, 98.01, 92.70, 73.66, 43.72, 40.34, 33.45, 31.54, 31.06, 21.41, 7.87.

HRMS (ESI): $C_{45}H_{40}NO_4^-$, calcd. 658.2963, found 658.2949.

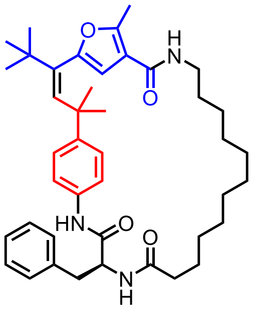

6a

6a: White solid.

^{1}H NMR (500 MHz, $CDCl_3$) δ7.87 (s, 1H), 7.36 – 7.31 (m, 2H), 7.29 – 7.22 (m, 3H), 7.10 (dd, *J* = 23.0, 8.7 Hz, 4H), 6.38 (d, *J* = 7.4 Hz, 1H), 6.10 (s, 1H), 5.68 (t, *J* = 5.5 Hz, 1H), 5.31 (s, 1H), 4.85 (dd, *J* = 13.9, 7.7 Hz, 1H), 3.32 (dd, *J* = 13.5, 7.0 Hz, 2H), 3.23 (dd, *J* = 13.6, 6.0 Hz, 1H), 3.10 (dd, *J* = 13.7, 8.1 Hz, 1H), 2.41 (s, 3H), 2.35 – 2.19 (m, 2H), 1.68 (dt, *J* = 15.2, 7.0 Hz, 2H), 1.57 (m, 2H), 1.38 – 1.31 (m, 14H), 1.28 (d, *J* = 3.4 Hz, 6H), 1.09 (s, 9H).

^{13}C NMR (126 MHz, $CDCl_3$) δ 173.40, 169.13, 167.04, 164.28, 154.10, 148.54, 147.40, 141.11, 136.57, 134.00, 129.36, 128.85, 127.22, 126.82, 120.16, 115.97, 108.12, 55.17, 39.69, 39.47, 38.75, 36.41, 36.22, 31.39, 30.96, 29.70, 29.48, 28.12, 28.00, 27.66, 27.61, 27.39, 27.25, 26.43, 24.68, 13.40.

MS (ESI): $C_{42}H_{58}N_3O_4^+$, calcd. 668.4422, found 668.4425.

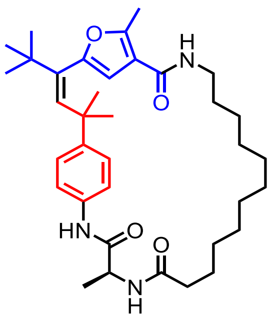

6b

6b: White solid.

^{1}H NMR (500 MHz, $CDCl_3$) δ 8.43 (s, 1H), 7.33 (d, *J* = 7.9 Hz, 2H), 7.12 (d, *J* = 8.1 Hz, 2H), 6.25 (d, *J* = 6.9 Hz, 1H), 6.09 (s, 1H), 5.63 (t, *J* = 5.9 Hz, 1H), 5.38 (s, 2H), 4.67 (dd, *J* = 14.3, 7.4 Hz, 1H), 3.33 (dd, *J* = 13.1, 6.6 Hz, 2H), 2.43 (s, 3H), 2.26 – 2.21 (m, 2H), 2.09 – 2.00 (m, 2H), 1.72 – 1.67 (m, 2H), 1.49 (d, *J* = 6.8 Hz, 3H), 1.37 – 1.32 (m, 14H), 1.28 (s, 6H), 1.07 (s, 9H).

^{13}C NMR (126 MHz, $CDCl_3$) δ173.63, 170.35, 164.28, 154.28, 148.63, 141.05, 139.54, 134.65, 129.74, 126.82, 119.84, 115.91, 108.03, 49.44, 39.82, 39.36, 36.42, 36.21, 31.39, 30.79, 29.70, 29.36, 28.17, 28.06, 27.77, 27.56, 27.48, 27.23, 26.32, 24.76, 22.69, 17.97, 14.11, 13.36.

MS (ESI): $C_{36}H_{54}N_3O_4^+$, calcd. 592.4109, found 592.4106.

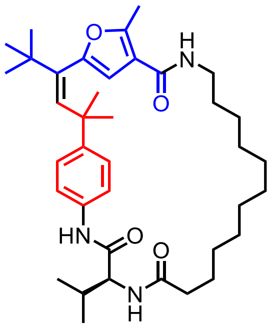

6c

6c: White solid.

^{1}H NMR (500 MHz, $CDCl_3$) δ 8.38 (s, 1H), 7.33 (d, *J* = 8.6 Hz, 2H), 7.12 (d, *J* = 8.6 Hz, 2H), 6.27 (d, *J* = 8.7 Hz, 1H), 6.09 (s, 1H), 5.76 (t, *J* = 6.0 Hz, 1H), 5.45 (s, 1H), 4.55-4.47 (m, 1H), 3.40-3.27 (m, 2H), 2.41 (s, 3H), 2.38-2.32 (m, 1H), 2.30-2.18

(m, 2H), 1.73-1.67 (m, 2H), 1.62-1.55 (m, 2H), 1.38-1.30 (m, 14H), 1.28 (s, 6H), 1.11-1.06 (m, 12H), 1.05 (d, J = 6.8 Hz, 3H).

^{13}C NMR (126 MHz, $CDCl_3$) δ 173.55, 169.91, 164.34, 154.02, 148.60, 147.07, 140.98, 139.72, 134.42, 126.78, 120.28, 116.09, 108.21, 58.96, 39.78, 39.43, 36.42, 34.68, 31.59, 29.72, 29.39, 28.10, 27.78, 26.93, 26.34, 25.29, 24.80, 22.66, 19.30, 18.47, 14.12, 13.36.

HRMS (ESI): $C_{38}H_{58}N_3O_4^+$, calcd. 620.4422, found 620.4419.

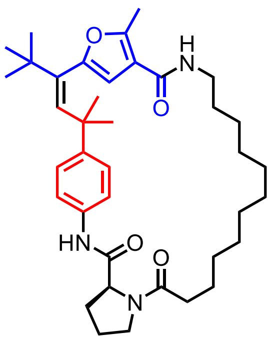

6d

6d: White solid.

^{1}H NMR (500 MHz, $CDCl_3$) δ 9.65 (s, 1H), 7.39 (d, J = 8.7 Hz, 2H), 7.14 (d, J = 8.7 Hz, 2H), 6.06 (s, 1H), 5.53 (t, J = 5.6 Hz, 1H), 5.48 (s, 1H), 4.80 (t, J = 9.4 Hz, 1H), 3.75 (q, J = 7.0 Hz, 1H), 3.62-3.53 (m, 1H), 3.51-3.43 (m, 1H), 3.43-3.35 (m, 1H), 3.35-3.24 (m, 1H), 2.63 (dd, J = 12.4, 6.5 Hz, 1H), 2.50 (s, 3H), 2.42-2.32 (m, 2H), 2.22-2.03 (m, 2H), 1.92-1.81 (m, 1H), 1.78-1.68 (m, 2H), 1.63-1.55 (m, 2H), 1.41-1.33 (m, 14H), 1.30 (s, 6H), 1.07 (s, 9H).

^{13}C NMR (126 MHz, $CDCl_3$) δ 174.47, 169.12, 164.16, 154.78, 148.86, 146.41, 140.76, 138.88, 135.64, 126.50, 119.56, 115.67, 107.74, 60.44, 47.70, 40.03, 39.27, 36.49, 34.32, 30.85, 30.69, 29.72, 29.25, 28.31, 28.24, 28.06, 27.88, 27.58, 26.46, 26.28, 25.14, 23.87, 13.39.

MS (ESI): $C_{38}H_{56}N_3O_4^+$, calcd. 618.4265, found 618.4270.

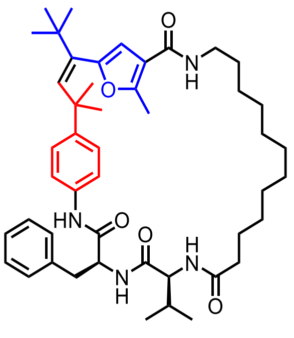

6e

6e: White solid.

^{1}H NMR (500 MHz, $CDCl_3$) δ 8.33 (s, 1H), 7.44 – 7.32 (m, 5H), 7.27 (d, *J* = 7.2 Hz, 2H), 7.11 (d, *J* = 8.6 Hz, 2H), 6.49 – 6.08 (m, 1H), 6.08 (d, *J* = 7.0 Hz, 1H), 6.06 (s, 1H), 5.62 (s, 1H), 5.48 (s, 1H), 4.90 (dd, *J* = 15.3, 6.9 Hz, 1H), 3.88 (t, *J* = 7.6 Hz, 1H), 3.49 – 3.40 (m, 1H), 3.33 – 3.22 (m, 3H), 2.43 (s, 3H), 2.33 – 2.16 (m, 4H), 2.06 – 1.97 (m, 1H), 1.60 – 1.55 (m, 2H), 1.35 (s, 6H), 1.31 – 1.26 (m, 14H), 1.07 (s, 9H), 0.94 (d, *J* = 6.7 Hz, 3H), 0.78 (d, *J* = 6.7 Hz, 3H).

^{13}C NMR (126 MHz, $CDCl_3$) δ 174.56, 171.68, 168.94, 164.28, 154.68, 148.75, 146.92, 141.00, 138.92, 136.66, 134.77, 129.33, 128.86, 127.17, 126.51, 120.38, 115.70, 107.83, 60.35, 54.79, 40.01, 39.13, 37.78, 36.44, 36.16, 31.17, 30.35, 29.93, 29.68, 29.08, 28.52, 28.42, 28.34, 28.10, 26.25, 25.24, 18.99, 18.69, 13.41.

MS (ESI): $C_{47}H_{67}N_4O_5^+$, calcd. 767.5106, found 767.5106.

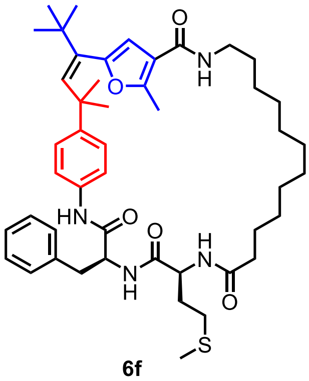

6f

6f: White solid.

^{1}H NMR (500 MHz, MeOD) δ 7.45 (d, *J* = 8.6 Hz, 2H), 7.33-7.29 (m, 3H), 7.28-7.21 (m, 2H), 7.14 (d, *J* = 8.7 Hz, 2H), 6.13 (s, 1H), 6.11 (d, *J* = 1.6 Hz, 1H), 4.85-4.82 (m, 1H), 4.28 (t, *J* = 7.2 Hz, 1H), 3.47-3.39 (m, 2H), 3.24-3.19 (m, 1H), 2.96-2.89 (m, 1H), 2.35 (s, 3H), 2.29-2.17 (m, 4H), 2.15-2.04 (m, 2H), 2.01 (s, 3H), 1.83-1.74 (m, 2H), 1.65-1.50 (m, 6H), 1.37 (s, 6H), 1.35-1.30 (m, *J* = 10.3, 5.2 Hz, 14H), 1.29 (s, 6H), 1.09 (s, 9H).

^{13}C NMR (126 MHz, MeOD) δ 175.24, 172.89, 170.07, 165.14, 154.79, 148.57, 146.27, 141.02, 138.94, 137.31, 134.94, 128.90, 128.20, 126.50, 126.07, 120.37, 115.45, 108.41, 54.95, 53.38, 39.73, 38.65, 37.30, 35.90, 34.98, 30.42, 30.34, 30.08, 29.93, 29.38, 29.26, 28.77, 28.57, 28.52, 28.27, 26.13, 25.18, 13.71, 12.24.

MS (ESI): $C_{47}H_{67}N_4O_5S^+$, calcd. 799.4827, found 799.4844.

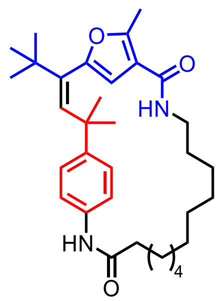

6g

6g: White solid.

^{1}H NMR (400 MHz, $CDCl_3$) δ 7.31 (s, 1H), 7.21 (d, J = 8.5 Hz, 2H), 7.04 (d, J = 8.5 Hz, 2H), 6.13 (s, 1H), 5.84 (m, 1H), 5.24 (s, 1H), 3.31 (dd, J = 14.3, 6.4 Hz, 2H), 2.42 – 2.31 (m, 5H), 1.80 – 1.73 (m, 2H), 1.62 – 1.50 (m, 2H), 1.48 – 1.34 (m, 14H), 1.32 (s, 6H), 1.05 (s, J = 13.3 Hz, 9H).

^{13}C NMR (126 MHz, $CDCl_3$) δ 171.65, 164.49, 153.32, 148.41, 147.00, 141.74, 140.43, 134.42, 126.98, 120.38, 116.32, 108.47, 77.26, 39.57, 39.34, 36.87, 36.30, 31.51, 29.63, 29.21, 27.18, 27.12, 26.88, 26.65, 26.22, 25.87, 25.50, 24.28, 13.35.

HRMS (ESI): $C_{33}H_{49}N_2O_3^+$, calcd. 520.2135, found 520.2128.

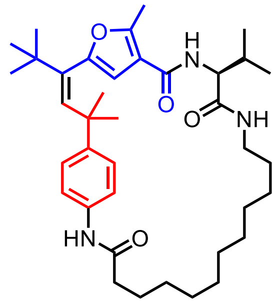

6h

6h: White solid.

^{1}H NMR (500 MHz, $CDCl_3$) δ 9.50 (s, 1H), 7.35 (d, J = 8.6 Hz, 2H), 7.00 (d, J = 8.6 Hz, 2H), 6.23 (s, 1H), 6.13 (dd, J = 7.9, 3.9 Hz, 1H), 5.42 (d, J = 9.3 Hz, 1H), 4.39 (s, 1H), 4.11 (t, J = 9.1 Hz, 1H), 3.75 (dq, J = 15.9, 7.9 Hz, 1H), 3.07 – 2.99 (m, 1H), 2.48 (s, 3H), 2.42 (ddd, J = 14.0, 10.0, 6.7 Hz, 1H), 2.36 – 2.27 (m, 1H), 1.96 (td, J = 13.4, 6.7 Hz, 1H), 1.88 – 1.73 (m, 2H), 1.61 – 1.55 (m, 2H), 1.46 – 1.24 (m, 20H), 1.01 (d, J = 6.7 Hz, 3H), 0.99 (s, 9H), 0.94 (d, J = 6.7 Hz, 3H).

^{13}C NMR (126 MHz, $CDCl_3$) δ 172.29, 171.84, 164.10, 155.13, 148.56, 148.07, 143.10, 140.87, 135.57, 126.94, 120.22, 114.87, 107.33, 77.22, 59.07, 38.83, 38.55, 37.09, 36.28, 33.96, 31.19, 29.56, 28.63, 28.50, 28.34, 27.65, 27.37, 27.10, 26.83, 25.11, 24.82, 19.30, 19.04, 13.46.

HRMS (ESI): $C_{38}H_{58}N_3O_4^+$, calcd. 620.2195, found 620.2191.

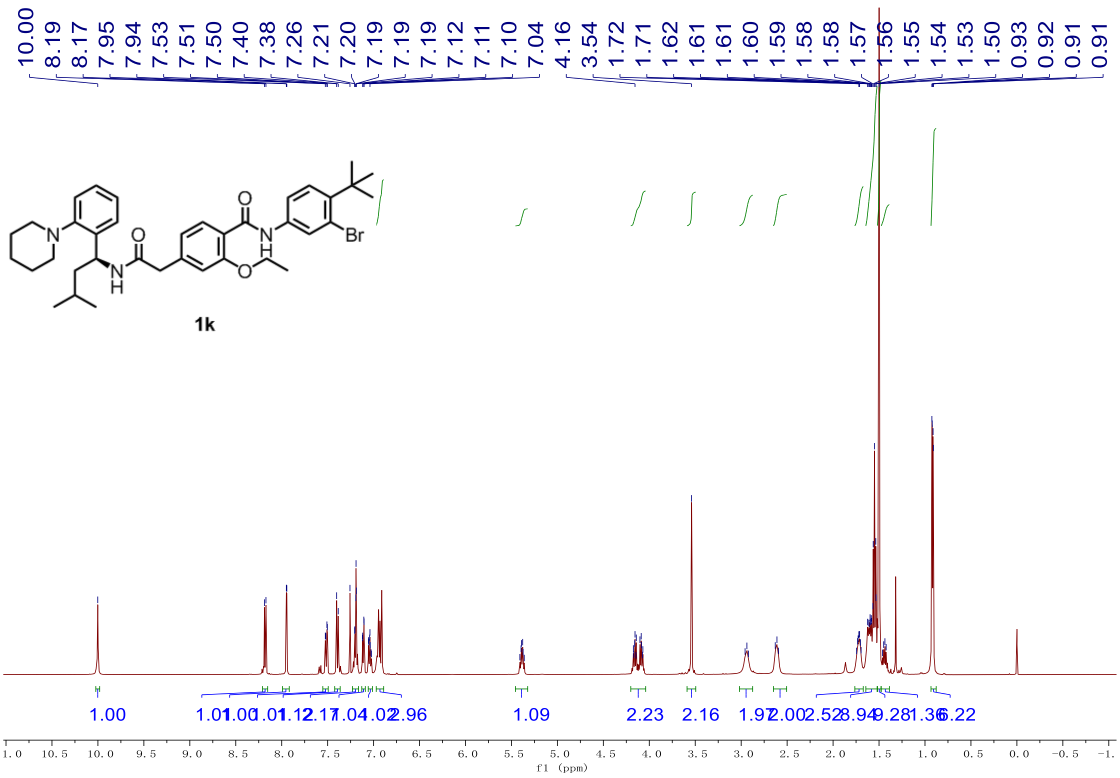

Supplementary Figure 5 ^{1}H NMR (400 MHz, $CDCl_3$) spectrum of compound **1k**

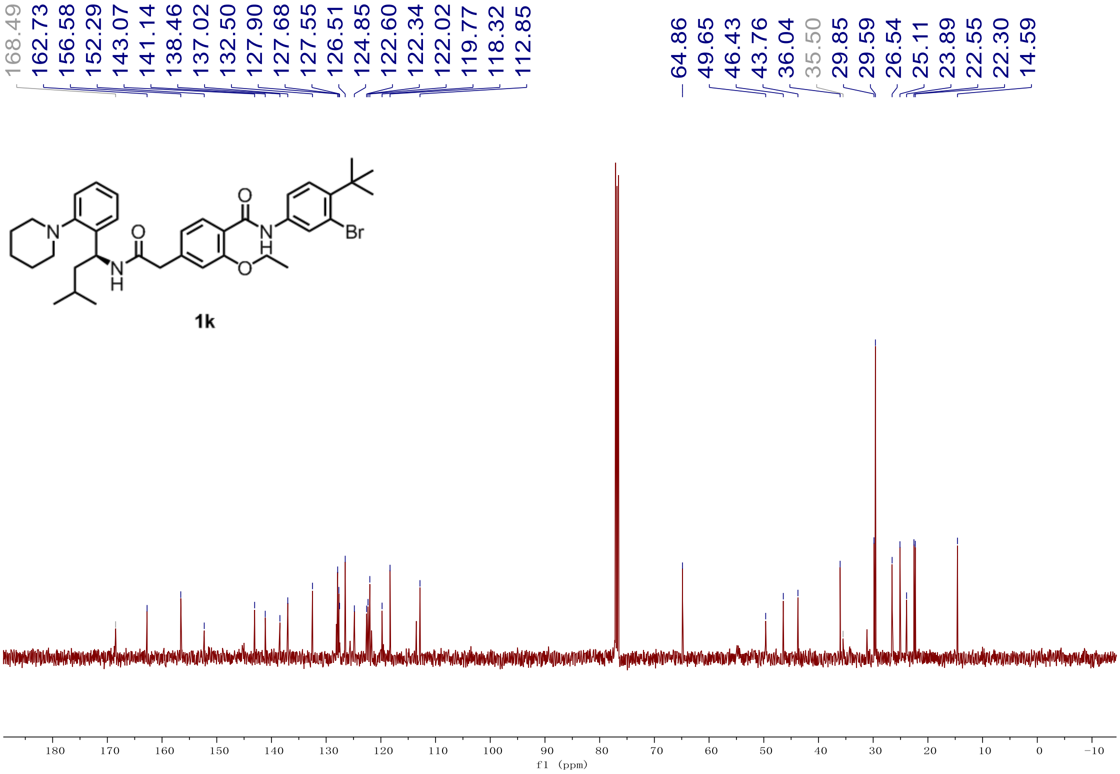

Supplementary Figure 6 ^{13}C NMR (101 MHz, $CDCl_3$) spectrum of compound **1k**

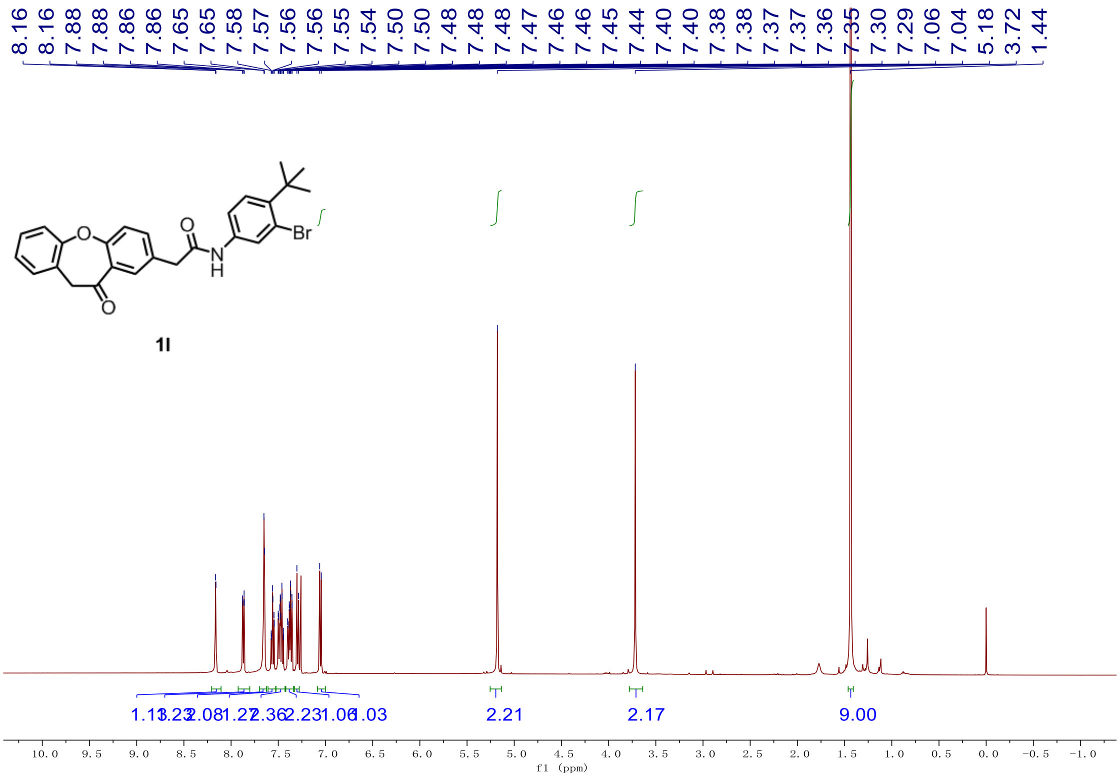

Supplementary Figure 7 ^{1}H NMR (400 MHz, $CDCl_3$) spectrum of compound **1l**

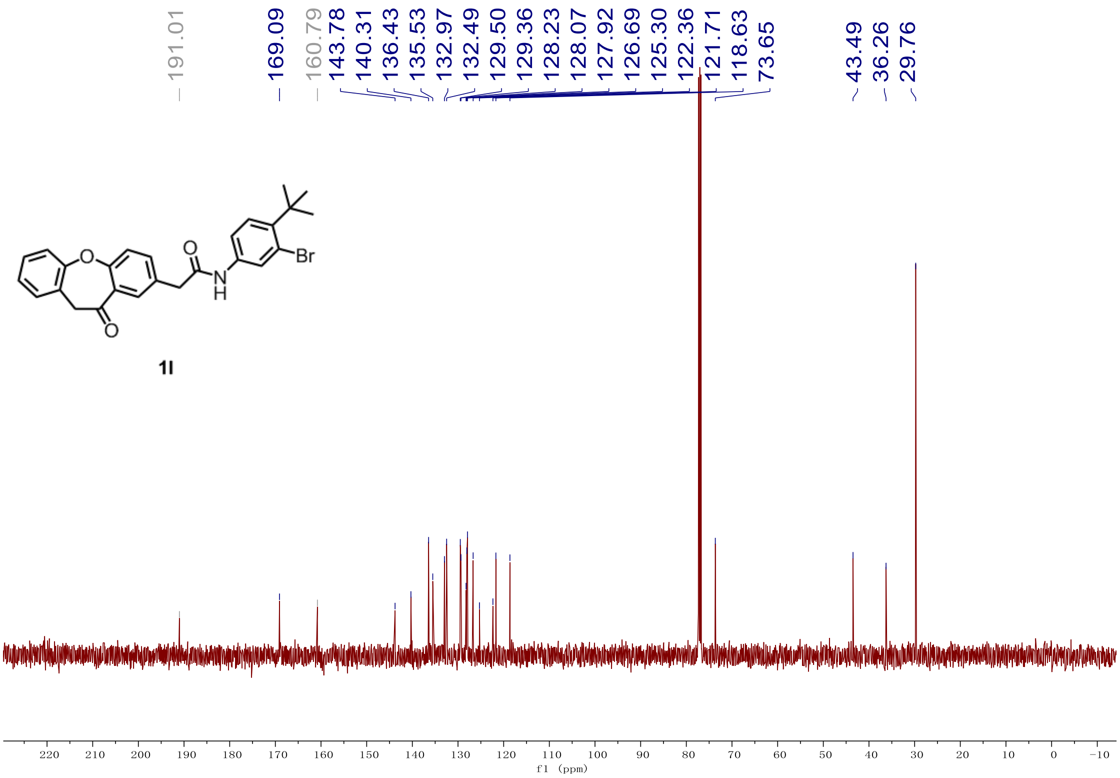

Supplementary Figure 8 ^{13}C NMR (101 MHz, $CDCl_3$) spectrum of compound **1l**

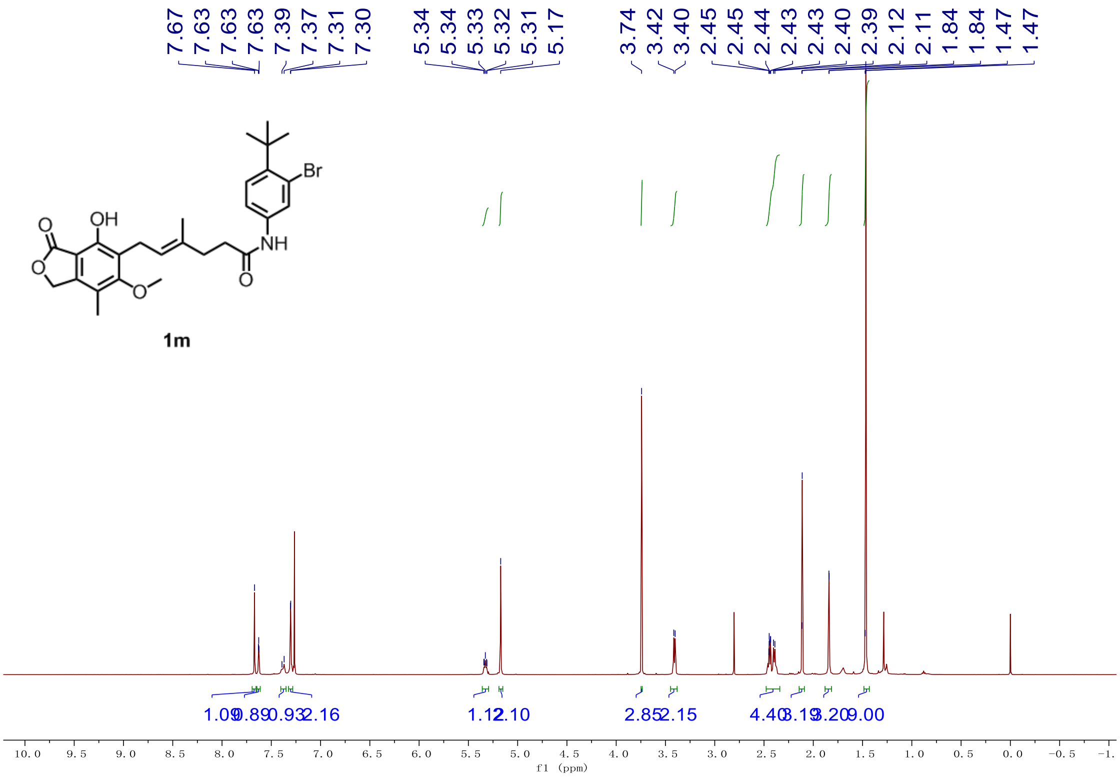

Supplementary Figure 9 ^{1}H NMR (400 MHz, $CDCl_3$) spectrum of compound **1m**

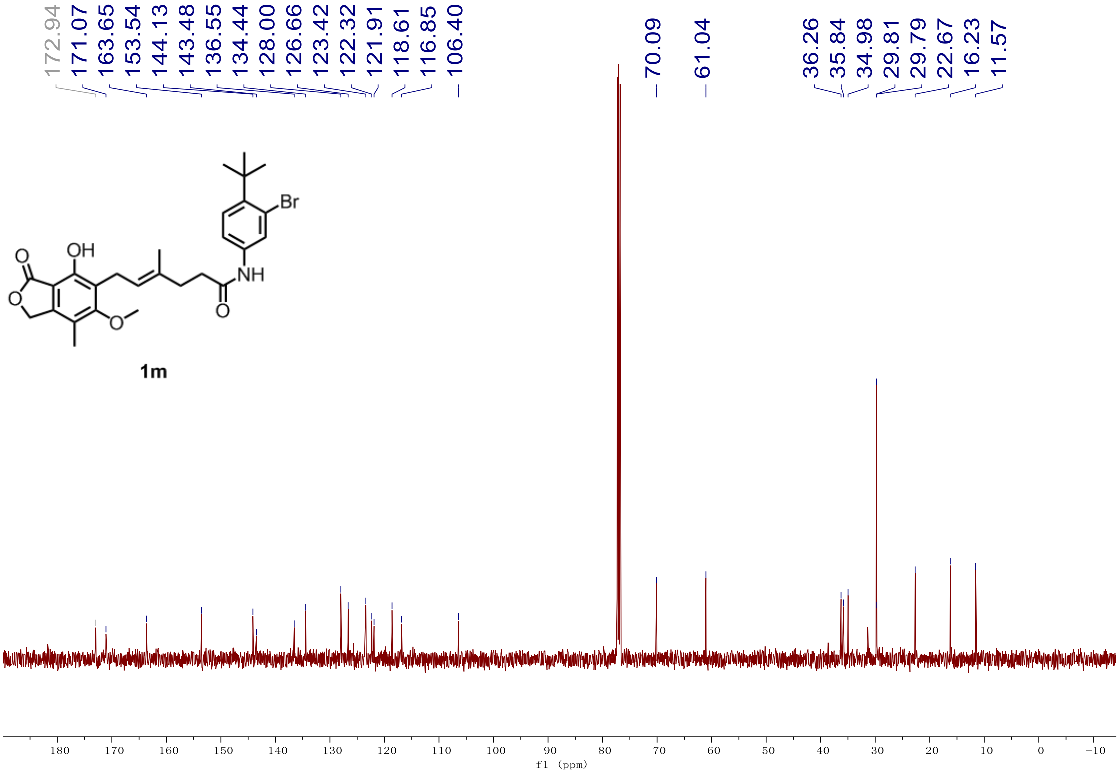

Supplementary Figure 10 ^{13}C NMR (101 MHz, $CDCl_3$) spectrum of compound **1m**

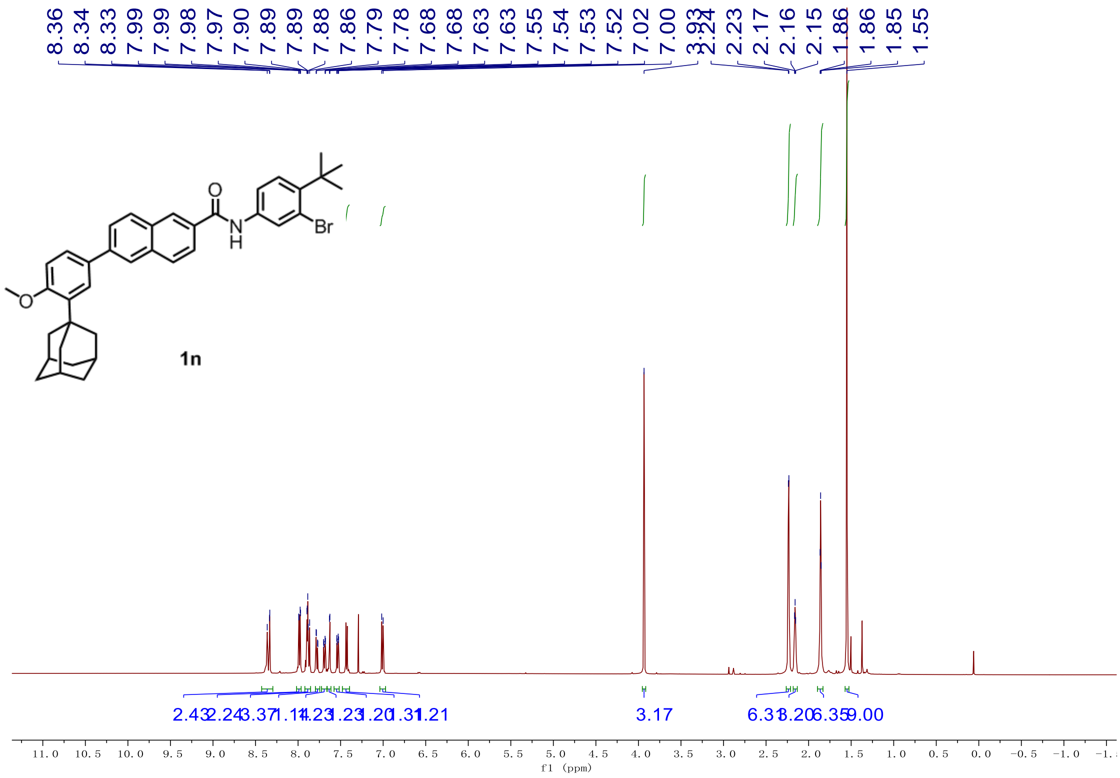

Supplementary Figure 11 ^{1}H NMR (400 MHz, $CDCl_3$) spectrum of compound **1n**

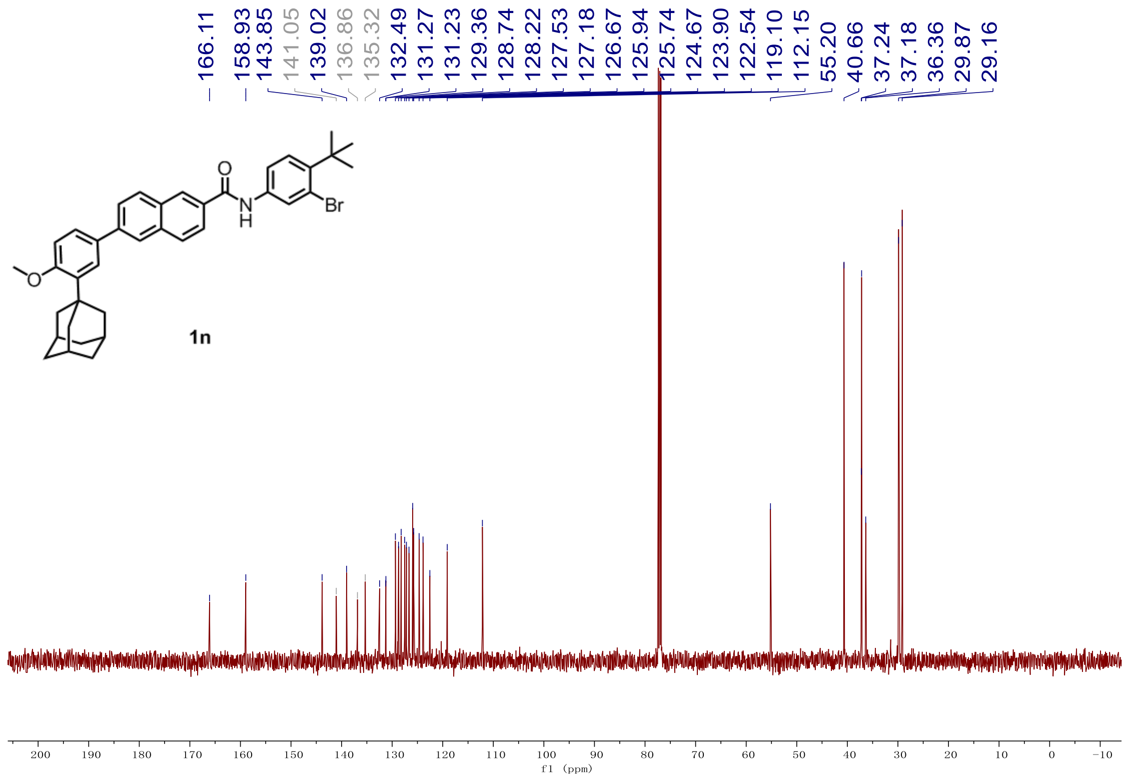

Supplementary Figure 12 ^{13}C NMR (101 MHz, $CDCl_3$) spectrum of compound **1n**

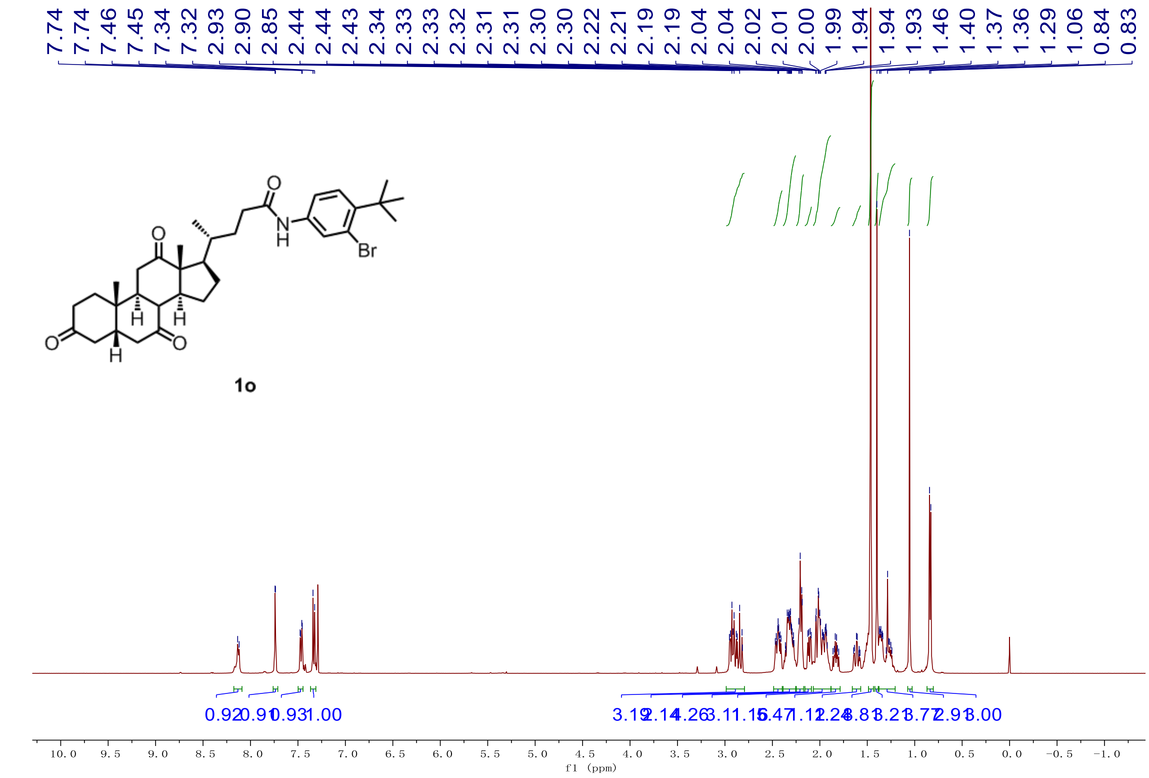

Supplementary Figure 13 ^{1}H NMR (400 MHz, $CDCl_3$) spectrum of compound **1o**

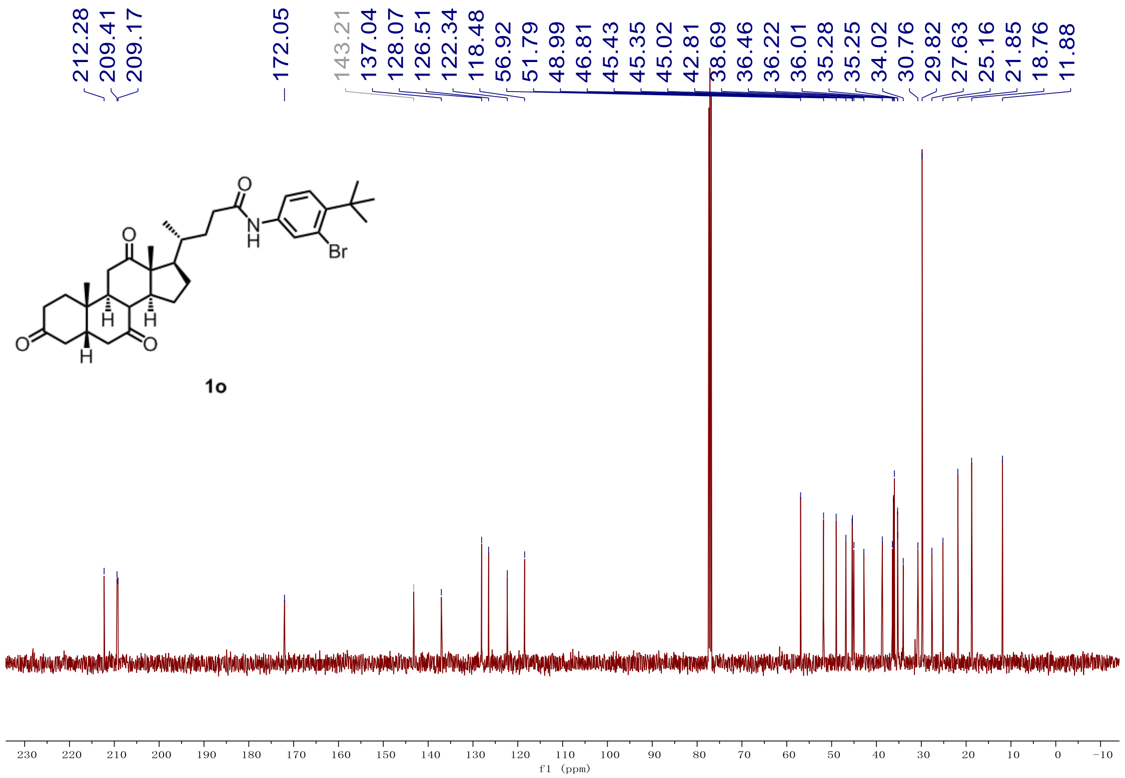

Supplementary Figure 14 ^{13}C NMR (101 MHz, $CDCl_3$) spectrum of compound **1o**

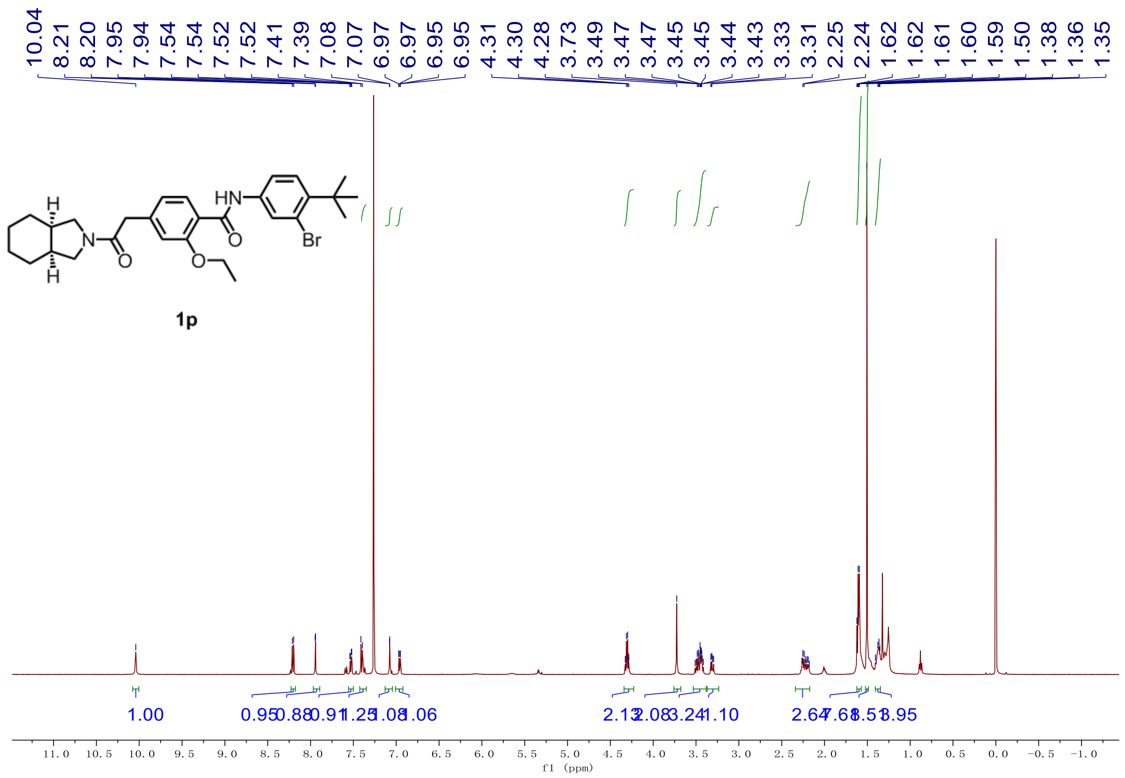

Supplementary Figure 15 ^{1}H NMR (400 MHz, $CDCl_3$) spectrum of compound **1p**

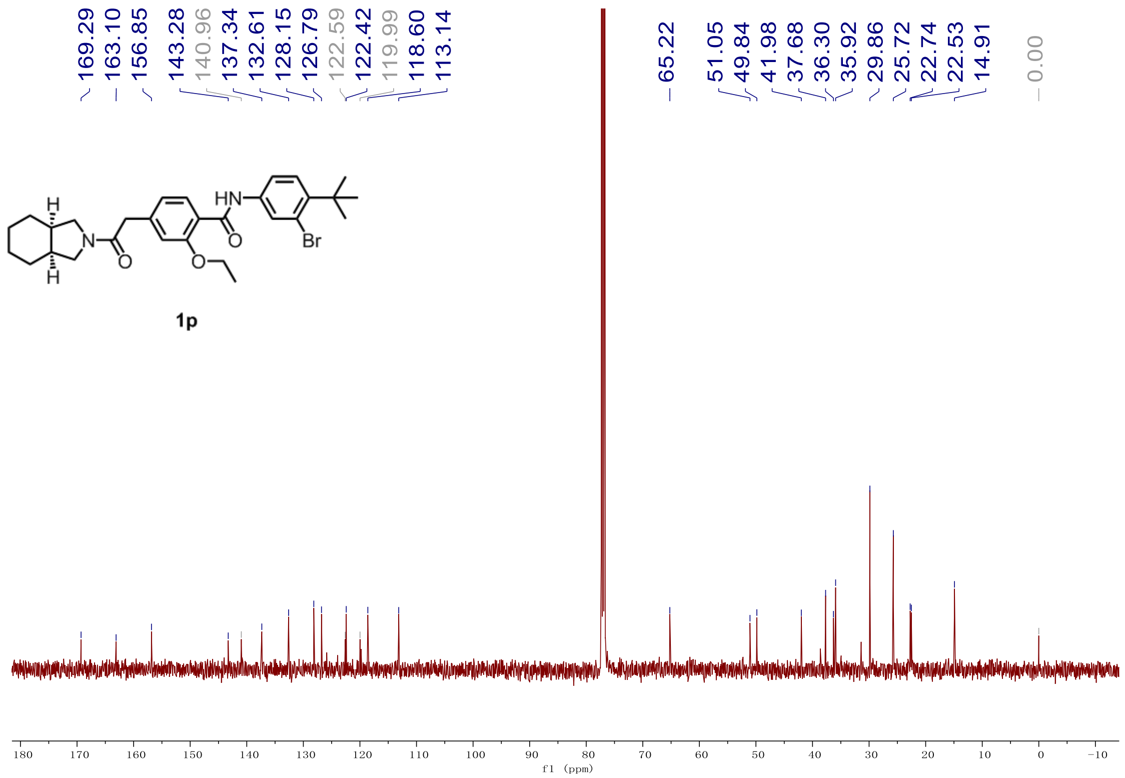

Supplementary Figure 16 ^{13}C NMR (101 MHz, $CDCl_3$) spectrum of compound **1p**

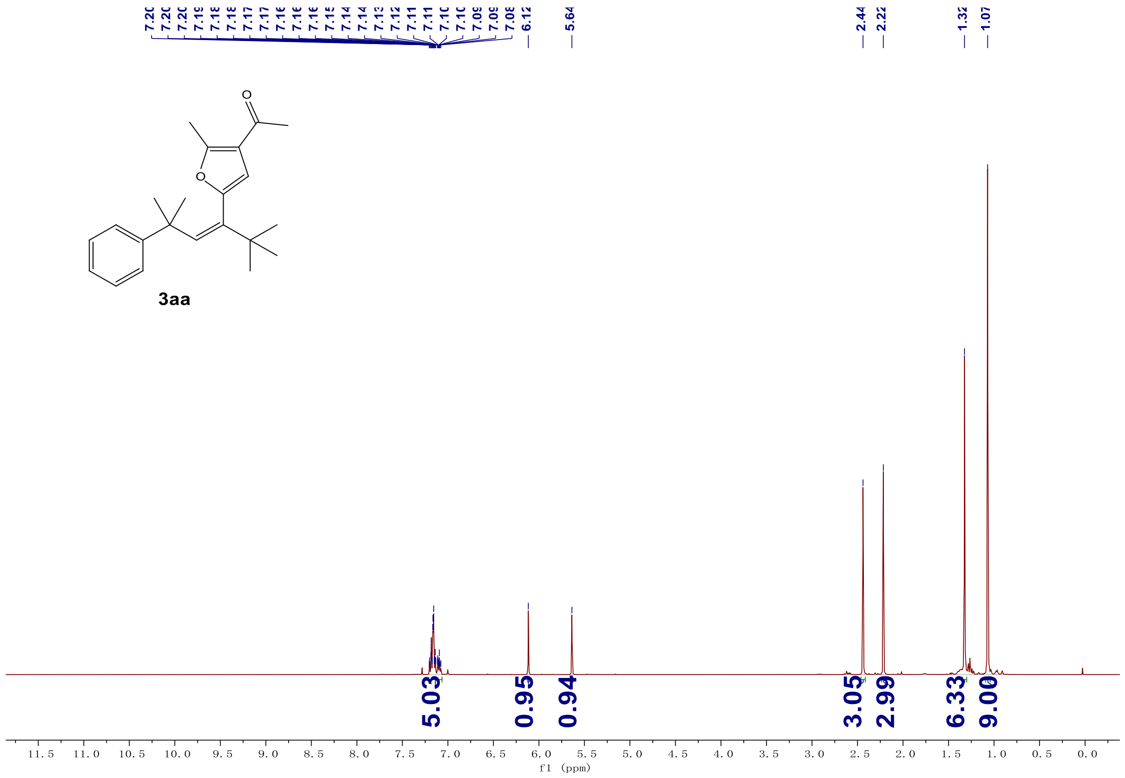

Supplementary Figure 17 ^{1}H NMR (400 MHz, $CDCl_3$) spectrum of compound **3aa**

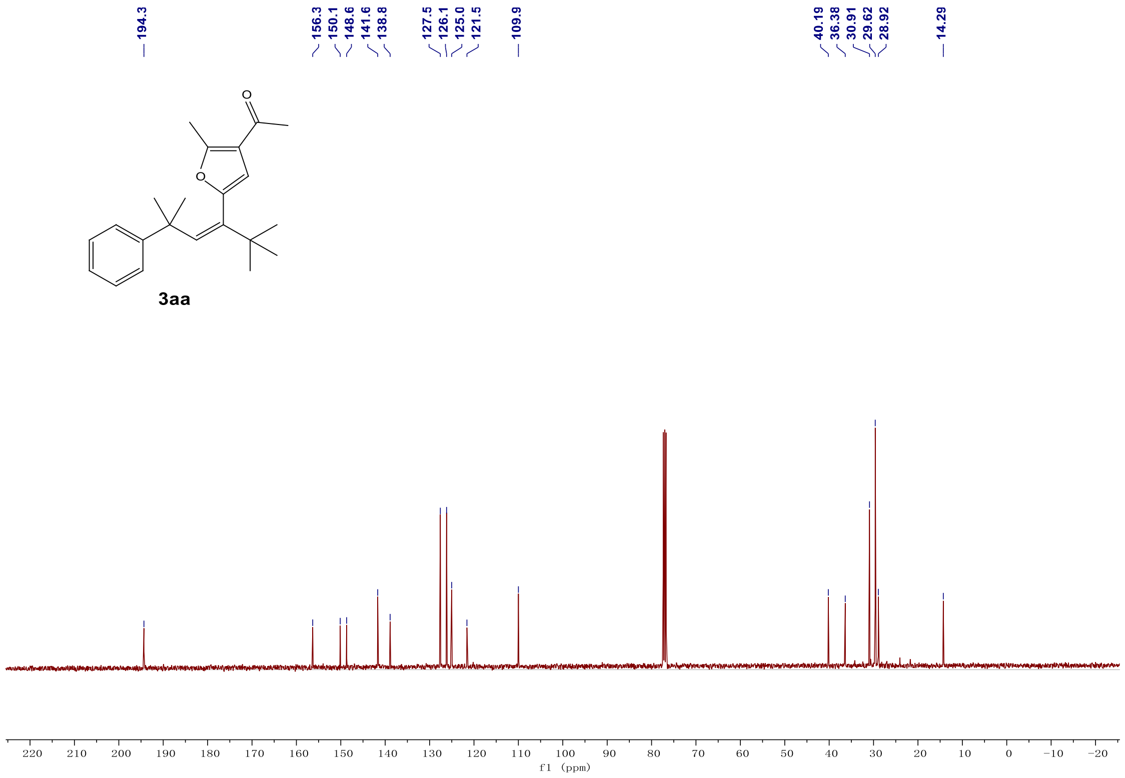

Supplementary Figure 18 ^{13}C NMR (101 MHz, $CDCl_3$) spectrum of compound **3aa**

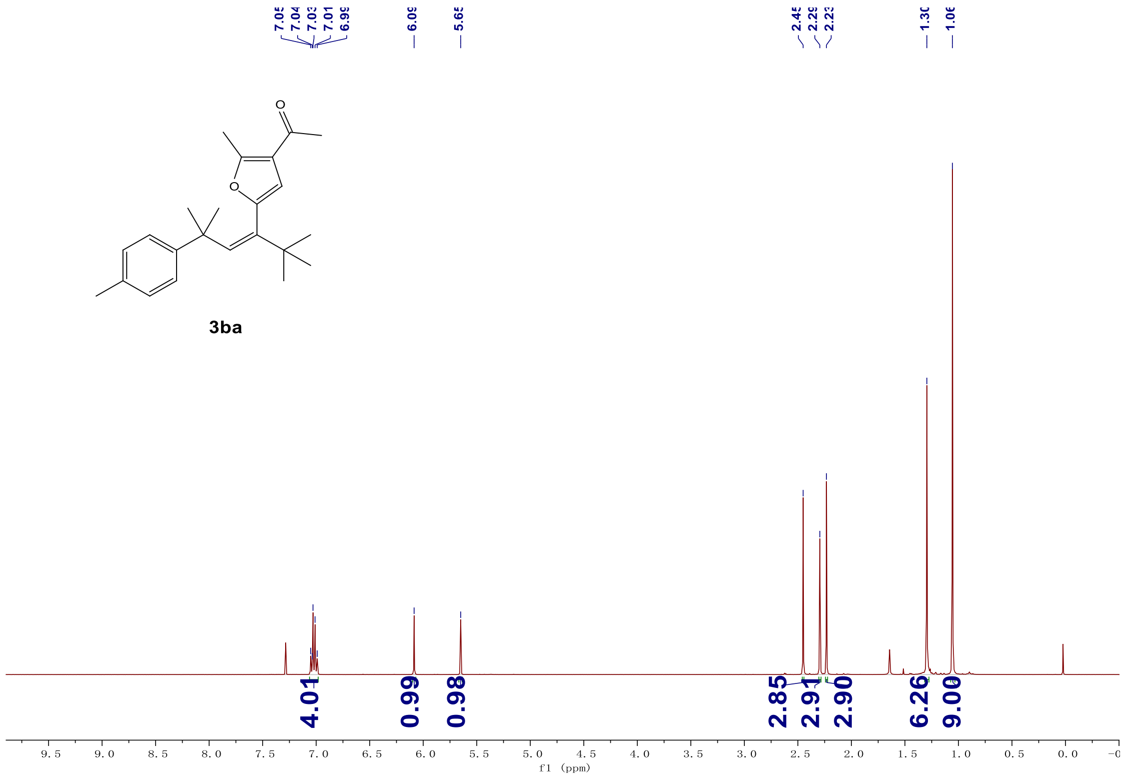

Supplementary Figure 19 ^{1}H NMR (400 MHz, $CDCl_3$) spectrum of compound **3ba**

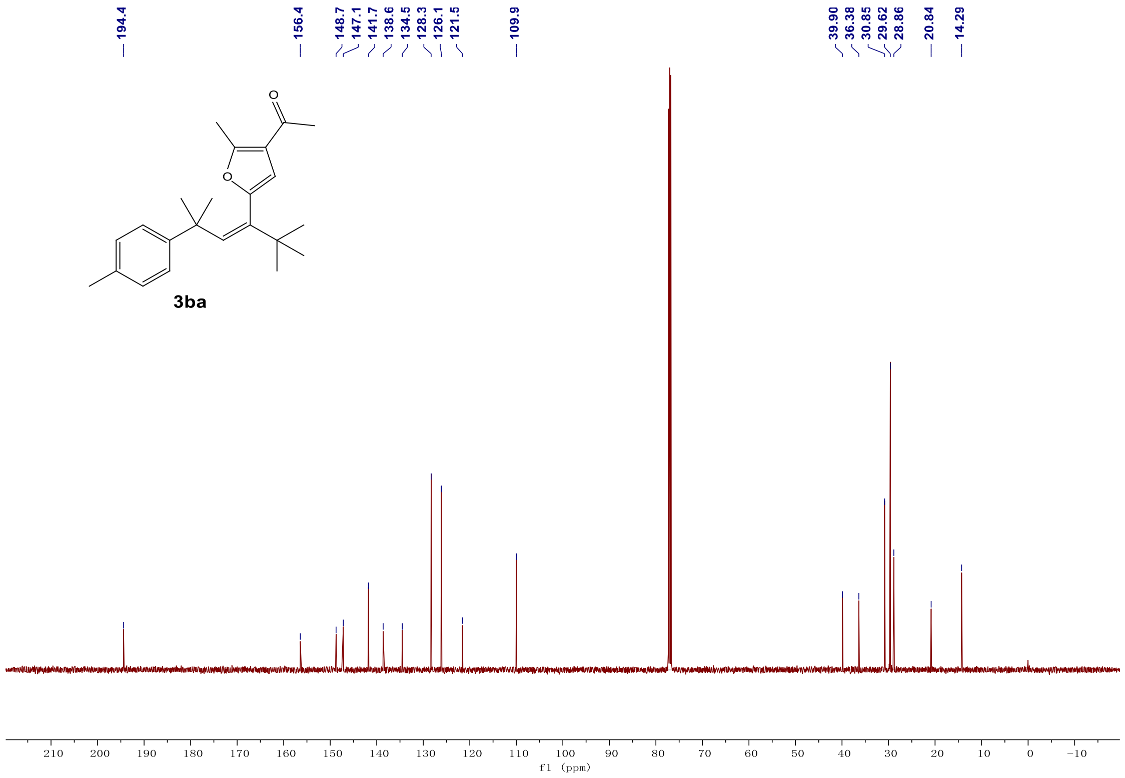

Supplementary Figure 20 ^{13}C NMR (101 MHz, $CDCl_3$) spectrum of compound **3ba**

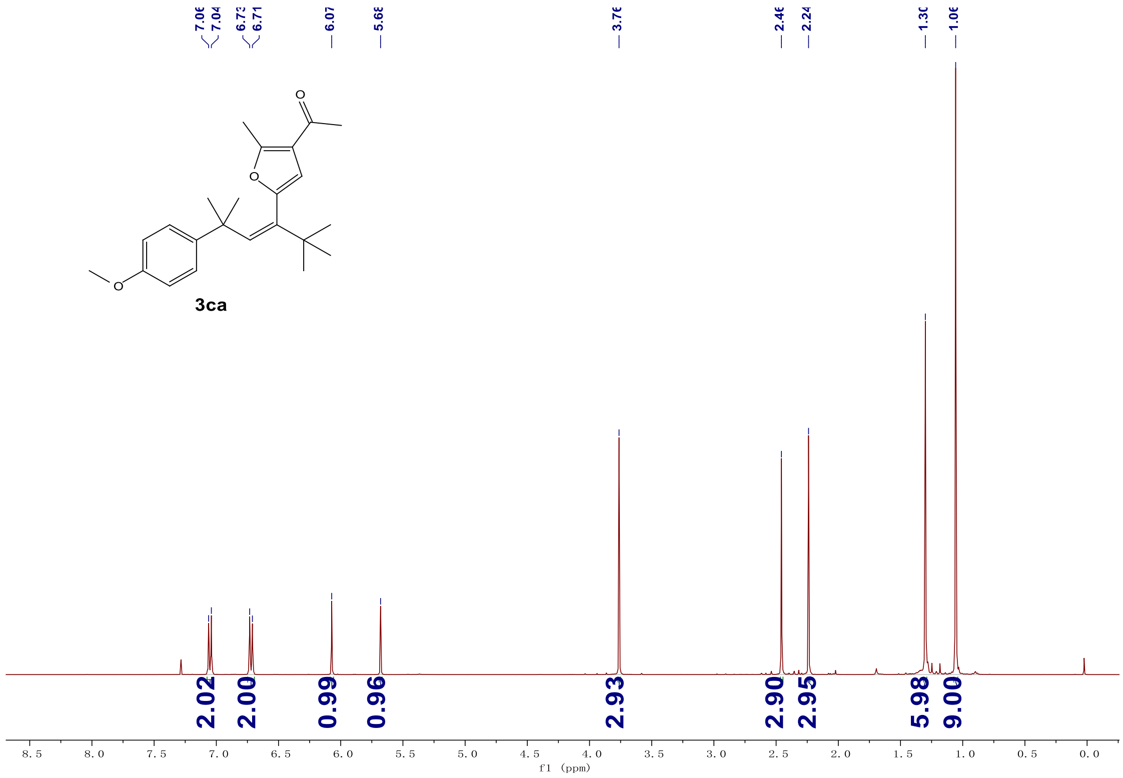

Supplementary Figure 21 ^{1}H NMR (400 MHz, $CDCl_3$) spectrum of compound **3ca**

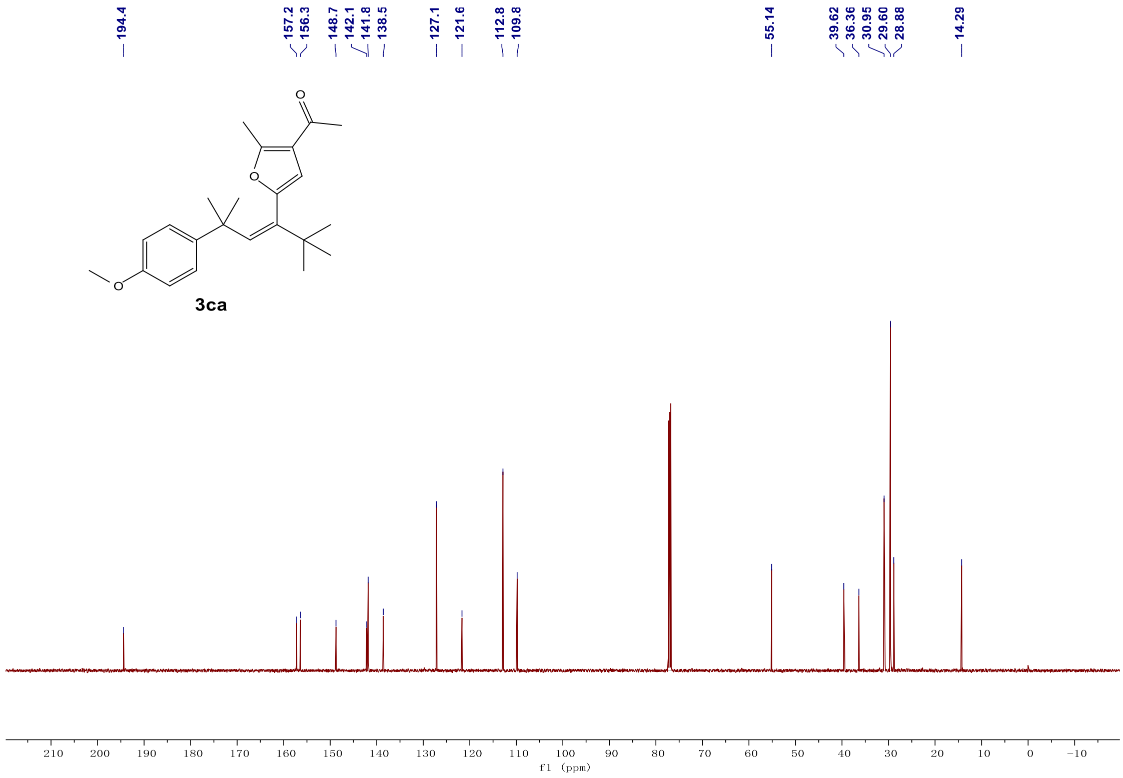

Supplementary Figure 22 ^{13}C NMR (101 MHz, $CDCl_3$) spectrum of compound **3ca**

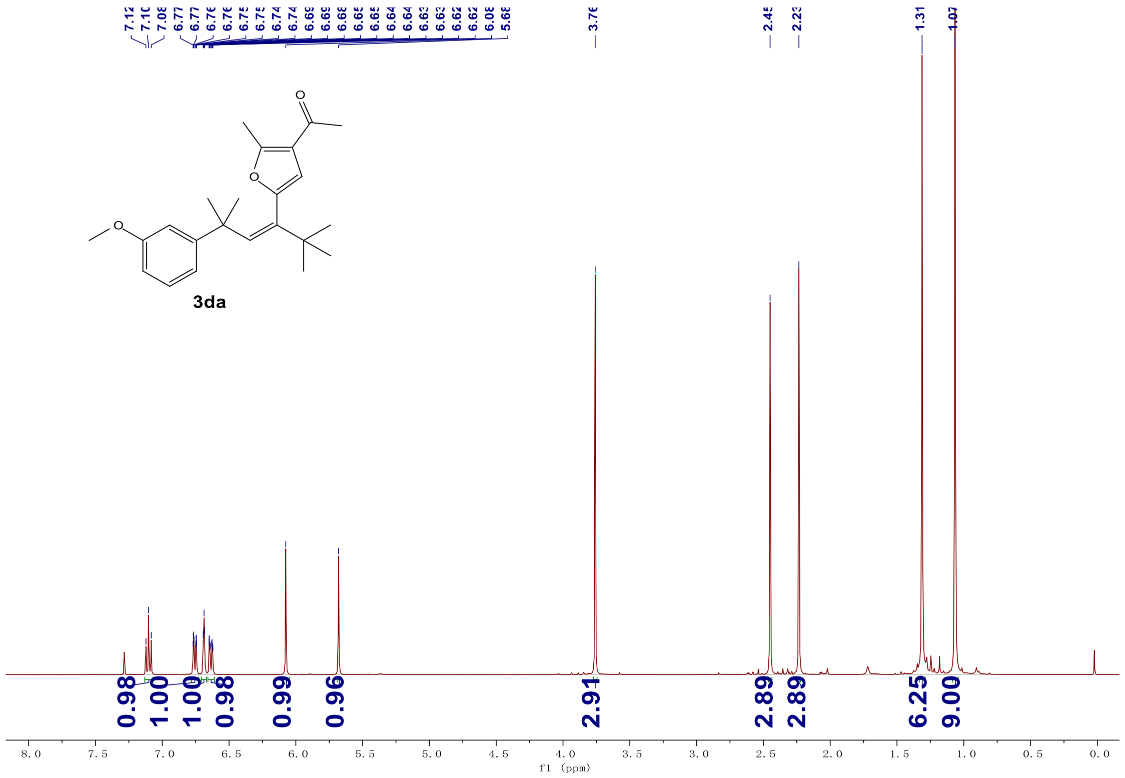

Supplementary Figure 23 ^{1}H NMR (400 MHz, $CDCl_3$) spectrum of compound **3da**

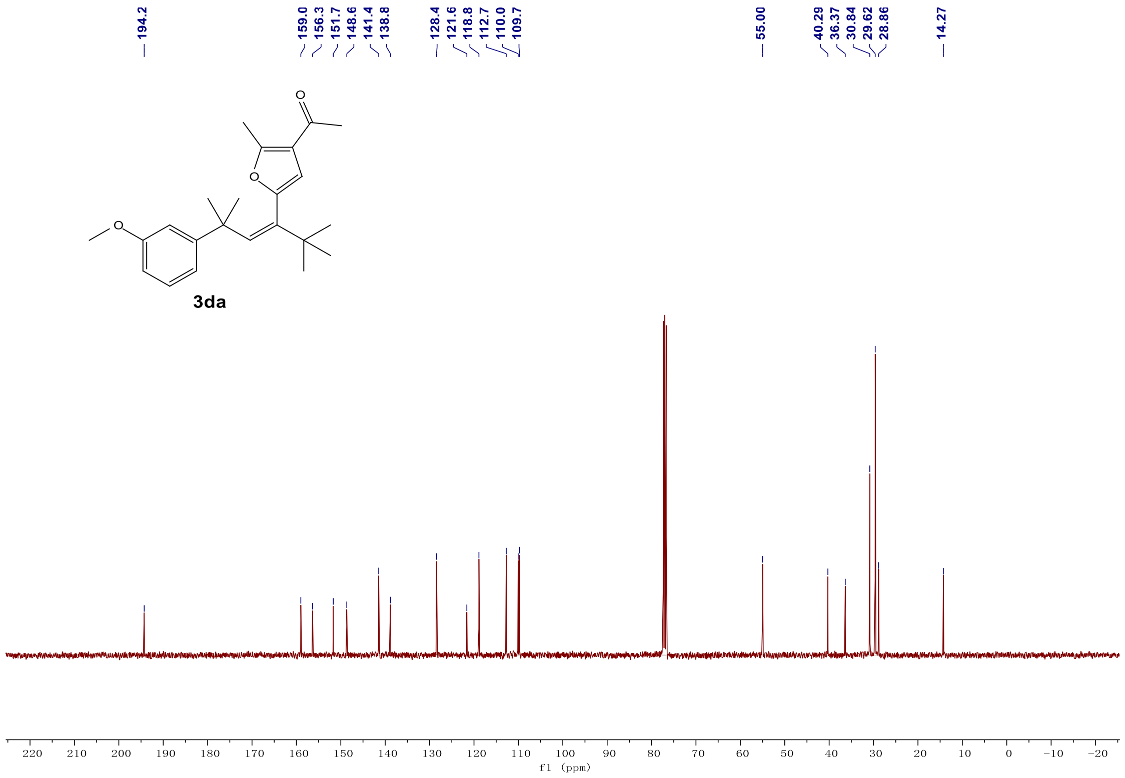

Supplementary Figure 24 ^{13}C NMR (101 MHz, $CDCl_3$) spectrum of compound **3da**

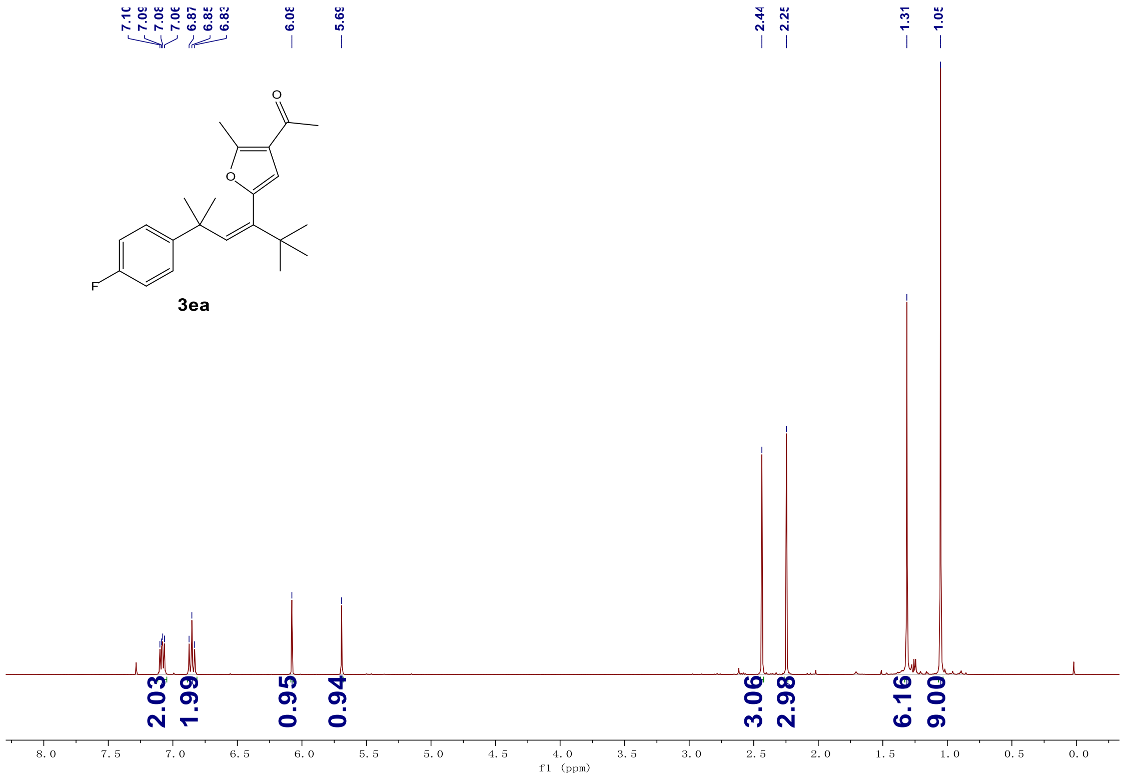

Supplementary Figure 25 ^{1}H NMR (400 MHz, $CDCl_3$) spectrum of compound **3ea**

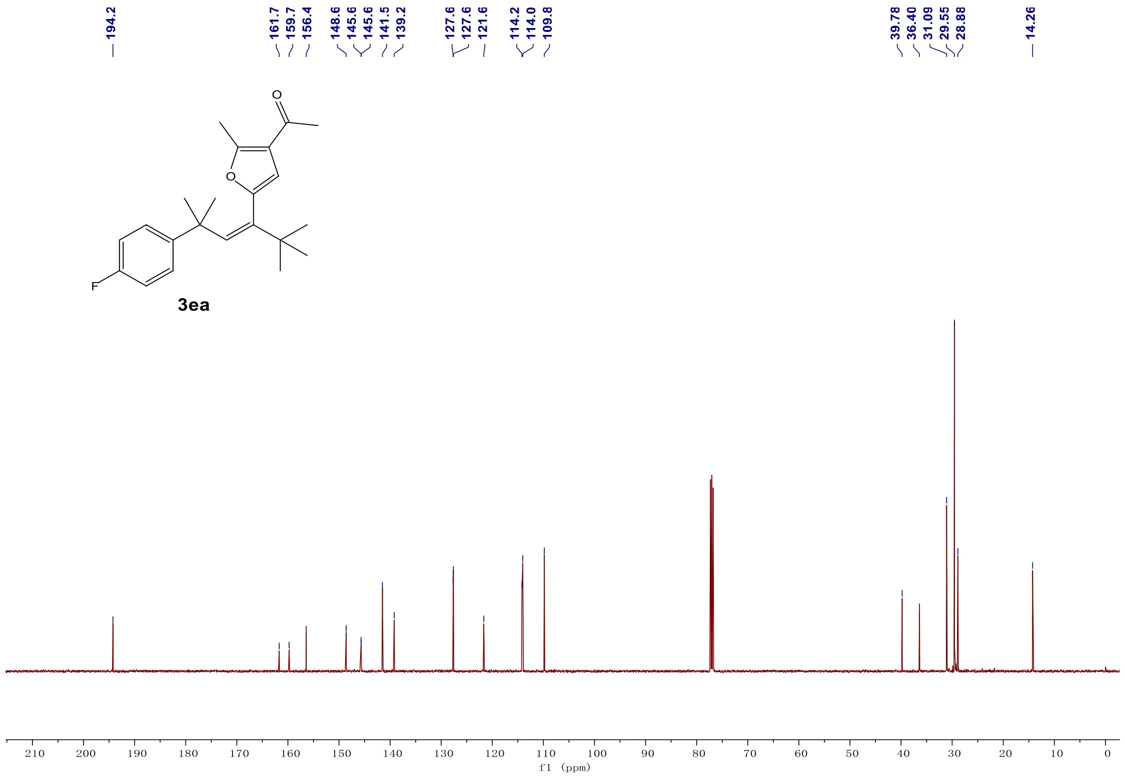

Supplementary Figure 26 ^{13}C NMR (101 MHz, $CDCl_3$) spectrum of compound **3ea**

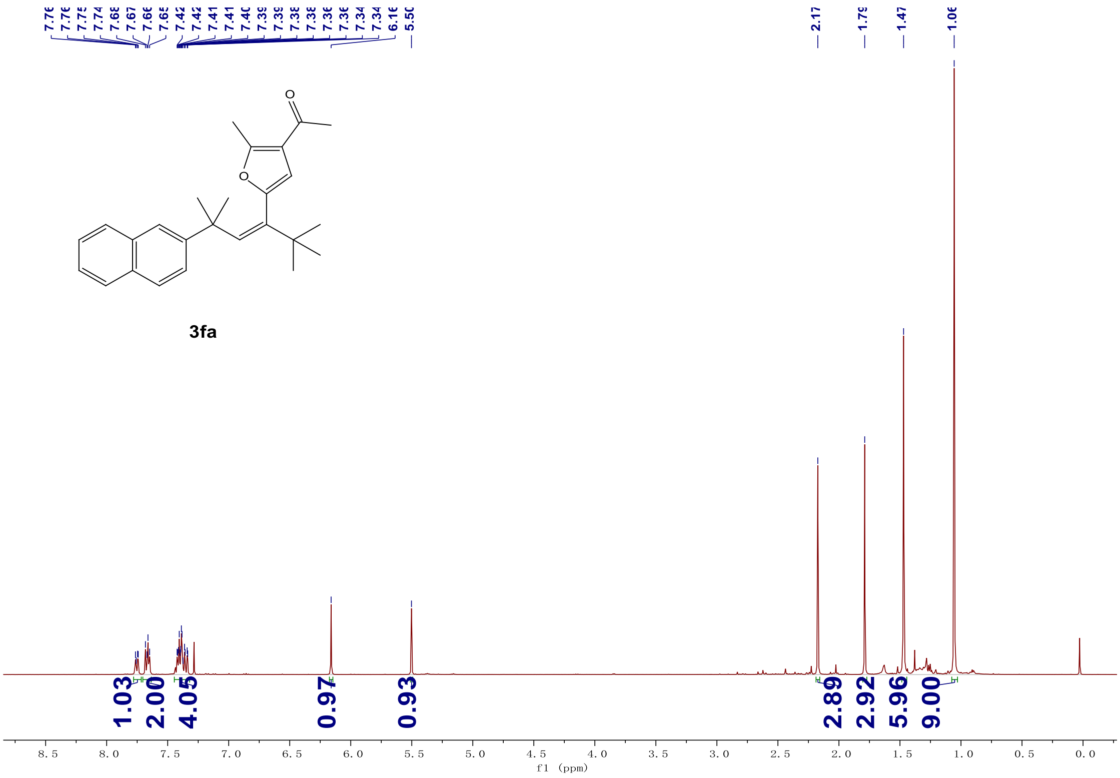

Supplementary Figure 27 ^{1}H NMR (400 MHz, $CDCl_3$) spectrum of compound **3fa**

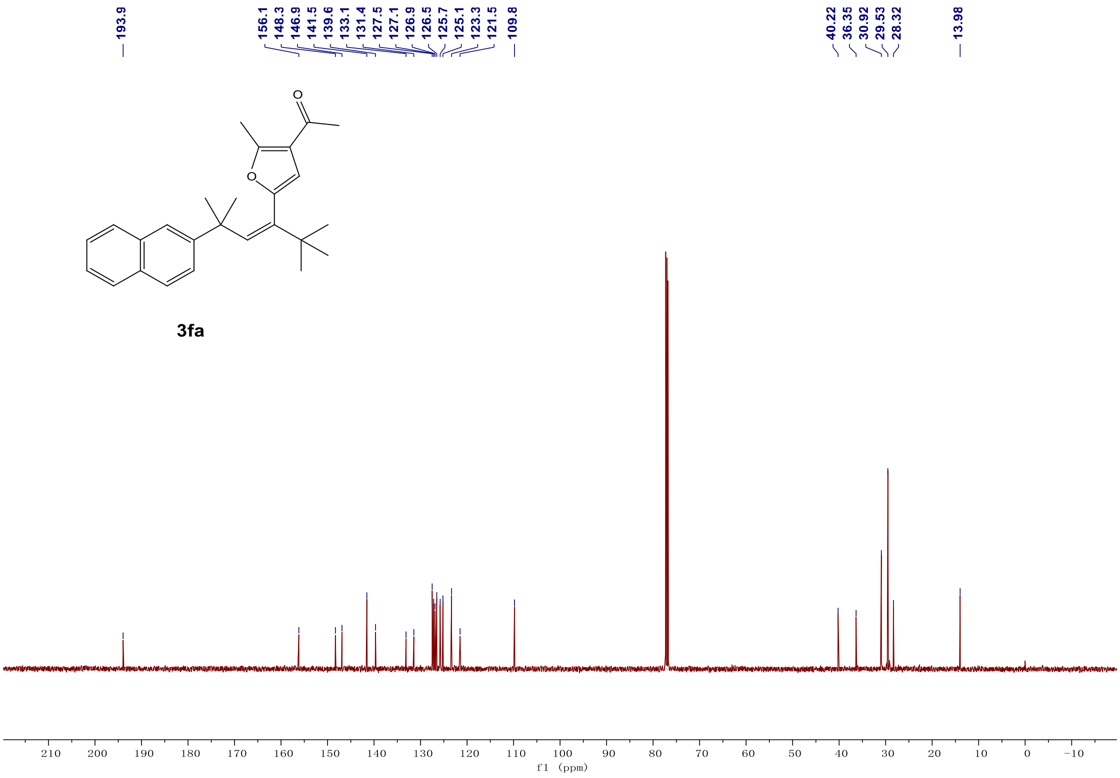

Supplementary Figure 28 ^{13}C NMR (101 MHz, $CDCl_3$) spectrum of compound **3fa**

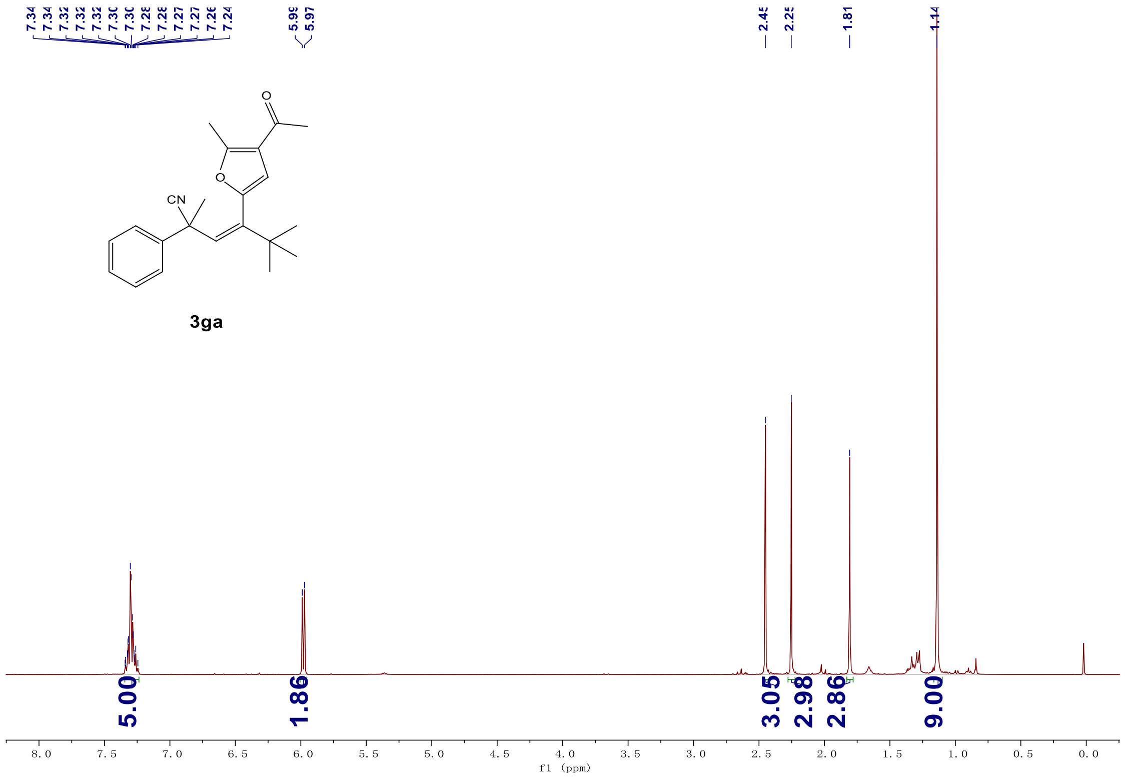

Supplementary Figure 29 ^{1}H NMR (400 MHz, $CDCl_3$) spectrum of compound **3ga**

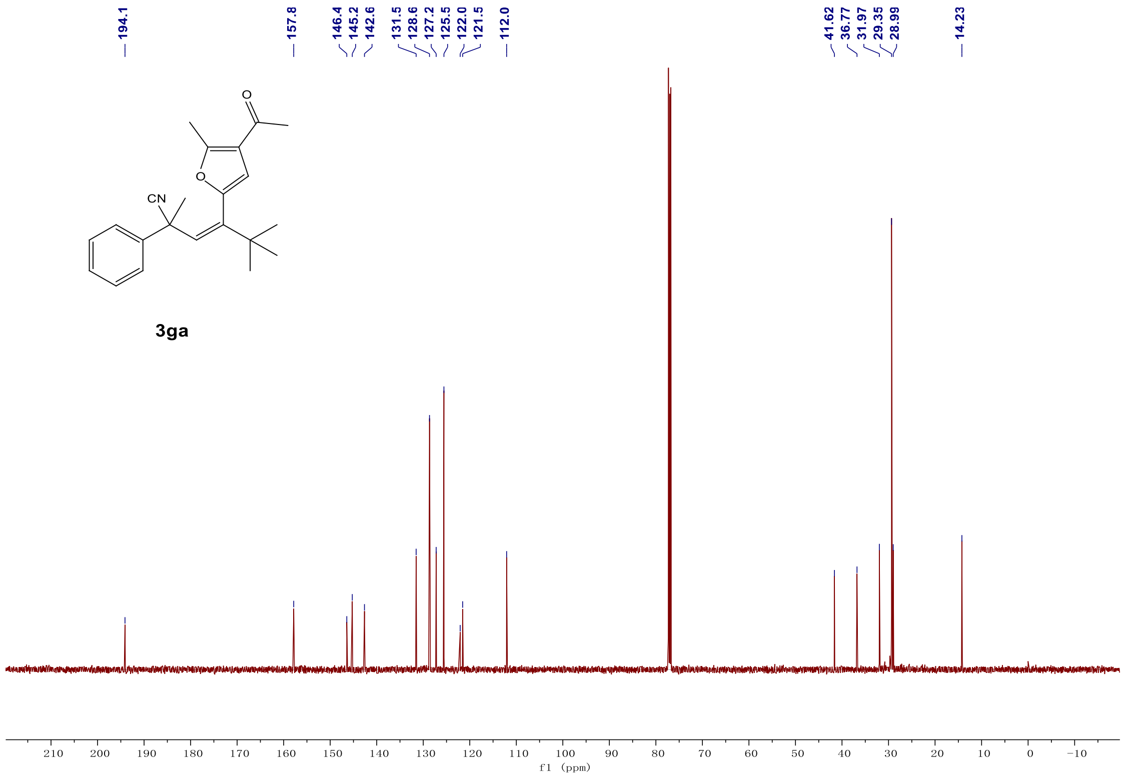

Supplementary Figure 30 ^{13}C NMR (101 MHz, $CDCl_3$) spectrum of compound **3ga**

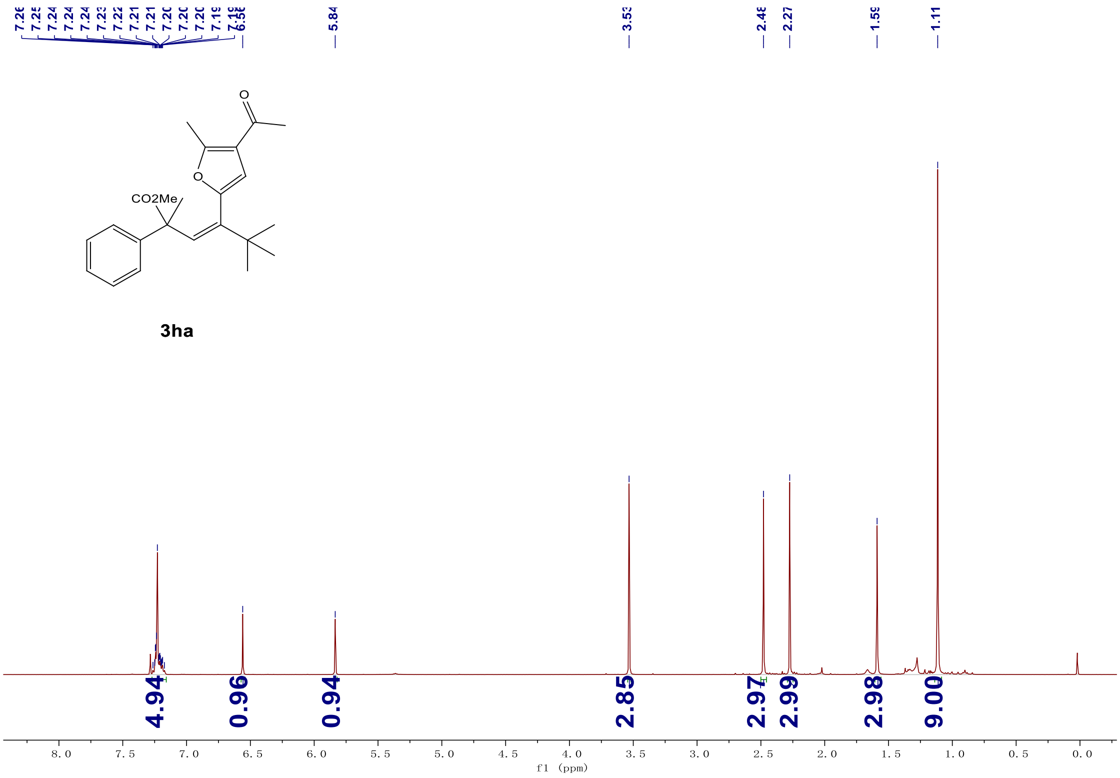

Supplementary Figure 31 ^{1}H NMR (400 MHz, $CDCl_3$) spectrum of compound **3ha**

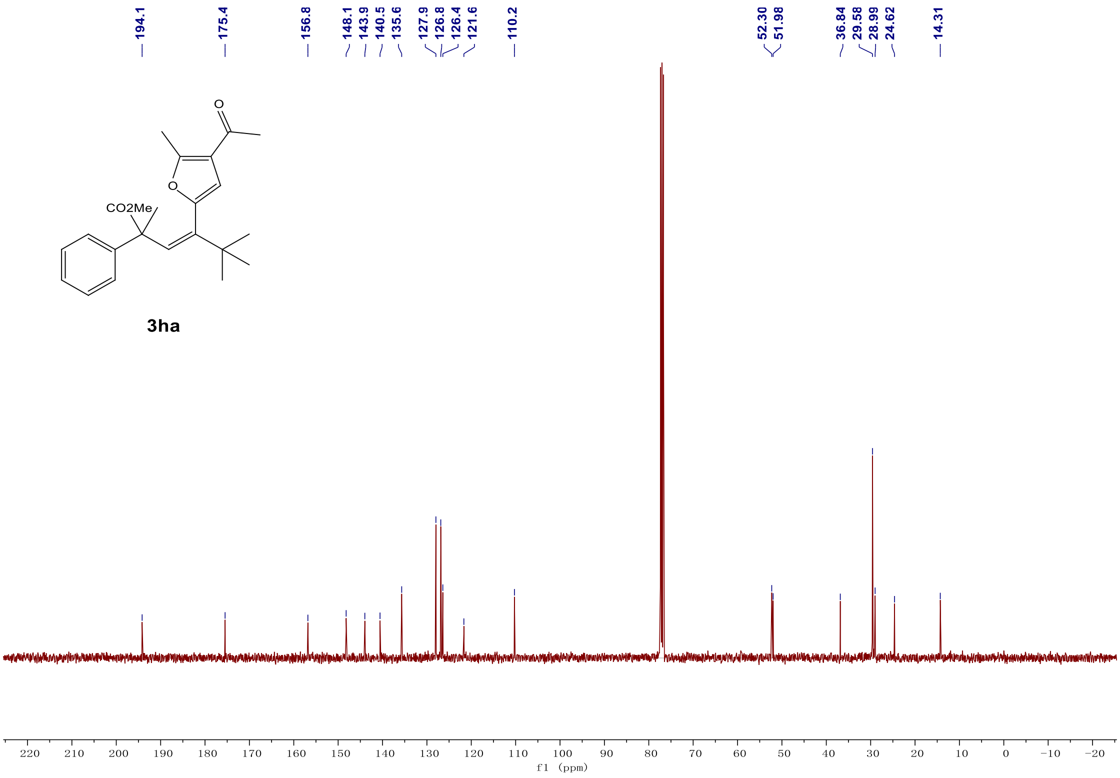

Supplementary Figure 32 ^{13}C NMR (101 MHz, $CDCl_3$) spectrum of compound **3ha**

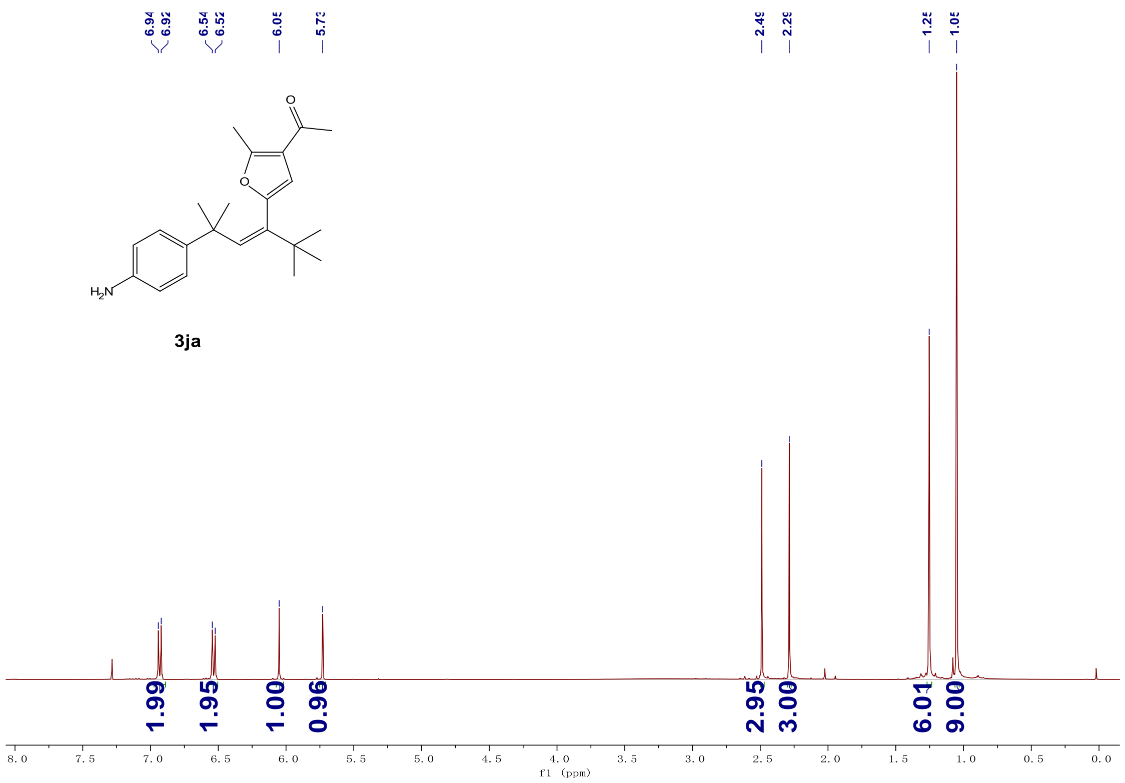

Supplementary Figure 33 ^{1}H NMR (400 MHz, $CDCl_3$) spectrum of compound **3ja**

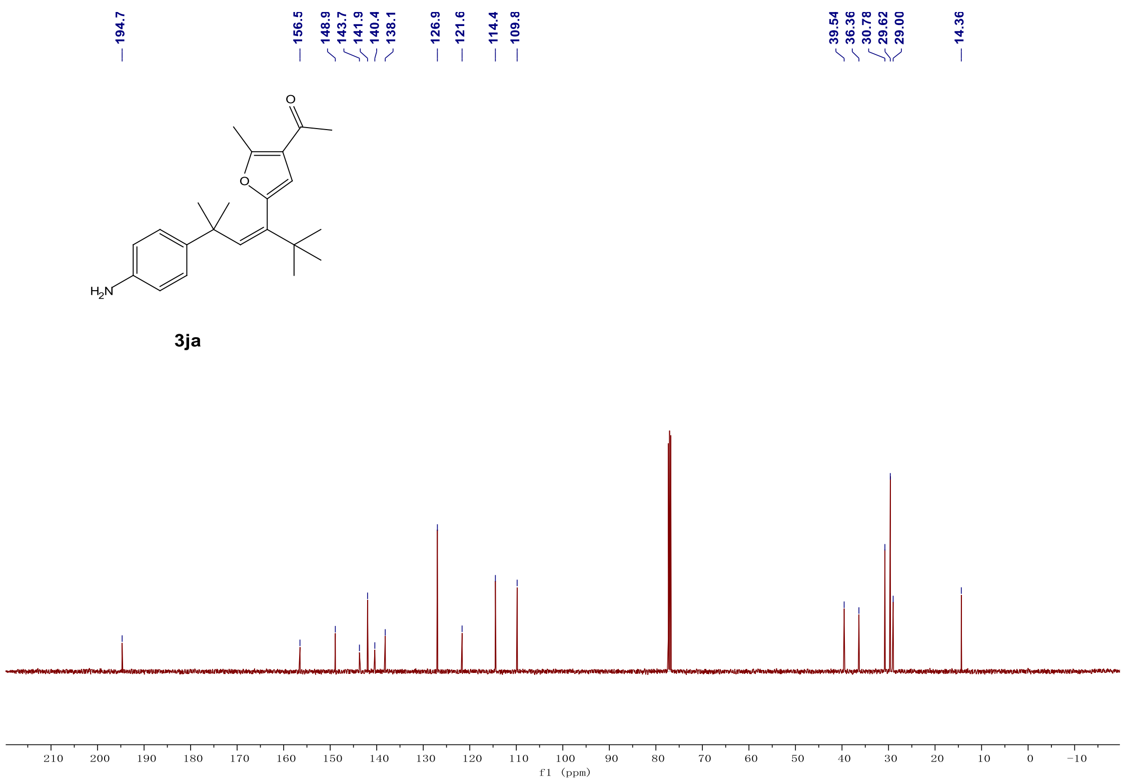

Supplementary Figure 34 ^{13}C NMR (101 MHz, $CDCl_3$) spectrum of compound **3ja**

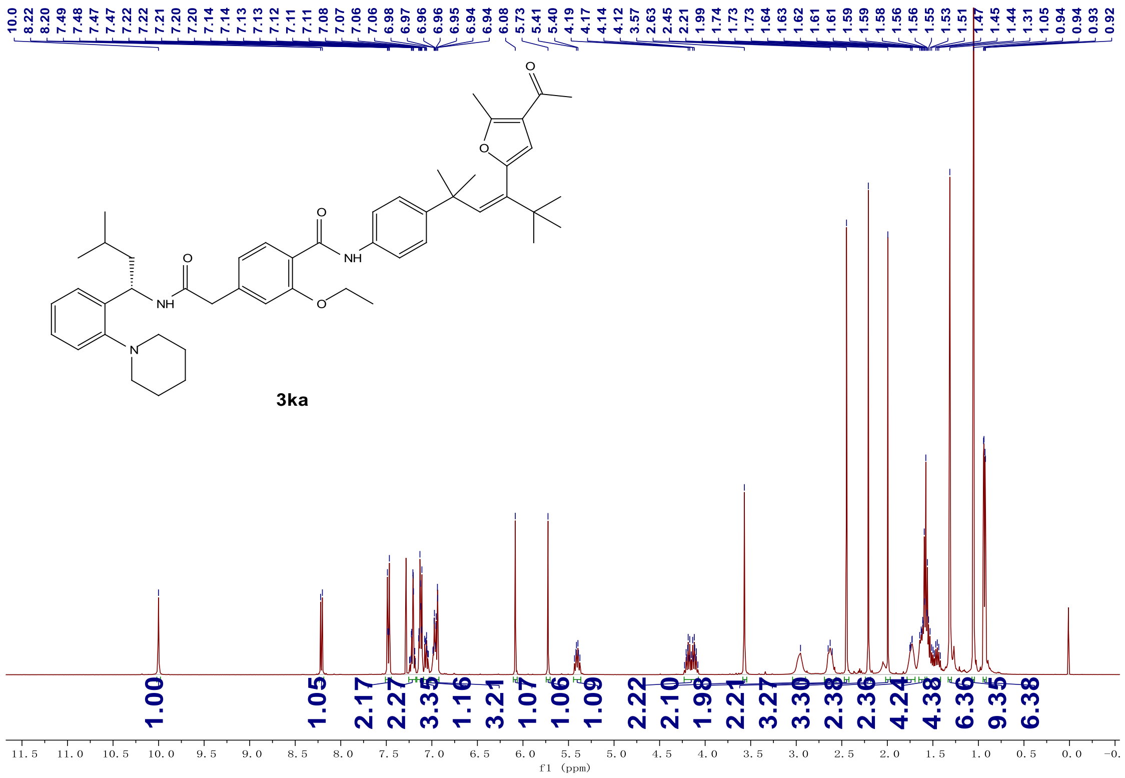

Supplementary Figure 35 ^{1}H NMR (400 MHz, $CDCl_3$) spectrum of compound **3ka**

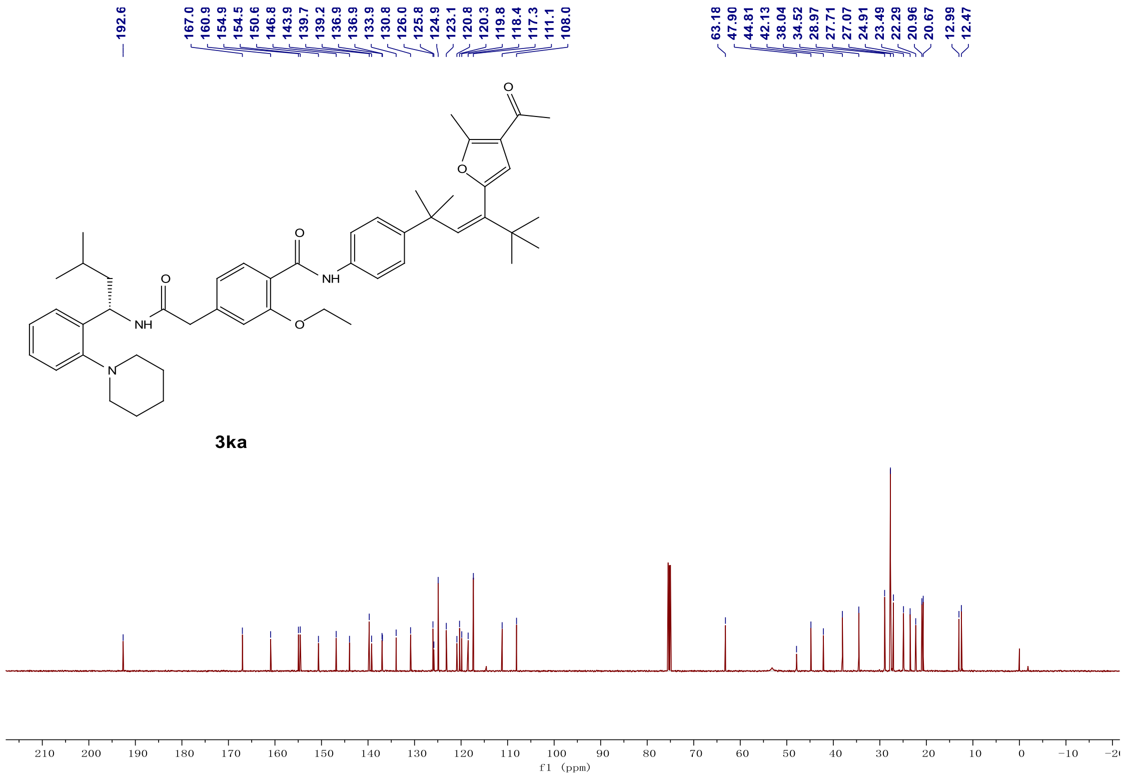

Supplementary Figure 36 ^{13}C NMR (101 MHz, $CDCl_3$) spectrum of compound **3ka**

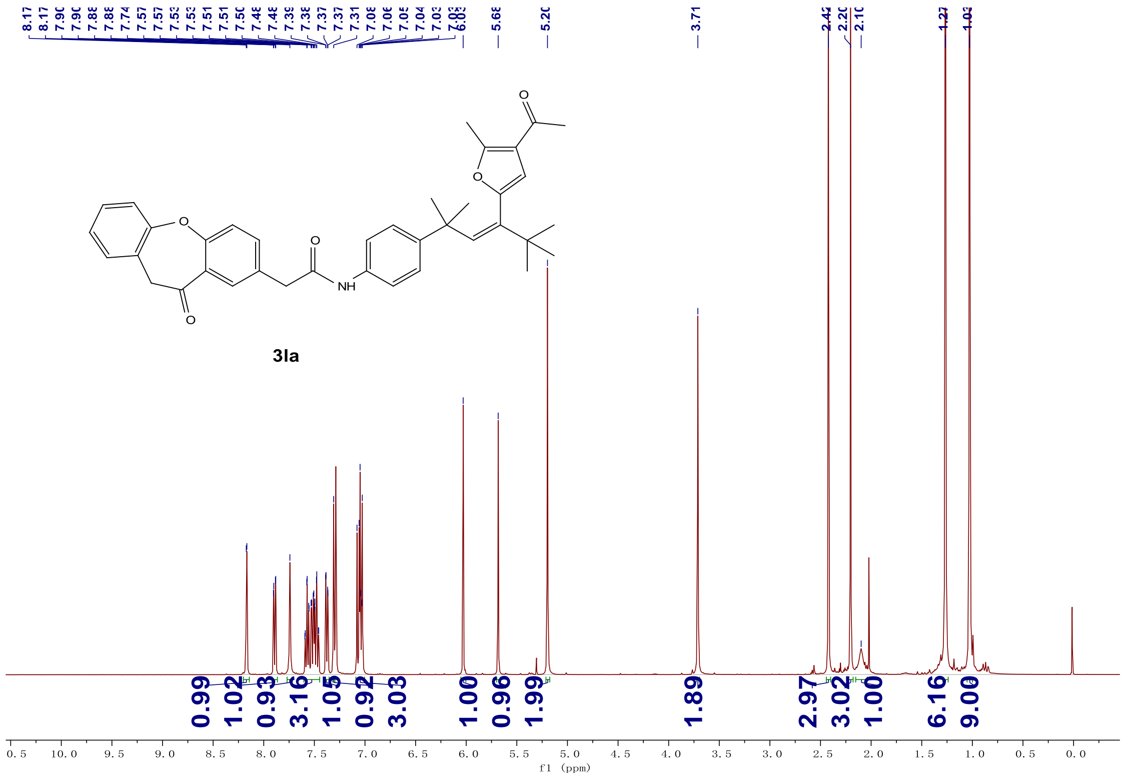

Supplementary Figure 37 ^{1}H NMR (400 MHz, $CDCl_3$) spectrum of compound **3la**

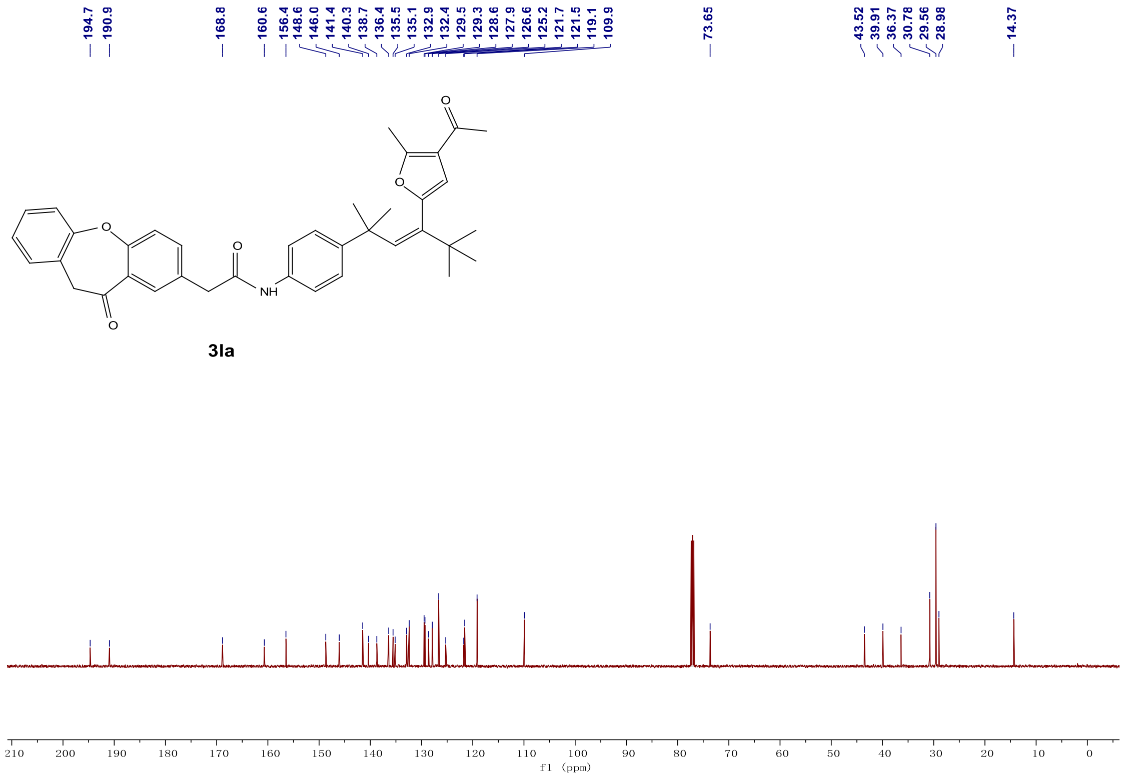

Supplementary Figure 38 ^{13}C NMR (101 MHz, $CDCl_3$) spectrum of compound **3la**

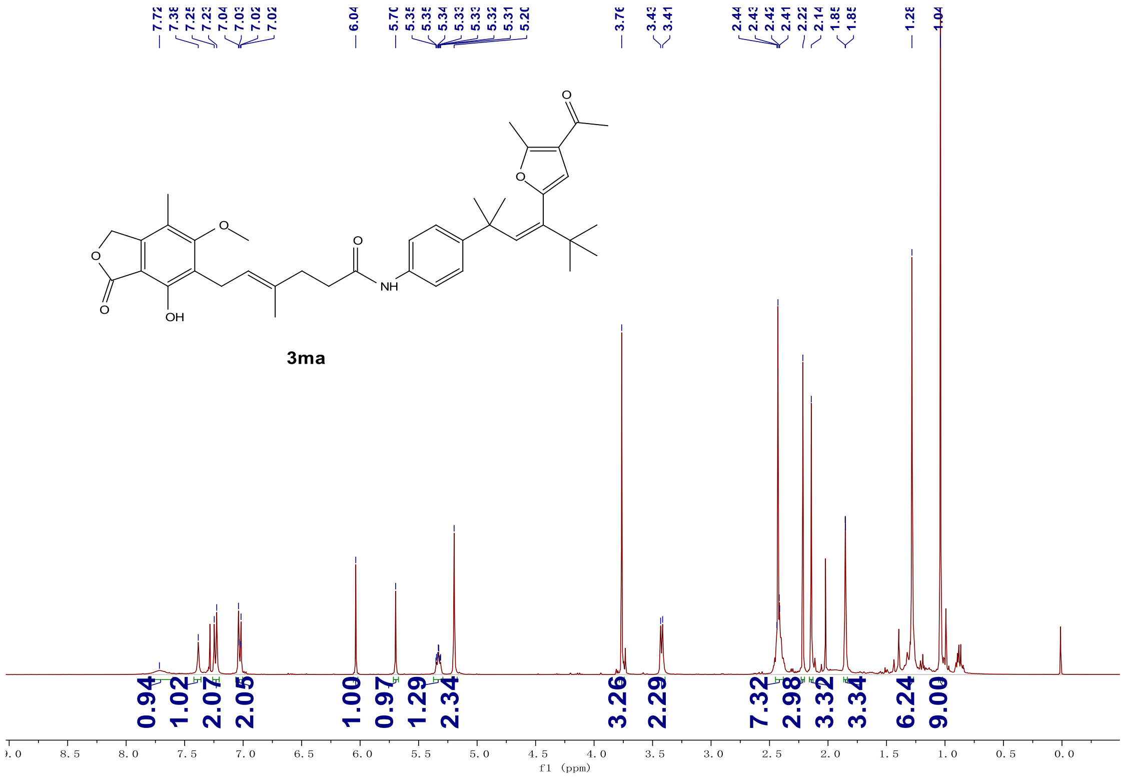

Supplementary Figure 39 ^{1}H NMR (400 MHz, $CDCl_3$) spectrum of compound **3ma**

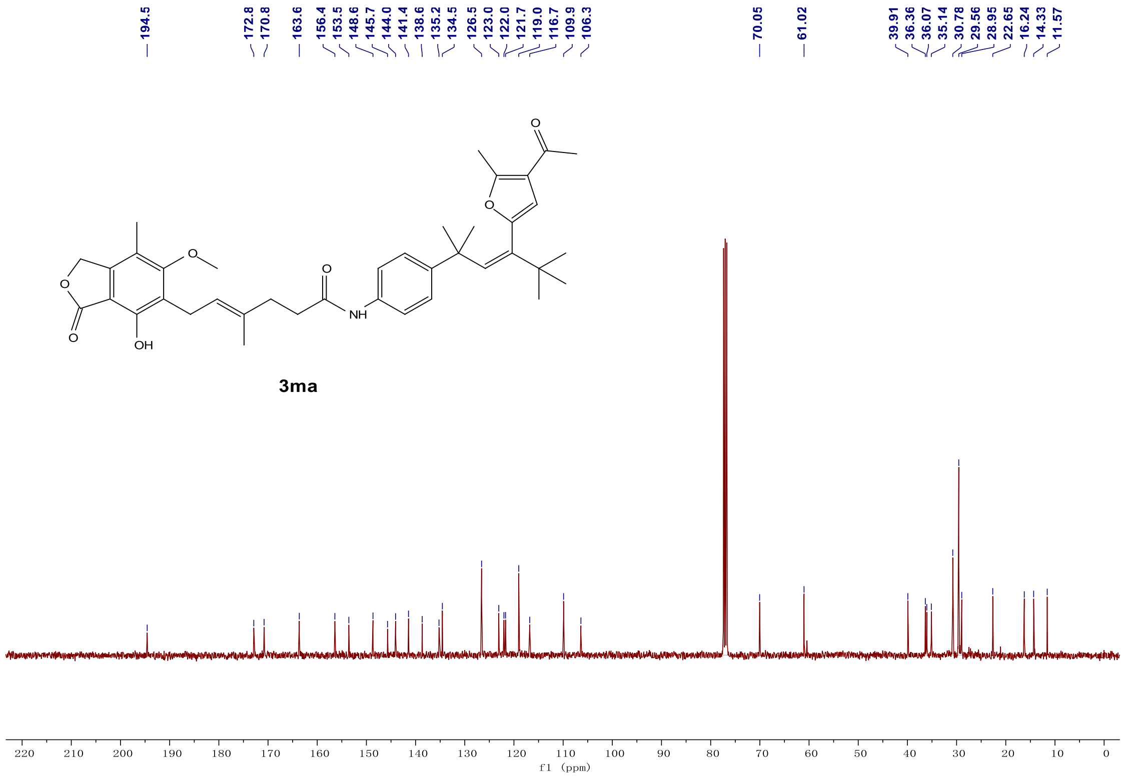

Supplementary Figure 40 ^{13}C NMR (101 MHz, $CDCl_3$) spectrum of compound **3ma**

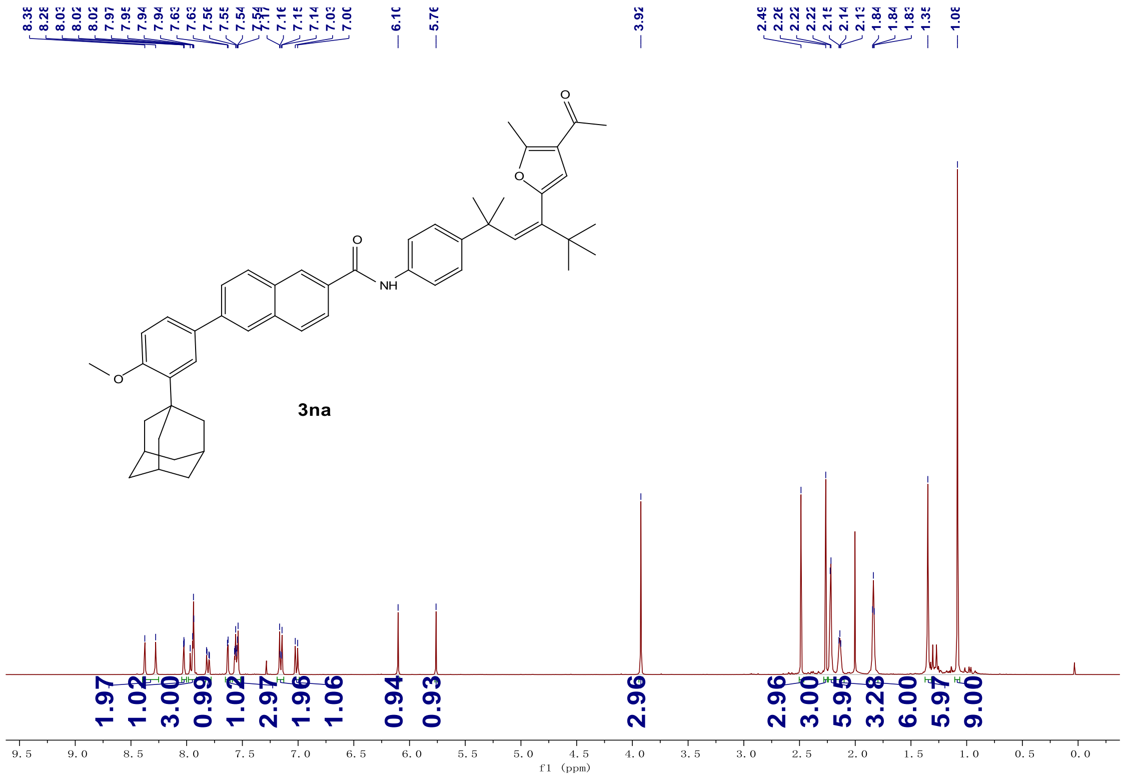

Supplementary Figure 41 ^{1}H NMR (400 MHz, $CDCl_3$) spectrum of compound **3na**

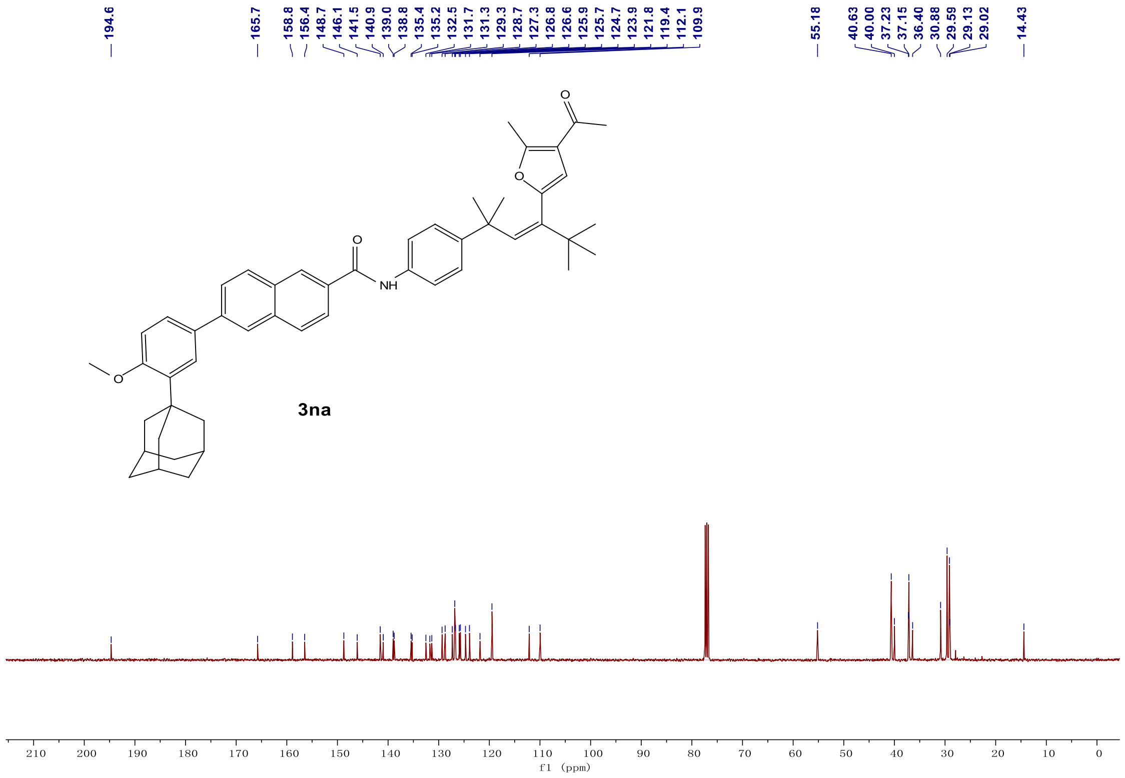

Supplementary Figure 42 ^{13}C NMR (101 MHz, $CDCl_3$) spectrum of compound **3na**

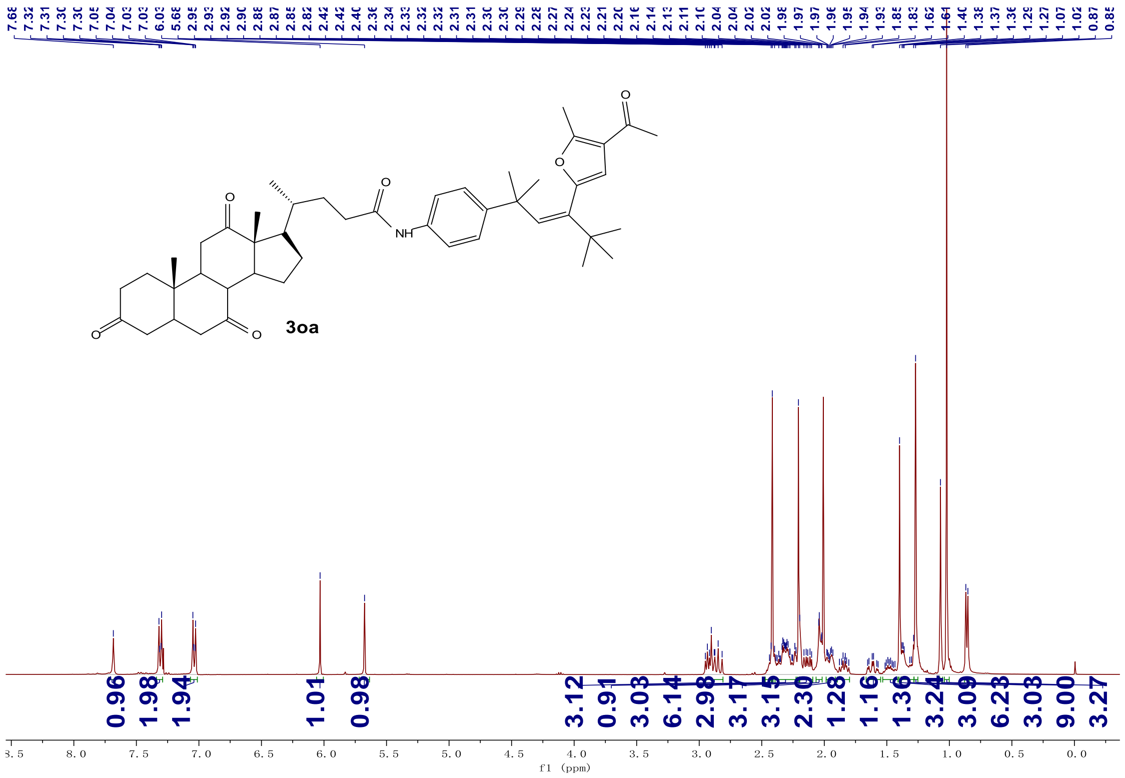

Supplementary Figure 43 ^{1}H NMR (400 MHz, $CDCl_3$) spectrum of compound **3oa**

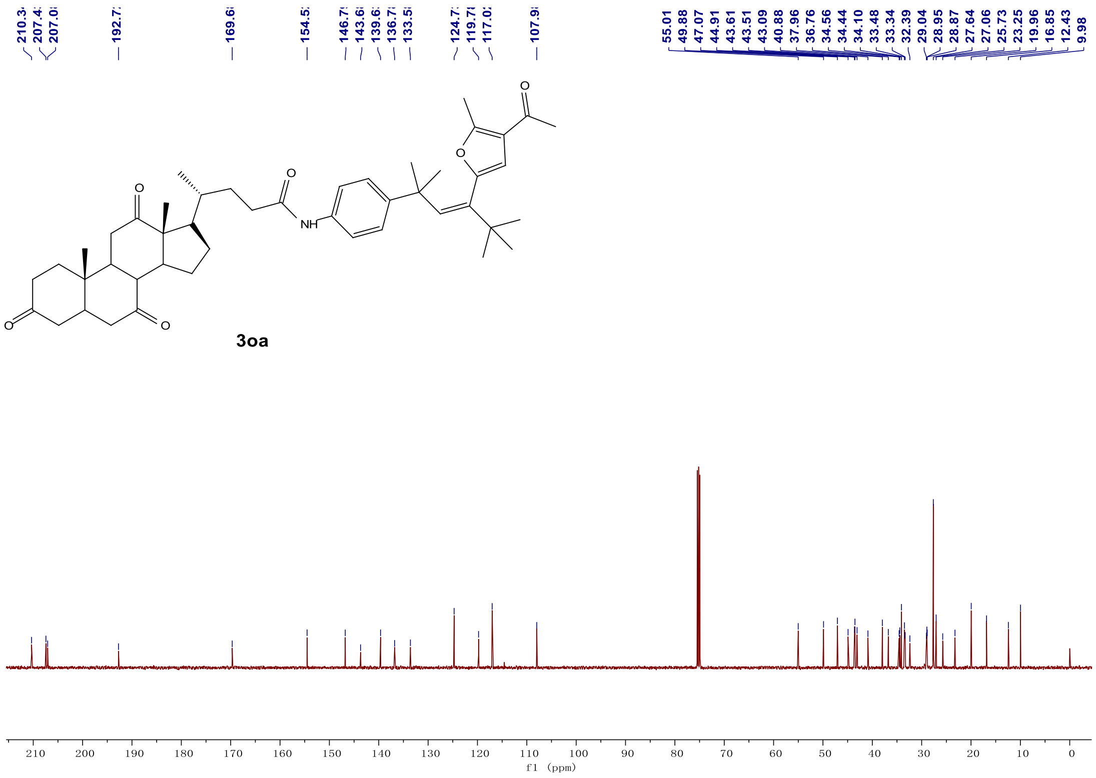

Supplementary Figure 44 ^{13}C NMR (101 MHz, $CDCl_3$) spectrum of compound **3oa**

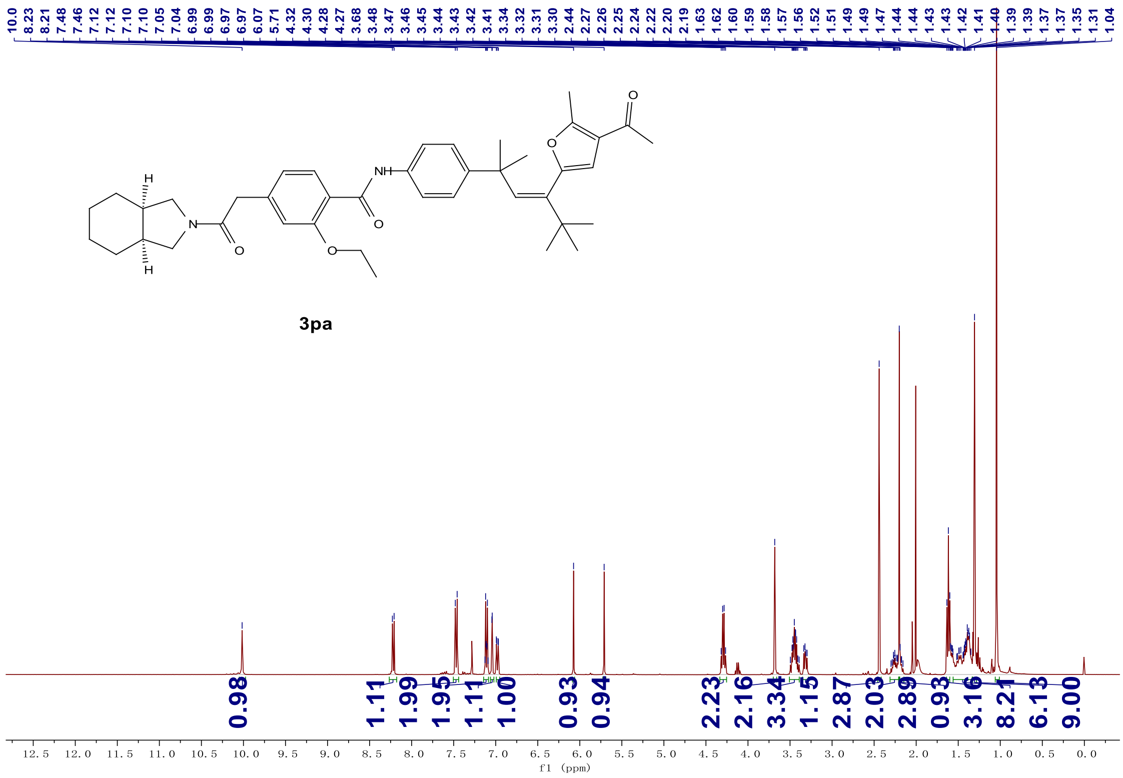

Supplementary Figure 45 ^{1}H NMR (400 MHz, $CDCl_3$) spectrum of compound **3pa**

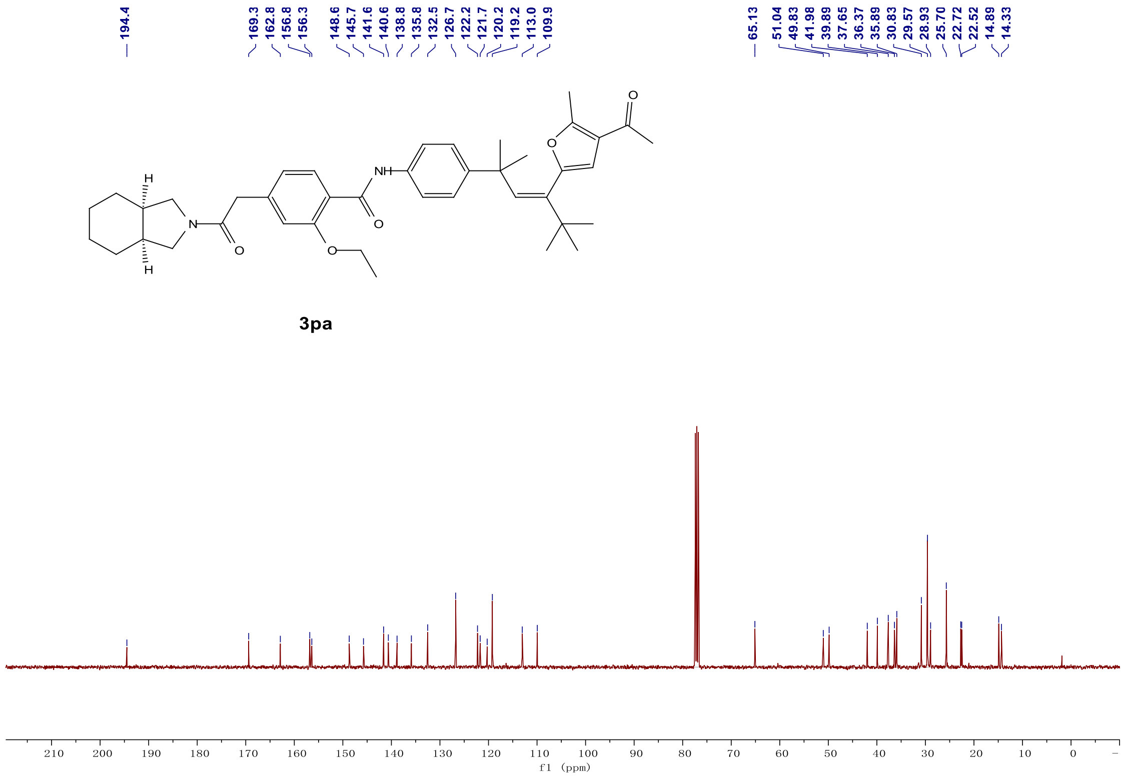

Supplementary Figure 46 ^{13}C NMR (101 MHz, $CDCl_3$) spectrum of compound **3pa**

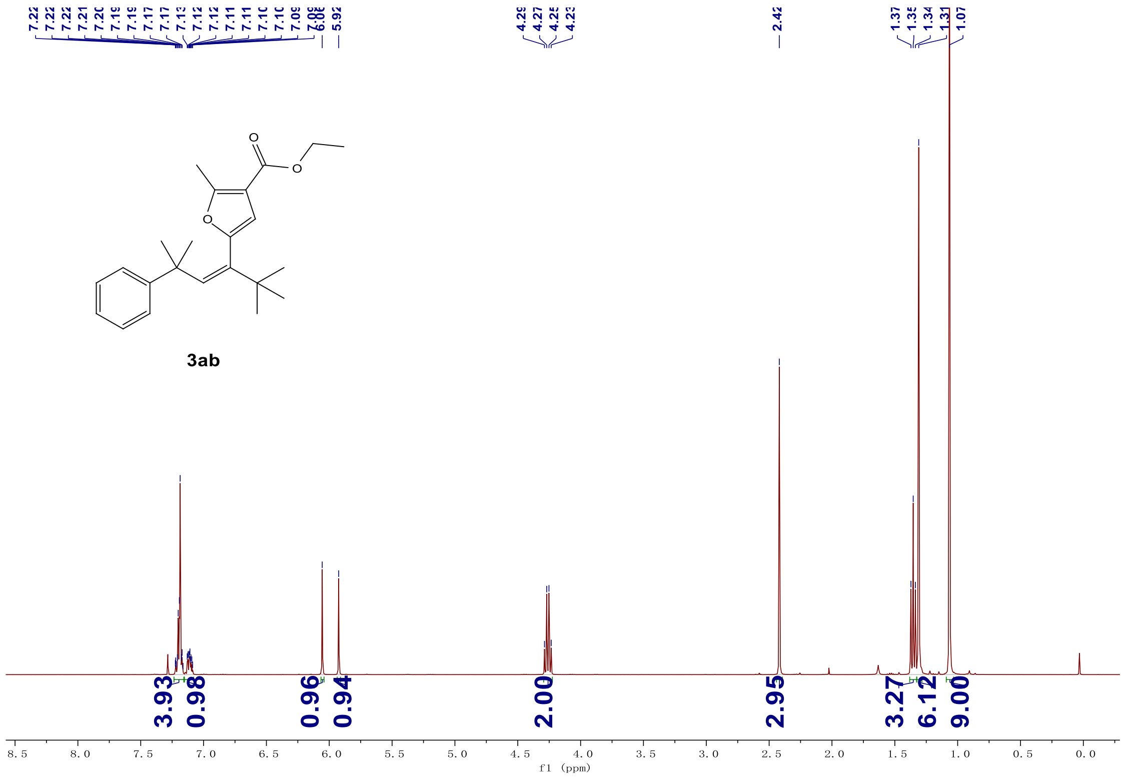

Supplementary Figure 47 ^{1}H NMR (400 MHz, $CDCl_3$) spectrum of compound **3ab**

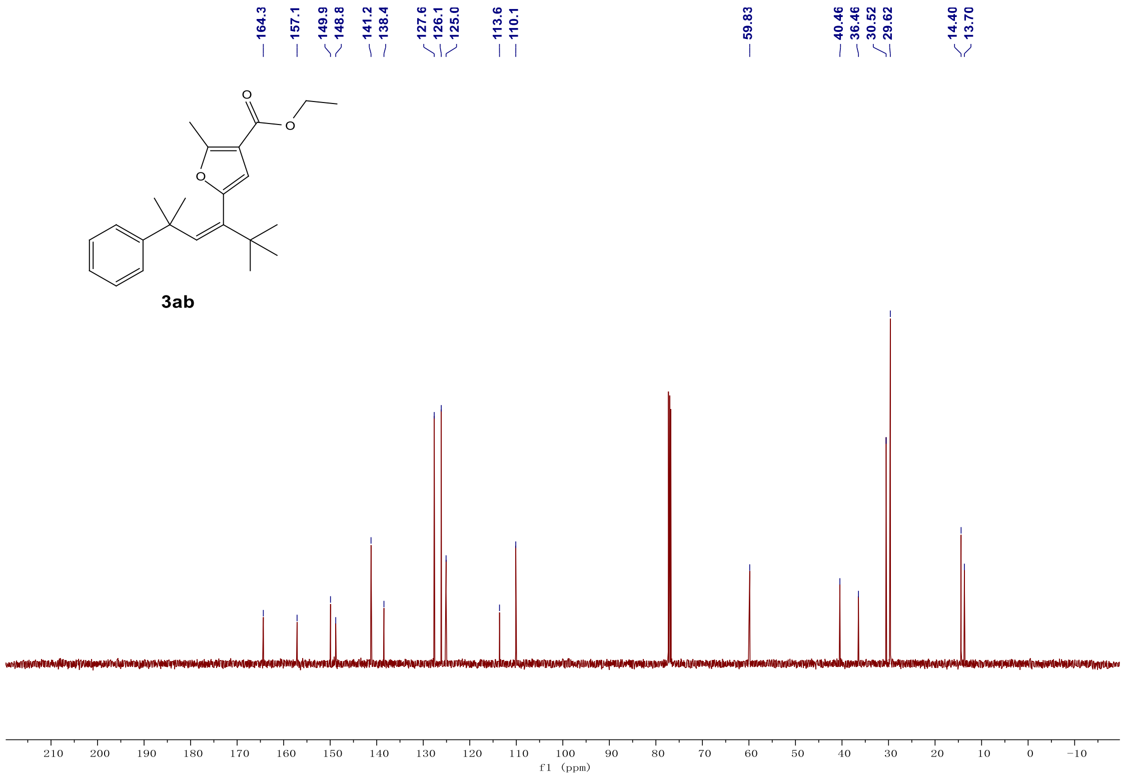

Supplementary Figure 48 ^{13}C NMR (101 MHz, $CDCl_3$) spectrum of compound **3ab**

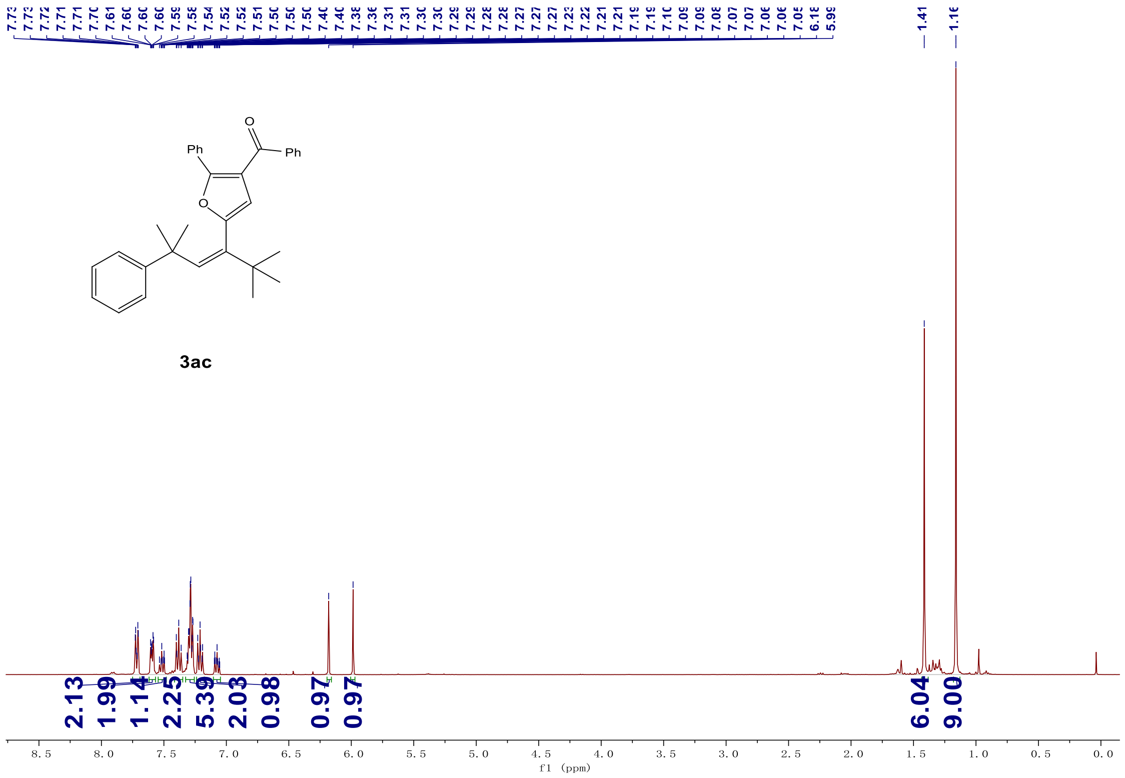

Supplementary Figure 49 ^{1}H NMR (400 MHz, $CDCl_3$) spectrum of compound **3ac**

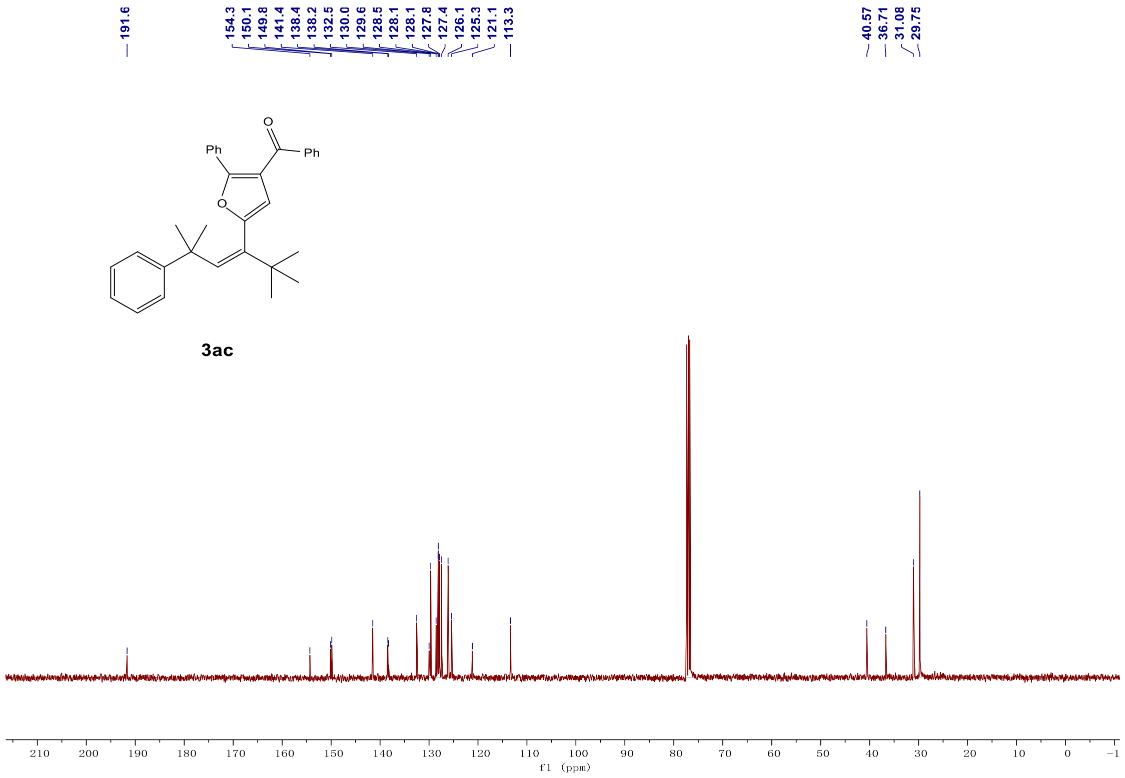

Supplementary Figure 50 ^{13}C NMR (101 MHz, $CDCl_3$) spectrum of compound **3ac**

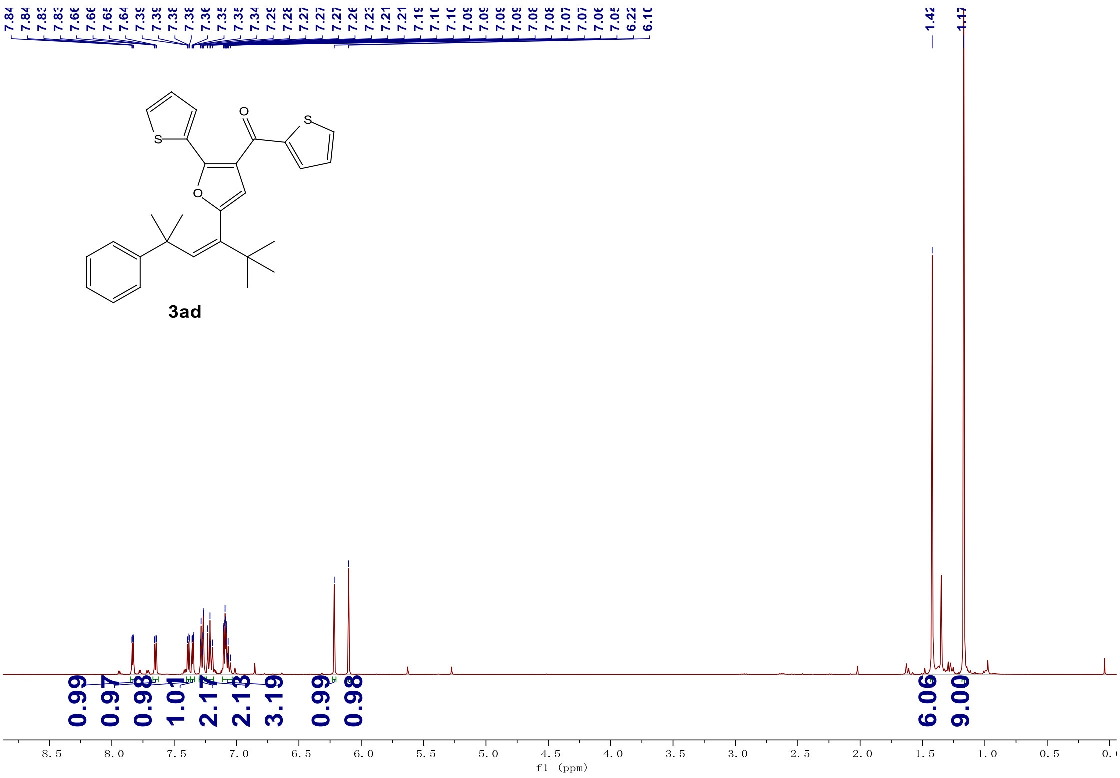

Supplementary Figure 51 ^{1}H NMR (400 MHz, $CDCl_3$) spectrum of compound **3ad**

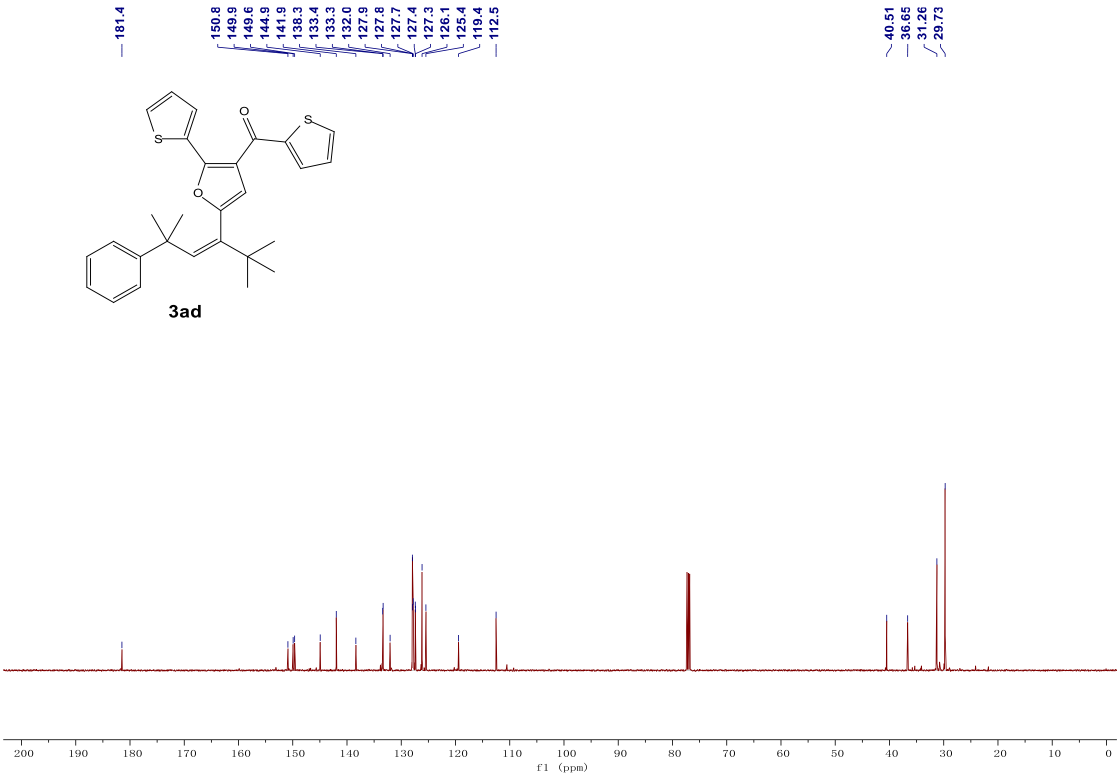

Supplementary Figure 52 ^{13}C NMR (101 MHz, $CDCl_3$) spectrum of compound **3ad**

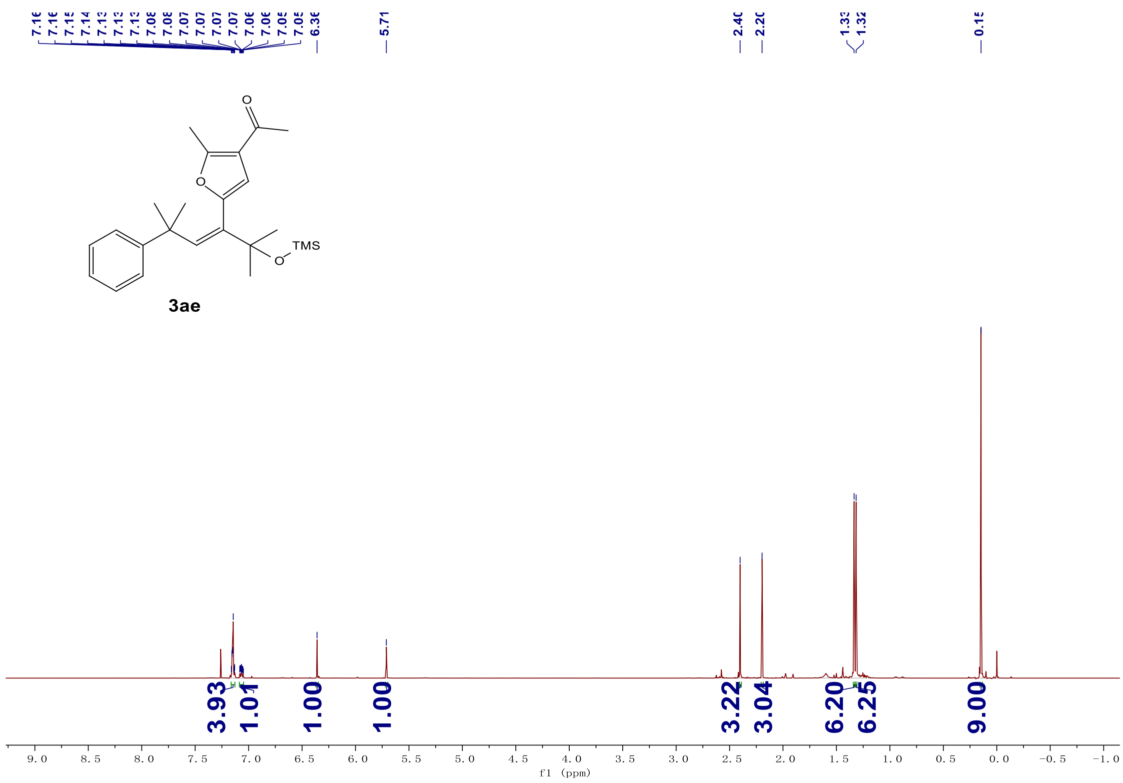

Supplementary Figure 53 ^{1}H NMR (400 MHz, $CDCl_3$) spectrum of compound **3ae**

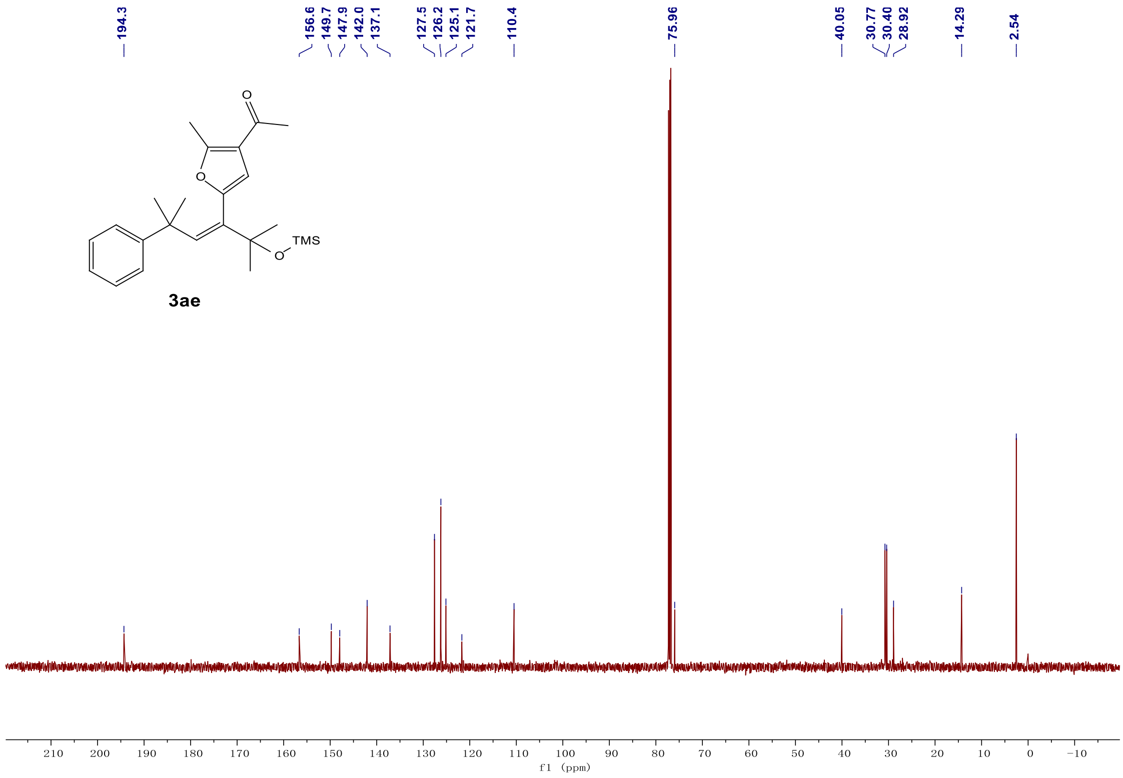

Supplementary Figure 54 ^{13}C NMR (101 MHz, $CDCl_3$) spectrum of compound **3ae**

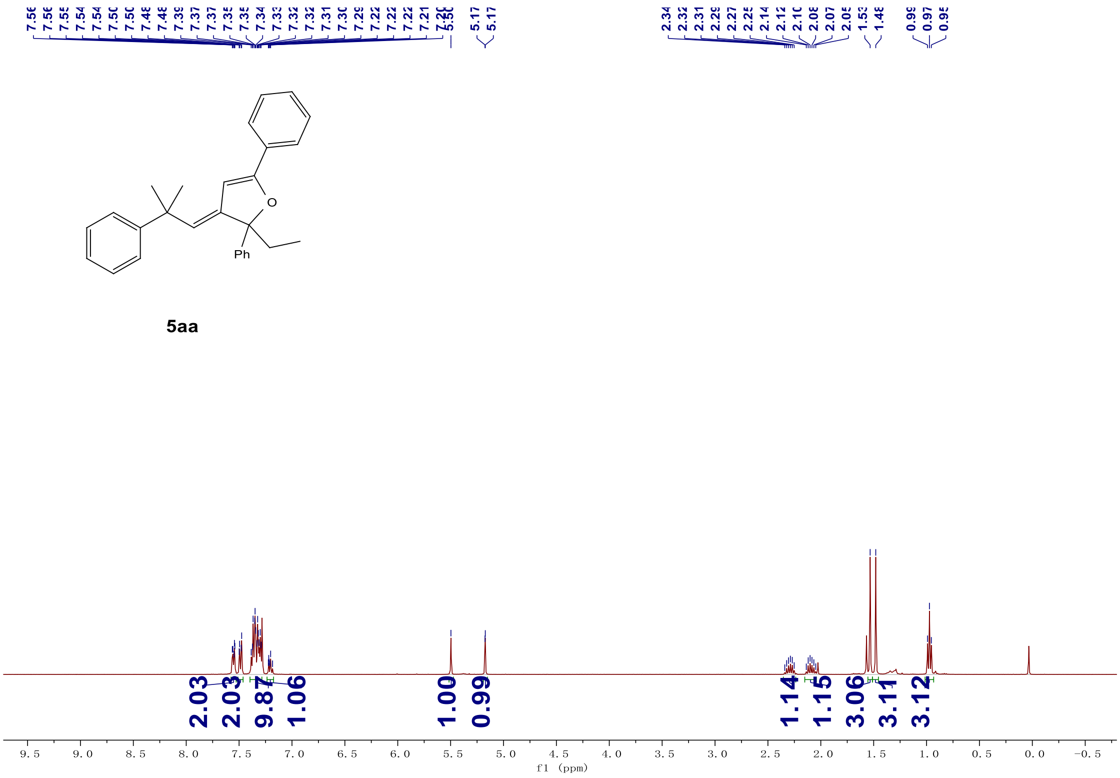

Supplementary Figure 55 ^{1}H NMR (400 MHz, $CDCl_3$) spectrum of compound **5aa**

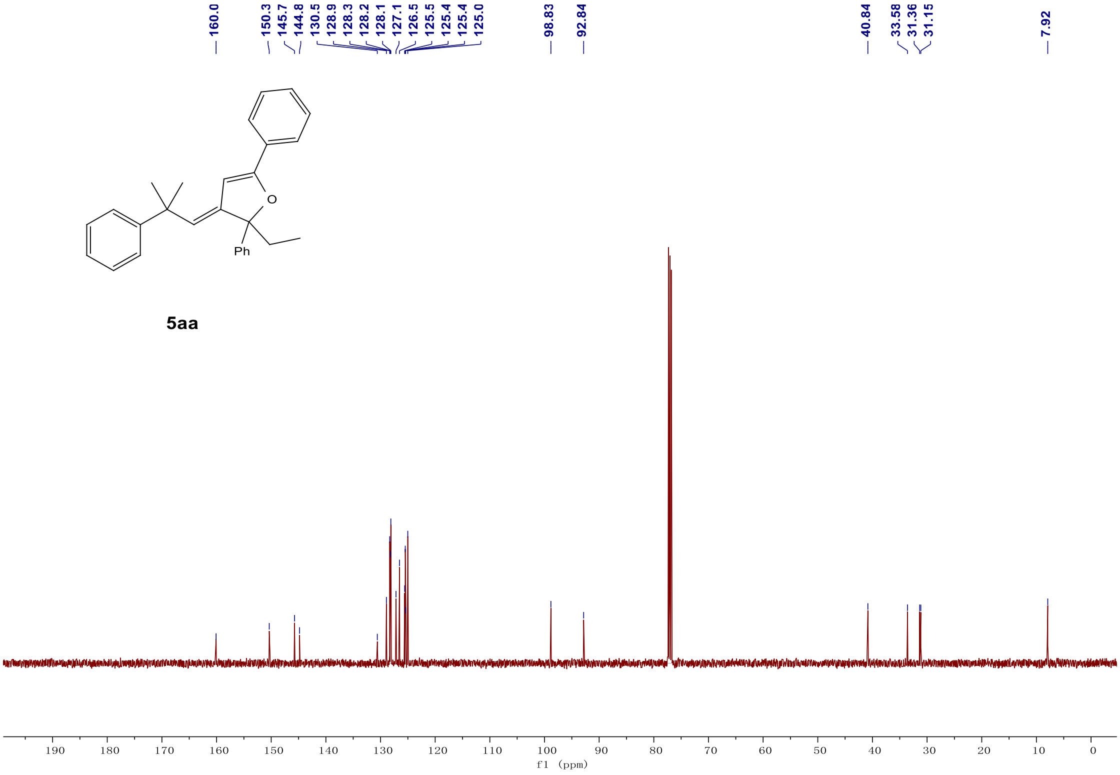

Supplementary Figure 56 ^{13}C NMR (101 MHz, $CDCl_3$) spectrum of compound **5aa**

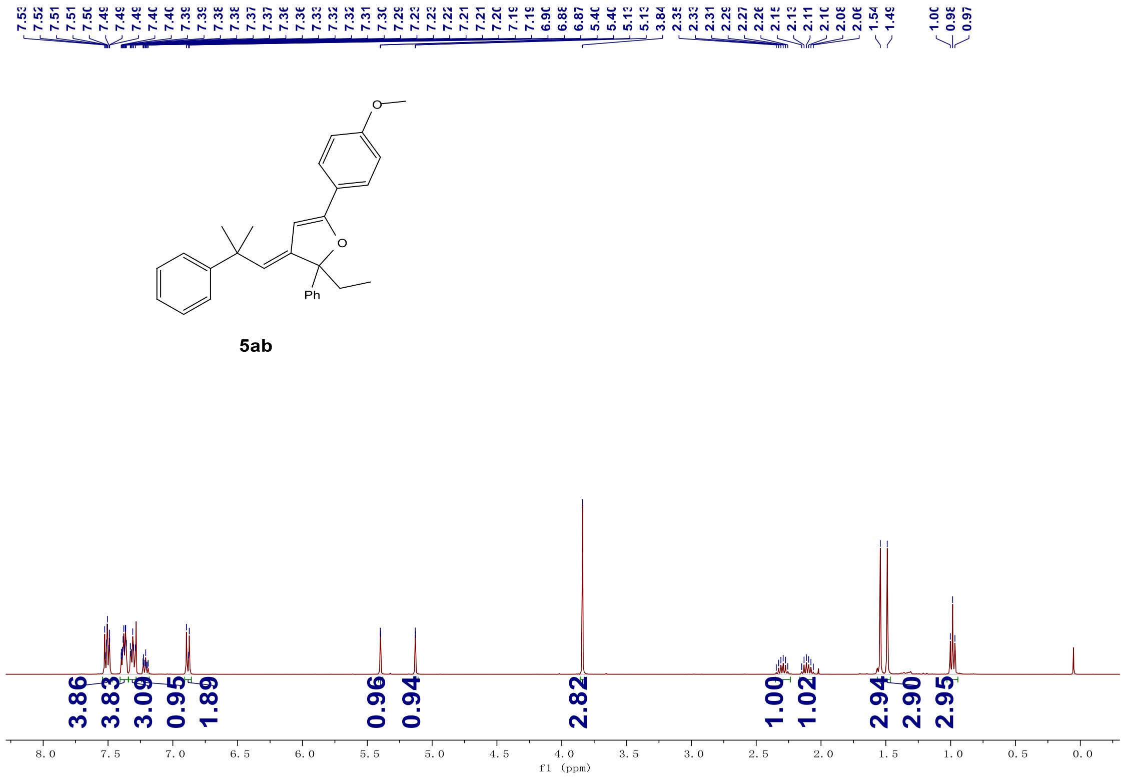

Supplementary Figure 57 ^{1}H NMR (400 MHz, $CDCl_3$) spectrum of compound **5ab**

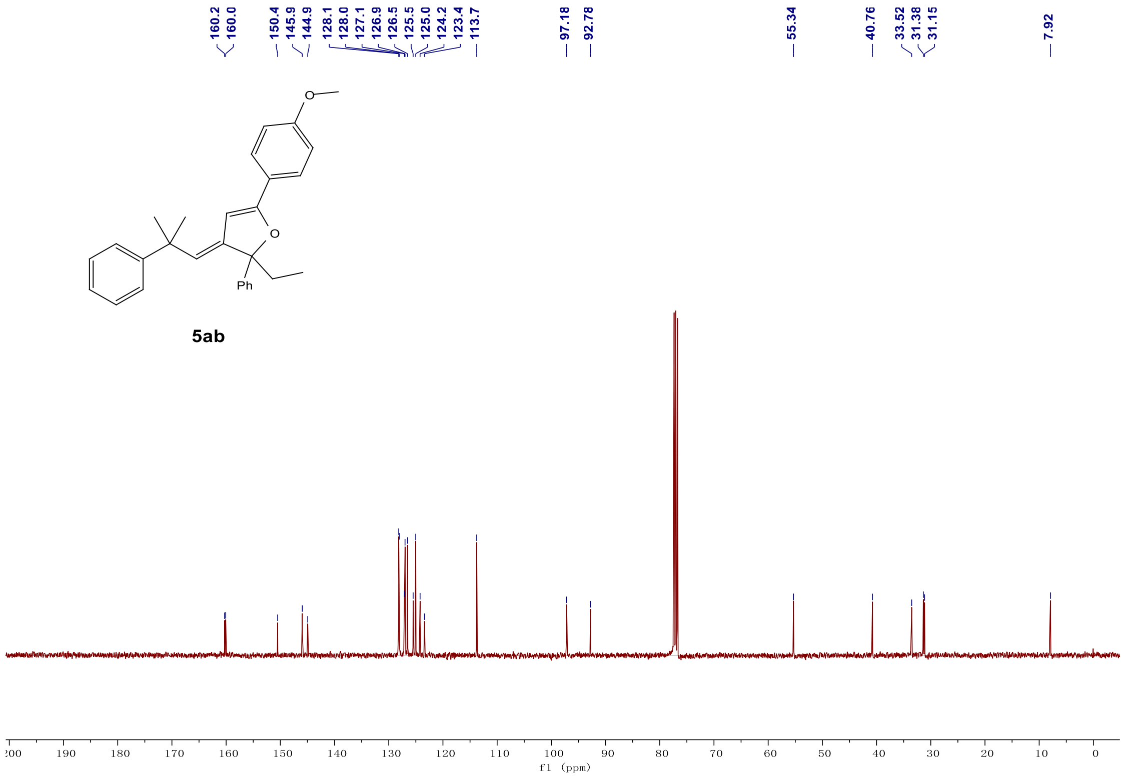

Supplementary Figure 58 ^{13}C NMR (101 MHz, $CDCl_3$) spectrum of compound **5ab**

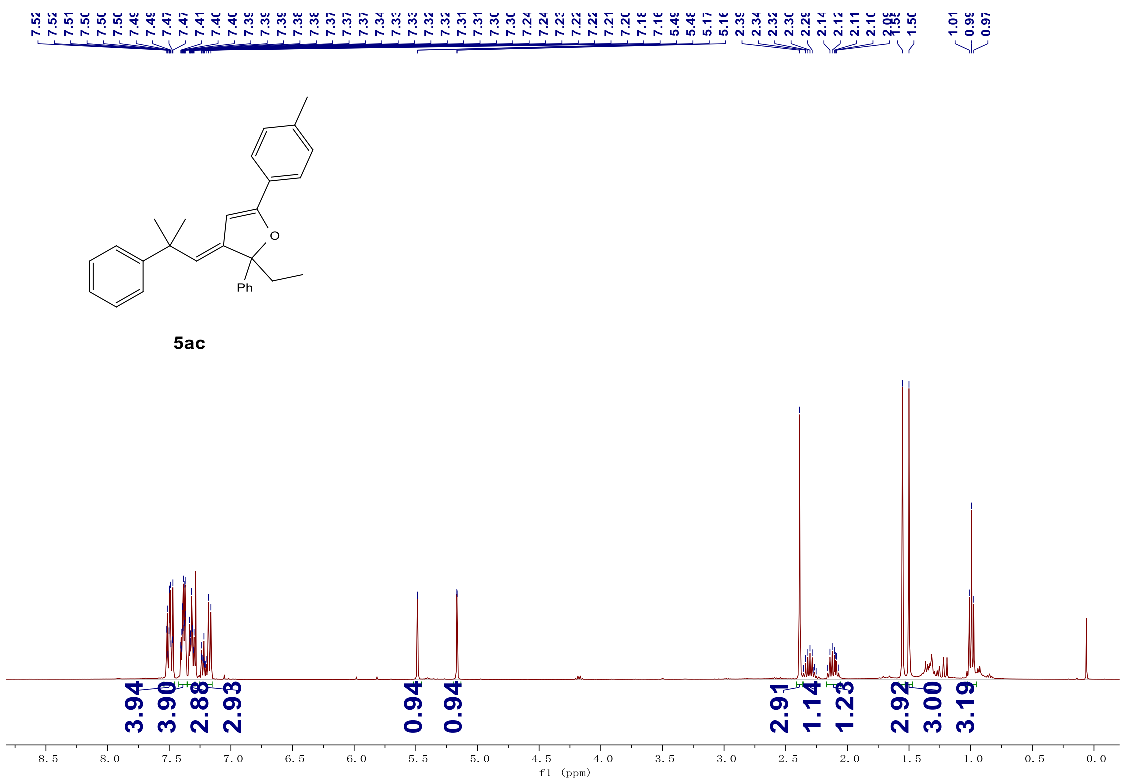

Supplementary Figure 59 ^{1}H NMR (400 MHz, $CDCl_3$) spectrum of compound **5ac**

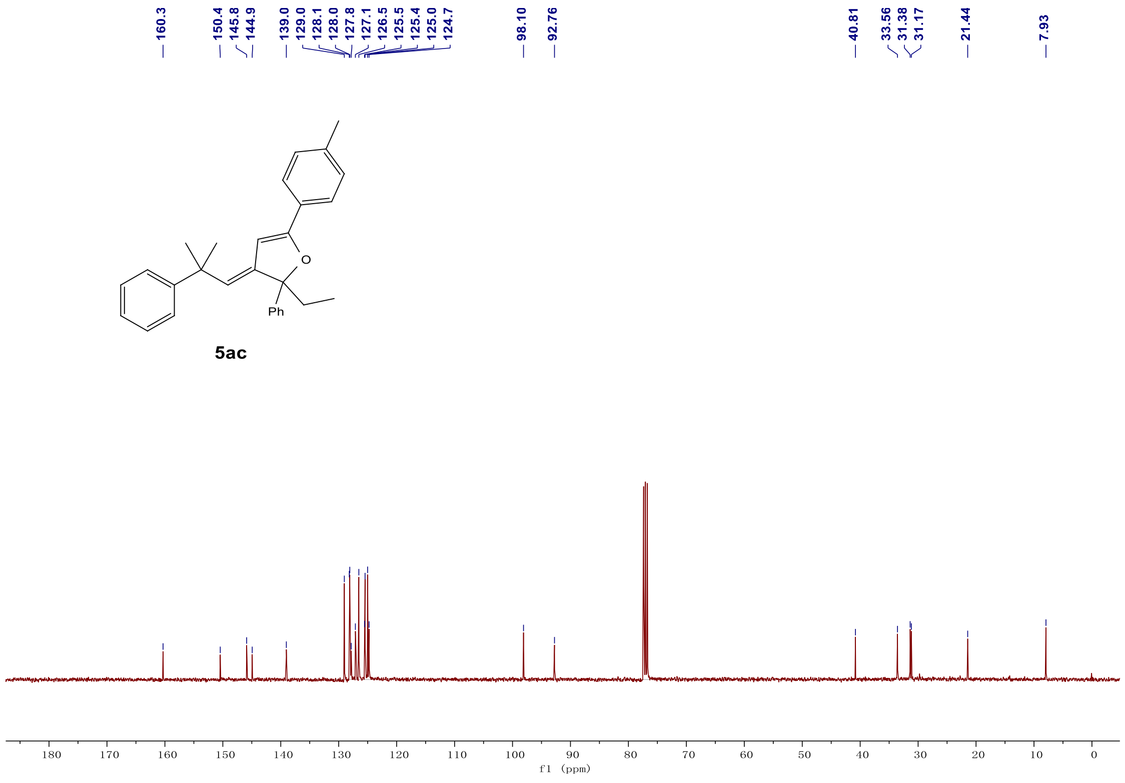

Supplementary Figure 60 ^{13}C NMR (101 MHz, $CDCl_3$) spectrum of compound **5ac**

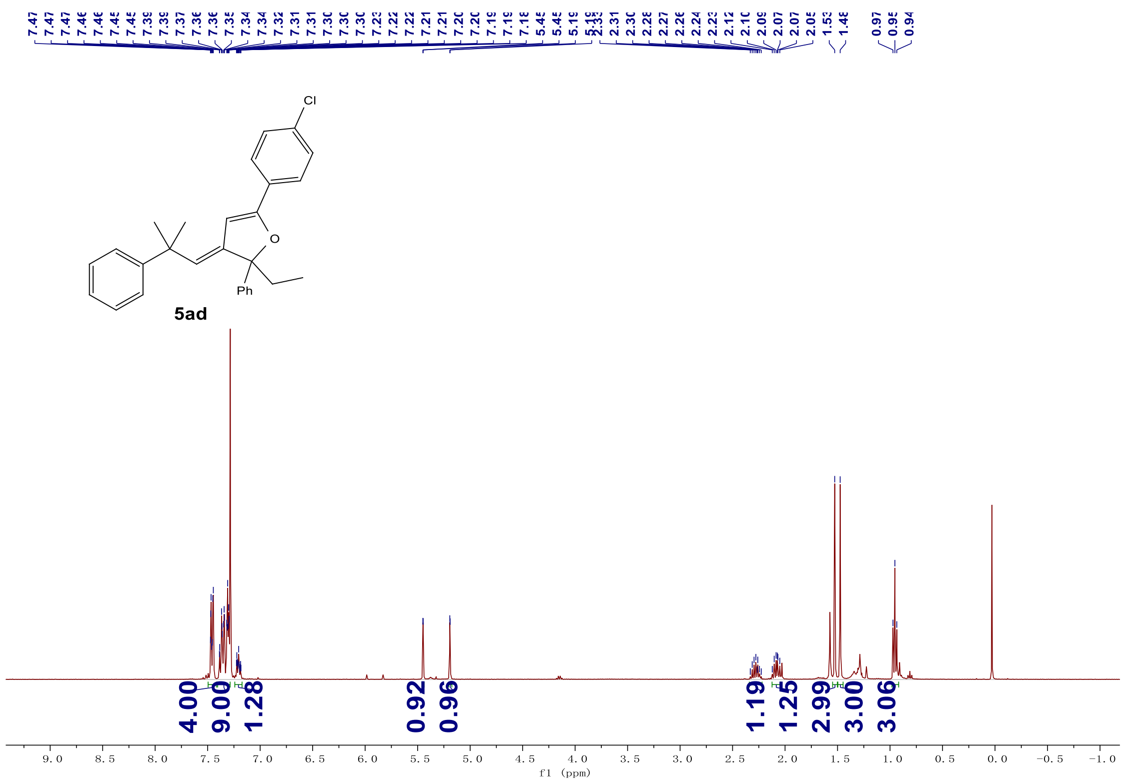

Supplementary Figure 61 ^{1}H NMR (400 MHz, $CDCl_3$) spectrum of compound **5ad**

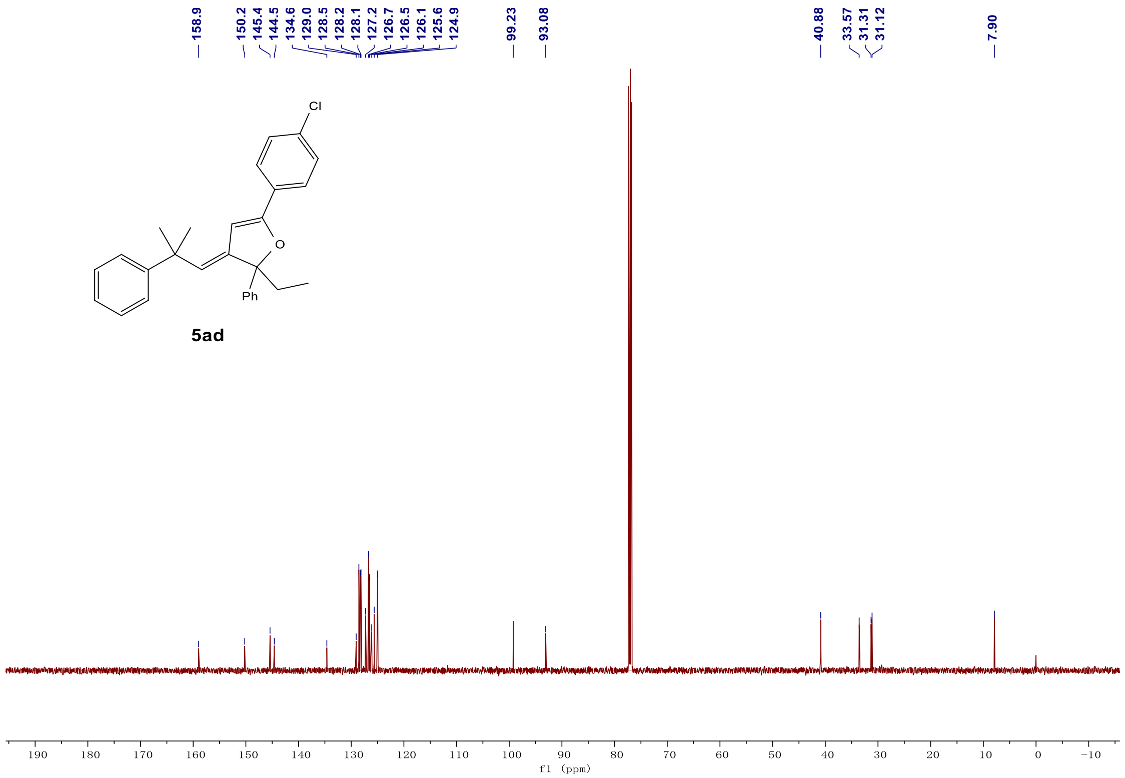

Supplementary Figure 62 ^{13}C NMR (101 MHz, $CDCl_3$) spectrum of compound **5ad**

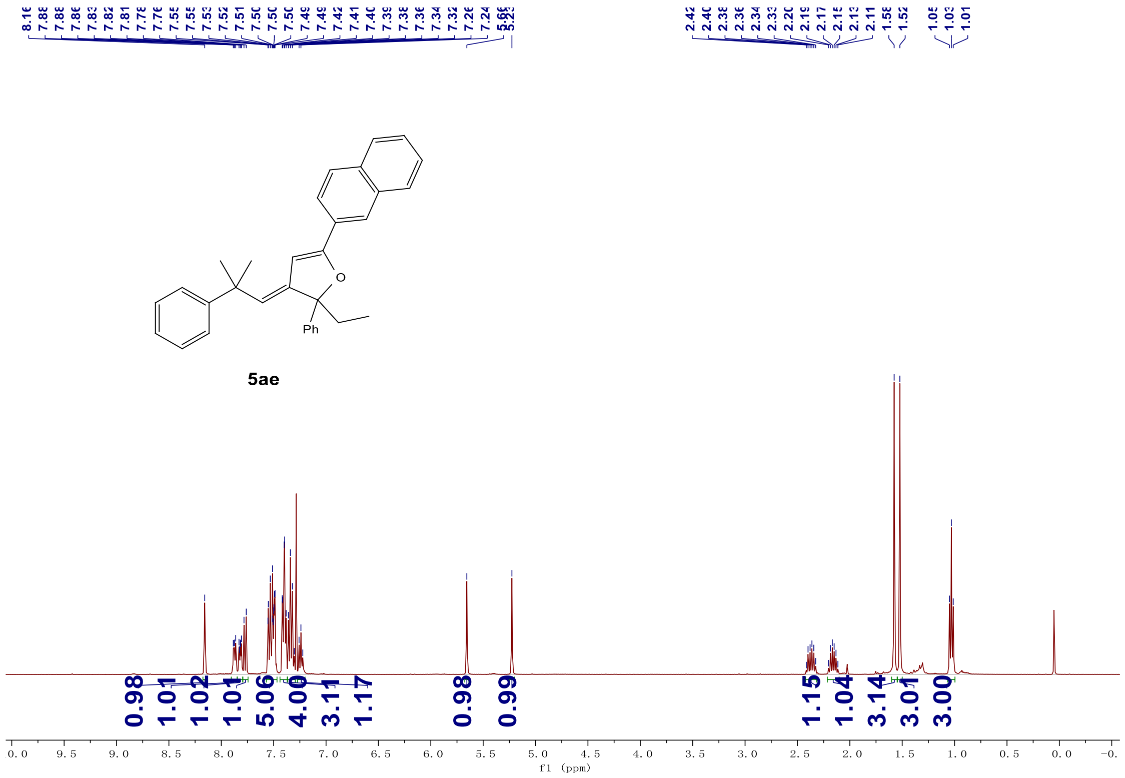

Supplementary Figure 63 ^{1}H NMR (400 MHz, $CDCl_3$) spectrum of compound **5ae**

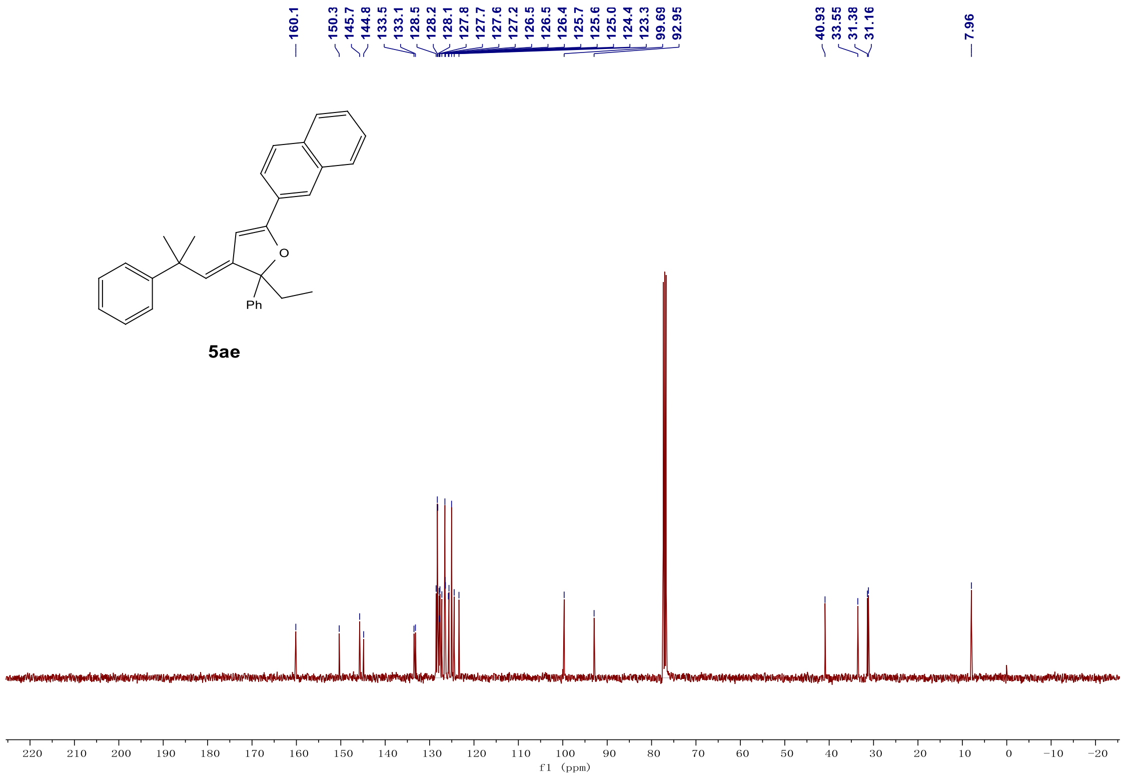

Supplementary Figure 64 ^{13}C NMR (101 MHz, $CDCl_3$) spectrum of compound **5ae**

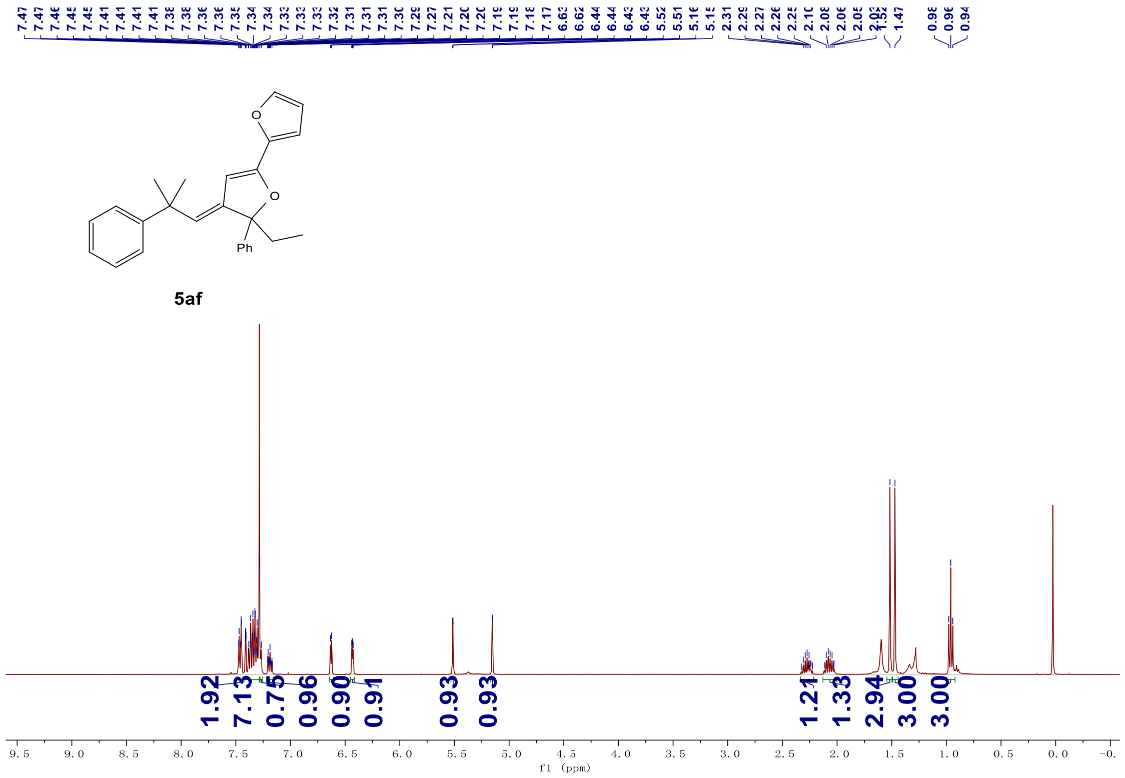

Supplementary Figure 65 ^{1}H NMR (400 MHz, $CDCl_3$) spectrum of compound **5af**

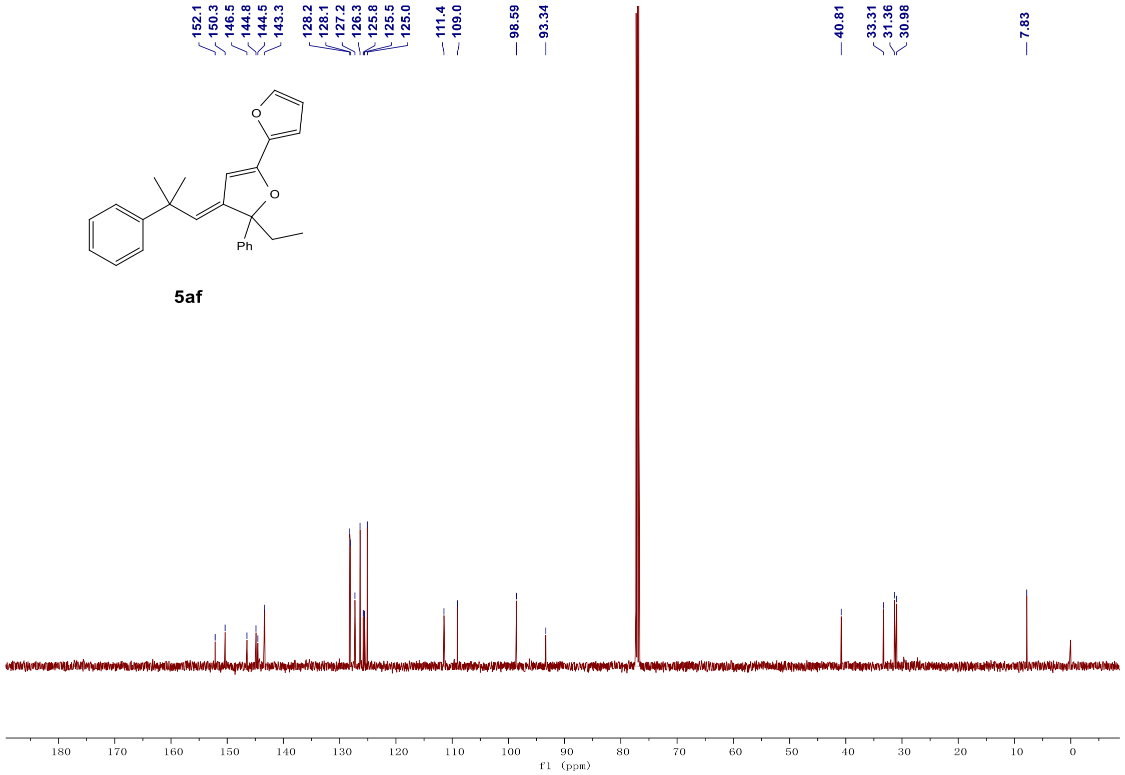

Supplementary Figure 66 ^{13}C NMR (101 MHz, $CDCl_3$) spectrum of compound **5af**

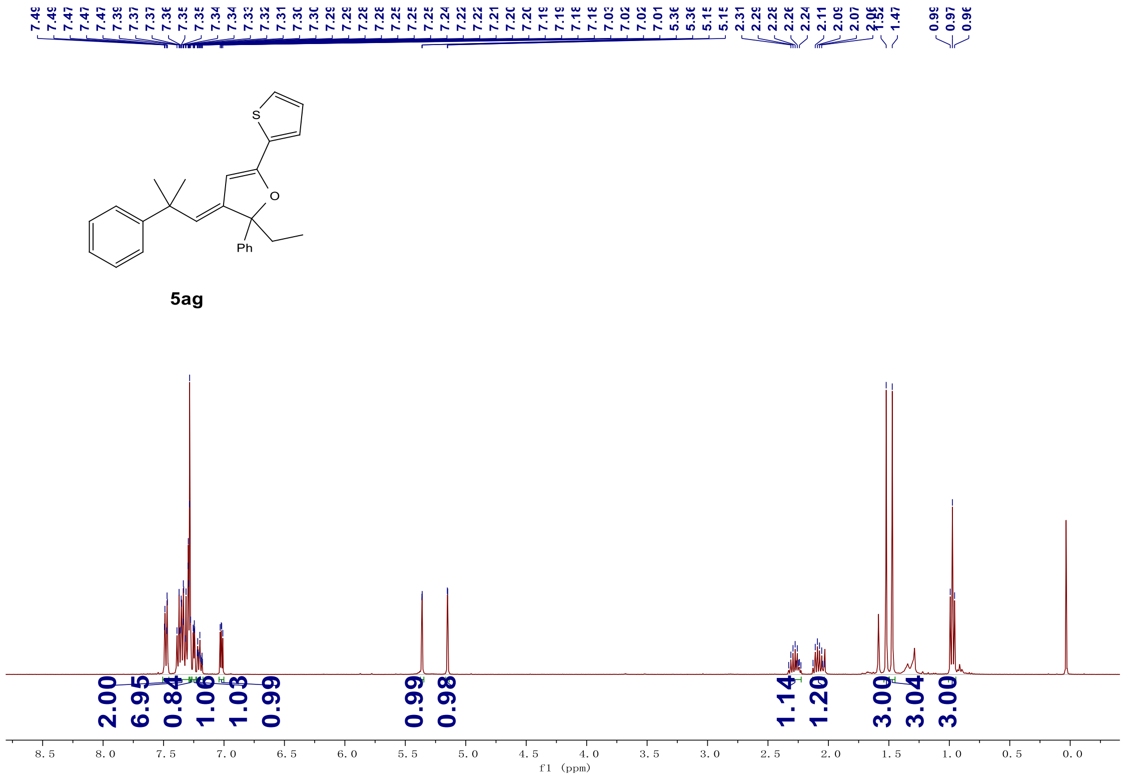

Supplementary Figure 67 ^{1}H NMR (400 MHz, $CDCl_3$) spectrum of compound **5ag**

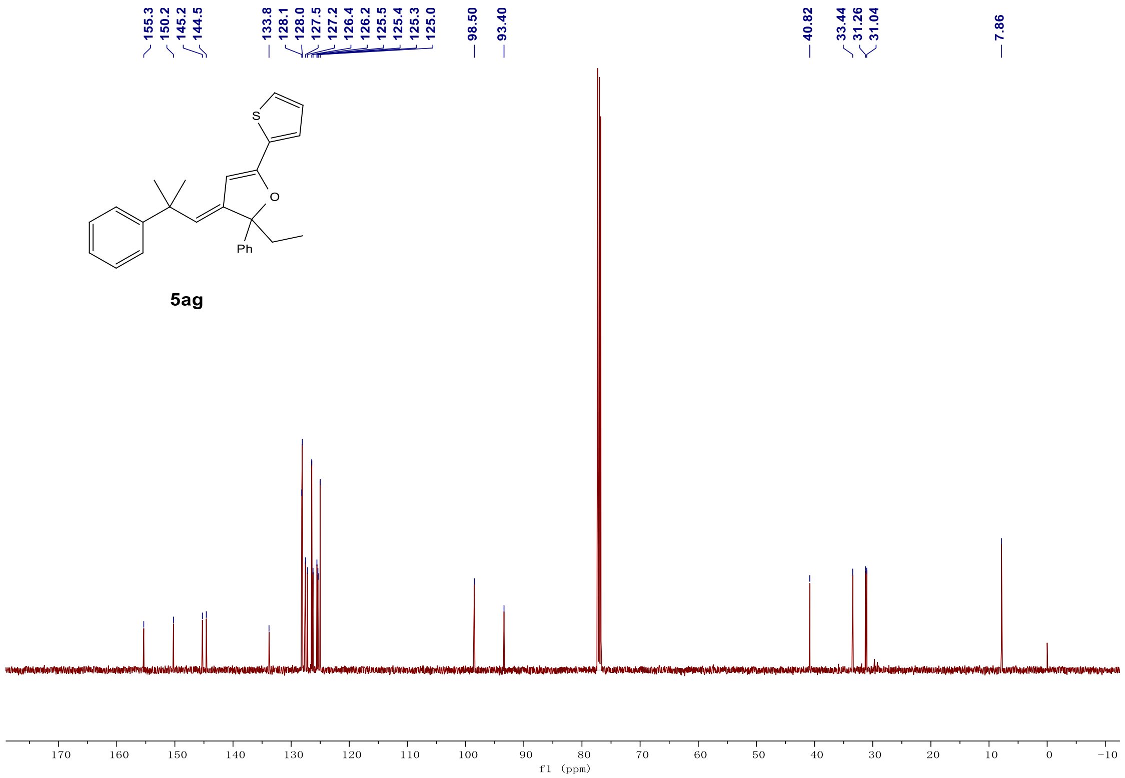

Supplementary Figure 68 ^{13}C NMR (101 MHz, $CDCl_3$) spectrum of compound **5ag**

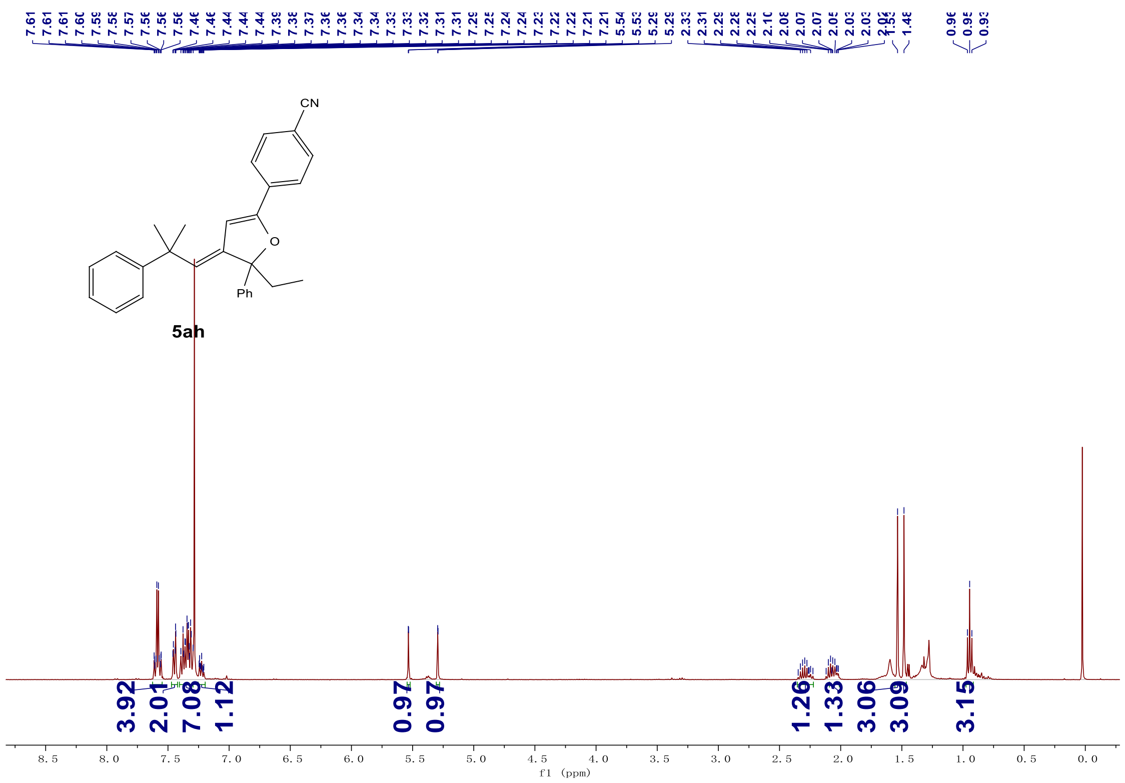

Supplementary Figure 69 ^{1}H NMR (400 MHz, $CDCl_3$) spectrum of compound **5ah**

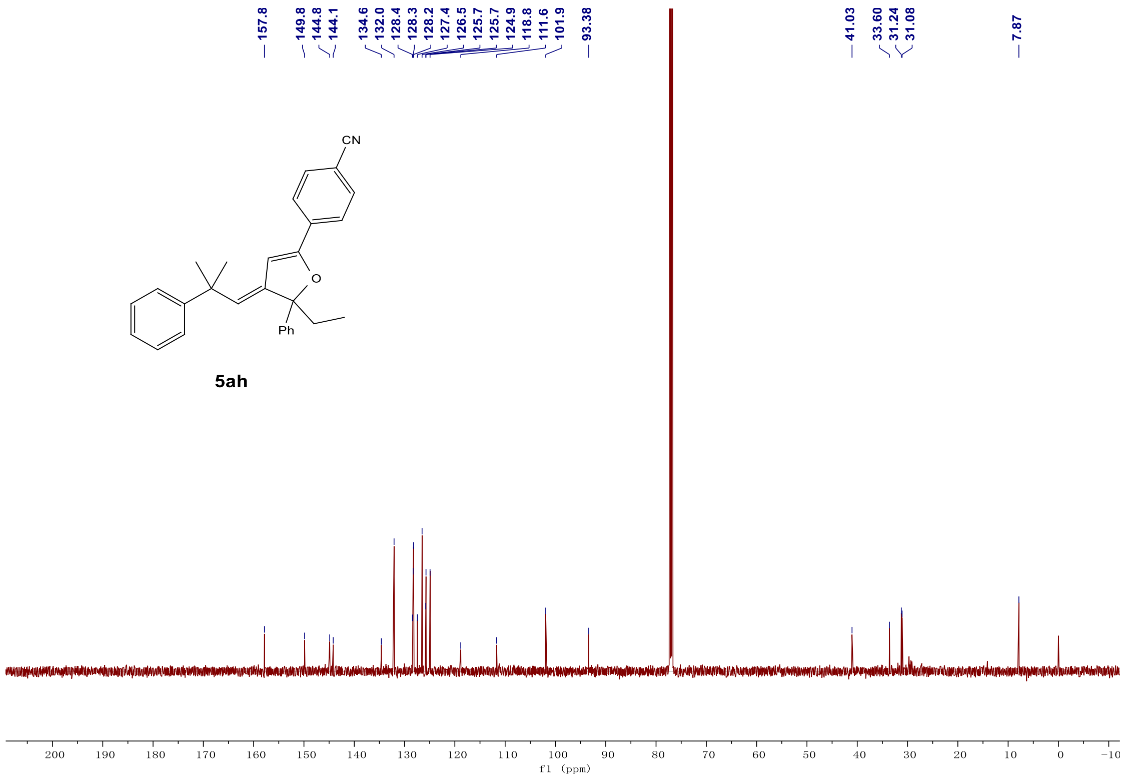

Supplementary Figure 70 ^{13}C NMR (101 MHz, $CDCl_3$) spectrum of compound **5ah**

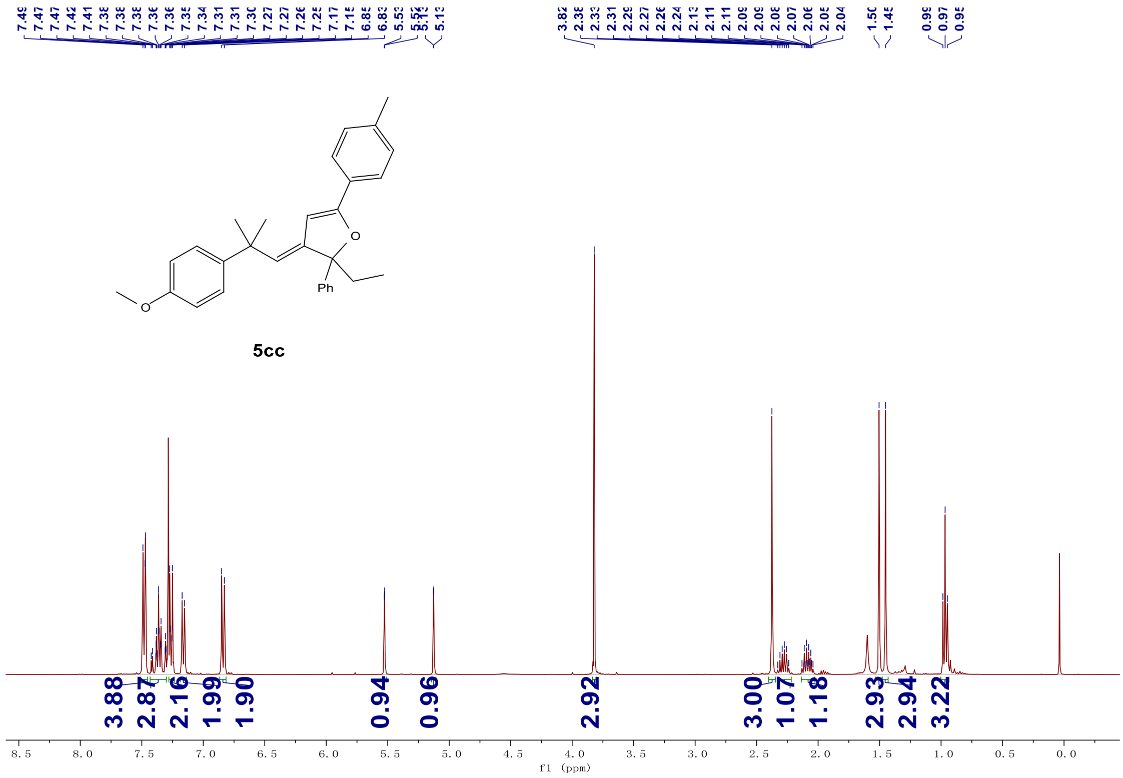

Supplementary Figure 71 ^{1}H NMR (400 MHz, $CDCl_3$) spectrum of compound **5cc**

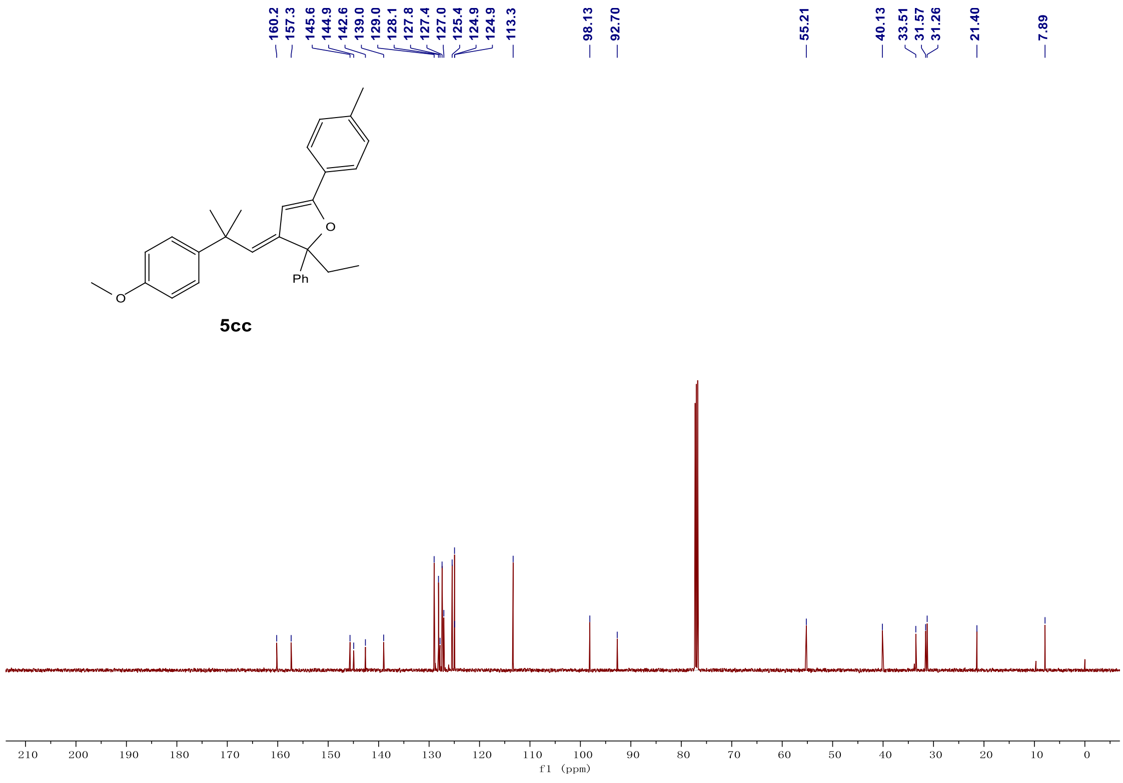

Supplementary Figure 72 ^{13}C NMR (101 MHz, $CDCl_3$) spectrum of compound **5cc**

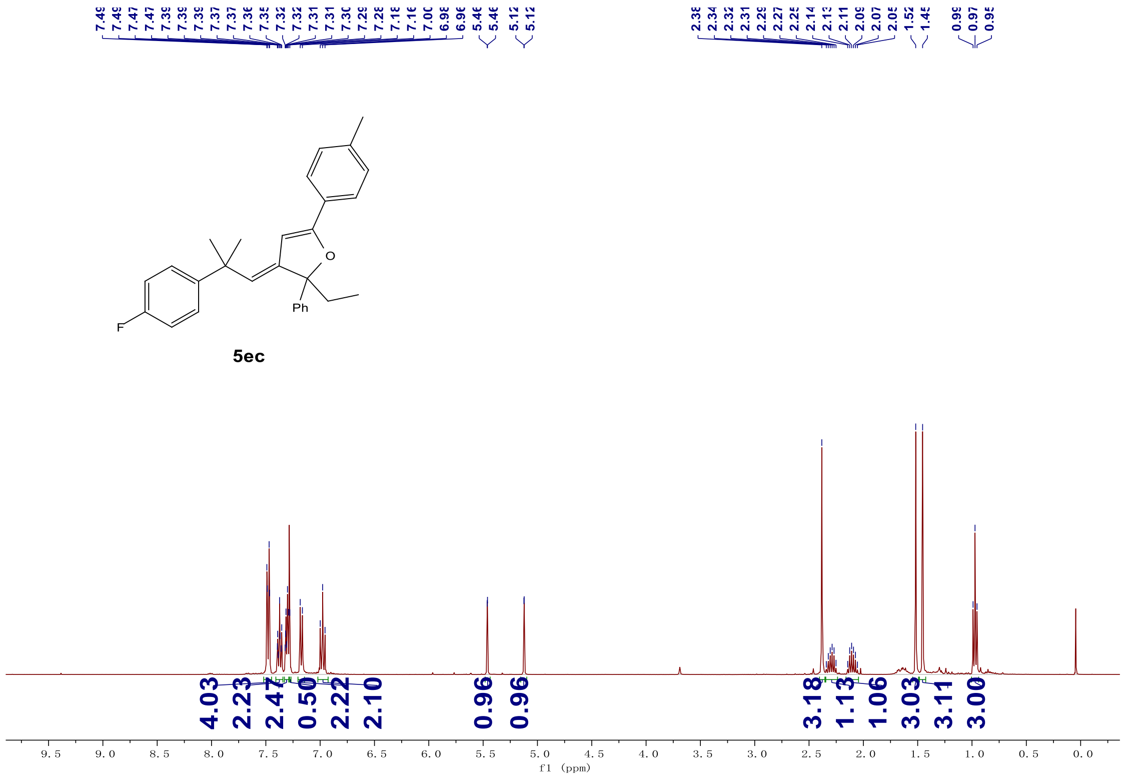

Supplementary Figure 73 ^{1}H NMR (400 MHz, $CDCl_3$) spectrum of compound **5ec**

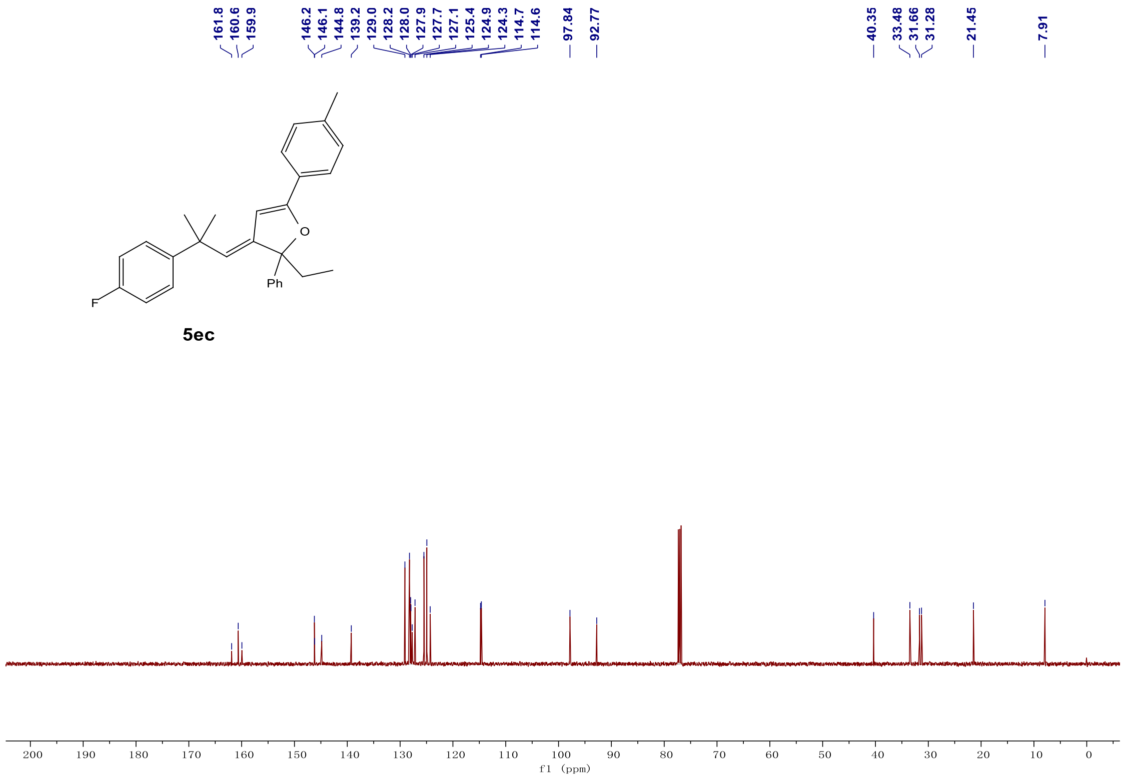

Supplementary Figure 74 ^{13}C NMR (101 MHz, $CDCl_3$) spectrum of compound **5ec**

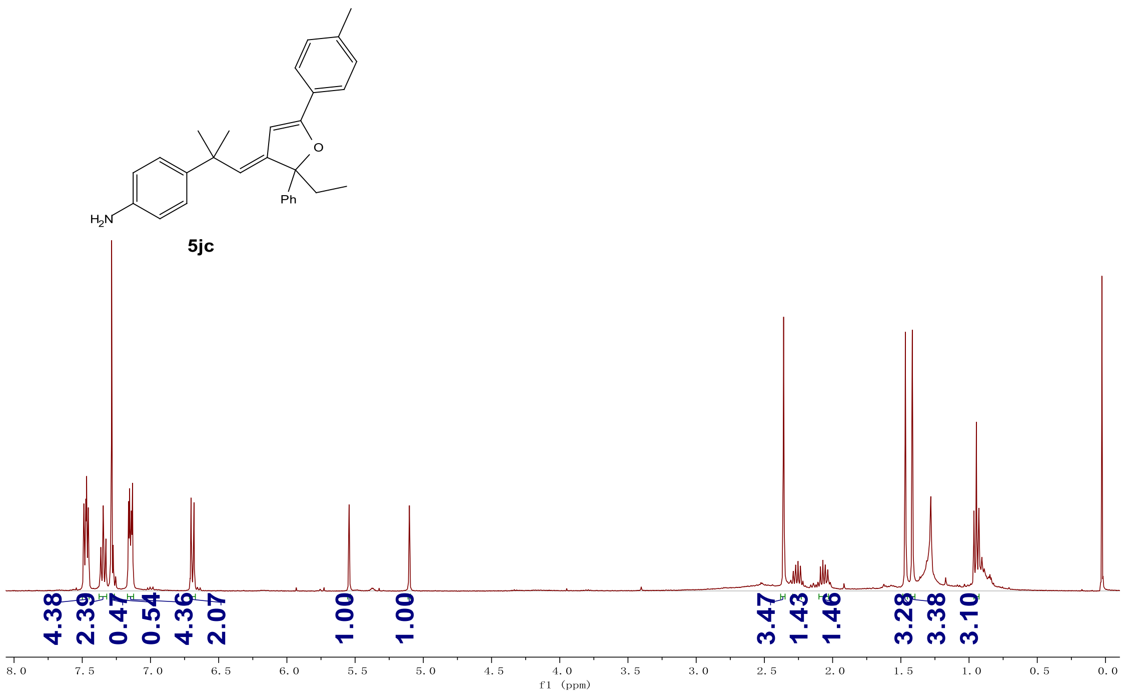

Supplementary Figure 75 ^{1}H NMR (400 MHz, $CDCl_3$) spectrum of compound **5jc**

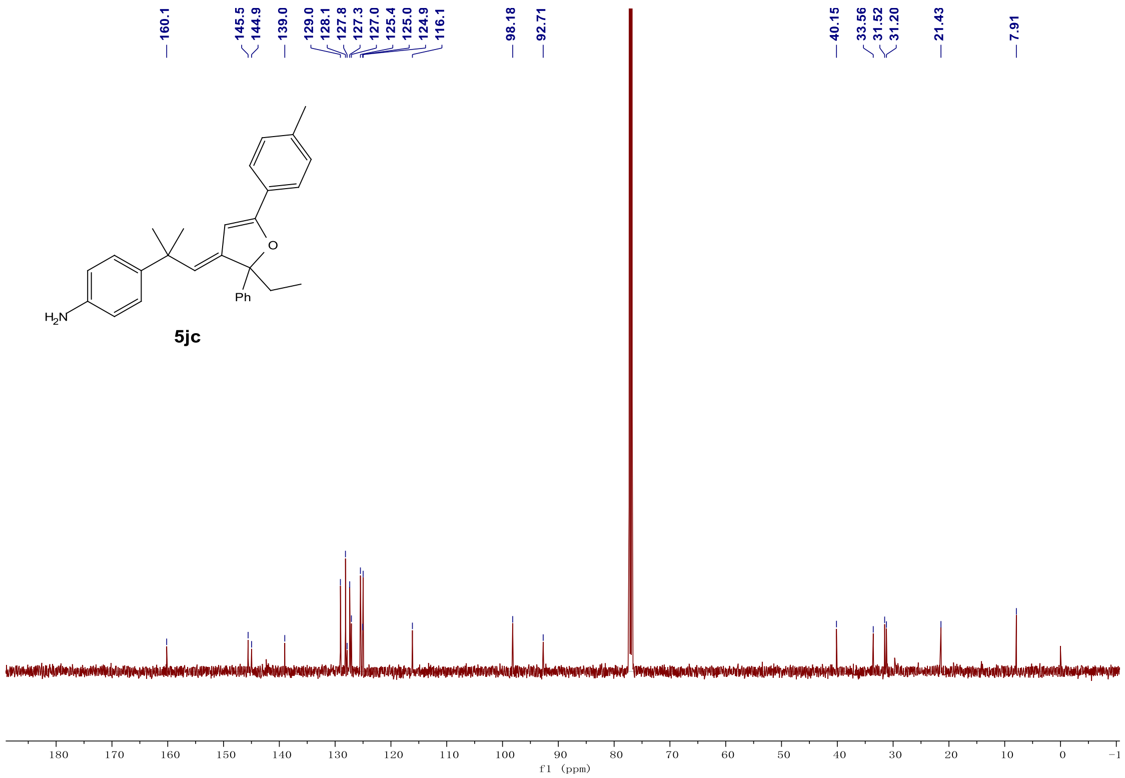

Supplementary Figure 76 ^{13}C NMR (101 MHz, $CDCl_3$) spectrum of compound **5jc**

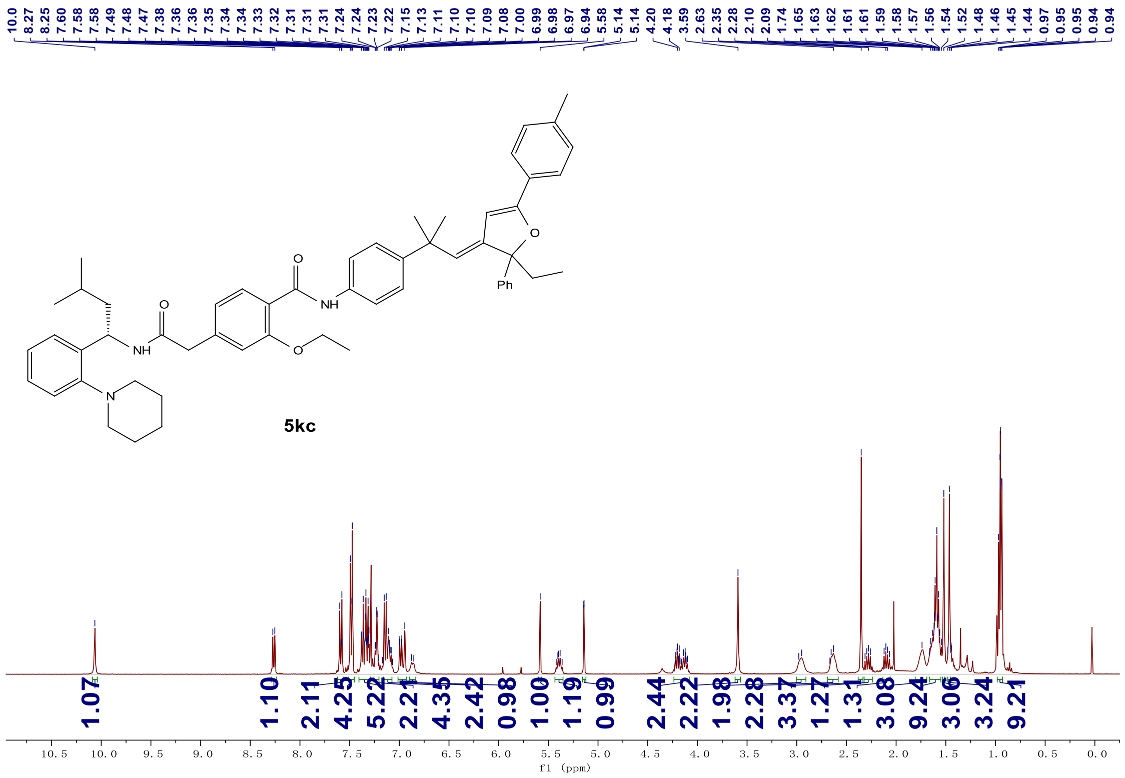

Supplementary Figure 77 ^{1}H NMR (400 MHz, $CDCl_3$) spectrum of compound **5kc**

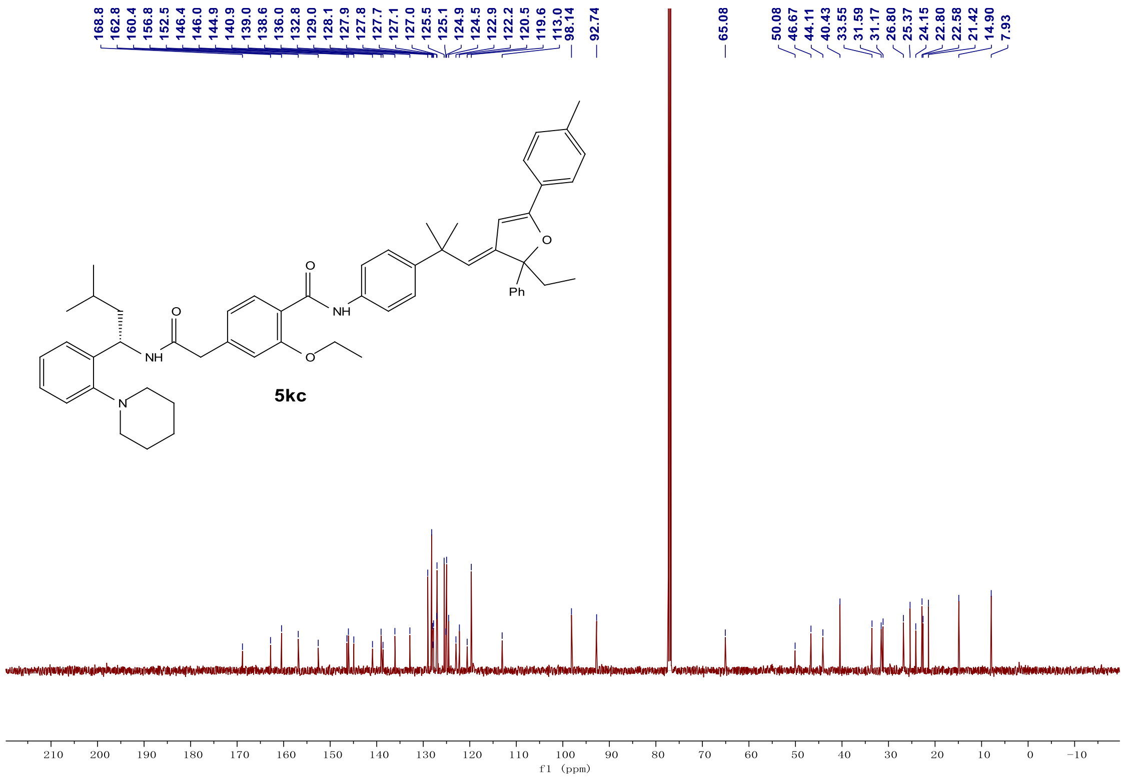

Supplementary Figure 78 ^{13}C NMR (101 MHz, $CDCl_3$) spectrum of compound **5kc**

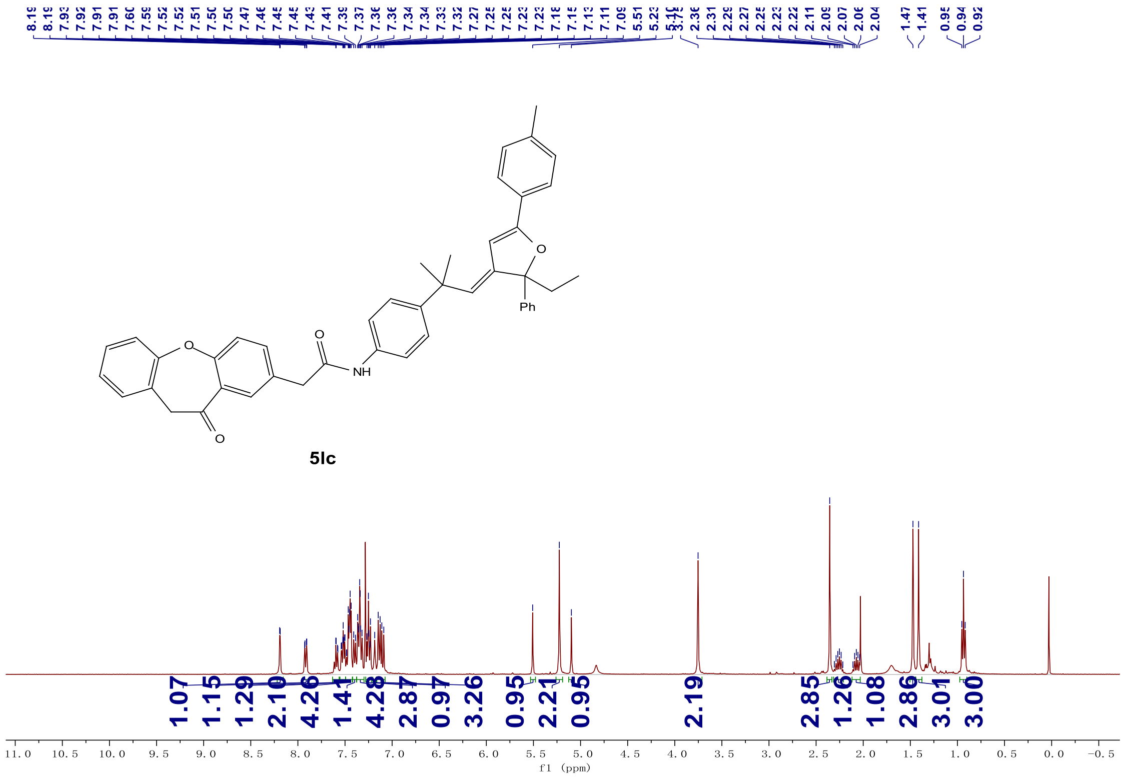

Supplementary Figure 79 ^{1}H NMR (400 MHz, $CDCl_3$) spectrum of compound **5lc**

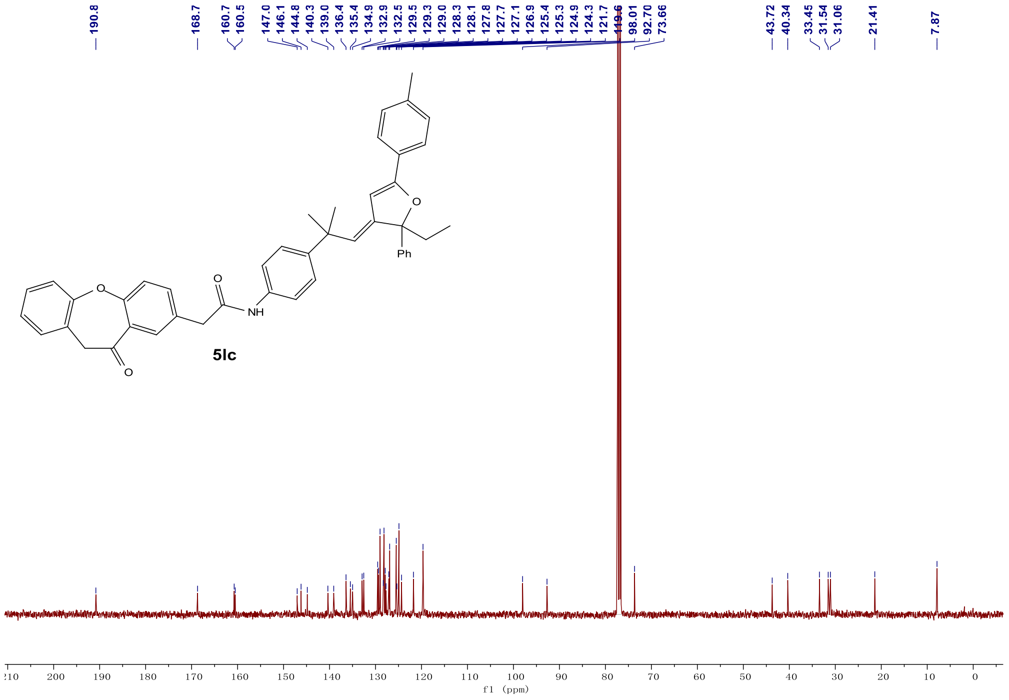

Supplementary Figure 80 ^{13}C NMR (101 MHz, $CDCl_3$) spectrum of compound **5lc**

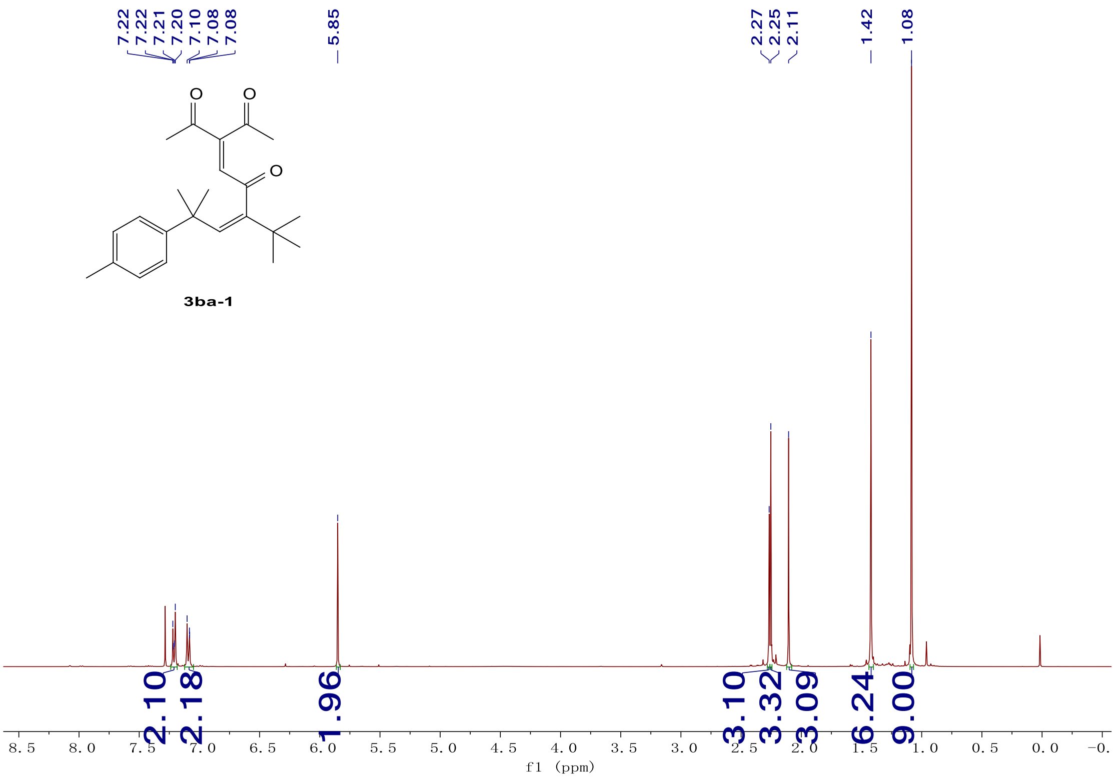

Supplementary Figure 81 ^{1}H NMR (400 MHz, $CDCl_3$) spectrum of compound **3ba-1**

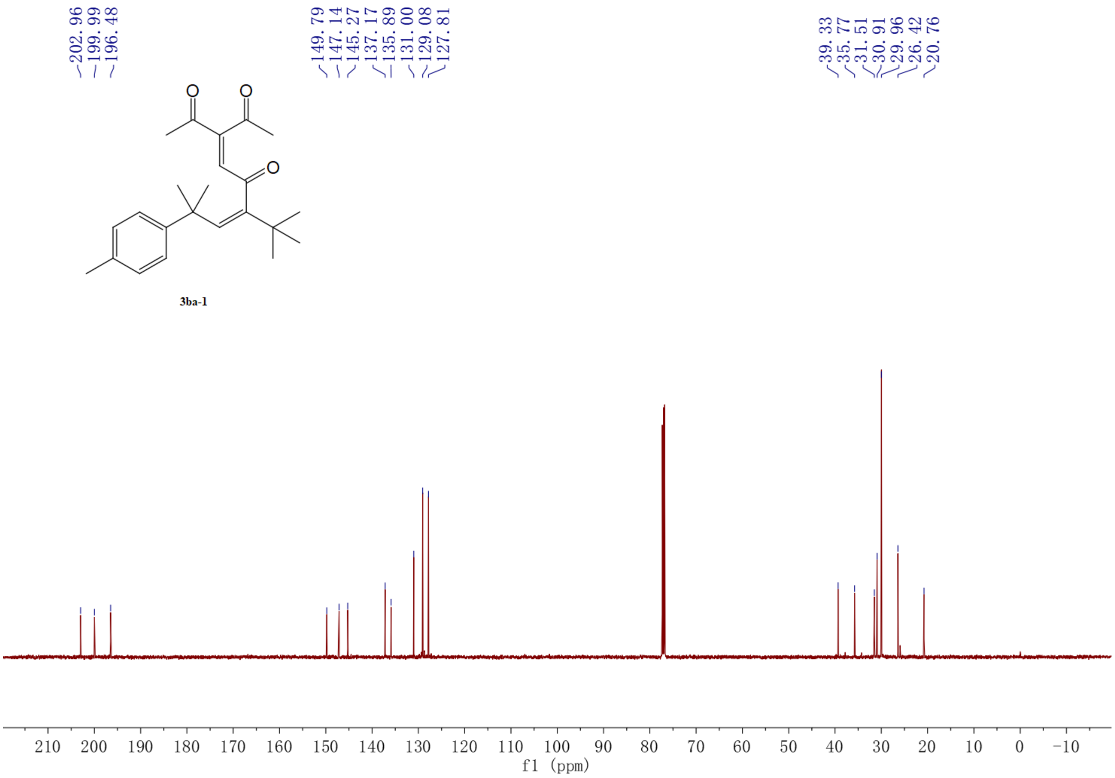

Supplementary Figure 82 ^{13}C NMR (101 MHz, $CDCl_3$) spectrum of compound **3ba-1**

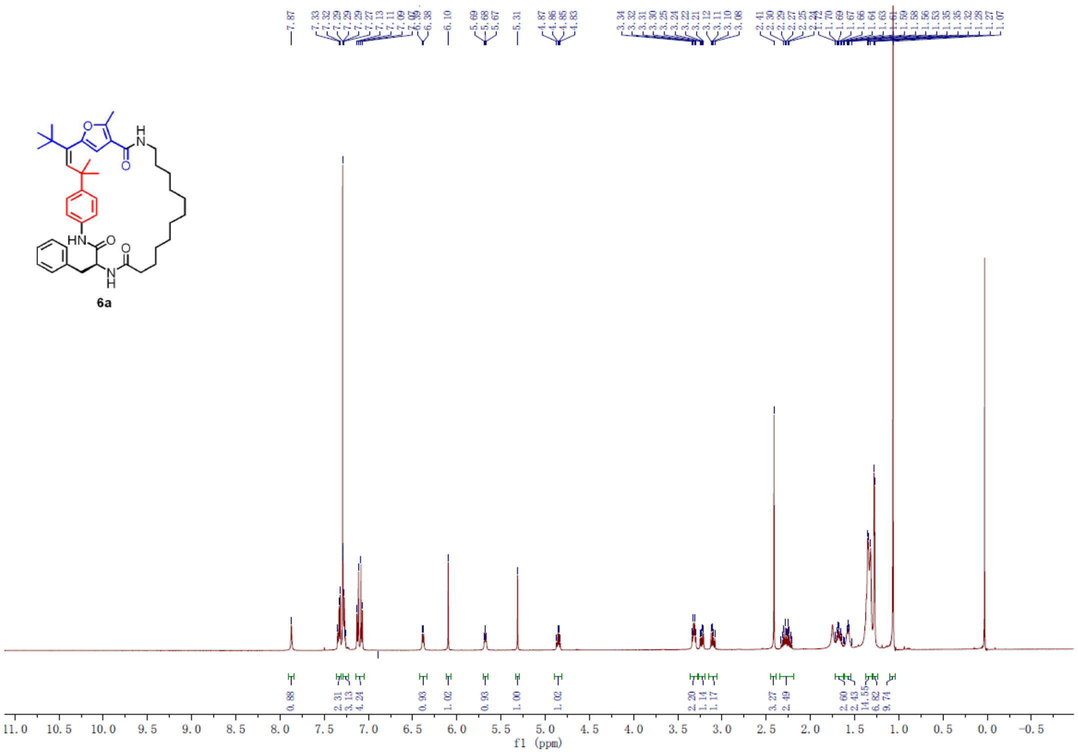

Supplementary Figure 83 ^{1}H NMR (400 MHz, $CDCl_3$) spectrum of compound **6a**

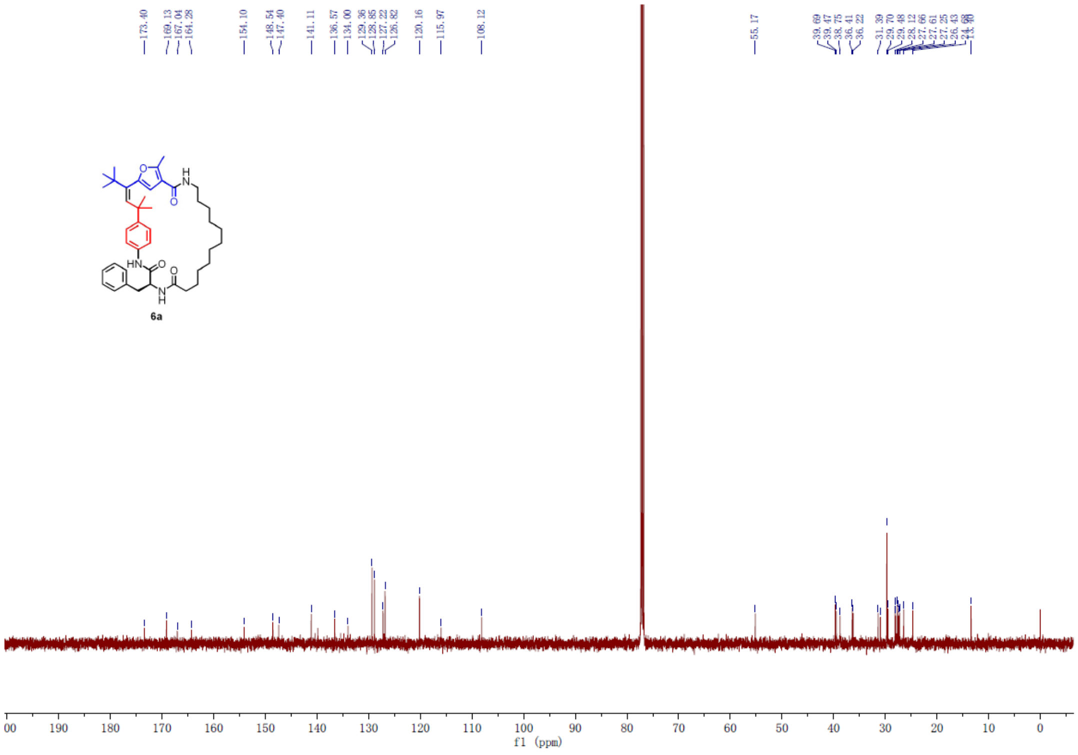

Supplementary Figure 84 ^{13}C NMR (101 MHz, $CDCl_3$) spectrum of compound **6a**

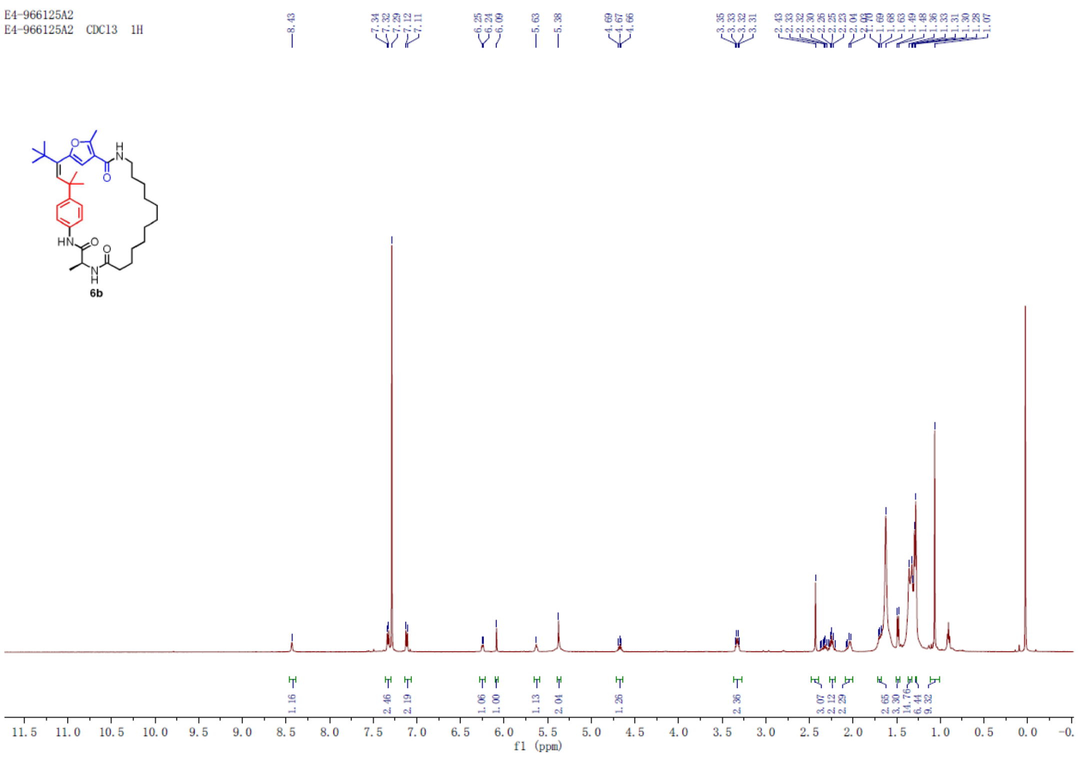

Supplementary Figure 85 ^{1}H NMR (400 MHz, $CDCl_3$) spectrum of compound **6b**

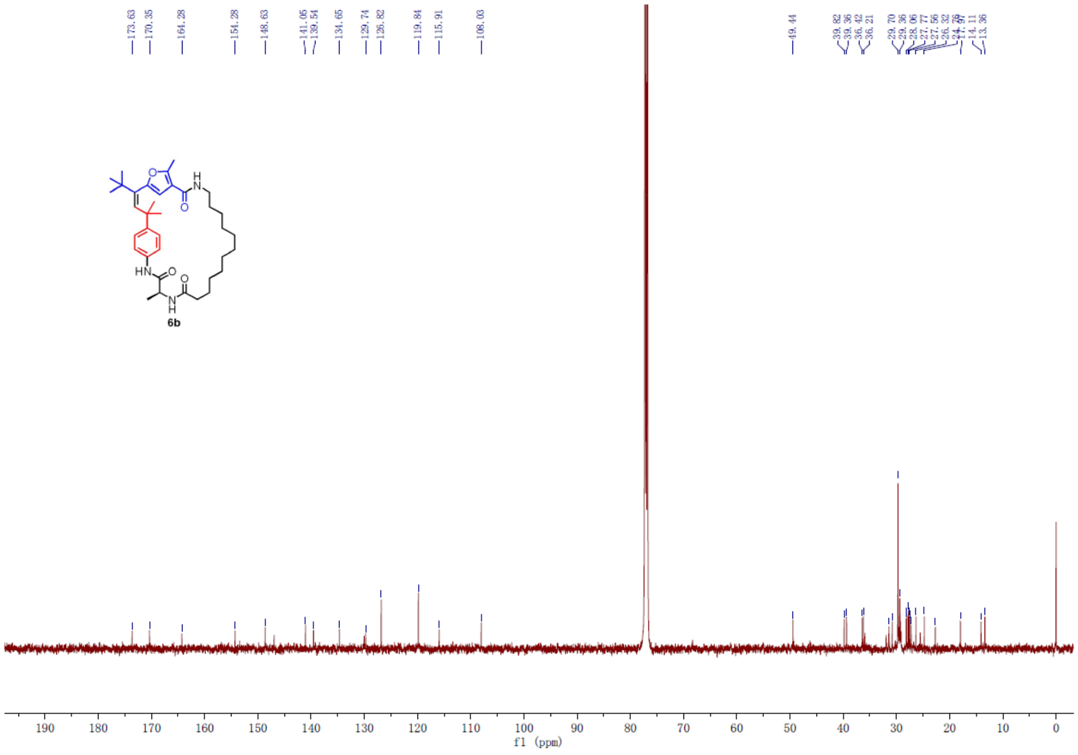

Supplementary Figure 86 ^{13}C NMR (101 MHz, $CDCl_3$) spectrum of compound **6b**

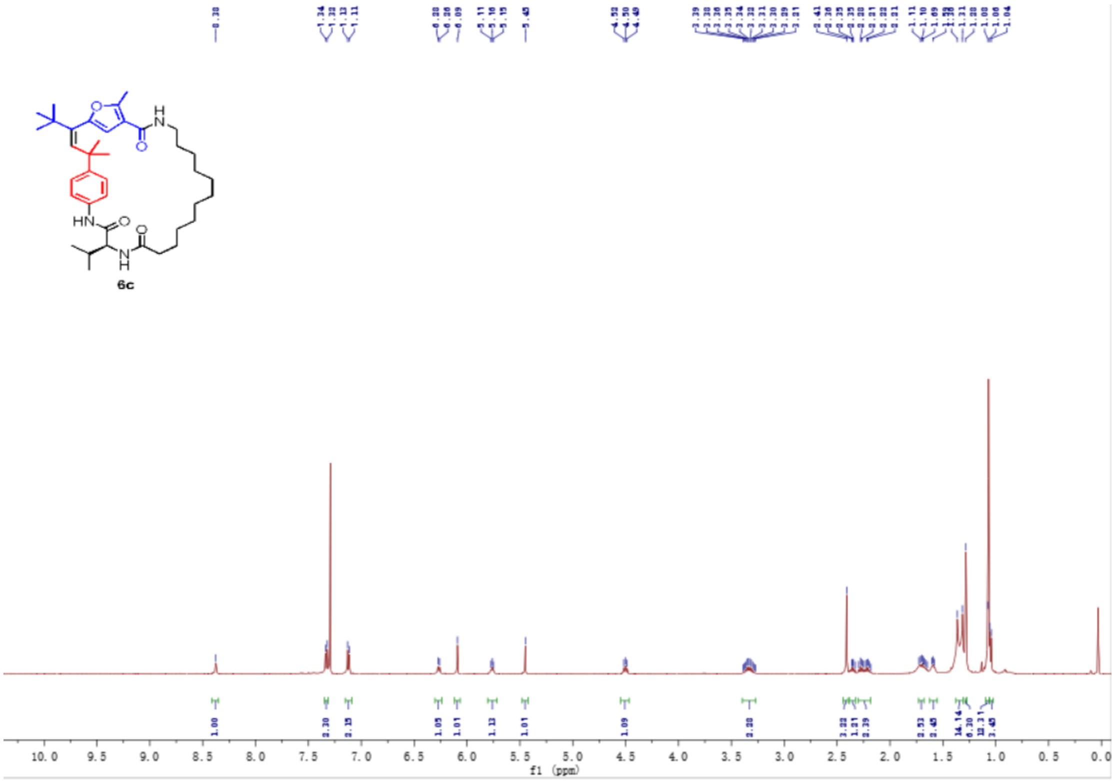

Supplementary Figure 87 ^{1}H NMR (400 MHz, $CDCl_3$) spectrum of compound **6c**

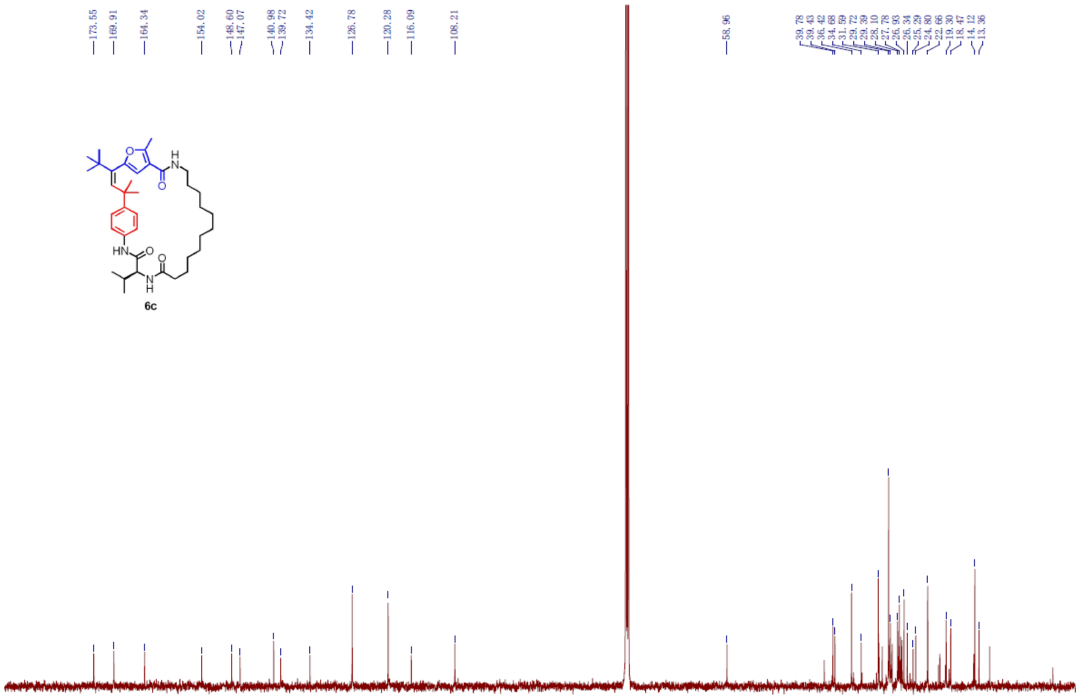

Supplementary Figure 88 ^{13}C NMR (101 MHz, $CDCl_3$) spectrum of compound **6c**

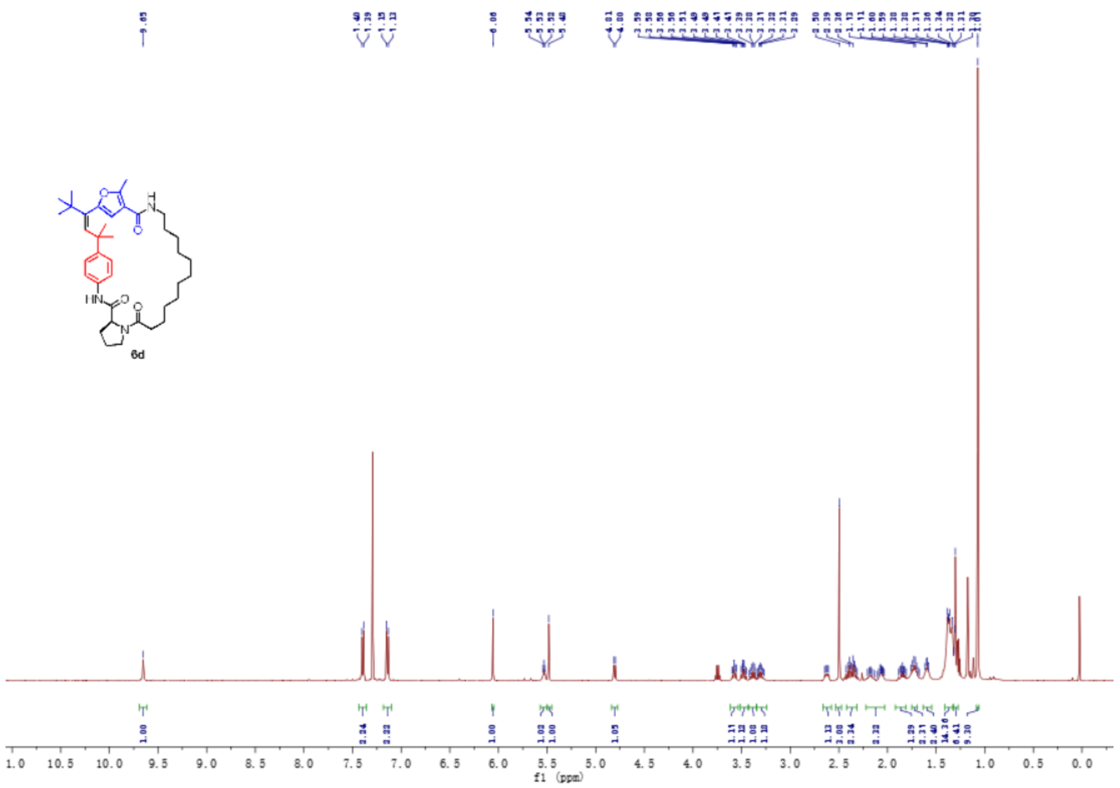

Supplementary Figure 89 ^{1}H NMR (400 MHz, $CDCl_3$) spectrum of compound **6d**

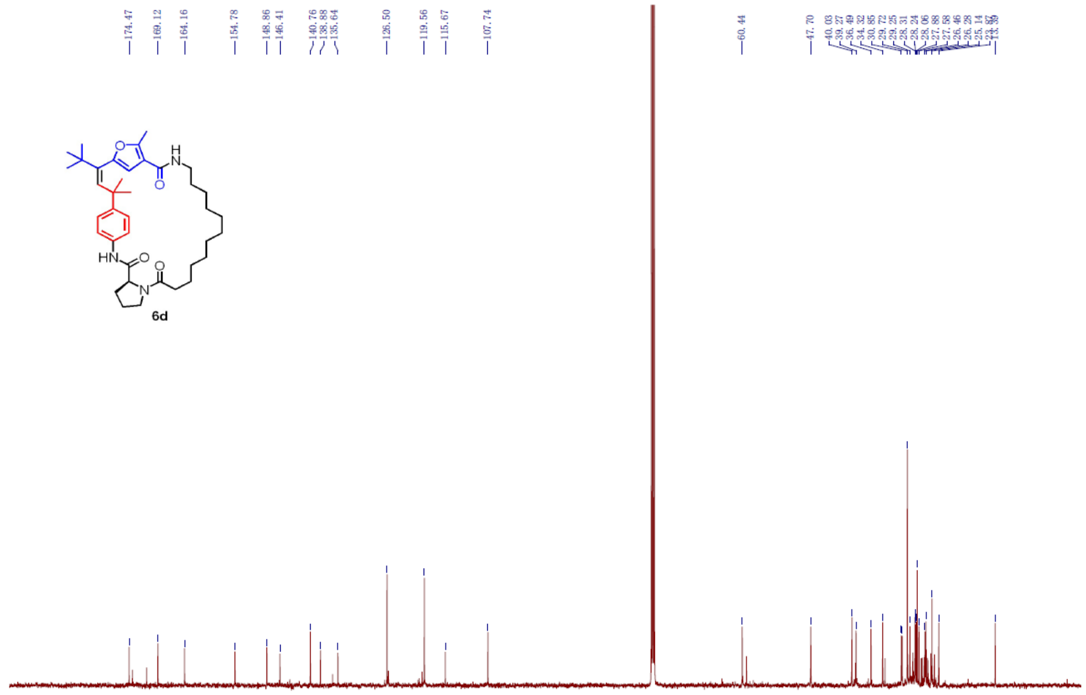

Supplementary Figure 90 ^{13}C NMR (101 MHz, $CDCl_3$) spectrum of compound **6d**

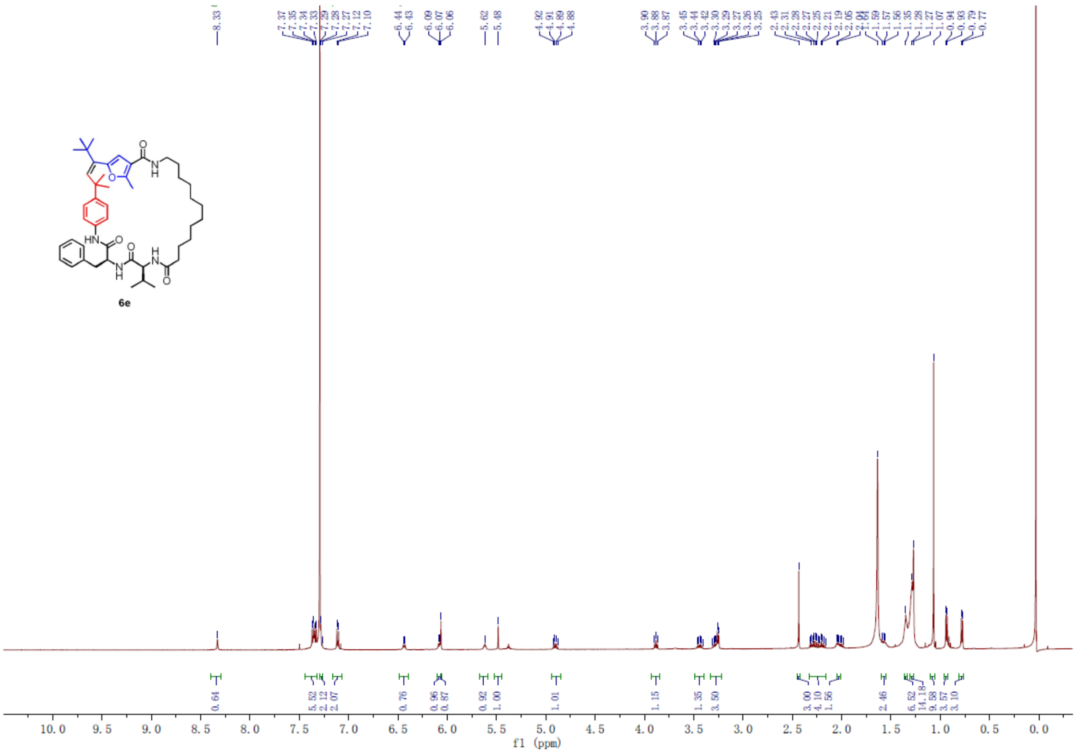

Supplementary Figure 91 ^{1}H NMR (400 MHz, $CDCl_3$) spectrum of compound **6e**

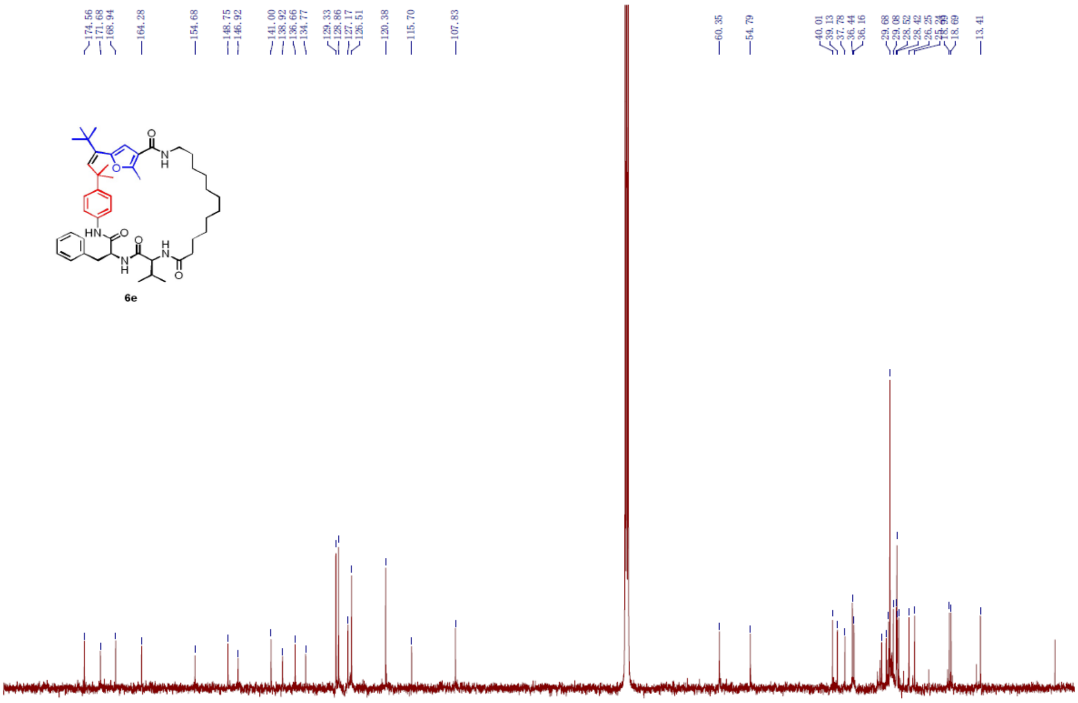

Supplementary Figure 92 ^{13}C NMR (101 MHz, $CDCl_3$) spectrum of compound **6e**

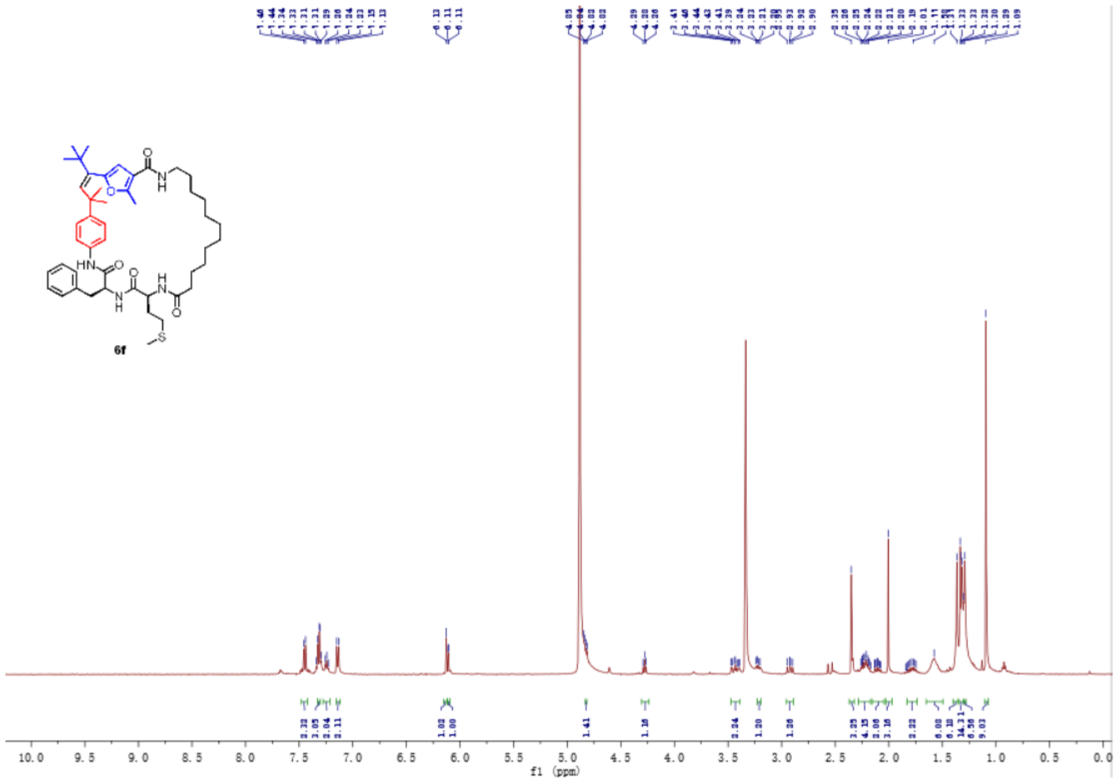

Supplementary Figure 93 ^{1}H NMR (400 MHz, $CDCl_3$) spectrum of compound **6f**

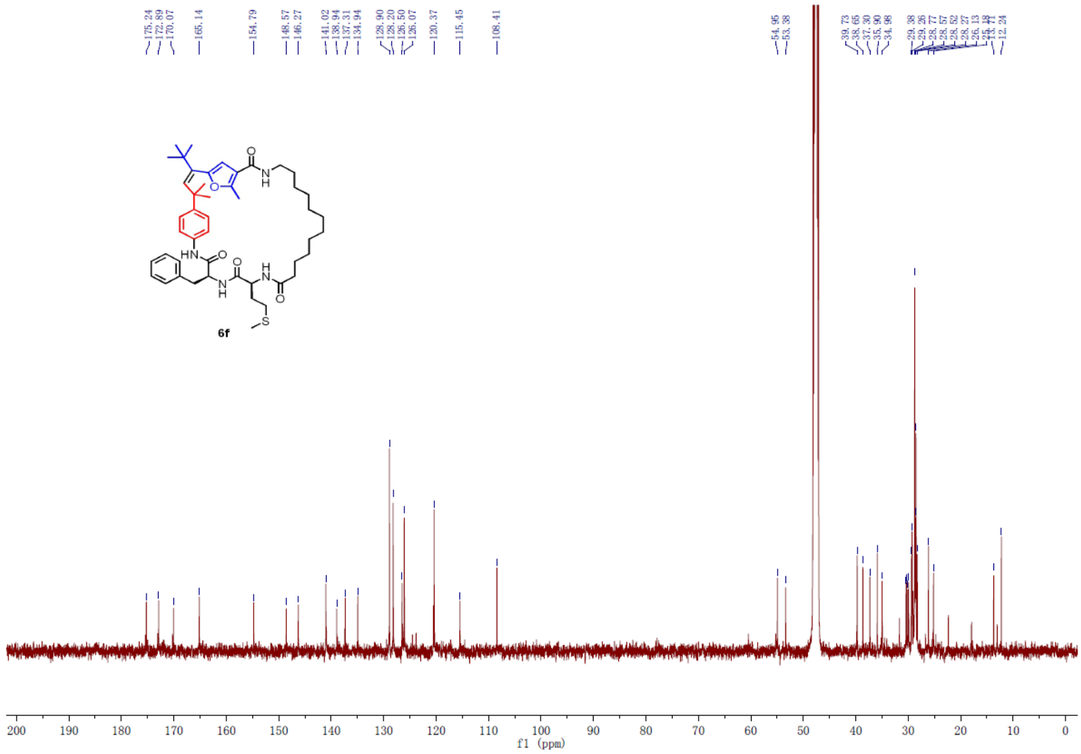

Supplementary Figure 94 ^{13}C NMR (101 MHz, $CDCl_3$) spectrum of compound **6f**

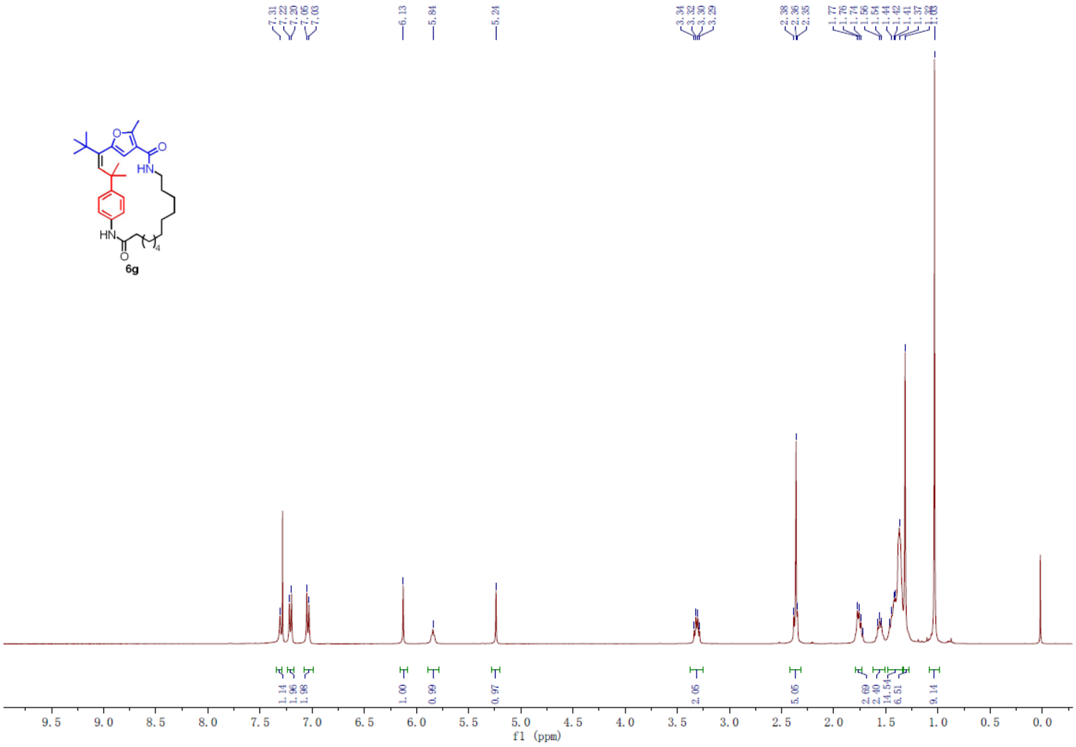

Supplementary Figure 95 ^{1}H NMR (400 MHz, $CDCl_3$) spectrum of compound **6g**

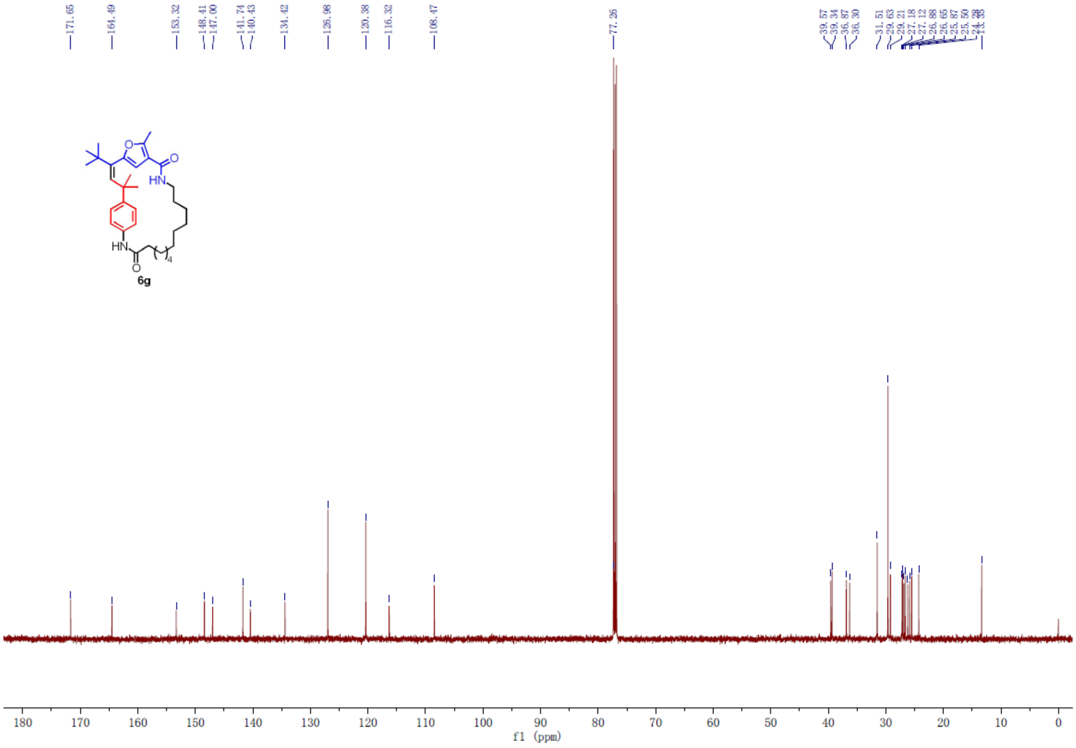

Supplementary Figure 96 ^{13}C NMR (101 MHz, $CDCl_3$) spectrum of compound **6g**

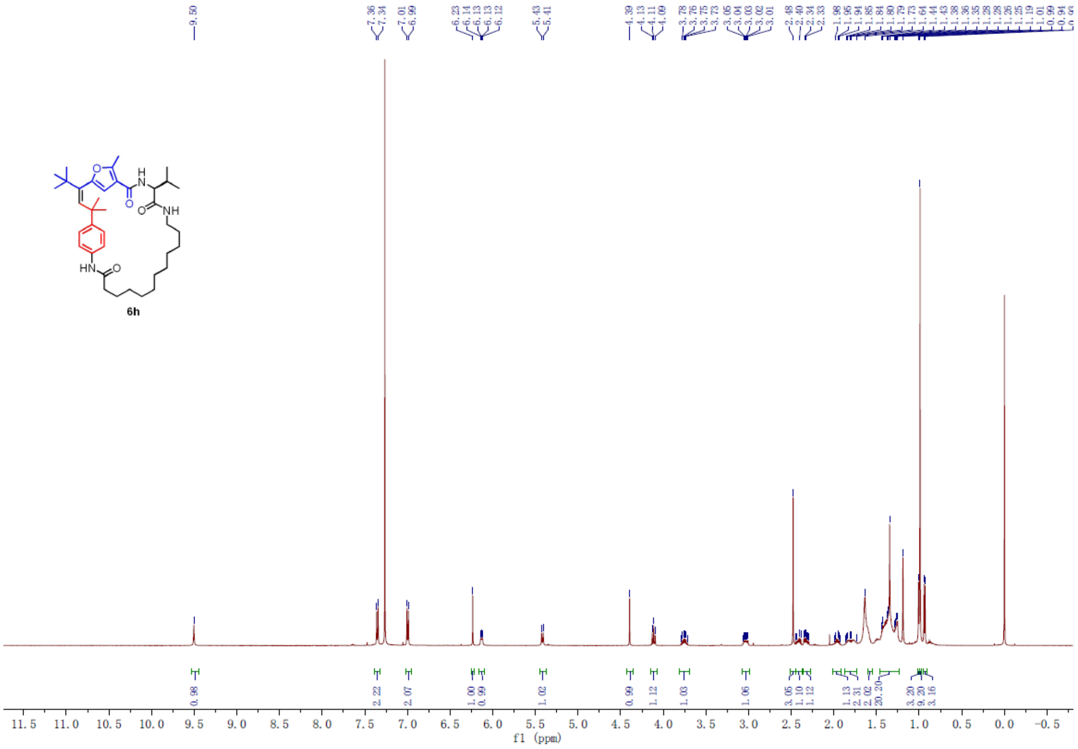

Supplementary Figure 97 ^{1}H NMR (400 MHz, $CDCl_3$) spectrum of compound **6h**

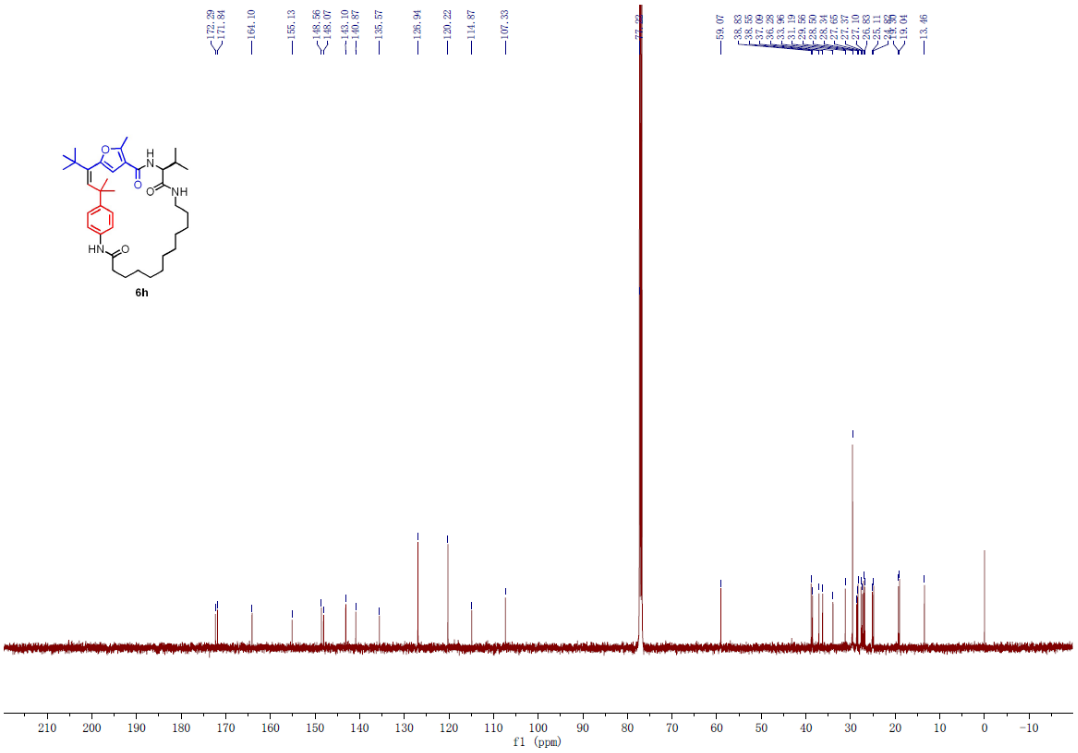

Supplementary Figure 98 ^{13}C NMR (101 MHz, $CDCl_3$) spectrum of compound **6h**

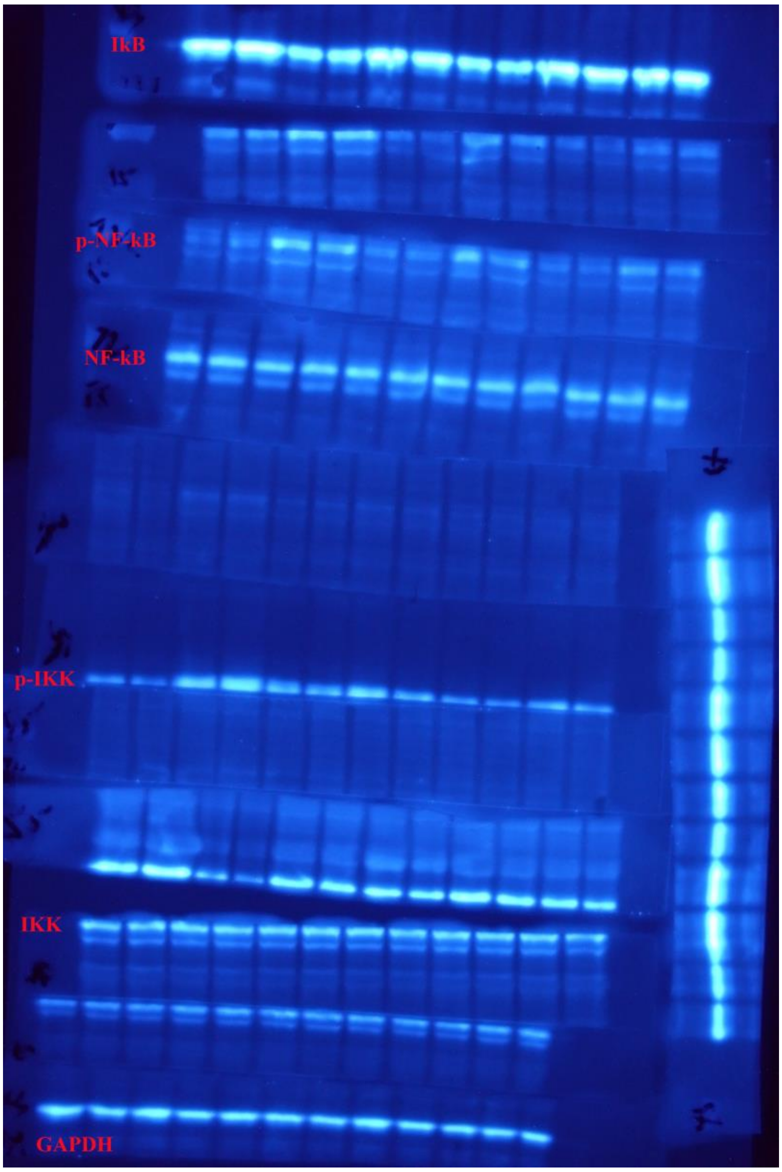

Supplementary Figure 99. Uncropped and unprocessed scans of blots showed in Figure 6b.

Supplementary References

1 Wu, Z. *et al.* Palladium-Catalyzed Alkylation with Alkyl Halides by C(sp(3))-H Activation. *Angew Chem Int Ed Engl* **56**, 12288-12291 (2017).

2 Gutierrez-Bonet, A., Julia-Hernandez, F., de Luis, B. & Martin, R. Pd-Catalyzed C(sp(3))-H Functionalization/Carbenoid Insertion: All-Carbon Quaternary Centers via Multiple C-C Bond Formation. *J Am Chem Soc* **138**, 6384-6387 (2016).

3 Mori, K., Isogai, R., Kamei, Y., Yamanaka, M. & Akiyama, T. Chiral Magnesium Bisphosphate-Catalyzed Asymmetric Double C(sp(3))-H Bond Functionalization Based on Sequential Hydride Shift/Cyclization Process. *J Am Chem Soc* **140**, 6203-6207 (2018).

4 Xia, Y. *et al.* Palladium-catalyzed carbene migratory insertion using conjugated ene-yne-ketones as carbene precursors. *J Am Chem Soc* **135**, 13502-13511 (2013).

5 Dudnik, A. S. *et al.* Metal-catalyzed 1,2-shift of diverse migrating groups in allenyl systems as a new paradigm toward densely functionalized heterocycles. *J Am Chem Soc* **130**, 1440-1452 (2008).

6 Zhao, Y. & Truhlar, D. G. The M06 suite of density functionals for main group thermochemistry, thermochemical kinetics, noncovalent interactions, excited states, and transition elements: two new functionals and systematic testing of four M06-class functionals and 12 other functionals. *Theoretical Chemistry Accounts* **120**, 215-241 (2007).

7 Marenich, A. V., Cramer, C. J. & Truhlar, D. G. Universal solvation model based on solute electron density and on a continuum model of the solvent defined by the bulk dielectric constant and atomic surface tensions. *J Phys Chem B* **113**, 6378-6396 (2009).

8 Fukui, K. Formulation of the reaction coordinate. *The Journal of Physical Chemistry* **74**, 4161-4163 (1970).

9 Fukui, K. The path of chemical reactions - the IRC approach. *Accounts of Chemical Research* **14**, 363-368 (2002).

10 Stoll, H. *et al.* Cu and Ag as one‐valence‐electron atoms: CI results and quadrupole corrections for Cu2, Ag2, CuH, and AgH. *The Journal of Chemical Physics* **81**, 2732-2736 (1984).

11 Schwerdtfeger, P., Dolg, M., Schwarz, W. H. E., Bowmaker, G. A. & Boyd, P. D. W. Relativistic effects in gold chemistry. I. Diatomic gold compounds. *The Journal of Chemical Physics* **91**, 1762-1774 (1989).

12 Andrae, D., Huermann, U., Dolg, M., Stoll, H. & Preu, H. Energy-adjustedab initio pseudopotentials for the second and third row transition elements: Molecular test for

M2 (M=Ag, Au) and MH (M=Ru, Os). *Theoretica Chimica Acta* **78**, 247-266 (1991).

13 Nicklass, A., Dolg, M., Stoll, H. & Preuss, H. Ab initioenergy‐adjusted pseudopotentials for the noble gases Ne through Xe: Calculation of atomic dipole and quadrupole polarizabilities. *The Journal of Chemical Physics* **102**, 8942-8952 (1995).

14 Igel-Mann, G., Stoll, H. & Preuss, H. Pseudopotentials for main group elements (IIIa through VIIa). *Molecular Physics* **65**, 1321-1328 (2006).

15 Bergner, A., Dolg, M., Küchle, W., Stoll, H. & Preuß, H. Ab initio energy-adjusted pseudopotentials for elements of groups 13–17. *Molecular Physics* **80**, 1431-1441 (2006).

16 Höllwarth, A. *et al.* A set of d-polarization functions for pseudo-potential basis sets of the main group elements Al Bi and f-type polarization functions for Zn, Cd, Hg. *Chemical Physics Letters* **208**, 237-240 (1993).

17 Frisch, M. J.; Trucks, G. W.; Schlegel, H. B.; Scuseria, G. E.; Robb, M. A.; Cheeseman, J. R.; Scalmani, G.; Barone, V.; Mennucci, B.; Petersson, G. A.; Nakatsuji, H.; Caricato, M.; Li, X.; Hratchian, H. P.; Izmaylov, A. F.; Bloino, J.; Zheng, G.; Sonnenberg, J. L.; Hada, M.; Ehara, M.; Toyota, K.; Fukuda, R.; Hasegawa, J.; Ishida, M.; Nakajima, T.; Honda, Y.; Kitao, O.; Nakai, H.; Vreven, T.; Montgomery, J. A., Jr.; Peralta, J. E.; Ogliaro, F.; Bearpark, M.; Heyd, J. J.; Brothers, E.; Kudin, K. N.; Staroverov, V. N.; Kobayashi, R.; Normand, J.; Raghavachari, K.; Rendell, A.; Burant, J. C.; Iyengar, S. S.; Tomasi, J.; Cossi, M.; Rega, N.; Millam, J. M.; Klene, M.; Knox, J. E.; Cross, J. B.; Bakken, V.; Adamo, C.; Jaramillo, J.; Gomperts, R.; Stratmann, R. E.; Yazyev, O.; Austin, A. J.; Cammi, R.; Pomelli, C.; Ochterski, J. W.; Martin, R. L.; Morokuma, K.; Zakrzewski, V. G.; Voth, G. A.; Salvador, P.; Dannenberg, J. J.; Dapprich, S.; Daniels, A. D.; Farkas, O.; Foresman, J. B.; Ortiz, J. V.; Cioslowski, J.; Fox, D. J. Gaussian 09, revision D.01; Gaussian, Inc.: Wallingford, CT, 2009.
