# Supplementary material for: Marine furanocembranoids-inspired macrocycles enabled by Pd-catalyzed unactivated C(sp3)-H olefination mediated by donor/donor carbenes
Source: Nat Commun. 2021 Feb 26;12:1304. doi: 10.1038/s41467-021-21484-x (PMC7910576; doi:10.1038/s41467-021-21484-x)
Supplement: Supplementary file 3 — Description of Additional Supplementary Files [file 41467_2021_21484_MOESM3_ESM.docx]

**Description of Additional Supplementary Files**

**Supplementary Data 1:**

Crystallographic Data of 3ac and 5ab

**Supplementary Data 2:**

Cartesian coordinates of DFT calculation
