# Supplementary material for: Marine furanocembranoids-inspired macrocycles enabled by Pd-catalyzed unactivated C(sp3)-H olefination mediated by donor/donor carbenes
Source: Nat Commun. 2021 Feb 26;12:1304. doi: 10.1038/s41467-021-21484-x (PMC7910576; doi:10.1038/s41467-021-21484-x)
Supplement: Supplementary file 4 — Supplementary Data 1 [file 41467_2021_21484_MOESM4_ESM.docx]

# X-ray molecular structure and Crystallographic Data

| Fractional Atomic Coordinates (×10^4^) and Equivalent Isotropic Displacement Parameters (Å^2^×10^3^) for 22019145_0m. U_eq_ is defined as 1/3 of the trace of the orthogonalised U_IJ_ tensor. | | | | |
| --- | --- | --- | --- | --- |
| Atom | ***x*** | ***y*** | ***z*** | U(eq) |
| O^2^ | 3554.0(12) | 820.8(12) | 5518.8(9) | 28.5(3) |
| O^1^ | 4524.0(11) | 2334.2(11) | 2149.5(9) | 24.0(3) |
| C^7^ | 4501.7(16) | 1666.3(16) | 4958.0(13) | 22.2(3) |
| C^8^ | 4775.9(16) | 2086.4(16) | 3785.1(13) | 21.7(3) |
| C^10^ | 5952.4(16) | 2543.3(16) | 2041.0(13) | 22.7(3) |
| C^9^ | 6138.6(17) | 2406.7(16) | 3013.2(13) | 23.3(3) |
| C^6^ | 5432.8(16) | 2283.4(16) | 5459.0(13) | 23.1(3) |
| C^18^ | 6945.7(16) | 2864.9(16) | 945.2(12) | 21.9(3) |
| C^12^ | 2279.6(17) | 1812.5(16) | 3493.5(13) | 24.6(3) |
| C^1^ | 6080.9(18) | 3600.4(17) | 5012.1(14) | 27.8(4) |
| C^19^ | 7420.6(17) | 1623.6(16) | 453.9(13) | 24.0(3) |
| C^23^ | 7452.3(17) | 4159.5(16) | 469.8(13) | 23.9(3) |
| C^27^ | 7866.9(17) | 5643.5(16) | 1665.2(13) | 24.0(3) |
| C^11^ | 3818.7(17) | 2062.1(16) | 3216.7(13) | 23.0(3) |
| C^28^ | 8924.1(18) | 4772.4(17) | 1895.2(14) | 26.9(4) |
| C^13^ | 1368.4(18) | 2182.7(17) | 4382.1(14) | 28.5(4) |
| C^32^ | 7549.3(19) | 6727.9(18) | 2180.3(14) | 30.2(4) |
| C^5^ | 5577.6(18) | 1538.7(18) | 6436.3(13) | 27.2(4) |
| C^24^ | 7092.0(17) | 5488.1(16) | 851.6(13) | 24.6(3) |
| C^17^ | 1702.1(19) | 1180.0(18) | 2873.4(15) | 31.4(4) |
| C^29^ | 9630.0(18) | 4960.1(18) | 2615.6(14) | 31.3(4) |
| C^25^ | 7599(2) | 6698.9(18) | -142.3(14) | 33.9(4) |
| C^3^ | 6967.7(18) | 3422(2) | 6525.0(15) | 33.4(4) |
| C^2^ | 6829.4(19) | 4167(2) | 5560.1(15) | 33.9(4) |
| C^26^ | 5474.9(18) | 5592.5(19) | 1312.9(15) | 31.8(4) |
| C^22^ | 8342(2) | 2064.3(18) | -704.4(14) | 32.1(4) |
| C^14^ | -76.8(19) | 1900.8(19) | 4656.3(16) | 35.3(4) |
| C^31^ | 8256.5(19) | 6923.1(19) | 2897.6(15) | 34.0(4) |
| C^21^ | 6115.7(19) | 795.0(19) | 476.7(15) | 33.0(4) |
| C^4^ | 6359.7(19) | 2098.6(19) | 6961.1(15) | 32.1(4) |
| C^16^ | 249(2) | 893.5(19) | 3162.5(16) | 38.0(5) |
| C^30^ | 9299.5(19) | 6038.8(19) | 3116.5(14) | 33.4(4) |
| C^20^ | 8281(2) | 693.8(18) | 1111.3(15) | 33.0(4) |
| C^15^ | -636.2(19) | 1247(2) | 4055.7(17) | 39.0(5) |

| Anisotropic Displacement Parameters (Å^2^×10^3^) for 22019145_0m. The Anisotropic displacement factor exponent takes the form: -2π^2^[h^2^a*^2^U_11_+2hka*b*U_12_+…]. | | | | | | |
| --- | --- | --- | --- | --- | --- | --- |
| Atom | **U_11_** | **U_22_** | **U_33_** | **U_23_** | **U_13_** | U_12_ |
| O^2^ | 27.5(6) | 30.3(6) | 25.1(6) | -3.4(5) | -5.4(5) | -4.1(5) |
| O^1^ | 20.9(5) | 29.5(6) | 20.6(6) | -4.2(5) | -5.7(5) | -2.2(5) |
| C^7^ | 20.5(8) | 22.9(8) | 22.5(8) | -5.9(6) | -4.5(6) | 1.8(6) |
| C^8^ | 20.9(8) | 21.2(7) | 22.5(8) | -4.7(6) | -5.5(6) | -0.3(6) |
| C^10^ | 20.7(8) | 23.3(8) | 24.1(8) | -5.0(6) | -6.9(7) | 0.2(6) |
| C^9^ | 22.5(8) | 23.9(8) | 23.4(8) | -5.7(6) | -6.4(7) | -0.3(6) |
| C^6^ | 21.4(8) | 25.8(8) | 22.3(8) | -8.6(6) | -4.6(6) | 3.7(6) |
| C^18^ | 21.1(8) | 25.7(8) | 20.7(8) | -6.1(6) | -8.1(6) | -0.4(6) |
| C^12^ | 23.1(8) | 22.3(8) | 26.4(9) | 0.5(6) | -8.7(7) | -0.7(6) |
| C^1^ | 29.8(9) | 28.9(9) | 23.8(9) | -7.2(7) | -5.5(7) | 0.0(7) |
| C^19^ | 26.3(8) | 23.4(8) | 22.6(8) | -6.1(6) | -6.5(7) | -2.1(7) |
| C^23^ | 24.1(8) | 26.5(8) | 20.2(8) | -6.2(6) | -4.3(7) | 0.9(7) |
| C^27^ | 22.9(8) | 22.5(8) | 23.5(8) | -4.2(6) | -2.6(7) | -2.8(6) |
| C^11^ | 24.9(8) | 22.5(8) | 20.4(8) | -4.6(6) | -4.8(7) | -0.5(6) |
| C^28^ | 26.8(8) | 21.9(8) | 30.3(9) | -5.2(7) | -5.9(7) | -1.7(7) |
| C^13^ | 25.1(8) | 26.5(8) | 30.8(9) | -2.3(7) | -6.5(7) | 1.2(7) |
| C^32^ | 28.3(9) | 30.4(9) | 32.2(10) | -10.4(7) | -6.8(8) | 3.2(7) |
| C^5^ | 30.1(9) | 27.2(8) | 25.3(9) | -7.8(7) | -8.4(7) | 4.1(7) |
| C^24^ | 26.7(8) | 22.9(8) | 24.1(8) | -6.2(7) | -6.4(7) | 1.7(7) |
| C^17^ | 33.4(9) | 33.2(9) | 29.5(9) | -2.9(7) | -15.0(8) | -3.1(8) |
| C^29^ | 25.5(9) | 31.8(9) | 32.9(10) | -0.5(7) | -8.0(7) | -3.3(7) |
| C^25^ | 47.3(11) | 24.5(9) | 27.5(9) | -4.6(7) | -8.3(8) | 4.1(8) |
| C^3^ | 26.0(9) | 46.8(11) | 32.9(10) | -20.9(9) | -7.6(8) | 0.5(8) |
| C^2^ | 31.9(10) | 35.3(10) | 33.9(10) | -14.1(8) | -2.8(8) | -7.1(8) |
| C^26^ | 29.0(9) | 34.0(9) | 37.0(10) | -15.1(8) | -11.8(8) | 7.2(8) |
| C^22^ | 39.5(10) | 29.3(9) | 26.6(9) | -12.2(7) | -3.3(8) | -0.8(8) |
| C^14^ | 25.4(9) | 32.6(9) | 38.8(11) | 2.0(8) | -3.4(8) | 5.4(8) |
| C^31^ | 33.9(10) | 37.0(10) | 32.7(10) | -16.7(8) | -5.1(8) | -1.1(8) |
| C^21^ | 31.9(9) | 32.3(9) | 38.1(11) | -14.0(8) | -10.0(8) | -3.5(8) |
| C^4^ | 33.3(9) | 40.3(10) | 27.4(9) | -12.4(8) | -13.1(8) | 9.0(8) |
| C^16^ | 36.7(10) | 35.9(10) | 43.7(12) | 2.7(9) | -24.3(9) | -7.6(8) |
| C^30^ | 31.0(9) | 41.1(10) | 27.5(9) | -6.7(8) | -7.7(8) | -7.2(8) |
| C^20^ | 37.7(10) | 29.7(9) | 34.3(10) | -9.6(8) | -13.1(8) | 6.5(8) |
| C^15^ | 21.4(9) | 36.2(10) | 50.4(12) | 11.2(9) | -12.9(9) | -3.4(8) |

| Bond Lengths for 22019145_0m. | | | | | |
| --- | --- | --- | --- | --- | --- |
| Atom | **Atom** | **Length/Å** | **Atom** | **Atom** | **Length/Å** |
| O^2^ | C^7^ | 1.2216(19) | C^19^ | C^20^ | 1.535(2) |
| O^1^ | C^10^ | 1.3862(18) | C^23^ | C^24^ | 1.526(2) |
| O^1^ | C^11^ | 1.3703(19) | C^27^ | C^28^ | 1.392(2) |
| C^7^ | C^8^ | 1.481(2) | C^27^ | C^32^ | 1.397(2) |
| C^7^ | C^6^ | 1.497(2) | C^27^ | C^24^ | 1.536(2) |
| C^8^ | C^9^ | 1.433(2) | C^28^ | C^29^ | 1.388(2) |
| C^8^ | C^11^ | 1.374(2) | C^13^ | C^14^ | 1.382(2) |
| C^10^ | C^9^ | 1.346(2) | C^32^ | C^31^ | 1.388(2) |
| C^10^ | C^18^ | 1.486(2) | C^5^ | C^4^ | 1.384(2) |
| C^6^ | C^1^ | 1.395(2) | C^24^ | C^25^ | 1.546(2) |
| C^6^ | C^5^ | 1.394(2) | C^24^ | C^26^ | 1.544(2) |
| C^18^ | C^19^ | 1.536(2) | C^17^ | C^16^ | 1.389(3) |
| C^18^ | C^23^ | 1.337(2) | C^29^ | C^30^ | 1.381(3) |
| C^12^ | C^11^ | 1.465(2) |  |  |  |

| Bond Angles for 22019145_0m. | | | | | | | |
| --- | --- | --- | --- | --- | --- | --- | --- |
| Atom | **Atom** | **Atom** | **Angle/˚** | **Atom** | **Atom** | **Atom** | Angle/˚ |
| C^11^ | O^1^ | C^10^ | 107.59(12) | C^18^ | C^23^ | C^24^ | 129.59(15) |
| O^2^ | C^7^ | C^8^ | 121.73(14) | C^28^ | C^27^ | C^32^ | 117.42(15) |
| O^2^ | C^7^ | C^6^ | 119.62(14) | C^28^ | C^27^ | C^24^ | 122.91(14) |
| C^8^ | C^7^ | C^6^ | 118.65(14) | C^32^ | C^27^ | C^24^ | 119.63(14) |
| C^9^ | C^8^ | C^7^ | 126.15(14) | O^1^ | C^11^ | C^8^ | 109.33(14) |
| C^11^ | C^8^ | C^7^ | 127.13(15) | O^1^ | C^11^ | C^12^ | 115.68(13) |
| C^11^ | C^8^ | C^9^ | 106.16(14) | C^8^ | C^11^ | C^12^ | 134.99(15) |
| O^1^ | C^10^ | C^18^ | 117.79(13) | C^29^ | C^28^ | C^27^ | 121.56(16) |
| C^9^ | C^10^ | O^1^ | 109.25(14) | C^14^ | C^13^ | C^12^ | 120.58(17) |
| C^9^ | C^10^ | C^18^ | 132.96(15) | C^31^ | C^32^ | C^27^ | 121.21(16) |
| C^10^ | C^9^ | C^8^ | 107.67(14) | C^4^ | C^5^ | C^6^ | 120.35(16) |
| C^1^ | C^6^ | C^7^ | 122.16(15) | C^23^ | C^24^ | C^27^ | 112.03(13) |
| C^5^ | C^6^ | C^7^ | 118.15(15) | C^23^ | C^24^ | C^25^ | 106.67(13) |
| C^5^ | C^6^ | C^1^ | 119.57(15) | C^23^ | C^24^ | C^26^ | 111.74(13) |
| C^10^ | C^18^ | C^19^ | 116.09(13) | C^27^ | C^24^ | C^25^ | 108.26(13) |
| C^23^ | C^18^ | C^10^ | 120.30(14) | C^27^ | C^24^ | C^26^ | 110.51(13) |
| C^23^ | C^18^ | C^19^ | 123.44(14) | C^26^ | C^24^ | C^25^ | 107.38(14) |
| C^13^ | C^12^ | C^11^ | 121.06(15) | C^16^ | C^17^ | C^12^ | 119.98(18) |
| C^17^ | C^12^ | C^11^ | 120.11(16) | C^30^ | C^29^ | C^28^ | 120.09(17) |
| C^17^ | C^12^ | C^13^ | 118.82(16) | C^2^ | C^3^ | C^4^ | 120.40(16) |
| C^2^ | C^1^ | C^6^ | 119.56(16) | C^3^ | C^2^ | C^1^ | 120.41(17) |
| C^22^ | C^19^ | C^18^ | 112.29(13) | C^15^ | C^14^ | C^13^ | 120.30(18) |
| C^22^ | C^19^ | C^21^ | 108.11(14) | C^30^ | C^31^ | C^32^ | 120.27(16) |
| C^22^ | C^19^ | C^20^ | 108.59(14) | C^5^ | C^4^ | C^3^ | 119.66(17) |
| C^21^ | C^19^ | C^18^ | 109.75(13) | C^15^ | C^16^ | C^17^ | 120.41(18) |
| C^21^ | C^19^ | C^20^ | 109.17(14) | C^29^ | C^30^ | C^31^ | 119.44(16) |
| C^20^ | C^19^ | C^18^ | 108.88(13) | C^14^ | C^15^ | C^16^ | 119.87(17) |

| Hydrogen Atom Coordinates (Å×10^4^) and Isotropic Displacement Parameters (Å^2^×10^3^) for 22019145_0m. | | | | |
| --- | --- | --- | --- | --- |
| Atom | ***x*** | ***y*** | ***z*** | **U(eq)** |
| H^9^ | 7015.39 | 2504.88 | 3160.77 | 28 |
| H^1^ | 6010.49 | 4105.94 | 4337.13 | 33 |
| H^23^ | 8138.3 | 4268.48 | -205.92 | 29 |
| H^28^ | 9168.49 | 4031.96 | 1551.01 | 32 |
| H^13^ | 1745.13 | 2632.44 | 4802.26 | 34 |
| H^32^ | 6836.32 | 7342.99 | 2036.87 | 36 |
| H^5^ | 5136.78 | 642.02 | 6743.73 | 33 |
| H^17^ | 2302.92 | 945.42 | 2253.57 | 38 |
| H^29^ | 10341.2 | 4345.86 | 2764.2 | 38 |
| H^25A^ | 8626.34 | 6666.45 | -452.73 | 51 |
| H^25B^ | 7380.82 | 7577.57 | 65.89 | 51 |
| H^25C^ | 7111.88 | 6620.14 | -670.21 | 51 |
| H^3^ | 7482.96 | 3818.71 | 6893.74 | 40 |
| H^2^ | 7248.86 | 5074.38 | 5267.87 | 41 |
| H^26A^ | 4984.8 | 5344.29 | 835.89 | 48 |
| H^26B^ | 5264.07 | 6541.2 | 1377.06 | 48 |
| H^26C^ | 5149.69 | 4958.2 | 2018.6 | 48 |
| H^22A^ | 7817.23 | 2685.37 | -1129.06 | 48 |
| H^22B^ | 8586.08 | 1244.73 | -994.1 | 48 |
| H^22C^ | 9214.06 | 2545.22 | -732.02 | 48 |
| H^14^ | -687.22 | 2159.25 | 5262.14 | 42 |
| H^31^ | 8023.64 | 7666.56 | 3239.73 | 41 |
| H^21A^ | 5548.91 | 438.11 | 1217.98 | 50 |
| H^21B^ | 6423.73 | 20.98 | 142.17 | 50 |
| H^21C^ | 5540.07 | 1396 | 84.62 | 50 |
| H^4^ | 6479.47 | 1578.28 | 7616.94 | 39 |
| H^16^ | -140.02 | 451.87 | 2744.01 | 46 |
| H^30^ | 9783.96 | 6172.5 | 3607.17 | 40 |
| H^20A^ | 9140.13 | 1205.8 | 1071.39 | 50 |
| H^20B^ | 8547.9 | -125.72 | 825.45 | 50 |
| H^20C^ | 7704.56 | 407.41 | 1856.81 | 50 |
| H^15^ | -1628.36 | 1039.28 | 4254.47 | 47 |

| **Fractional Atomic Coordinates (×10^4^) and Equivalent Isotropic Displacement Parameters (Å^2^×10^3^) for ZZ. U_eq_ is defined as 1/3 of of the trace of the orthogonalised U_IJ_ tensor.** | | | | |
| --- | --- | --- | --- | --- |
| **Atom** | ***x*** | ***y*** | ***z*** | ***U(eq)*** |
| O1 | 4698(2) | 4620.4(7) | 4662(4) | 18.4(8) |
| O002 | -226(2) | 5363.6(7) | -731(4) | 18.0(8) |
| O003 | 6347(3) | 1657.6(10) | 10473(5) | 32.5(10) |
| O004 | 11314(3) | 1685.6(10) | 10542(5) | 33.2(10) |
| C15 | 6546(3) | 4068.7(10) | 3863(5) | 20.5(11) |
| C006 | -119(3) | 5215.4(11) | 403(5) | 16.2(10) |
| C8 | 4851(3) | 4781.9(11) | 5787(5) | 18.4(10) |
| C4 | 4706(4) | 5243.0(12) | 6438(6) | 24.5(12) |
| C009 | 731(3) | 5980.4(10) | -869(5) | 19.7(10) |
| C14 | 6011(3) | 3890.1(10) | 3943(5) | 19.1(10) |
| C00B | 13(3) | 5636.0(10) | -234(5) | 15.5(10) |
| O00C | -1324(3) | 4176.0(11) | -752(5) | 34.5(10) |
| C00D | 2067(3) | 5462.4(11) | 4352(5) | 22.8(11) |
| C13 | 5487(3) | 3984.3(10) | 4357(5) | 18.1(10) |
| C00F | 1503(3) | 5580.5(11) | 4594(6) | 21.6(11) |
| C00G | -767(3) | 5802.3(9) | -993(5) | 12.8(9) |
| C6 | 4117(3) | 5128.9(12) | 3965(6) | 24.3(12) |
| C19 | 3573(3) | 4187.2(10) | 3048(5) | 19.1(10) |
| C18 | 4168(3) | 4197.3(9) | 4706(4) | 14.1(9) |
| C00K | -855(3) | 4851.1(12) | -1347(5) | 21.8(11) |
| C11 | 4949(3) | 4355.1(10) | 5213(5) | 16.6(10) |
| C00M | -290(4) | 4770.9(13) | 1148(6) | 25.1(12) |
| O2 | 3748(3) | 5831.0(10) | 4359(5) | 35.1(10) |
| C00O | 232(3) | 5352.8(11) | 1628(5) | 18.6(11) |
| C9 | 5266(3) | 4661.2(11) | 7073(6) | 22.1(11) |
| C12 | 5491(3) | 4246.6(10) | 4708(5) | 16.3(10) |
| C00R | -1460(3) | 5698.1(10) | -787(5) | 20.0(10) |
| C5 | 4553(3) | 5053.4(11) | 5387(5) | 18.0(11) |
| C16 | 6551(3) | 4326.9(11) | 4213(6) | 21.0(11) |
| C24 | 6605(3) | 4453.4(11) | 10061(6) | 22.9(11) |
| C00V | 385(3) | 5622.0(11) | 1381(5) | 18.8(11) |
| C00W | 1876(3) | 5862.6(11) | -1061(6) | 29.4(12) |
| C7 | 3835(4) | 5389.8(13) | 3577(6) | 28.8(13) |
| C17 | 6041(3) | 4419.4(11) | 4652(6) | 19.0(11) |
| C00Z | 656(3) | 5723.9(11) | -567(5) | 16.5(10) |
| C010 | -422(3) | 4944.6(11) | 65(5) | 18.7(11) |
| C011 | 1339(3) | 6058.2(12) | -1110(6) | 27.7(12) |
| C22 | 7006(3) | 3985.1(12) | 9995(5) | 27.4(12) |
| C013 | -1166(4) | 4594.9(13) | -1665(6) | 28.7(13) |
| C23 | 5642(3) | 4072.7(11) | 9664(5) | 26.0(12) |
| C015 | 1107(3) | 5844.9(10) | 3854(5) | 18.3(10) |
| C016 | 748(3) | 5826.8(11) | 2239(5) | 18.8(10) |
| C017 | 420(3) | 5927.8(11) | 4081(5) | 22.1(11) |
| C20 | 5869(3) | 4193.4(11) | 7767(5) | 23.7(11) |
| C019 | 1771(3) | 6059.6(11) | 4492(6) | 25.9(12) |
| O01A | 10295(3) | 2887.7(9) | 10340(5) | 46.5(12) |
| O01B | 5305(3) | 2851.9(9) | 10351(5) | 46.3(12) |
| C10 | 5436(3) | 4388.8(10) | 6871(5) | 16.1(10) |
| C28 | 6644(4) | 4831.5(13) | 11450(7) | 35.9(15) |
| C01E | 2236(4) | 5098.5(12) | 5877(7) | 32.2(14) |
| C25 | 7146(4) | 4583.3(12) | 9812(6) | 29.3(13) |
| C01G | 2442(4) | 5224.1(12) | 4983(6) | 29.3(13) |
| C01H | 1204(3) | 5530.1(11) | -504(6) | 22.1(11) |
| C01I | 1693(4) | 5212.2(12) | 6144(6) | 30.1(13) |
| C01J | 1317(4) | 5456.1(12) | 5496(6) | 26.5(13) |
| C29 | 6361(4) | 4577.8(13) | 10899(6) | 29.0(13) |
| C01L | 1817(3) | 5604.0(12) | -753(6) | 29.5(13) |
| C21 | 6282(3) | 4179.7(11) | 9387(5) | 20.3(11) |
| C01N | 6089(4) | 1913.8(12) | 10272(7) | 25.9(12) |
| C27 | 7171(4) | 4958.9(13) | 11177(8) | 41.4(16) |
| C2 | 3999(4) | 5570.3(14) | 4627(7) | 29.3(13) |
| C01Q | -603(4) | 4519.4(13) | 834(7) | 27.7(13) |
| C01R | 5619(4) | 1995.3(14) | 8843(7) | 32.1(14) |
| C3 | 4432(4) | 5498.7(13) | 6047(6) | 26.9(13) |
| C01T | -1043(4) | 4432.0(13) | -570(7) | 29.1(13) |
| C01U | 11059(4) | 1943.3(13) | 10320(7) | 29.2(13) |
| C01V | 10617(4) | 2025.7(14) | 8883(7) | 33.0(14) |
| C01W | 6835(5) | 1566.2(15) | 11916(8) | 41.1(16) |
| C26 | 7438(4) | 4836.9(14) | 10352(7) | 39.9(16) |
| C01Y | 8697(4) | 2950.9(14) | 4104(7) | 38.4(15) |
| C01Z | 11782(5) | 1595.6(15) | 11988(8) | 40.9(16) |
| C020 | 9637(4) | 3132.8(13) | 8204(8) | 39.2(15) |
| C021 | 9223(4) | 3335.5(13) | 7269(7) | 35.6(14) |
| C022 | 3685(4) | 2922.7(15) | 4106(8) | 43.8(16) |
| C023 | 5476(4) | 2429.7(14) | 9591(7) | 37.6(15) |
| C024 | 6227(4) | 2090.1(13) | 11346(7) | 31.2(14) |
| C025 | 10465(4) | 2464.6(14) | 9594(7) | 36.6(15) |
| C026 | 8915(5) | 3075.0(14) | 10236(7) | 45.3(17) |
| C027 | 3923(5) | 3043.7(16) | 10243(8) | 50.7(18) |
| C028 | -1840(4) | 4079.8(14) | -2166(7) | 37.6(16) |
| C029 | 4651(4) | 3100.4(14) | 8231(8) | 45.1(16) |
| C02A | 11182(4) | 2121.1(13) | 11370(7) | 32.0(14) |
| C02B | 10881(4) | 2378.1(14) | 10994(7) | 36.3(14) |
| C02C | 4800(4) | 2832.2(14) | 7973(8) | 39.0(15) |
| C02D | 8843(4) | 3344.9(14) | 5690(8) | 42.5(15) |
| C02E | 4245(4) | 3305.8(14) | 7309(8) | 42.2(15) |
| C02F | 7590(5) | 2713.3(15) | 4613(9) | 51.3(18) |
| C02G | 5327(4) | 2251.9(14) | 8509(7) | 33.5(14) |
| C02H | 10337(4) | 2281.7(14) | 8528(7) | 33.6(14) |
| C02I | 3846(4) | 3314.2(14) | 5708(8) | 45.1(16) |
| C02J | 8483(4) | 3082.1(13) | 4970(7) | 36.2(14) |
| C02K | 9790(4) | 2864.7(14) | 7949(8) | 38.9(15) |
| C02L | 8374(5) | 2708.9(16) | 3514(8) | 49.0(18) |
| C02M | 4479(5) | 3218.9(14) | 10237(7) | 49.9(17) |
| C02N | 9472(4) | 3251.3(14) | 10236(7) | 48.1(17) |
| C1 | 3309(5) | 5919.2(14) | 2917(8) | 40.0(16) |
| C02P | 8373(5) | 3149.5(15) | 10611(8) | 51.2(18) |
| C02Q | 5921(4) | 2345.4(14) | 10990(7) | 34.9(14) |
| C02R | 10174(4) | 2737.2(14) | 9200(8) | 41.3(15) |
| C02S | 7910(4) | 2955.8(15) | 5199(8) | 46.8(17) |
| C02T | 8160(5) | 3556.0(15) | 5057(8) | 52.4(19) |
| C02U | 9510(5) | 3446.6(15) | 5416(9) | 55(2) |
| C02V | 3359(5) | 3124.6(17) | 10555(8) | 60(2) |
| C02W | 5175(4) | 2702.0(14) | 9212(8) | 40.6(15) |
| C02X | 10048(4) | 3160.9(13) | 9802(8) | 47.6(17) |
| C02Y | 3358(5) | 2680.1(18) | 3521(9) | 57(2) |
| C02Z | 3486(4) | 3050.9(15) | 5006(8) | 43.2(16) |
| C030 | 3159(5) | 3525.2(15) | 5110(9) | 57(2) |
| C031 | 2602(5) | 2682.1(17) | 4662(10) | 59(2) |
| C032 | 4499(5) | 3416.2(16) | 5409(10) | 59(2) |
| C033 | 2924(5) | 2924.3(16) | 5259(9) | 52.6(19) |
| C034 | 7815(5) | 2586.9(17) | 3778(10) | 57(2) |
| C035 | 5056(4) | 3125.1(14) | 9837(8) | 52.4(18) |
| C036 | 2821(5) | 2555.6(18) | 3824(10) | 63(2) |
| C037 | 9439(5) | 3515.6(15) | 10591(8) | 55.3(19) |
| C038 | 8887(5) | 3591.4(18) | 10959(9) | 66(2) |
| C039 | 4428(6) | 3485.9(16) | 10530(9) | 70(2) |
| C03A | 8368(6) | 3416.1(17) | 10986(8) | 64(2) |
| C03B | 10846(5) | 3321.6(16) | 10451(10) | 79(2) |
| C03C | 3852(6) | 3563.2(19) | 10834(10) | 82(3) |
| C03D | 3325(6) | 3394(2) | 10845(10) | 78(3) |
| C03E | 5860(6) | 3281.5(17) | 10466(12) | 92(3) |
| C0AA | 6450(9) | 3292(3) | 11756(16) | 62(4) |
| C1AA | 11454(9) | 3326(3) | 11753(16) | 64(4) |
| C2AA | 11470(9) | 3214(3) | 10516(17) | 61(4) |
| C3AA | 6492(9) | 3181(3) | 10596(17) | 62(4) |

| **Anisotropic Displacement Parameters (Å^2^×10^3^) for ZZ. The Anisotropic displacement factor exponent takes the form: -2π^2^[h^2^a*^2^U_11_+2hka*b*U_12_+…].** | | | | | | |
| --- | --- | --- | --- | --- | --- | --- |
| **Atom** | **U_11_** | **U_22_** | **U_33_** | **U_23_** | **U_13_** | **U_12_** |
| O1 | 29.8(19) | 20.0(18) | 12.0(16) | -0.1(14) | 15.7(15) | 2.4(14) |
| O002 | 25.6(18) | 20.6(18) | 15.7(16) | -0.1(14) | 16.3(15) | -0.8(14) |
| O003 | 40(3) | 20(2) | 36(2) | 2.3(19) | 19(2) | 10.7(19) |
| O004 | 38(3) | 21(2) | 36(2) | 2.2(19) | 16(2) | 11.5(19) |
| C15 | 15(2) | 29(2) | 23(2) | 4.7(19) | 13.6(19) | -4.6(19) |
| C006 | 22(2) | 21(2) | 13(2) | 5.7(18) | 14.0(19) | 3.2(19) |
| C8 | 20(2) | 22(2) | 17(2) | -2.2(19) | 12.6(19) | -0.3(19) |
| C4 | 27(3) | 28(3) | 19(2) | -2(2) | 13(2) | 0(2) |
| C009 | 21(2) | 23(2) | 25(2) | -3.5(19) | 18(2) | -4.4(19) |
| C14 | 22(2) | 17(2) | 23(2) | 2.6(18) | 15.7(19) | 2.9(18) |
| C00B | 19(2) | 19(2) | 14(2) | -1.5(17) | 13.2(18) | -1.8(18) |
| O00C | 37(2) | 32(2) | 29(2) | -3(2) | 14(2) | -3(2) |
| C00D | 20(2) | 27(3) | 16(2) | 0(2) | 5(2) | -4(2) |
| C13 | 20(2) | 21(2) | 19(2) | 1.9(18) | 14.3(19) | -3.3(18) |
| C00F | 23(2) | 18(2) | 16(2) | 0.3(19) | 6(2) | -5.0(19) |
| C00G | 13(2) | 15(2) | 12.1(19) | 2.6(16) | 8.1(17) | 4.5(16) |
| C6 | 26(3) | 30(3) | 20(2) | 1(2) | 15(2) | 2(2) |
| C19 | 13(2) | 26(2) | 14(2) | -6.7(19) | 4.1(18) | -7.3(19) |
| C18 | 12(2) | 21(2) | 12.4(19) | -0.2(16) | 8.6(16) | 2.2(16) |
| C00K | 28(3) | 26(3) | 15(2) | 3(2) | 14(2) | 1(2) |
| C11 | 25(2) | 19(2) | 13(2) | -1.0(17) | 14.5(19) | 0.8(18) |
| C00M | 28(3) | 31(3) | 16(2) | 3(2) | 12(2) | -1(2) |
| O2 | 38(3) | 30(2) | 32(2) | 4(2) | 15(2) | 2(2) |
| C00O | 24(2) | 23(2) | 11(2) | -0.4(18) | 10.6(19) | -3.0(19) |
| C9 | 27(3) | 25(2) | 16(2) | -2.8(19) | 13(2) | 0(2) |
| C12 | 17(2) | 21(2) | 15(2) | 4.7(18) | 10.9(18) | 1.0(18) |
| C00R | 10(2) | 23(2) | 26(2) | 3(2) | 8.8(19) | -0.2(19) |
| C5 | 21(2) | 23(2) | 17(2) | -1.8(19) | 15(2) | -2.0(19) |
| C16 | 16(2) | 23(2) | 26(2) | 6(2) | 13(2) | -4.1(19) |
| C24 | 24(2) | 25(2) | 15(2) | 0.9(19) | 7(2) | 5(2) |
| C00V | 21(2) | 26(2) | 15(2) | -0.2(18) | 13.1(19) | 2.1(19) |
| C00W | 22(2) | 42(3) | 34(3) | -7(2) | 23(2) | -8(2) |
| C7 | 34(3) | 32(3) | 20(2) | 4(2) | 13(2) | -1(2) |
| C17 | 18(2) | 19(2) | 23(2) | 2.6(19) | 13(2) | -6.9(19) |
| C00Z | 16(2) | 26(2) | 14(2) | -0.9(18) | 12.8(18) | 4.9(18) |
| C010 | 20(2) | 22(2) | 17(2) | -3.4(19) | 12(2) | -0.4(19) |
| C011 | 29(3) | 29(3) | 34(3) | -3(2) | 23(2) | -2(2) |
| C22 | 31(3) | 29(3) | 13(2) | -2(2) | 6(2) | 7(2) |
| C013 | 33(3) | 28(3) | 23(3) | -6(2) | 14(2) | 3(2) |
| C23 | 32(3) | 27(3) | 13(2) | 1(2) | 8(2) | -5(2) |
| C015 | 26(2) | 18(2) | 10(2) | 3.6(18) | 8.9(19) | 1.6(19) |
| C016 | 26(2) | 21(2) | 11(2) | 1.4(18) | 10.4(19) | 1.2(19) |
| C017 | 28(3) | 21(2) | 13(2) | -1.4(19) | 8(2) | 3(2) |
| C20 | 30(3) | 22(2) | 17(2) | -0.8(19) | 12(2) | 3(2) |
| C019 | 28(3) | 21(3) | 18(2) | 1(2) | 4(2) | -6(2) |
| O01A | 42(2) | 24(2) | 49(3) | 2(2) | 8(2) | 5.6(19) |
| O01B | 38(2) | 26(2) | 56(3) | 1(2) | 12(2) | 4.2(19) |
| C10 | 21(2) | 22(2) | 7.6(19) | -1.4(17) | 8.8(18) | -2.7(18) |
| C28 | 28(3) | 31(3) | 37(3) | -11(2) | 10(2) | 10(2) |
| C01E | 26(3) | 21(3) | 35(3) | 7(2) | 6(2) | -2(2) |
| C25 | 27(3) | 34(3) | 26(3) | -4(2) | 13(2) | 0(2) |
| C01G | 24(3) | 26(3) | 30(3) | -1(2) | 8(2) | -1(2) |
| C01H | 21(2) | 26(3) | 23(2) | -6(2) | 15(2) | 1(2) |
| C01I | 33(3) | 27(3) | 25(3) | 8(2) | 11(2) | -4(2) |
| C01J | 28(3) | 28(3) | 20(2) | 4(2) | 10(2) | -1(2) |
| C29 | 33(3) | 33(3) | 19(2) | -3(2) | 12(2) | 3(2) |
| C01L | 22(2) | 40(3) | 33(3) | -10(2) | 19(2) | 3(2) |
| C21 | 24(2) | 24(2) | 8(2) | 1.7(18) | 5.2(18) | 3(2) |
| C01N | 28(3) | 15(2) | 36(3) | 3(2) | 18(2) | 3(2) |
| C27 | 28(3) | 28(3) | 47(3) | -10(2) | 6(2) | 2(2) |
| C2 | 27(3) | 30(3) | 29(3) | 0(2) | 14(2) | 1(2) |
| C01Q | 32(3) | 26(3) | 27(3) | 6(2) | 17(2) | -3(2) |
| C01R | 32(3) | 28(3) | 36(3) | -1(2) | 18(2) | -1(2) |
| C3 | 28(3) | 28(3) | 25(3) | -4(2) | 15(2) | 2(2) |
| C01T | 30(3) | 28(3) | 30(3) | 1(2) | 17(2) | 0(2) |
| C01U | 23(3) | 24(3) | 40(3) | 1(2) | 15(2) | -2(2) |
| C01V | 34(3) | 27(3) | 37(3) | 1(2) | 18(2) | 1(2) |
| C01W | 48(4) | 38(4) | 35(3) | 6(3) | 20(3) | 7(3) |
| C26 | 29(3) | 41(3) | 43(3) | 0(3) | 14(3) | -3(2) |
| C01Y | 30(3) | 33(3) | 43(3) | 4(3) | 13(3) | 6(2) |
| C01Z | 44(4) | 35(4) | 36(3) | 2(3) | 15(3) | 8(3) |
| C020 | 32(3) | 28(3) | 54(3) | -2(2) | 21(3) | -2(2) |
| C021 | 30(3) | 29(3) | 48(3) | -1(2) | 21(2) | 0(2) |
| C022 | 32(3) | 39(3) | 52(4) | 8(3) | 17(3) | 4(3) |
| C023 | 29(3) | 30(3) | 45(3) | -1(2) | 15(3) | 0(2) |
| C024 | 27(3) | 30(3) | 34(3) | 1(2) | 15(2) | 4(2) |
| C025 | 30(3) | 32(3) | 41(3) | -3(2) | 14(3) | 2(2) |
| C026 | 51(4) | 36(3) | 35(3) | -2(3) | 13(3) | 13(3) |
| C027 | 51(4) | 46(4) | 40(3) | -3(3) | 15(3) | 17(3) |
| C028 | 36(3) | 32(3) | 36(3) | -10(3) | 14(3) | -8(3) |
| C029 | 33(3) | 35(3) | 61(4) | 7(3) | 21(3) | 3(2) |
| C02A | 27(3) | 28(3) | 34(3) | -5(2) | 11(2) | 3(2) |
| C02B | 34(3) | 28(3) | 37(3) | -6(2) | 12(2) | -3(2) |
| C02C | 31(3) | 32(3) | 51(3) | 2(3) | 20(3) | 5(2) |
| C02D | 40(3) | 34(3) | 55(3) | 3(3) | 27(3) | 5(2) |
| C02E | 32(3) | 34(3) | 59(3) | 2(3) | 24(3) | 2(2) |
| C02F | 35(3) | 40(3) | 69(4) | 9(3) | 21(3) | -1(3) |
| C02G | 31(3) | 30(3) | 36(3) | 4(2) | 16(2) | -2(2) |
| C02H | 32(3) | 31(3) | 38(3) | 4(2) | 19(2) | 2(2) |
| C02I | 37(3) | 35(3) | 66(4) | 10(3) | 30(3) | 10(2) |
| C02J | 31(3) | 31(3) | 41(3) | 8(2) | 16(2) | 4(2) |
| C02K | 34(3) | 32(3) | 45(3) | 0(2) | 17(3) | 1(2) |
| C02L | 40(3) | 46(4) | 45(3) | -1(3) | 12(3) | 7(3) |
| C02M | 52(3) | 32(3) | 38(3) | -7(3) | 5(3) | 12(3) |
| C02N | 51(3) | 36(3) | 38(3) | -2(3) | 11(3) | 10(3) |
| C1 | 41(4) | 28(3) | 41(4) | 9(3) | 15(3) | 6(3) |
| C02P | 53(4) | 48(3) | 41(3) | 4(3) | 17(3) | 12(3) |
| C02Q | 33(3) | 29(3) | 39(3) | -3(2) | 16(2) | 0(2) |
| C02R | 28(3) | 33(3) | 52(3) | -2(3) | 14(3) | 1(2) |
| C02S | 35(3) | 40(3) | 60(4) | 10(3) | 22(3) | 6(3) |
| C02T | 63(4) | 37(4) | 59(4) | 10(3) | 34(4) | 9(3) |
| C02U | 61(4) | 39(4) | 86(5) | -4(4) | 52(4) | -6(3) |
| C02V | 67(4) | 64(4) | 43(3) | 3(3) | 27(3) | 23(3) |
| C02W | 32(3) | 32(3) | 48(3) | -3(3) | 15(3) | 1(2) |
| C02X | 42(3) | 22(3) | 57(3) | 0(3) | 12(3) | 1(2) |
| C02Y | 43(4) | 54(4) | 57(4) | 4(3) | 15(3) | 8(3) |
| C02Z | 34(3) | 42(3) | 51(3) | 16(3) | 21(3) | 4(3) |
| C030 | 58(4) | 44(4) | 81(5) | 24(4) | 45(4) | 15(3) |
| C031 | 40(3) | 53(4) | 74(4) | 17(3) | 24(3) | -4(3) |
| C032 | 59(4) | 43(4) | 96(6) | 4(4) | 57(4) | 5(3) |
| C033 | 38(3) | 48(4) | 66(4) | 19(3) | 24(3) | 3(3) |
| C034 | 40(3) | 39(3) | 63(4) | -2(3) | 9(3) | -3(3) |
| C035 | 42(3) | 27(3) | 63(4) | 3(3) | 11(3) | 6(3) |
| C036 | 45(4) | 50(4) | 67(4) | 5(3) | 11(3) | -6(3) |
| C037 | 59(4) | 37(3) | 50(4) | -6(3) | 15(3) | 9(3) |
| C038 | 77(4) | 51(4) | 49(4) | -6(3) | 20(3) | 22(3) |
| C039 | 78(4) | 42(4) | 59(4) | -10(3) | 16(3) | 14(3) |
| C03A | 78(4) | 60(4) | 44(4) | -1(3) | 26(3) | 23(3) |
| C03B | 56(4) | 51(4) | 97(5) | -2(4) | 19(3) | -5(3) |
| C03C | 102(5) | 61(4) | 56(4) | -14(3) | 24(4) | 32(4) |
| C03D | 89(5) | 82(5) | 58(4) | -2(4) | 35(4) | 45(4) |
| C03E | 61(4) | 50(4) | 117(6) | 2(4) | 14(4) | -1(3) |
| C0AA | 64(7) | 68(8) | 57(7) | -10(6) | 34(6) | -12(6) |
| C1AA | 59(7) | 78(9) | 50(7) | 12(7) | 26(5) | 9(7) |
| C2AA | 62(7) | 58(8) | 64(8) | -5(6) | 34(7) | -6(6) |
| C3AA | 62(7) | 69(8) | 66(8) | 11(7) | 42(7) | -12(6) |

| **Anisotropic Displacement Parameters (Å^2^×10^3^) for ZZ. The Anisotropic displacement factor exponent takes the form: -2π^2^[h^2^a*^2^U_11_+2hka*b*U_12_+…].** | | | | | | |
| --- | --- | --- | --- | --- | --- | --- |
| **Atom** | **U_11_** | **U_22_** | **U_33_** | **U_23_** | **U_13_** | **U_12_** |
| O1 | 29.8(19) | 20.0(18) | 12.0(16) | -0.1(14) | 15.7(15) | 2.4(14) |
| O002 | 25.6(18) | 20.6(18) | 15.7(16) | -0.1(14) | 16.3(15) | -0.8(14) |
| O003 | 40(3) | 20(2) | 36(2) | 2.3(19) | 19(2) | 10.7(19) |
| O004 | 38(3) | 21(2) | 36(2) | 2.2(19) | 16(2) | 11.5(19) |
| C15 | 15(2) | 29(2) | 23(2) | 4.7(19) | 13.6(19) | -4.6(19) |
| C006 | 22(2) | 21(2) | 13(2) | 5.7(18) | 14.0(19) | 3.2(19) |
| C8 | 20(2) | 22(2) | 17(2) | -2.2(19) | 12.6(19) | -0.3(19) |
| C4 | 27(3) | 28(3) | 19(2) | -2(2) | 13(2) | 0(2) |
| C009 | 21(2) | 23(2) | 25(2) | -3.5(19) | 18(2) | -4.4(19) |
| C14 | 22(2) | 17(2) | 23(2) | 2.6(18) | 15.7(19) | 2.9(18) |
| C00B | 19(2) | 19(2) | 14(2) | -1.5(17) | 13.2(18) | -1.8(18) |
| O00C | 37(2) | 32(2) | 29(2) | -3(2) | 14(2) | -3(2) |
| C00D | 20(2) | 27(3) | 16(2) | 0(2) | 5(2) | -4(2) |
| C13 | 20(2) | 21(2) | 19(2) | 1.9(18) | 14.3(19) | -3.3(18) |
| C00F | 23(2) | 18(2) | 16(2) | 0.3(19) | 6(2) | -5.0(19) |
| C00G | 13(2) | 15(2) | 12.1(19) | 2.6(16) | 8.1(17) | 4.5(16) |
| C6 | 26(3) | 30(3) | 20(2) | 1(2) | 15(2) | 2(2) |
| C19 | 13(2) | 26(2) | 14(2) | -6.7(19) | 4.1(18) | -7.3(19) |
| C18 | 12(2) | 21(2) | 12.4(19) | -0.2(16) | 8.6(16) | 2.2(16) |
| C00K | 28(3) | 26(3) | 15(2) | 3(2) | 14(2) | 1(2) |
| C11 | 25(2) | 19(2) | 13(2) | -1.0(17) | 14.5(19) | 0.8(18) |
| C00M | 28(3) | 31(3) | 16(2) | 3(2) | 12(2) | -1(2) |
| O2 | 38(3) | 30(2) | 32(2) | 4(2) | 15(2) | 2(2) |
| C00O | 24(2) | 23(2) | 11(2) | -0.4(18) | 10.6(19) | -3.0(19) |
| C9 | 27(3) | 25(2) | 16(2) | -2.8(19) | 13(2) | 0(2) |
| C12 | 17(2) | 21(2) | 15(2) | 4.7(18) | 10.9(18) | 1.0(18) |
| C00R | 10(2) | 23(2) | 26(2) | 3(2) | 8.8(19) | -0.2(19) |
| C5 | 21(2) | 23(2) | 17(2) | -1.8(19) | 15(2) | -2.0(19) |
| C16 | 16(2) | 23(2) | 26(2) | 6(2) | 13(2) | -4.1(19) |
| C24 | 24(2) | 25(2) | 15(2) | 0.9(19) | 7(2) | 5(2) |
| C00V | 21(2) | 26(2) | 15(2) | -0.2(18) | 13.1(19) | 2.1(19) |
| C00W | 22(2) | 42(3) | 34(3) | -7(2) | 23(2) | -8(2) |
| C7 | 34(3) | 32(3) | 20(2) | 4(2) | 13(2) | -1(2) |
| C17 | 18(2) | 19(2) | 23(2) | 2.6(19) | 13(2) | -6.9(19) |
| C00Z | 16(2) | 26(2) | 14(2) | -0.9(18) | 12.8(18) | 4.9(18) |
| C010 | 20(2) | 22(2) | 17(2) | -3.4(19) | 12(2) | -0.4(19) |
| C011 | 29(3) | 29(3) | 34(3) | -3(2) | 23(2) | -2(2) |
| C22 | 31(3) | 29(3) | 13(2) | -2(2) | 6(2) | 7(2) |
| C013 | 33(3) | 28(3) | 23(3) | -6(2) | 14(2) | 3(2) |
| C23 | 32(3) | 27(3) | 13(2) | 1(2) | 8(2) | -5(2) |
| C015 | 26(2) | 18(2) | 10(2) | 3.6(18) | 8.9(19) | 1.6(19) |
| C016 | 26(2) | 21(2) | 11(2) | 1.4(18) | 10.4(19) | 1.2(19) |
| C017 | 28(3) | 21(2) | 13(2) | -1.4(19) | 8(2) | 3(2) |
| C20 | 30(3) | 22(2) | 17(2) | -0.8(19) | 12(2) | 3(2) |
| C019 | 28(3) | 21(3) | 18(2) | 1(2) | 4(2) | -6(2) |
| O01A | 42(2) | 24(2) | 49(3) | 2(2) | 8(2) | 5.6(19) |
| O01B | 38(2) | 26(2) | 56(3) | 1(2) | 12(2) | 4.2(19) |
| C10 | 21(2) | 22(2) | 7.6(19) | -1.4(17) | 8.8(18) | -2.7(18) |
| C28 | 28(3) | 31(3) | 37(3) | -11(2) | 10(2) | 10(2) |
| C01E | 26(3) | 21(3) | 35(3) | 7(2) | 6(2) | -2(2) |
| C25 | 27(3) | 34(3) | 26(3) | -4(2) | 13(2) | 0(2) |
| C01G | 24(3) | 26(3) | 30(3) | -1(2) | 8(2) | -1(2) |
| C01H | 21(2) | 26(3) | 23(2) | -6(2) | 15(2) | 1(2) |
| C01I | 33(3) | 27(3) | 25(3) | 8(2) | 11(2) | -4(2) |
| C01J | 28(3) | 28(3) | 20(2) | 4(2) | 10(2) | -1(2) |
| C29 | 33(3) | 33(3) | 19(2) | -3(2) | 12(2) | 3(2) |
| C01L | 22(2) | 40(3) | 33(3) | -10(2) | 19(2) | 3(2) |
| C21 | 24(2) | 24(2) | 8(2) | 1.7(18) | 5.2(18) | 3(2) |
| C01N | 28(3) | 15(2) | 36(3) | 3(2) | 18(2) | 3(2) |
| C27 | 28(3) | 28(3) | 47(3) | -10(2) | 6(2) | 2(2) |
| C2 | 27(3) | 30(3) | 29(3) | 0(2) | 14(2) | 1(2) |
| C01Q | 32(3) | 26(3) | 27(3) | 6(2) | 17(2) | -3(2) |
| C01R | 32(3) | 28(3) | 36(3) | -1(2) | 18(2) | -1(2) |
| C3 | 28(3) | 28(3) | 25(3) | -4(2) | 15(2) | 2(2) |
| C01T | 30(3) | 28(3) | 30(3) | 1(2) | 17(2) | 0(2) |
| C01U | 23(3) | 24(3) | 40(3) | 1(2) | 15(2) | -2(2) |
| C01V | 34(3) | 27(3) | 37(3) | 1(2) | 18(2) | 1(2) |
| C01W | 48(4) | 38(4) | 35(3) | 6(3) | 20(3) | 7(3) |
| C26 | 29(3) | 41(3) | 43(3) | 0(3) | 14(3) | -3(2) |
| C01Y | 30(3) | 33(3) | 43(3) | 4(3) | 13(3) | 6(2) |
| C01Z | 44(4) | 35(4) | 36(3) | 2(3) | 15(3) | 8(3) |
| C020 | 32(3) | 28(3) | 54(3) | -2(2) | 21(3) | -2(2) |
| C021 | 30(3) | 29(3) | 48(3) | -1(2) | 21(2) | 0(2) |
| C022 | 32(3) | 39(3) | 52(4) | 8(3) | 17(3) | 4(3) |
| C023 | 29(3) | 30(3) | 45(3) | -1(2) | 15(3) | 0(2) |
| C024 | 27(3) | 30(3) | 34(3) | 1(2) | 15(2) | 4(2) |
| C025 | 30(3) | 32(3) | 41(3) | -3(2) | 14(3) | 2(2) |
| C026 | 51(4) | 36(3) | 35(3) | -2(3) | 13(3) | 13(3) |
| C027 | 51(4) | 46(4) | 40(3) | -3(3) | 15(3) | 17(3) |
| C028 | 36(3) | 32(3) | 36(3) | -10(3) | 14(3) | -8(3) |
| C029 | 33(3) | 35(3) | 61(4) | 7(3) | 21(3) | 3(2) |
| C02A | 27(3) | 28(3) | 34(3) | -5(2) | 11(2) | 3(2) |
| C02B | 34(3) | 28(3) | 37(3) | -6(2) | 12(2) | -3(2) |
| C02C | 31(3) | 32(3) | 51(3) | 2(3) | 20(3) | 5(2) |
| C02D | 40(3) | 34(3) | 55(3) | 3(3) | 27(3) | 5(2) |
| C02E | 32(3) | 34(3) | 59(3) | 2(3) | 24(3) | 2(2) |
| C02F | 35(3) | 40(3) | 69(4) | 9(3) | 21(3) | -1(3) |
| C02G | 31(3) | 30(3) | 36(3) | 4(2) | 16(2) | -2(2) |
| C02H | 32(3) | 31(3) | 38(3) | 4(2) | 19(2) | 2(2) |
| C02I | 37(3) | 35(3) | 66(4) | 10(3) | 30(3) | 10(2) |
| C02J | 31(3) | 31(3) | 41(3) | 8(2) | 16(2) | 4(2) |
| C02K | 34(3) | 32(3) | 45(3) | 0(2) | 17(3) | 1(2) |
| C02L | 40(3) | 46(4) | 45(3) | -1(3) | 12(3) | 7(3) |
| C02M | 52(3) | 32(3) | 38(3) | -7(3) | 5(3) | 12(3) |
| C02N | 51(3) | 36(3) | 38(3) | -2(3) | 11(3) | 10(3) |
| C1 | 41(4) | 28(3) | 41(4) | 9(3) | 15(3) | 6(3) |
| C02P | 53(4) | 48(3) | 41(3) | 4(3) | 17(3) | 12(3) |
| C02Q | 33(3) | 29(3) | 39(3) | -3(2) | 16(2) | 0(2) |
| C02R | 28(3) | 33(3) | 52(3) | -2(3) | 14(3) | 1(2) |
| C02S | 35(3) | 40(3) | 60(4) | 10(3) | 22(3) | 6(3) |
| C02T | 63(4) | 37(4) | 59(4) | 10(3) | 34(4) | 9(3) |
| C02U | 61(4) | 39(4) | 86(5) | -4(4) | 52(4) | -6(3) |
| C02V | 67(4) | 64(4) | 43(3) | 3(3) | 27(3) | 23(3) |
| C02W | 32(3) | 32(3) | 48(3) | -3(3) | 15(3) | 1(2) |
| C02X | 42(3) | 22(3) | 57(3) | 0(3) | 12(3) | 1(2) |
| C02Y | 43(4) | 54(4) | 57(4) | 4(3) | 15(3) | 8(3) |
| C02Z | 34(3) | 42(3) | 51(3) | 16(3) | 21(3) | 4(3) |
| C030 | 58(4) | 44(4) | 81(5) | 24(4) | 45(4) | 15(3) |
| C031 | 40(3) | 53(4) | 74(4) | 17(3) | 24(3) | -4(3) |
| C032 | 59(4) | 43(4) | 96(6) | 4(4) | 57(4) | 5(3) |
| C033 | 38(3) | 48(4) | 66(4) | 19(3) | 24(3) | 3(3) |
| C034 | 40(3) | 39(3) | 63(4) | -2(3) | 9(3) | -3(3) |
| C035 | 42(3) | 27(3) | 63(4) | 3(3) | 11(3) | 6(3) |
| C036 | 45(4) | 50(4) | 67(4) | 5(3) | 11(3) | -6(3) |
| C037 | 59(4) | 37(3) | 50(4) | -6(3) | 15(3) | 9(3) |
| C038 | 77(4) | 51(4) | 49(4) | -6(3) | 20(3) | 22(3) |
| C039 | 78(4) | 42(4) | 59(4) | -10(3) | 16(3) | 14(3) |
| C03A | 78(4) | 60(4) | 44(4) | -1(3) | 26(3) | 23(3) |
| C03B | 56(4) | 51(4) | 97(5) | -2(4) | 19(3) | -5(3) |
| C03C | 102(5) | 61(4) | 56(4) | -14(3) | 24(4) | 32(4) |
| C03D | 89(5) | 82(5) | 58(4) | -2(4) | 35(4) | 45(4) |
| C03E | 61(4) | 50(4) | 117(6) | 2(4) | 14(4) | -1(3) |
| C0AA | 64(7) | 68(8) | 57(7) | -10(6) | 34(6) | -12(6) |
| C1AA | 59(7) | 78(9) | 50(7) | 12(7) | 26(5) | 9(7) |
| C2AA | 62(7) | 58(8) | 64(8) | -5(6) | 34(7) | -6(6) |
| C3AA | 62(7) | 69(8) | 66(8) | 11(7) | 42(7) | -12(6) |

| **Bond Angles for ZZ.** | | | | | | | |
| --- | --- | --- | --- | --- | --- | --- | --- |
| **Atom** | **Atom** | **Atom** | **Angle/˚** | **Atom** | **Atom** | **Atom** | **Angle/˚** |
| C8 | O1 | C11 | 107.9(4) | C00M | C01Q | C01T | 120.3(5) |
| C006 | O002 | C00B | 107.8(4) | C02G | C01R | C01N | 120.2(6) |
| C01N | O003 | C01W | 117.1(5) | C2 | C3 | C4 | 120.2(6) |
| C01U | O004 | C01Z | 117.2(5) | O00C | C01T | C013 | 124.4(6) |
| C16 | C15 | C14 | 119.5(4) | O00C | C01T | C01Q | 115.0(5) |
| O002 | C006 | C010 | 115.7(4) | C013 | C01T | C01Q | 120.6(6) |
| C00O | C006 | O002 | 112.7(5) | O004 | C01U | C01V | 115.4(6) |
| C00O | C006 | C010 | 131.6(5) | O004 | C01U | C02A | 125.7(6) |
| O1 | C8 | C5 | 115.0(4) | C02A | C01U | C01V | 118.9(6) |
| C9 | C8 | O1 | 113.7(5) | C02H | C01V | C01U | 120.7(6) |
| C9 | C8 | C5 | 131.3(5) | C25 | C26 | C27 | 118.3(6) |
| C3 | C4 | C5 | 120.3(5) | C02L | C01Y | C02J | 121.6(7) |
| C00Z | C009 | C011 | 121.7(5) | C021 | C020 | C02K | 130.7(7) |
| C13 | C14 | C15 | 119.0(5) | C021 | C020 | C02X | 122.9(6) |
| O002 | C00B | C00G | 107.0(4) | C02K | C020 | C02X | 106.4(6) |
| O002 | C00B | C00V | 104.6(4) | C020 | C021 | C02D | 129.2(6) |
| O002 | C00B | C00Z | 108.3(4) | C02Y | C022 | C02Z | 121.0(7) |
| C00G | C00B | C00V | 112.2(4) | C02G | C023 | C02W | 119.3(6) |
| C00G | C00B | C00Z | 113.3(4) | C02Q | C023 | C02G | 118.8(6) |
| C00V | C00B | C00Z | 110.9(4) | C02Q | C023 | C02W | 121.8(6) |
| C01T | O00C | C028 | 118.3(5) | C02Q | C024 | C01N | 119.7(6) |
| C01G | C00D | C00F | 122.0(5) | C02B | C025 | C02H | 117.8(6) |
| C12 | C13 | C14 | 121.5(4) | C02B | C025 | C02R | 122.2(6) |
| C00D | C00F | C015 | 119.3(4) | C02H | C025 | C02R | 120.0(6) |
| C01J | C00F | C00D | 118.5(5) | C02P | C026 | C02N | 122.5(7) |
| C01J | C00F | C015 | 122.2(5) | C02V | C027 | C02M | 122.0(7) |
| C00B | C00G | C00R | 113.8(4) | C02C | C029 | C035 | 106.0(6) |
| C5 | C6 | C7 | 120.6(5) | C02E | C029 | C02C | 131.3(7) |
| C11 | C18 | C19 | 114.3(4) | C02E | C029 | C035 | 122.7(7) |
| C013 | C00K | C010 | 121.0(5) | C01U | C02A | C02B | 119.9(6) |
| O1 | C11 | C18 | 106.7(4) | C025 | C02B | C02A | 122.0(6) |
| O1 | C11 | C12 | 108.7(4) | C02W | C02C | C029 | 108.5(6) |
| O1 | C11 | C10 | 104.5(4) | C021 | C02D | C02J | 112.9(6) |
| C18 | C11 | C12 | 114.5(4) | C021 | C02D | C02T | 108.6(6) |
| C18 | C11 | C10 | 110.3(3) | C021 | C02D | C02U | 107.3(6) |
| C12 | C11 | C10 | 111.6(4) | C02J | C02D | C02T | 109.1(6) |
| C01Q | C00M | C010 | 120.9(5) | C02J | C02D | C02U | 112.5(6) |
| C2 | O2 | C1 | 118.2(5) | C02T | C02D | C02U | 106.2(6) |
| C006 | C00O | C00V | 110.0(4) | C029 | C02E | C02I | 128.7(7) |
| C8 | C9 | C10 | 108.7(4) | C02S | C02F | C034 | 120.7(7) |
| C13 | C12 | C11 | 123.1(4) | C01R | C02G | C023 | 120.4(6) |
| C13 | C12 | C17 | 118.5(4) | C01V | C02H | C025 | 120.7(6) |
| C17 | C12 | C11 | 118.4(4) | C02E | C02I | C02Z | 112.6(6) |
| C4 | C5 | C8 | 120.9(5) | C02E | C02I | C030 | 107.7(6) |
| C6 | C5 | C8 | 120.3(5) | C02E | C02I | C032 | 107.2(6) |
| C6 | C5 | C4 | 118.8(5) | C02Z | C02I | C030 | 109.4(6) |
| C15 | C16 | C17 | 121.5(4) | C02Z | C02I | C032 | 112.8(6) |
| C25 | C24 | C29 | 118.5(5) | C030 | C02I | C032 | 106.7(6) |
| C25 | C24 | C21 | 119.1(5) | C01Y | C02J | C02D | 123.8(6) |
| C29 | C24 | C21 | 122.4(5) | C01Y | C02J | C02S | 117.1(7) |
| C00O | C00V | C00B | 104.3(4) | C02S | C02J | C02D | 119.2(6) |
| C016 | C00V | C00B | 122.6(5) | C02R | C02K | C020 | 108.0(6) |
| C016 | C00V | C00O | 133.1(5) | C01Y | C02L | C034 | 120.0(7) |
| C01L | C00W | C011 | 121.4(5) | C027 | C02M | C035 | 120.2(6) |
| C2 | C7 | C6 | 119.3(6) | C027 | C02M | C039 | 117.4(7) |
| C16 | C17 | C12 | 120.0(5) | C039 | C02M | C035 | 122.3(8) |
| C009 | C00Z | C00B | 122.5(4) | C026 | C02N | C02X | 120.5(6) |
| C009 | C00Z | C01H | 119.7(4) | C026 | C02N | C037 | 117.3(7) |
| C01H | C00Z | C00B | 117.8(5) | C037 | C02N | C02X | 122.1(7) |
| C00K | C010 | C006 | 121.4(5) | C026 | C02P | C03A | 118.6(8) |
| C00M | C010 | C006 | 120.5(5) | C023 | C02Q | C024 | 121.6(6) |
| C00M | C010 | C00K | 118.1(5) | O01A | C02R | C025 | 113.3(6) |
| C009 | C011 | C00W | 118.1(5) | C02K | C02R | O01A | 114.1(6) |
| C01T | C013 | C00K | 119.1(6) | C02K | C02R | C025 | 132.5(7) |
| C016 | C015 | C00F | 110.7(4) | C02F | C02S | C02J | 121.2(7) |
| C016 | C015 | C019 | 108.9(4) | C027 | C02V | C03D | 119.1(9) |
| C017 | C015 | C00F | 112.2(4) | O01B | C02W | C023 | 113.9(6) |
| C017 | C015 | C016 | 108.3(4) | C02C | C02W | O01B | 113.7(6) |
| C017 | C015 | C019 | 108.4(4) | C02C | C02W | C023 | 132.3(7) |
| C019 | C015 | C00F | 108.3(4) | O01A | C02X | C020 | 103.8(5) |
| C00V | C016 | C015 | 128.1(5) | O01A | C02X | C02N | 106.9(6) |
| C10 | C20 | C21 | 129.4(5) | O01A | C02X | C03B | 106.1(6) |
| C02R | O01A | C02X | 106.7(5) | C020 | C02X | C03B | 111.4(7) |
| C02W | O01B | C035 | 107.5(5) | C02N | C02X | C020 | 112.8(6) |
| C9 | C10 | C11 | 103.8(4) | C02N | C02X | C03B | 114.8(6) |
| C20 | C10 | C11 | 122.6(5) | C022 | C02Y | C036 | 120.2(8) |
| C20 | C10 | C9 | 133.6(5) | C022 | C02Z | C02I | 123.1(6) |
| C27 | C28 | C29 | 119.9(6) | C022 | C02Z | C033 | 117.6(7) |
| C01I | C01E | C01G | 120.3(6) | C033 | C02Z | C02I | 119.4(7) |
| C24 | C25 | C26 | 121.7(5) | C036 | C031 | C033 | 121.3(8) |
| C00D | C01G | C01E | 118.8(6) | C031 | C033 | C02Z | 120.6(8) |
| C01L | C01H | C00Z | 119.5(5) | C02F | C034 | C02L | 119.4(7) |
| C01E | C01I | C01J | 120.3(5) | O01B | C035 | C029 | 103.4(6) |
| C00F | C01J | C01I | 120.2(6) | O01B | C035 | C02M | 109.1(6) |
| C24 | C29 | C28 | 120.8(6) | O01B | C035 | C03E | 105.4(6) |
| C00W | C01L | C01H | 119.6(5) | C029 | C035 | C03E | 109.7(7) |
| C24 | C21 | C22 | 109.0(4) | C02M | C035 | C029 | 112.1(6) |
| C23 | C21 | C24 | 112.7(4) | C02M | C035 | C03E | 116.0(7) |
| C23 | C21 | C22 | 109.3(5) | C031 | C036 | C02Y | 119.3(8) |
| C23 | C21 | C20 | 106.3(4) | C038 | C037 | C02N | 120.2(8) |
| C20 | C21 | C24 | 110.5(4) | C03A | C038 | C037 | 121.5(8) |
| C20 | C21 | C22 | 109.0(4) | C03C | C039 | C02M | 119.6(9) |
| O003 | C01N | C01R | 115.1(6) | C038 | C03A | C02P | 119.9(8) |
| O003 | C01N | C024 | 125.6(6) | C1AA | C03B | C02X | 128.3(11) |
| C024 | C01N | C01R | 119.2(6) | C2AA | C03B | C02X | 118.5(10) |
| C28 | C27 | C26 | 120.7(6) | C03D | C03C | C039 | 122.6(8) |
| C7 | C2 | O2 | 123.8(6) | C03C | C03D | C02V | 119.3(8) |
| C7 | C2 | C3 | 121.0(6) | C0AA | C03E | C035 | 128.0(11) |
| C3 | C2 | O2 | 115.2(6) | C3AA | C03E | C035 | 121.8(10) |

| **Hydrogen Atom Coordinates (Å×10^4^) and Isotropic Displacement Parameters (Å^2^×10^3^) for ZZ.** | | | | |
| --- | --- | --- | --- | --- |
| **Atom** | ***x*** | ***y*** | ***z*** | **U(eq)** |
| H15 | 6899.93 | 4009.19 | 3567.18 | 25 |
| H4 | 4998.9 | 5193.97 | 7419.6 | 29 |
| H009 | 357.95 | 6109.01 | -916.49 | 24 |
| H14 | 6007.09 | 3708.33 | 3719.11 | 23 |
| H00D | 2200.72 | 5547.34 | 3734.93 | 27 |
| H13 | 5119.02 | 3864.99 | 4397.92 | 22 |
| H00A | -977.14 | 5806.52 | -2029.83 | 15 |
| H00B | -630.12 | 5986.21 | -631.08 | 15 |
| H6 | 4007.89 | 5003.16 | 3244.26 | 29 |
| H19A | 3841.45 | 4096.04 | 2621.91 | 29 |
| H19B | 3430.75 | 4367.85 | 2669.73 | 29 |
| H19C | 3074.13 | 4091.38 | 2809 | 29 |
| H18A | 3874.08 | 4275.82 | 5129.36 | 17 |
| H18B | 4319.84 | 4013.95 | 5071.13 | 17 |
| H00K | -936.58 | 4964.35 | -2099.55 | 26 |
| H00M | 21.66 | 4827.57 | 2117.15 | 30 |
| H00O | 362.44 | 5283.85 | 2525.47 | 22 |
| H9 | 5421.39 | 4740.3 | 7963.37 | 27 |
| H00C | -1948 | 5809.57 | -1330.39 | 30 |
| H00E | -1270.95 | 5704.02 | 231.26 | 30 |
| H00F | -1596.12 | 5515.26 | -1131.59 | 30 |
| H16 | 6911.44 | 4446.89 | 4155.02 | 25 |
| H00W | 2286.41 | 5911.2 | -1245.89 | 35 |
| H7 | 3534.79 | 5441.08 | 2598.05 | 35 |
| H17 | 6066 | 4599.78 | 4915.2 | 23 |
| H011 | 1391.37 | 6237.93 | -1302.02 | 33 |
| H22A | 7406.31 | 4048.95 | 9775.71 | 41 |
| H22B | 6805.52 | 3810.32 | 9562.44 | 41 |
| H22C | 7266.37 | 3972.01 | 11035.5 | 41 |
| H013 | -1460.14 | 4533.33 | -2625.92 | 34 |
| H23A | 5890.85 | 4047.21 | 10695.33 | 39 |
| H23B | 5434.12 | 3903.01 | 9170.02 | 39 |
| H23C | 5188.14 | 4199.07 | 9305.93 | 39 |
| H016 | 791.4 | 5982.14 | 1799.2 | 23 |
| H01G | -0.73 | 5789.54 | 3710.83 | 33 |
| H01H | 175.7 | 6093.92 | 3570.64 | 33 |
| H01I | 646.44 | 5953.07 | 5105.18 | 33 |
| H20 | 5929.49 | 4040.12 | 7333.14 | 28 |
| H01J | 1959.42 | 6086.83 | 5500.58 | 39 |
| H01K | 1544.93 | 6225.37 | 3964.1 | 39 |
| H01L | 2233.96 | 6003.71 | 4418.46 | 39 |
| H28 | 6469.1 | 4915.08 | 12013.23 | 43 |
| H01M | 2477.68 | 4932.93 | 6299.26 | 39 |
| H25 | 7325.57 | 4498.2 | 9259.23 | 35 |
| H01N | 2831.4 | 5147.48 | 4811.4 | 35 |
| H01O | 1156.41 | 5351.13 | -294.84 | 27 |
| H01P | 1565.21 | 5127.58 | 6769.52 | 36 |
| H01Q | 935.31 | 5534.13 | 5682.82 | 32 |
| H29 | 5999.07 | 4489.4 | 11099.71 | 35 |
| H01S | 2189.59 | 5475.65 | -707.6 | 35 |
| H27 | 7358.93 | 5131.59 | 11548.58 | 50 |
| H01T | -518.85 | 4404.67 | 1582.11 | 33 |
| H01R | 5499.48 | 1873.95 | 8098.59 | 39 |
| H3 | 4541.38 | 5626.17 | 6760.68 | 32 |
| H01V | 10511.21 | 1903.41 | 8150.16 | 40 |
| H01A | 6952.02 | 1377.3 | 11921.08 | 62 |
| H01B | 6537.59 | 1593.45 | 12399.03 | 62 |
| H01C | 7351.8 | 1665.23 | 12419.69 | 62 |
| H26 | 7807.99 | 4924.52 | 10166.78 | 48 |
| H01Y | 9076.32 | 3031.49 | 3918.99 | 46 |
| H01D | 11912.55 | 1407.67 | 12005.54 | 61 |
| H01E | 11463.77 | 1619.46 | 12436.5 | 61 |
| H01F | 12291.14 | 1697.81 | 12516.84 | 61 |
| H021 | 9169.96 | 3492.53 | 7686.91 | 43 |
| H022 | 4053.12 | 3005.08 | 3898.68 | 53 |
| H024 | 6530.3 | 2035.65 | 12319.41 | 37 |
| H026 | 8911.64 | 2896.42 | 9963.82 | 54 |
| H027 | 3935.56 | 2862.59 | 10024.36 | 61 |
| H02R | -2343.77 | 4185.07 | -2661.95 | 56 |
| H02T | -1976.58 | 3894.14 | -2134.94 | 56 |
| H02U | -1555.41 | 4093.52 | -2680.7 | 56 |
| H02A | 11469.99 | 2067.89 | 12344.83 | 38 |
| H02B | 10966.04 | 2497.67 | 11725.14 | 44 |
| H02C | 4653.26 | 2760.66 | 7071.46 | 47 |
| H02E | 4209.97 | 3464.24 | 7736.87 | 51 |
| H02F | 7207.56 | 2631.8 | 4787.08 | 62 |
| H02G | 5023.53 | 2307.68 | 7537.77 | 40 |
| H02H | 10054.01 | 2335.85 | 7554.31 | 40 |
| H02K | 9643.27 | 2792.1 | 7049.42 | 47 |
| H02L | 8528.97 | 2623.77 | 2926.72 | 59 |
| H1A | 2791.34 | 5821.69 | 2372.42 | 60 |
| H1B | 3637.6 | 5887.87 | 2494.07 | 60 |
| H1C | 3194.61 | 6108.84 | 2887.07 | 60 |
| H02P | 8008.29 | 3024.36 | 10619.43 | 61 |
| H02Q | 6021.46 | 2465.07 | 11730.03 | 42 |
| H02S | 7743.13 | 3040.6 | 5770.85 | 56 |
| H02D | 7732.26 | 3504.66 | 5235.96 | 79 |
| H02I | 7922.22 | 3570.2 | 4023.52 | 79 |
| H02J | 8391.04 | 3727.32 | 5510.86 | 79 |
| H02M | 9705.79 | 3620.6 | 5863.59 | 83 |
| H02N | 9274.67 | 3461.48 | 4382.85 | 83 |
| H02O | 9969.9 | 3321.8 | 5828.65 | 83 |
| H02V | 2994.96 | 3000.76 | 10575.43 | 72 |
| H02Y | 3497.77 | 2596.59 | 2908.14 | 69 |
| H03A | 2719.85 | 3464.63 | 5237.6 | 86 |
| H03B | 2939.69 | 3552.28 | 4088.59 | 86 |
| H03C | 3383.66 | 3692.26 | 5624.58 | 86 |
| H031 | 2218.58 | 2600.8 | 4835.83 | 71 |
| H03D | 4730.17 | 3582.89 | 5923.8 | 88 |
| H03E | 4242.12 | 3445.83 | 4379.6 | 88 |
| H03F | 4936.15 | 3284.52 | 5736.3 | 88 |
| H033 | 2766.35 | 3007.54 | 5847.83 | 63 |
| H034 | 7594.76 | 2417.97 | 3380.95 | 68 |
| H036 | 2609.92 | 2384.68 | 3449.97 | 76 |
| H037 | 9797.64 | 3642.85 | 10577.55 | 66 |
| H038 | 8872.53 | 3771 | 11199.21 | 79 |
| H039 | 4788.11 | 3612.66 | 10517.7 | 84 |
| H03M | 7998.99 | 3472.11 | 11256.84 | 77 |
| H03P | 11083.63 | 3273.42 | 9878.12 | 95 |
| H03Q | 10669.16 | 3508.37 | 10211.67 | 95 |
| H03N | 10719.58 | 3487.35 | 9893.02 | 95 |
| H03O | 11011.2 | 3372.16 | 11435.95 | 95 |
| H03G | 3829.79 | 3744.16 | 11044.59 | 98 |
| H03H | 2930.64 | 3454.41 | 11045.9 | 94 |
| H03I | 6104.87 | 3222.55 | 9920.08 | 111 |
| H03J | 5691.48 | 3467.72 | 10181.74 | 111 |
| H03K | 5733.45 | 3442.64 | 9873.41 | 111 |
| H03L | 6010.89 | 3341.47 | 11430.56 | 111 |
| H0AA | 6279.17 | 3389.29 | 12321.83 | 93 |
| H0AB | 6914.84 | 3382.48 | 11811.3 | 93 |
| H0AC | 6611.92 | 3111.87 | 12134.61 | 93 |
| H1AA | 11259.22 | 3372.31 | 12383.94 | 96 |
| H1AB | 11854.63 | 3457.39 | 11855.24 | 96 |
| H1AC | 11711.81 | 3150.66 | 12015.63 | 96 |
| H2AA | 11595.9 | 3045.43 | 11031.64 | 92 |
| H2AB | 11943.8 | 3332.45 | 11022 | 92 |
| H2AC | 11346.16 | 3182.05 | 9544.8 | 92 |
| H3AA | 6622.79 | 3015.32 | 11139.38 | 93 |
| H3AB | 6951.5 | 3304.29 | 11104.7 | 93 |
| H3AC | 6393.48 | 3143.91 | 9645.57 | 93 |
