# Supplementary material for: Marine furanocembranoids-inspired macrocycles enabled by Pd-catalyzed unactivated C(sp3)-H olefination mediated by donor/donor carbenes
Source: Nat Commun. 2021 Feb 26;12:1304. doi: 10.1038/s41467-021-21484-x (PMC7910576; doi:10.1038/s41467-021-21484-x)
Supplement: Supplementary file 5 — Supplementary Data 2 [file 41467_2021_21484_MOESM5_ESM.docx]

**Cartesian coordinates**

**HOAc**

E = -228.968030265 a.u.

C -0.132881000 0.129496000 -0.000048000

C 1.361524000 0.020260000 0.000049000

H 1.810156000 1.014396000 -0.000240000

H 1.702655000 -0.531616000 0.883566000

H 1.702806000 -0.531940000 -0.883234000

O -0.795191000 -1.045016000 0.000118000

H -0.169411000 -1.785538000 -0.000802000

O -0.757067000 1.162036000 -0.000030000

**OAc^-^**

E = -228.469525563 a.u.

C -0.206579000 0.000854000 -0.000010000

C 1.339967000 -0.055104000 -0.000003000

H 1.737712000 0.471227000 -0.878238000

H 1.737659000 0.470747000 0.878536000

H 1.724551000 -1.082117000 -0.000252000

O -0.806721000 -1.098041000 0.000002000

O -0.693311000 1.156247000 0.000002000

**Br^-^**

E = -2571.38927178 a.u.

Br 0.000000000 0.000000000 0.000000000

**1a**

E = -401.952278900 a.u.

C -0.409963000 3.015272000 -0.000044000

C -1.193946000 1.870212000 -0.000010000

C -0.599256000 0.609384000 0.000000000

C 0.796399000 0.423323000 -0.000018000

C 1.542790000 1.613952000 -0.000056000

C 0.970561000 2.879949000 -0.000070000

H -0.881049000 3.995097000 -0.000050000

H -2.277594000 1.952817000 0.000007000

H 2.626148000 1.553322000 -0.000077000

H 1.612675000 3.757650000 -0.000100000

Br -1.890903000 -0.827972000 0.000030000

C 1.523634000 -0.931828000 0.000004000

C 1.176096000 -1.728811000 1.263835000

H 0.113228000 -1.974602000 1.338829000

H 1.736755000 -2.672844000 1.268146000

H 1.458896000 -1.167547000 2.164073000

C 3.044988000 -0.761105000 -0.000018000

H 3.507954000 -1.755202000 0.000008000

H 3.405799000 -0.231691000 -0.890650000

H 3.405818000 -0.231636000 0.890573000

C 1.176065000 -1.728871000 -1.263781000

H 0.113197000 -1.974679000 -1.338729000

H 1.458828000 -1.167644000 -2.164053000

H 1.736733000 -2.672899000 -1.268068000

**2a**

E = -616.878068635 a.u.

C 1.733992000 -0.582752000 -0.022901000

C 0.553991000 -0.874479000 -0.027375000

C 3.145592000 -0.185104000 -0.009975000

C 3.750980000 -0.438702000 -1.394390000

H 3.226983000 0.134974000 -2.168271000

H 4.805062000 -0.133047000 -1.394462000

H 3.701940000 -1.501302000 -1.660229000

C 3.234117000 1.305064000 0.333644000

H 2.800257000 1.508167000 1.320255000

H 4.285123000 1.620759000 0.347841000

H 2.701529000 1.913540000 -0.407692000

C 3.894274000 -1.008557000 1.042314000

H 3.472325000 -0.852212000 2.042302000

H 3.849429000 -2.079952000 0.813407000

H 4.948854000 -0.705446000 1.062687000

C -0.827283000 -1.178271000 -0.037944000

H -1.109814000 -2.228230000 -0.129105000

C -1.780764000 -0.227733000 0.047841000

C -3.231774000 -0.509053000 0.004188000

O -4.015633000 0.431122000 0.034747000

C -1.409867000 1.227371000 0.201654000

O -1.184074000 1.691004000 1.300905000

C -3.699169000 -1.931013000 -0.075917000

H -3.327043000 -2.414306000 -0.986873000

H -3.328298000 -2.512999000 0.775843000

H -4.791287000 -1.949927000 -0.079279000

C -1.321003000 2.012084000 -1.066968000

H -0.539487000 1.577167000 -1.704957000

H -2.264823000 1.931473000 -1.619971000

H -1.091607000 3.061192000 -0.861731000

**A**

E = -1531.22346166 a.u.

C 2.858852000 -1.655932000 -0.148698000

C 3.502706000 -2.856564000 -0.468833000

C 4.714569000 -2.868039000 -1.153043000

C 5.308137000 -1.668362000 -1.526903000

C 4.679735000 -0.467937000 -1.215459000

C 3.457571000 -0.438848000 -0.536520000

H 3.062149000 -3.808761000 -0.182642000

H 5.192593000 -3.816456000 -1.388705000

H 6.255962000 -1.664656000 -2.060648000

H 5.136516000 0.478445000 -1.505359000

C 2.849436000 0.890130000 -0.241244000

C 3.213793000 1.589226000 0.920088000

C 1.953485000 1.471051000 -1.161095000

C 2.617106000 2.826585000 1.184557000

C 1.398945000 2.716050000 -0.872885000

C 1.703124000 3.402560000 0.305586000

H 2.893705000 3.362056000 2.094284000

H 0.702509000 3.161036000 -1.587258000

P 1.253157000 -1.664847000 0.782434000

P -0.558536000 -0.323878000 0.206182000

C 0.805104000 -3.450556000 0.860580000

C 1.802145000 -1.414696000 2.529883000

C 1.068364000 4.728319000 0.597503000

H 1.418259000 5.140490000 1.550136000

H 1.290660000 5.461243000 -0.188489000

H -0.025762000 4.646635000 0.648899000

C 4.268702000 1.066562000 1.850795000

H 5.273211000 1.306597000 1.474775000

H 4.177378000 1.520594000 2.843833000

H 4.231865000 -0.022136000 1.966748000

C 1.602152000 0.769398000 -2.438613000

H 0.957392000 1.394707000 -3.065351000

H 2.495410000 0.510812000 -3.022068000

H 1.062695000 -0.169355000 -2.242981000

H -0.120645000 -3.516035000 1.444550000

H 1.572152000 -4.058404000 1.355007000

H 0.605916000 -3.845649000 -0.141148000

H 0.958942000 -1.678889000 3.179680000

H 2.049133000 -0.362871000 2.705059000

H 2.662076000 -2.050638000 2.774742000

C -1.934871000 1.976323000 1.937712000

C -1.787208000 1.775048000 0.563057000

C -2.442189000 0.696019000 -0.082825000

C -3.198231000 -0.267452000 0.661272000

C -3.322640000 0.008775000 2.034342000

C -2.738011000 1.107574000 2.661191000

H -1.452096000 2.829444000 2.410055000

H -1.266469000 2.514380000 -0.042429000

H -3.913147000 -0.664849000 2.647710000

H -2.901499000 1.264126000 3.724847000

B -2.602184000 0.955864000 -2.012007000

C -3.949359000 -1.466068000 0.060233000

C -5.247521000 -0.964974000 -0.587180000

H -5.058074000 -0.277030000 -1.418779000

H -5.823343000 -1.814112000 -0.978285000

H -5.871789000 -0.444415000 0.150703000

C -4.333626000 -2.480260000 1.143109000

H -4.780771000 -3.359503000 0.663723000

H -3.456201000 -2.819263000 1.711188000

H -5.076509000 -2.093493000 1.850358000

C -3.112196000 -2.244591000 -0.962648000

H -2.821284000 -1.651994000 -1.833648000

H -2.193580000 -2.630224000 -0.496578000

H -3.694068000 -3.102032000 -1.326366000

**TS1**

E = -1531.21625798 a.u.

C -2.531952000 0.955076000 -2.862246000

C -2.129378000 -0.210733000 -2.219292000

C -2.296324000 -0.345162000 -0.828971000

C -2.931045000 0.648867000 -0.036739000

C -3.349574000 1.781485000 -0.748991000

C -3.158970000 1.947377000 -2.119961000

H -2.378104000 1.065623000 -3.933621000

H -1.706013000 -1.034637000 -2.790469000

H -3.838718000 2.587910000 -0.208486000

H -3.511793000 2.857242000 -2.600796000

Br -2.495165000 -2.502829000 -0.346609000

C -3.204215000 0.562982000 1.473433000

C -3.416323000 1.953797000 2.081733000

H -2.561235000 2.615572000 1.887091000

H -3.523597000 1.854300000 3.168907000

H -4.325394000 2.446536000 1.718329000

C -4.491844000 -0.241303000 1.700483000

H -4.724025000 -0.283599000 2.773313000

H -4.405248000 -1.268962000 1.329492000

H -5.339694000 0.235154000 1.190380000

C -2.050056000 -0.072769000 2.259125000

H -2.283203000 -0.045512000 3.332822000

H -1.113816000 0.486182000 2.106139000

H -1.876323000 -1.120519000 1.992804000

C 3.314100000 -0.873507000 0.049693000

C 4.429860000 -1.701597000 -0.116514000

C 5.644677000 -1.194487000 -0.567070000

C 5.763924000 0.160681000 -0.853575000

C 4.664800000 0.995604000 -0.686278000

C 3.434485000 0.499610000 -0.244173000

H 4.360759000 -2.764699000 0.104226000

H 6.495014000 -1.861307000 -0.691694000

H 6.708680000 0.567639000 -1.207393000

H 4.747223000 2.059758000 -0.907805000

C 2.301628000 1.458805000 -0.101437000

C 1.446570000 1.699212000 -1.195950000

C 2.124347000 2.168602000 1.097244000

C 0.417703000 2.630179000 -1.059853000

C 1.067398000 3.077852000 1.200324000

C 0.208599000 3.328439000 0.131839000

H -0.239251000 2.819522000 -1.911230000

H 0.931028000 3.624096000 2.135396000

P 1.694894000 -1.569529000 0.618824000

Pd -0.420807000 -0.906546000 -0.221946000

C 1.775712000 -1.363312000 2.454858000

C 1.941876000 -3.392091000 0.512477000

C -0.876976000 4.356467000 0.235531000

H -0.556833000 5.312440000 -0.200903000

H -1.149046000 4.553413000 1.278947000

H -1.779707000 4.046564000 -0.306332000

C 1.645476000 0.988493000 -2.501970000

H 2.643123000 1.176822000 -2.919914000

H 0.903840000 1.313612000 -3.240033000

H 1.548528000 -0.101179000 -2.388245000

C 3.085631000 2.019898000 2.240372000

H 3.987016000 2.626003000 2.071953000

H 3.427319000 0.987536000 2.376652000

H 2.636572000 2.359944000 3.180189000

H 2.759387000 -3.752985000 1.147575000

H 2.113813000 -3.696435000 -0.525285000

H 1.006230000 -3.852577000 0.851889000

H 0.992189000 -1.993163000 2.894252000

H 2.751627000 -1.682379000 2.841611000

H 1.583365000 -0.325637000 2.744137000

**IM1**

E = -1531.2558971 a.u.

C 3.090533000 -2.721236000 -1.737651000

C 2.071482000 -1.789522000 -1.591323000

C 1.996093000 -0.961323000 -0.462922000

C 2.977717000 -1.018065000 0.539581000

C 3.988429000 -1.985751000 0.362135000

C 4.053412000 -2.827447000 -0.737913000

H 3.126313000 -3.357509000 -2.620261000

H 1.309706000 -1.709274000 -2.366652000

H 4.766457000 -2.073482000 1.119463000

H 4.859092000 -3.554544000 -0.817662000

Br 1.871548000 1.869916000 -1.819086000

C 3.108835000 -0.133004000 1.793624000

C 3.121784000 -1.020248000 3.045939000

H 3.959686000 -1.726767000 3.052396000

H 3.207837000 -0.399177000 3.947725000

H 2.193852000 -1.603814000 3.128550000

C 2.009593000 0.910839000 1.984627000

H 2.226065000 1.483540000 2.897608000

H 1.967189000 1.621083000 1.149302000

H 1.017190000 0.460861000 2.114137000

C 4.436971000 0.634033000 1.709645000

H 4.559578000 1.273035000 2.594836000

H 5.304516000 -0.034428000 1.661382000

H 4.458997000 1.278994000 0.820977000

C -2.567981000 -1.663331000 -0.034370000

C -3.156883000 -2.895815000 -0.329760000

C -4.496295000 -2.967751000 -0.703945000

C -5.260698000 -1.809063000 -0.785239000

C -4.681560000 -0.576578000 -0.498516000

C -3.339784000 -0.490389000 -0.129296000

H -2.578903000 -3.815042000 -0.271529000

H -4.940475000 -3.934698000 -0.929072000

H -6.307105000 -1.862323000 -1.076878000

H -5.268904000 0.338489000 -0.567645000

C -2.701535000 0.820071000 0.146269000

C -1.951446000 1.440374000 -0.885117000

C -2.798011000 1.411626000 1.409925000

C -1.239944000 2.604751000 -0.580636000

C -2.068345000 2.580526000 1.666961000

C -1.268439000 3.175099000 0.700066000

H -0.672922000 3.096638000 -1.369540000

H -2.127388000 3.028345000 2.660202000

P -0.803013000 -1.522326000 0.498625000

Pd 0.399740000 0.252236000 -0.506052000

C -0.906298000 -1.439833000 2.334230000

C -0.147106000 -3.221246000 0.290041000

C -0.458165000 4.399334000 0.989984000

H -0.722822000 5.225127000 0.317674000

H -0.599722000 4.742230000 2.020274000

H 0.612134000 4.202639000 0.840524000

C -2.052480000 0.965467000 -2.309491000

H -3.041036000 1.210051000 -2.723075000

H -1.297107000 1.451845000 -2.935689000

H -1.928987000 -0.119900000 -2.405061000

C -3.669261000 0.830009000 2.484268000

H -4.727119000 1.066666000 2.306734000

H -3.597916000 -0.262581000 2.537321000

H -3.403343000 1.237008000 3.465820000

H -0.739428000 -3.926881000 0.882904000

H -0.152864000 -3.520332000 -0.762492000

H 0.884699000 -3.238498000 0.656479000

H 0.086255000 -1.669575000 2.739059000

H -1.620566000 -2.187960000 2.697775000

H -1.203724000 -0.443954000 2.674729000

**IM2**

E = -1746.23806745 a.u.

C 0.634050000 1.959398000 2.914276000

C 0.929153000 0.888559000 2.071034000

C 1.641158000 1.061081000 0.877513000

C 2.106297000 2.350128000 0.514459000

C 1.776414000 3.408237000 1.375594000

C 1.046767000 3.231937000 2.549027000

H 0.071307000 1.792905000 3.831802000

H 0.573104000 -0.105993000 2.346528000

H 2.102099000 4.416738000 1.133114000

H 0.813930000 4.092484000 3.173193000

C 3.003467000 2.617820000 -0.707076000

C 3.293028000 4.106488000 -0.907965000

H 3.852028000 4.541873000 -0.070281000

H 3.909221000 4.230427000 -1.807520000

H 2.376243000 4.692415000 -1.053879000

C 2.378125000 2.114245000 -2.011125000

H 3.083601000 2.260100000 -2.840569000

H 2.131408000 1.043653000 -1.967486000

H 1.464465000 2.673146000 -2.255072000

C 4.350536000 1.918458000 -0.488155000

H 5.030585000 2.136327000 -1.323717000

H 4.827488000 2.277194000 0.434441000

H 4.241921000 0.829788000 -0.409096000

C -1.225928000 -1.584606000 0.360679000

C -0.506726000 -2.666567000 0.890518000

C -1.108248000 -3.603087000 1.721371000

C -2.452141000 -3.462997000 2.049162000

C -3.176640000 -2.398887000 1.527660000

C -2.593014000 -1.452813000 0.676136000

H 0.552272000 -2.772447000 0.648798000

H -0.524807000 -4.432054000 2.114911000

H -2.936806000 -4.180698000 2.707105000

H -4.232460000 -2.286768000 1.773292000

C -3.478274000 -0.380712000 0.141739000

C -4.182029000 -0.593455000 -1.057873000

C -3.643213000 0.816687000 0.854562000

C -4.997812000 0.424518000 -1.550906000

C -4.473652000 1.808852000 0.330719000

C -5.153760000 1.635393000 -0.873137000

H -5.532368000 0.264878000 -2.489167000

H -4.586736000 2.746636000 0.876905000

P -0.228061000 -0.497591000 -0.755971000

Pd 1.969533000 -0.629138000 -0.154280000

C -0.392690000 -1.259869000 -2.415738000

C -1.000200000 1.141940000 -0.985026000

C -6.043772000 2.706997000 -1.425159000

H -7.094780000 2.390230000 -1.427544000

H -5.976580000 3.628722000 -0.837397000

H -5.786197000 2.947913000 -2.463910000

C -4.100217000 -1.902835000 -1.785774000

H -4.733860000 -2.659749000 -1.302601000

H -4.444916000 -1.801663000 -2.820559000

H -3.084838000 -2.315468000 -1.800124000

C -2.934323000 1.045190000 2.155952000

H -3.420720000 0.502444000 2.978487000

H -1.894810000 0.697658000 2.121688000

H -2.928460000 2.108487000 2.421158000

H -1.978904000 1.046196000 -1.463116000

H -1.089405000 1.681541000 -0.037382000

H -0.334631000 1.702018000 -1.650878000

H 0.314595000 -0.773395000 -3.097233000

H -1.410787000 -1.128127000 -2.800328000

H -0.155974000 -2.327237000 -2.357408000

C 3.817983000 -2.379186000 -0.406622000

O 2.718515000 -2.528327000 -1.022852000

O 4.017297000 -1.357657000 0.320937000

C 4.874841000 -3.434024000 -0.508826000

H 5.872429000 -2.999579000 -0.401721000

H 4.734753000 -4.158034000 0.302628000

H 4.797392000 -3.971780000 -1.457439000

**TS_2-3_**

E = -1746.19643043 a.u.

C -1.001089000 -3.703639000 0.096287000

C -0.851277000 -2.326645000 0.265832000

C -1.901510000 -1.435937000 0.011511000

C -3.150870000 -1.967403000 -0.379426000

C -3.282582000 -3.347002000 -0.565817000

C -2.218298000 -4.214944000 -0.337579000

H -0.162599000 -4.367646000 0.302914000

H 0.113043000 -1.959286000 0.618062000

H -4.240576000 -3.756436000 -0.886461000

H -2.346735000 -5.285617000 -0.485135000

C -4.316160000 -1.020450000 -0.590892000

C -5.650644000 -1.642944000 -0.174208000

H -5.626028000 -1.975124000 0.872407000

H -6.451917000 -0.897845000 -0.271089000

H -5.934266000 -2.502001000 -0.796118000

C -4.396443000 -0.638806000 -2.073156000

H -5.196467000 0.095986000 -2.244058000

H -3.452533000 -0.199103000 -2.422454000

H -4.609610000 -1.519943000 -2.693793000

C -4.060329000 0.228409000 0.259465000

H -4.971754000 0.850392000 0.279929000

H -3.920706000 -0.038319000 1.319476000

H -3.613085000 1.374940000 -0.409800000

C 1.449610000 0.093682000 1.396189000

C 0.720102000 0.052992000 2.592705000

C 1.311983000 -0.327857000 3.791265000

C 2.654556000 -0.688704000 3.805430000

C 3.389113000 -0.653001000 2.626257000

C 2.813692000 -0.260531000 1.412652000

H -0.339112000 0.316947000 2.573665000

H 0.723303000 -0.348236000 4.705389000

H 3.131910000 -0.996747000 4.733051000

H 4.443158000 -0.929801000 2.632535000

C 3.695269000 -0.213339000 0.212363000

C 4.407772000 0.961312000 -0.078340000

C 3.858713000 -1.355462000 -0.593489000

C 5.208960000 1.002196000 -1.222645000

C 4.675274000 -1.277032000 -1.720026000

C 5.349709000 -0.102201000 -2.059647000

H 5.746725000 1.922245000 -1.457556000

H 4.789374000 -2.159173000 -2.352151000

P 0.477784000 0.697905000 -0.057625000

Pd -1.833253000 0.573118000 0.210891000

C 1.027774000 2.428606000 -0.319804000

C 1.014545000 -0.080856000 -1.629031000

C 6.229608000 -0.051174000 -3.271459000

H 5.756024000 -0.536070000 -4.133368000

H 6.472116000 0.980363000 -3.549112000

H 7.178317000 -0.575408000 -3.095568000

C 4.371472000 2.148806000 0.838941000

H 5.055482000 2.004457000 1.686889000

H 4.688234000 3.058714000 0.317354000

H 3.378837000 2.326997000 1.267122000

C 3.174508000 -2.646567000 -0.255474000

H 3.482966000 -3.019958000 0.729905000

H 2.081778000 -2.542708000 -0.218955000

H 3.410497000 -3.420008000 -0.993803000

H 2.041847000 0.188698000 -1.892421000

H 0.900787000 -1.167876000 -1.599145000

H 0.333515000 0.312481000 -2.394031000

H 0.415277000 2.863873000 -1.116973000

H 2.080282000 2.446401000 -0.624112000

H 0.885548000 3.016104000 0.591263000

C -2.769653000 3.254318000 -0.316310000

O -3.637328000 2.562856000 -0.938863000

O -1.884802000 2.745668000 0.422496000

C -2.821285000 4.745820000 -0.444202000

H -1.810302000 5.150771000 -0.549159000

H -3.440147000 5.053780000 -1.289391000

H -3.248382000 5.164092000 0.474663000

**IM3**

E = -1746.22365022 a.u.

C -2.549941000 -3.476233000 -0.774121000

C -1.760054000 -2.536605000 -0.107718000

C -2.162807000 -1.199941000 0.015831000

C -3.401301000 -0.826422000 -0.554557000

C -4.185147000 -1.769583000 -1.221315000

C -3.765524000 -3.093975000 -1.331335000

H -2.208753000 -4.507318000 -0.856545000

H -0.803748000 -2.869076000 0.297403000

H -5.137750000 -1.467886000 -1.659508000

H -4.385719000 -3.822646000 -1.850388000

C -3.770800000 0.632743000 -0.437262000

C -5.267660000 0.861539000 -0.221045000

H -5.637481000 0.289949000 0.640656000

H -5.458729000 1.925948000 -0.024987000

H -5.868648000 0.580099000 -1.096955000

C -3.348359000 1.359692000 -1.718282000

H -3.558482000 2.437052000 -1.642772000

H -2.273524000 1.231488000 -1.913571000

H -3.890388000 0.972290000 -2.592003000

C -2.973213000 1.159763000 0.757709000

H -2.950617000 2.262551000 0.777937000

H -3.453055000 0.839217000 1.698264000

H -1.046504000 2.387363000 -0.255697000

C 1.955841000 -2.016925000 0.443824000

C 2.124787000 -3.385794000 0.680569000

C 2.889855000 -4.176119000 -0.172033000

C 3.504937000 -3.603538000 -1.278947000

C 3.341018000 -2.244767000 -1.528058000

C 2.566010000 -1.439673000 -0.689685000

H 1.652879000 -3.854621000 1.541423000

H 3.004614000 -5.237972000 0.034520000

H 4.109377000 -4.211243000 -1.948750000

H 3.815343000 -1.787029000 -2.396347000

C 2.409663000 0.002814000 -1.030878000

C 3.331468000 0.948210000 -0.555921000

C 1.362111000 0.406094000 -1.884864000

C 3.142836000 2.298626000 -0.870372000

C 1.221289000 1.757667000 -2.191039000

C 2.094599000 2.722977000 -1.682120000

H 3.853704000 3.033011000 -0.486926000

H 0.405549000 2.069706000 -2.846926000

P 0.899029000 -0.994747000 1.571653000

Pd -1.106187000 0.259071000 0.921629000

C 0.413933000 -2.170007000 2.905979000

C 2.108392000 0.012563000 2.539800000

C 1.908133000 4.169725000 -2.023658000

H 2.064655000 4.348201000 -3.095581000

H 0.888370000 4.503801000 -1.792639000

H 2.610896000 4.808299000 -1.476925000

C 4.548150000 0.536734000 0.220031000

H 5.342055000 0.197947000 -0.460337000

H 4.950990000 1.376080000 0.797799000

H 4.353213000 -0.292090000 0.908292000

C 0.442131000 -0.604740000 -2.501683000

H 0.978613000 -1.237284000 -3.222248000

H 0.001745000 -1.279284000 -1.756329000

H -0.378555000 -0.114780000 -3.038192000

H 2.951237000 -0.606878000 2.867638000

H 2.469988000 0.871827000 1.967455000

H 1.578272000 0.385718000 3.423052000

H -0.124262000 -1.579190000 3.656495000

H 1.284909000 -2.633115000 3.384943000

H -0.265301000 -2.946319000 2.539974000

C -0.030844000 3.118150000 1.129605000

O -0.608125000 3.246640000 -0.051079000

O -0.116321000 2.090074000 1.799861000

C 0.751709000 4.304113000 1.564034000

H 0.633207000 4.456984000 2.639253000

H 1.811833000 4.094076000 1.370509000

H 0.467521000 5.202799000 1.013113000

**IM4**

E = -1517.22702662 a.u.

C 3.674470000 -2.235229000 -1.928068000

C 2.476223000 -1.583687000 -1.619631000

C 2.399554000 -0.695478000 -0.542425000

C 3.570868000 -0.428925000 0.196590000

C 4.759886000 -1.090787000 -0.107374000

C 4.812814000 -1.999163000 -1.164395000

H 3.711663000 -2.927813000 -2.767811000

H 1.601165000 -1.780779000 -2.240044000

H 5.660130000 -0.885394000 0.473530000

H 5.745053000 -2.510277000 -1.398390000

C 3.406590000 0.598358000 1.285740000

C 4.670224000 1.420124000 1.536026000

H 5.042599000 1.873111000 0.607340000

H 4.456976000 2.232888000 2.244238000

H 5.481701000 0.815784000 1.965886000

C 3.009321000 -0.097253000 2.592397000

H 2.844569000 0.641263000 3.390888000

H 2.079836000 -0.671289000 2.467734000

H 3.795854000 -0.787685000 2.928190000

C 2.259904000 1.495958000 0.800422000

H 1.867675000 2.107137000 1.629754000

H 2.617667000 2.184332000 0.016380000

C -2.497008000 -1.459404000 0.337129000

C -3.130158000 -2.622744000 0.782414000

C -4.359112000 -2.558086000 1.434080000

C -4.972048000 -1.327257000 1.645414000

C -4.353233000 -0.161973000 1.203654000

C -3.120151000 -0.216467000 0.553765000

H -2.669275000 -3.596014000 0.624230000

H -4.836746000 -3.473652000 1.776070000

H -5.931373000 -1.273241000 2.155356000

H -4.823480000 0.807731000 1.365695000

C -2.434685000 1.010893000 0.079962000

C -2.732567000 1.553255000 -1.170981000

C -1.402078000 1.563971000 0.881155000

C -1.944708000 2.608442000 -1.659654000

C -0.638931000 2.608575000 0.350815000

C -0.887401000 3.130286000 -0.932323000

H -2.167908000 3.018863000 -2.645539000

H 0.108473000 3.091478000 0.978466000

P -0.869303000 -1.492370000 -0.554952000

Pd 0.731968000 0.312414000 0.007545000

C -0.217694000 -3.158279000 -0.121925000

C -1.414225000 -1.770654000 -2.299255000

C -0.013828000 4.218361000 -1.472037000

H -0.414528000 4.636912000 -2.401311000

H 0.104988000 5.035432000 -0.749876000

H 0.994583000 3.836931000 -1.685800000

C -3.866322000 1.025622000 -1.999445000

H -4.835808000 1.371201000 -1.615715000

H -3.785641000 1.366580000 -3.037140000

H -3.902936000 -0.070508000 -2.001440000

C -1.231545000 1.141959000 2.315362000

H -2.101033000 1.447677000 2.913848000

H -1.139588000 0.054320000 2.426810000

H -0.342310000 1.604255000 2.757877000

H -2.198280000 -2.536661000 -2.335560000

H -1.789356000 -0.838737000 -2.734407000

H -0.556472000 -2.108909000 -2.891730000

H 0.750243000 -3.287545000 -0.617328000

H -0.884439000 -3.964142000 -0.448279000

H -0.059934000 -3.221251000 0.960797000

**IM5A**

E = -2134.12749392 a.u.

C -0.944337000 4.354327000 -0.308412000

C -0.694749000 3.008387000 -0.588750000

C 0.593310000 2.469690000 -0.494056000

C 1.648847000 3.333355000 -0.122682000

C 1.396590000 4.675098000 0.164467000

C 0.102091000 5.187283000 0.074761000

H -1.957885000 4.746576000 -0.389565000

H -1.531621000 2.377325000 -0.889587000

H 2.216965000 5.333315000 0.454564000

H -0.085818000 6.236338000 0.297142000

C 3.009700000 2.683336000 -0.046807000

C 3.260303000 2.193770000 1.384070000

H 2.483555000 1.484800000 1.705451000

H 4.232756000 1.683871000 1.455516000

H 3.267492000 3.033651000 2.093119000

C 4.150722000 3.621121000 -0.442395000

H 5.096513000 3.063144000 -0.485454000

H 3.979096000 4.068179000 -1.430844000

H 4.289217000 4.438939000 0.278752000

C 2.935670000 1.491514000 -1.009354000

H 2.995204000 1.848901000 -2.051163000

H 3.746580000 0.764501000 -0.842119000

C -1.849760000 -0.073768000 1.244021000

C -0.847948000 0.245066000 2.169208000

C -1.143984000 0.515051000 3.500981000

C -2.467346000 0.485080000 3.925503000

C -3.472194000 0.183114000 3.013771000

C -3.189523000 -0.103676000 1.672757000

H 0.187258000 0.297938000 1.826142000

H -0.343926000 0.757940000 4.197394000

H -2.719448000 0.701809000 4.961263000

H -4.513180000 0.163947000 3.336334000

C -4.336867000 -0.413877000 0.775748000

C -4.802867000 -1.732388000 0.656001000

C -4.986330000 0.629218000 0.089820000

C -5.862128000 -1.999981000 -0.215894000

C -6.047941000 0.323780000 -0.758907000

C -6.494077000 -0.987897000 -0.934915000

H -6.211023000 -3.028465000 -0.322382000

H -6.543270000 1.132270000 -1.299789000

P -1.177756000 -0.475727000 -0.474403000

Pd 1.114108000 0.526026000 -0.818916000

C -1.241767000 -2.349067000 -0.527232000

C -2.454371000 -0.091340000 -1.790795000

C -7.638813000 -1.284531000 -1.854617000

H -7.782954000 -2.362574000 -1.983383000

H -8.578213000 -0.868169000 -1.468113000

H -7.483300000 -0.841363000 -2.845970000

C -4.228658000 -2.844992000 1.483528000

H -4.651306000 -2.832248000 2.498068000

H -4.462480000 -3.822198000 1.046751000

H -3.141767000 -2.773463000 1.600792000

C -4.563760000 2.056847000 0.273112000

H -3.473841000 2.179440000 0.236663000

H -5.006423000 2.700552000 -0.494905000

H -4.883848000 2.442792000 1.250946000

C 1.944282000 -1.477180000 -1.804556000

C 2.129455000 -1.568137000 -0.591973000

C 1.948498000 -1.643727000 -3.269483000

C 0.646403000 -1.139932000 -3.890848000

H 0.496670000 -0.072148000 -3.682477000

H 0.683117000 -1.278481000 -4.978944000

H -0.219349000 -1.694809000 -3.506783000

C 3.138313000 -0.873591000 -3.850405000

H 4.078780000 -1.192017000 -3.383911000

H 3.203558000 -1.063744000 -4.929275000

H 3.024369000 0.206500000 -3.698923000

C 2.116817000 -3.142093000 -3.553396000

H 3.058653000 -3.519615000 -3.137115000

H 1.291217000 -3.722713000 -3.124341000

H 2.128112000 -3.307730000 -4.638177000

C 2.286015000 -1.693424000 0.808534000

H 1.354140000 -1.626485000 1.373743000

C 3.444169000 -1.906069000 1.481336000

C 3.455048000 -1.946368000 2.967113000

O 4.505464000 -2.107030000 3.572395000

C 4.727947000 -2.098675000 0.729015000

O 4.993274000 -1.398660000 -0.233651000

C 2.168963000 -1.719632000 3.712757000

H 1.790229000 -0.708036000 3.516973000

H 1.386396000 -2.427340000 3.416416000

H 2.363877000 -1.823528000 4.782429000

C 5.637630000 -3.210268000 1.157607000

H 6.252075000 -2.873216000 1.998849000

H 5.076486000 -4.084384000 1.504816000

H 6.287969000 -3.480807000 0.321207000

H -0.826387000 -2.676790000 -1.486931000

H -0.640513000 -2.771702000 0.284113000

H -2.274725000 -2.703807000 -0.449348000

H -1.937875000 -0.208392000 -2.751071000

H -3.299002000 -0.785084000 -1.746765000

H -2.814770000 0.938278000 -1.715688000

**TS_5-6A_**

E = -2134.11201306 a.u.

C -1.519795000 -4.348102000 -2.551284000

C -1.083777000 -3.152504000 -1.973338000

C -1.821396000 -2.492722000 -0.980527000

C -3.044423000 -3.088058000 -0.586011000

C -3.475977000 -4.287382000 -1.155298000

C -2.717808000 -4.920543000 -2.139509000

H -0.918791000 -4.829199000 -3.322351000

H -0.139788000 -2.733069000 -2.318696000

H -4.417382000 -4.735665000 -0.833673000

H -3.064954000 -5.853163000 -2.581545000

C -3.803692000 -2.365011000 0.501537000

C -3.394363000 -2.942221000 1.861307000

H -2.310239000 -2.854864000 2.019819000

H -3.901628000 -2.410482000 2.680106000

H -3.661437000 -4.005911000 1.934581000

C -5.323103000 -2.482912000 0.367278000

H -5.814516000 -1.842233000 1.112830000

H -5.664007000 -2.163400000 -0.626864000

H -5.682355000 -3.507960000 0.534801000

C -3.376159000 -0.899406000 0.403124000

H -3.873422000 -0.415074000 -0.456376000

H -3.671804000 -0.339284000 1.305037000

C 2.163220000 -1.563087000 0.252509000

C 1.553693000 -2.734804000 0.725302000

C 2.270409000 -3.710294000 1.409685000

C 3.626324000 -3.520973000 1.647902000

C 4.244900000 -2.362623000 1.192673000

C 3.541200000 -1.376217000 0.492013000

H 0.485599000 -2.877223000 0.553368000

H 1.766549000 -4.608682000 1.759216000

H 4.202334000 -4.270138000 2.186677000

H 5.308260000 -2.206264000 1.374731000

C 4.312490000 -0.183923000 0.040187000

C 4.452239000 0.922553000 0.894789000

C 4.947789000 -0.191268000 -1.213642000

C 5.169215000 2.035029000 0.453274000

C 5.646020000 0.945252000 -1.624325000

C 5.763005000 2.070955000 -0.808304000

H 5.262037000 2.900081000 1.111672000

H 6.123487000 0.946240000 -2.605938000

P 1.005723000 -0.448890000 -0.685712000

Pd -1.345753000 -0.712758000 -0.029318000

C 1.544176000 1.303476000 -0.504560000

C 1.493731000 -0.693905000 -2.452817000

C 6.536131000 3.269292000 -1.267909000

H 6.319945000 4.148674000 -0.651686000

H 7.617901000 3.088844000 -1.215214000

H 6.308340000 3.518206000 -2.311196000

C 3.836604000 0.919678000 2.262029000

H 4.327363000 0.191033000 2.921407000

H 3.919309000 1.904306000 2.735422000

H 2.774410000 0.641324000 2.234701000

C 4.935597000 -1.411615000 -2.086954000

H 3.976970000 -1.940527000 -2.060533000

H 5.159794000 -1.156040000 -3.128551000

H 5.696703000 -2.132278000 -1.756214000

C -1.270134000 1.020865000 1.251140000

C -1.747551000 1.963341000 0.499945000

C -0.893758000 0.962640000 2.706648000

C -0.191974000 -0.344209000 3.067086000

H 0.749874000 -0.458769000 2.513917000

H 0.044236000 -0.353261000 4.139556000

H -0.825146000 -1.214073000 2.847568000

C 0.025994000 2.136215000 3.069873000

H -0.456058000 3.100258000 2.884988000

H 0.284563000 2.070851000 4.135117000

H 0.964513000 2.103280000 2.500841000

C -2.196043000 1.070714000 3.516164000

H -2.721288000 2.010252000 3.309830000

H -2.873976000 0.236289000 3.295867000

H -1.954808000 1.034140000 4.586778000

C -2.204688000 2.418380000 -0.739673000

H -2.208132000 1.707598000 -1.565288000

C -2.614327000 3.720362000 -0.866877000

C -3.118617000 4.322746000 -2.112721000

O -3.453381000 5.498569000 -2.154463000

C -2.484492000 4.407930000 0.406107000

O -2.044128000 3.707607000 1.343613000

C -3.206049000 3.440450000 -3.325252000

H -2.212955000 3.072706000 -3.611364000

H -3.833438000 2.562700000 -3.129617000

H -3.630269000 4.010924000 -4.154474000

C -2.828624000 5.831837000 0.637960000

H -2.245445000 6.473945000 -0.030328000

H -3.880453000 6.013423000 0.394222000

H -2.631873000 6.093867000 1.680076000

H 0.811212000 1.905121000 -1.056939000

H 1.524053000 1.620861000 0.542667000

H 2.535120000 1.482648000 -0.930509000

H 0.725703000 -0.234990000 -3.087167000

H 2.456260000 -0.208548000 -2.652054000

H 1.572585000 -1.755291000 -2.705288000

**IM6A**

E = -2134.13686299 a.u.

C -1.538434000 -4.385088000 -2.553051000

C -1.077697000 -3.193847000 -1.984966000

C -1.782882000 -2.512215000 -0.980921000

C -3.008219000 -3.093914000 -0.567541000

C -3.467132000 -4.289777000 -1.123902000

C -2.737036000 -4.939015000 -2.118984000

H -0.957696000 -4.877162000 -3.333071000

H -0.132387000 -2.793289000 -2.350576000

H -4.408767000 -4.724626000 -0.784261000

H -3.106150000 -5.868317000 -2.550535000

C -3.748681000 -2.366442000 0.533199000

C -3.323513000 -2.947557000 1.886401000

H -2.236406000 -2.870120000 2.028197000

H -3.813186000 -2.412526000 2.713958000

H -3.598387000 -4.009061000 1.963411000

C -5.270989000 -2.482051000 0.421347000

H -5.750993000 -1.841096000 1.174143000

H -5.626234000 -2.161576000 -0.567519000

H -5.630392000 -3.506202000 0.593979000

C -3.331093000 -0.897824000 0.436969000

H -3.818695000 -0.421911000 -0.432415000

H -3.660716000 -0.343379000 1.329542000

C 2.194813000 -1.545599000 0.253780000

C 1.601057000 -2.724158000 0.730075000

C 2.326497000 -3.677377000 1.435780000

C 3.674098000 -3.456956000 1.694179000

C 4.276104000 -2.291049000 1.236449000

C 3.564147000 -1.326756000 0.513118000

H 0.538492000 -2.888993000 0.543715000

H 1.835762000 -4.582259000 1.787191000

H 4.256679000 -4.187856000 2.250680000

H 5.332940000 -2.110994000 1.433776000

C 4.319455000 -0.126593000 0.054801000

C 4.422502000 0.997834000 0.890894000

C 4.974824000 -0.143338000 -1.188767000

C 5.125332000 2.116038000 0.440583000

C 5.659145000 0.998387000 -1.607956000

C 5.740666000 2.140619000 -0.810747000

H 5.190465000 2.994584000 1.084306000

H 6.153204000 0.990499000 -2.581276000

P 1.027893000 -0.469131000 -0.715227000

Pd -1.287277000 -0.713791000 -0.008535000

C 1.553665000 1.291638000 -0.606909000

C 1.502407000 -0.780384000 -2.474889000

C 6.499535000 3.344681000 -1.279090000

H 6.270520000 4.226570000 -0.671138000

H 7.583359000 3.178456000 -1.222776000

H 6.270982000 3.581768000 -2.325028000

C 3.786884000 1.008157000 2.249007000

H 4.262467000 0.279992000 2.919844000

H 3.870223000 1.995276000 2.717116000

H 2.722481000 0.739500000 2.210200000

C 4.996542000 -1.376754000 -2.043451000

H 4.041732000 -1.913675000 -2.037795000

H 5.247368000 -1.133401000 -3.081890000

H 5.753490000 -2.087100000 -1.682118000

C -1.290369000 0.991374000 1.132277000

C -1.829037000 2.132057000 0.526062000

C -0.918415000 1.010770000 2.602411000

C -0.203773000 -0.279520000 3.004120000

H 0.740004000 -0.399221000 2.455216000

H 0.030896000 -0.260867000 4.077115000

H -0.824914000 -1.162863000 2.805678000

C 0.009079000 2.186961000 2.957232000

H -0.487142000 3.156942000 2.874598000

H 0.350511000 2.068237000 3.994102000

H 0.903613000 2.203706000 2.320786000

C -2.210629000 1.118872000 3.433837000

H -2.767439000 2.037917000 3.222585000

H -2.872713000 0.262844000 3.252648000

H -1.946659000 1.116995000 4.499792000

C -2.235705000 2.337081000 -0.788212000

H -2.179425000 1.578019000 -1.562658000

C -2.701884000 3.653266000 -0.911797000

C -3.242983000 4.342686000 -2.094505000

O -3.584119000 5.514315000 -2.028996000

C -2.571950000 4.221749000 0.355816000

O -2.063701000 3.345591000 1.207598000

C -3.352009000 3.538658000 -3.353222000

H -2.360226000 3.195601000 -3.673941000

H -3.960021000 2.641733000 -3.183219000

H -3.801267000 4.143996000 -4.143543000

C -2.902703000 5.561861000 0.863208000

H -2.385160000 6.329354000 0.278687000

H -3.975376000 5.756435000 0.751228000

H -2.626121000 5.651474000 1.916342000

H 0.804877000 1.863759000 -1.169321000

H 1.540216000 1.642586000 0.429348000

H 2.536605000 1.468650000 -1.052027000

H 0.723479000 -0.361040000 -3.123053000

H 2.453781000 -0.284614000 -2.700253000

H 1.601546000 -1.849493000 -2.683370000

**TS_6-7A_**

E = -2134.11506541 a.u.

C -2.164982000 -4.005035000 -2.650891000

C -1.248353000 -3.017306000 -2.285334000

C -1.233923000 -2.513199000 -0.990260000

C -2.145935000 -2.979047000 -0.000350000

C -3.063918000 -3.952959000 -0.415755000

C -3.074992000 -4.466119000 -1.712221000

H -2.165685000 -4.396015000 -3.666772000

H -0.559524000 -2.637749000 -3.035502000

H -3.796773000 -4.338701000 0.287832000

H -3.802630000 -5.229978000 -1.978531000

C -2.135037000 -2.465763000 1.467373000

C -0.776295000 -2.808967000 2.097530000

H 0.060214000 -2.240494000 1.667762000

H -0.790725000 -2.613900000 3.176616000

H -0.560634000 -3.876479000 1.949673000

C -3.177618000 -3.222329000 2.299280000

H -3.110311000 -2.893190000 3.343680000

H -4.203838000 -3.032320000 1.960197000

H -3.003477000 -4.305359000 2.286304000

C -2.573751000 -0.936557000 1.624483000

H -3.524306000 -0.892711000 1.076894000

H -2.852797000 -0.853631000 2.686030000

C 2.649382000 -1.461230000 -0.554823000

C 2.470427000 -2.853333000 -0.562279000

C 3.420988000 -3.708856000 -0.024139000

C 4.575280000 -3.178761000 0.544879000

C 4.756152000 -1.802731000 0.569910000

C 3.809300000 -0.921307000 0.031002000

H 1.560714000 -3.275580000 -0.991207000

H 3.257349000 -4.783595000 -0.046882000

H 5.329477000 -3.835282000 0.972649000

H 5.653689000 -1.379008000 1.020127000

C 4.087790000 0.536910000 0.141898000

C 3.696679000 1.226365000 1.302583000

C 4.770014000 1.208624000 -0.882985000

C 3.931091000 2.598339000 1.386158000

C 4.996832000 2.580905000 -0.758825000

C 4.575250000 3.295190000 0.361690000

H 3.608172000 3.136211000 2.279855000

H 5.514687000 3.106067000 -1.562982000

P 1.259282000 -0.547490000 -1.359576000

Pd -0.635031000 -0.812270000 -0.178721000

C 1.571084000 1.250133000 -1.525050000

C 1.409116000 -1.068280000 -3.112711000

C 4.829513000 4.766772000 0.484777000

H 3.923972000 5.307032000 0.786283000

H 5.590366000 4.976523000 1.247895000

H 5.182937000 5.194077000 -0.459717000

C 3.056803000 0.499827000 2.447219000

H 3.778559000 -0.158159000 2.950623000

H 2.671059000 1.201283000 3.195133000

H 2.230100000 -0.144082000 2.117616000

C 5.231933000 0.473714000 -2.104876000

H 4.397078000 -0.010885000 -2.630030000

H 5.724984000 1.150141000 -2.810593000

H 5.940768000 -0.325154000 -1.850456000

C -1.737366000 0.360705000 1.266954000

C -2.546510000 1.256556000 0.407740000

C -1.215627000 1.130126000 2.524865000

C -0.525285000 0.202035000 3.523216000

H 0.293556000 -0.357279000 3.053593000

H -0.099252000 0.792104000 4.347228000

H -1.214998000 -0.523353000 3.973889000

C -0.191875000 2.190978000 2.112102000

H -0.618021000 2.924037000 1.416143000

H 0.169632000 2.743719000 2.992620000

H 0.675150000 1.727773000 1.619179000

C -2.357293000 1.848356000 3.260700000

H -2.808377000 2.630195000 2.634579000

H -3.156359000 1.154288000 3.555269000

H -1.989055000 2.332000000 4.176835000

C -2.243626000 2.206508000 -0.524823000

H -1.247586000 2.410205000 -0.901752000

C -3.463313000 2.864228000 -0.911209000

C -3.627845000 3.941597000 -1.890260000

O -4.716638000 4.442482000 -2.151349000

C -4.463471000 2.245497000 -0.199232000

O -3.931921000 1.294053000 0.593100000

C -2.374184000 4.417407000 -2.572316000

H -1.656536000 4.799590000 -1.835493000

H -1.879539000 3.590278000 -3.097313000

H -2.618439000 5.208714000 -3.285231000

C -5.931804000 2.390349000 -0.165497000

H -6.289026000 2.549809000 0.859508000

H -6.228463000 3.237017000 -0.786472000

H -6.425267000 1.484741000 -0.542283000

H 0.713659000 1.642011000 -2.087073000

H 1.612864000 1.741936000 -0.548901000

H 2.488432000 1.449065000 -2.089387000

H 0.577269000 -0.645984000 -3.687233000

H 2.353731000 -0.675266000 -3.508025000

H 1.416082000 -2.156187000 -3.214523000

**IM7A**

E = -2134.18010177 a.u.

C -2.013825000 3.083849000 2.474965000

C -1.500478000 1.870298000 2.015495000

C -1.301093000 1.637274000 0.650600000

C -1.688257000 2.613596000 -0.286809000

C -2.172188000 3.837177000 0.196044000

C -2.327280000 4.080406000 1.559286000

H -2.159123000 3.244658000 3.542322000

H -1.244170000 1.099568000 2.743015000

H -2.450208000 4.624340000 -0.501983000

H -2.708935000 5.041872000 1.898014000

C -1.606714000 2.316632000 -1.779833000

C -0.142463000 2.276133000 -2.232110000

H 0.447530000 1.532813000 -1.679116000

H -0.073189000 2.046914000 -3.304727000

H 0.324295000 3.258203000 -2.069369000

C -2.293627000 3.388632000 -2.626994000

H -2.268169000 3.092942000 -3.683921000

H -3.346281000 3.525982000 -2.346824000

H -1.789305000 4.360406000 -2.551610000

C -2.378108000 0.993480000 -2.056720000

H -3.435572000 1.244921000 -1.893849000

H -2.296442000 0.801660000 -3.136361000

C 2.724863000 1.300937000 0.681801000

C 2.269184000 2.629487000 0.691819000

C 2.979111000 3.647472000 0.070158000

C 4.166232000 3.350318000 -0.593648000

C 4.622299000 2.039550000 -0.619783000

C 3.920903000 0.998988000 0.005732000

H 1.328196000 2.873221000 1.187873000

H 2.601410000 4.667159000 0.098827000

H 4.731426000 4.134997000 -1.091544000

H 5.548745000 1.796092000 -1.139909000

C 4.498948000 -0.370425000 -0.096637000

C 4.023713000 -1.264718000 -1.071777000

C 5.533989000 -0.757739000 0.768060000

C 4.590135000 -2.534913000 -1.163223000

C 6.072625000 -2.041112000 0.649978000

C 5.618426000 -2.942654000 -0.310217000

H 4.215922000 -3.228160000 -1.918918000

H 6.869380000 -2.343839000 1.331154000

P 1.548763000 0.112462000 1.479883000

Pd -0.426450000 -0.082149000 0.080212000

C 2.387258000 -1.482366000 1.855669000

C 1.470509000 0.794879000 3.192245000

C 6.210557000 -4.313505000 -0.435067000

H 5.445657000 -5.092689000 -0.326546000

H 6.670670000 -4.460560000 -1.420627000

H 6.981176000 -4.489656000 0.323038000

C 2.884807000 -0.880316000 -1.966750000

H 3.102845000 0.021874000 -2.555399000

H 2.638807000 -1.689497000 -2.663699000

H 1.977246000 -0.655940000 -1.382578000

C 6.033596000 0.167264000 1.837884000

H 5.215241000 0.519013000 2.481788000

H 6.771343000 -0.332040000 2.474626000

H 6.507346000 1.065219000 1.420540000

C -2.037457000 -0.276418000 -1.260727000

C -3.202259000 -0.730935000 -0.449781000

C -1.487327000 -1.477772000 -2.127166000

C -0.518034000 -1.020724000 -3.219623000

H 0.272870000 -0.371720000 -2.828871000

H -0.037859000 -1.899526000 -3.672098000

H -1.024235000 -0.485076000 -4.031656000

C -0.724926000 -2.443576000 -1.205261000

H -1.359174000 -2.842305000 -0.405401000

H -0.337308000 -3.297169000 -1.780672000

H 0.187735000 -1.998740000 -0.746176000

C -2.610933000 -2.261412000 -2.812272000

H -3.270137000 -2.762095000 -2.090884000

H -3.231757000 -1.608373000 -3.439969000

H -2.183300000 -3.038454000 -3.462067000

C -3.352980000 -1.364149000 0.745876000

H -2.550530000 -1.585351000 1.441517000

C -4.751602000 -1.661923000 0.913786000

C -5.396563000 -2.329185000 2.048698000

O -6.602787000 -2.545381000 2.091475000

C -5.376240000 -1.167826000 -0.206080000

O -4.461343000 -0.611789000 -1.024374000

C -4.492901000 -2.744316000 3.176210000

H -3.727760000 -3.444288000 2.817548000

H -3.962501000 -1.875413000 3.585285000

H -5.078169000 -3.219889000 3.966955000

C -6.782992000 -1.111473000 -0.647896000

H -7.412548000 -1.612464000 0.089388000

H -7.119450000 -0.072725000 -0.759023000

H -6.909543000 -1.599509000 -1.622460000

H 1.691303000 -2.026285000 2.506735000

H 2.551438000 -2.080933000 0.955349000

H 3.334146000 -1.344929000 2.388916000

H 0.855071000 0.127712000 3.806617000

H 2.482099000 0.839319000 3.614000000

H 1.028601000 1.794499000 3.212362000

**TS_4-4B_**

E = -1517.21253785 a.u.

C 5.882736000 -0.715721000 0.580184000

C 4.845830000 0.168885000 0.307504000

C 3.640043000 -0.237772000 -0.297503000

C 3.482366000 -1.620047000 -0.569181000

C 4.540574000 -2.499081000 -0.311300000

C 5.735464000 -2.057831000 0.244306000

H 6.803205000 -0.356880000 1.036852000

H 4.993590000 1.224017000 0.538463000

H 4.418814000 -3.561182000 -0.524368000

H 6.542035000 -2.764631000 0.429946000

C 2.131502000 -2.190747000 -0.969210000

C 1.591776000 -3.003458000 0.215884000

H 1.506515000 -2.380511000 1.116989000

H 0.593008000 -3.399517000 -0.018487000

H 2.242808000 -3.853770000 0.455529000

C 2.226498000 -3.106658000 -2.193787000

H 1.223944000 -3.448526000 -2.486444000

H 2.666318000 -2.579111000 -3.050249000

H 2.831992000 -4.001294000 -1.998397000

C 1.145455000 -1.062676000 -1.270887000

H 1.287738000 -0.661692000 -2.290226000

H 0.114952000 -1.439057000 -1.204138000

C -1.432616000 -0.134581000 1.328296000

C -0.543991000 -0.432230000 2.371439000

C -0.931605000 -1.203980000 3.460059000

C -2.226937000 -1.707252000 3.514406000

C -3.116861000 -1.421647000 2.486246000

C -2.747811000 -0.633279000 1.390050000

H 0.479184000 -0.053527000 2.320685000

H -0.220892000 -1.415831000 4.255434000

H -2.544388000 -2.321158000 4.354351000

H -4.134818000 -1.809541000 2.521731000

C -3.780450000 -0.367482000 0.351934000

C -3.927103000 -1.254971000 -0.729743000

C -4.633449000 0.739191000 0.477983000

C -4.889911000 -0.985952000 -1.699542000

C -5.583047000 0.978533000 -0.519506000

C -5.722907000 0.132515000 -1.617477000

H -4.993643000 -1.668320000 -2.544941000

H -6.236139000 1.847919000 -0.429123000

P -0.703846000 0.970437000 0.036647000

Pd 1.561691000 0.587757000 -0.158461000

C -1.614157000 0.889663000 -1.552600000

C -1.111846000 2.663178000 0.615881000

C -6.752550000 0.391705000 -2.675051000

H -6.314497000 0.353312000 -3.679820000

H -7.546742000 -0.365779000 -2.648994000

H -7.224807000 1.371720000 -2.547043000

C -3.062120000 -2.474792000 -0.841338000

H -3.284116000 -3.196814000 -0.043873000

H -3.212166000 -2.980692000 -1.801034000

H -1.994553000 -2.232781000 -0.746442000

C -4.576585000 1.637937000 1.678526000

H -3.551572000 1.830483000 2.016769000

H -5.057465000 2.600844000 1.474012000

H -5.102792000 1.185538000 2.530843000

H -1.030410000 1.493342000 -2.258216000

H -1.672765000 -0.132774000 -1.935635000

H -2.618651000 1.314921000 -1.468168000

H -0.655887000 3.387136000 -0.068042000

H -0.709682000 2.820479000 1.621720000

H -2.197538000 2.812829000 0.621195000

C 3.108397000 2.976574000 -0.279846000

C 3.642506000 4.374125000 -0.176658000

H 3.127028000 4.942030000 0.600541000

H 4.712677000 4.330996000 0.056613000

H 3.543589000 4.886137000 -1.139175000

O 3.560978000 2.256623000 -1.234428000

H 3.338536000 0.978617000 -0.920219000

O 2.274310000 2.567979000 0.568985000

**IM4B**

E = -1517.22919094 a.u.

C 4.172824000 1.925026000 -0.592661000

C 2.833743000 1.542281000 -0.719501000

C 2.393596000 0.267634000 -0.345307000

C 3.371300000 -0.648009000 0.106274000

C 4.706434000 -0.268525000 0.244915000

C 5.108475000 1.023772000 -0.094899000

H 4.480138000 2.929071000 -0.886368000

H 2.123258000 2.267179000 -1.117009000

H 5.446433000 -0.983105000 0.609291000

H 6.151848000 1.316571000 0.011757000

C 2.849147000 -2.035321000 0.397216000

C 2.437002000 -2.149923000 1.868654000

H 1.696462000 -1.381533000 2.134185000

H 1.991657000 -3.135426000 2.071376000

H 3.305657000 -2.029014000 2.530678000

C 3.856878000 -3.142192000 0.084096000

H 3.383170000 -4.128003000 0.189840000

H 4.239186000 -3.057716000 -0.941716000

H 4.715998000 -3.122137000 0.768269000

C 1.626972000 -2.195700000 -0.509180000

H 1.925100000 -2.268646000 -1.566001000

H 1.046876000 -3.093681000 -0.250700000

C -3.009754000 -0.804746000 -0.010142000

C -4.277484000 -1.392732000 0.053945000

C -5.399528000 -0.630095000 0.348261000

C -5.257177000 0.735522000 0.585441000

C -4.000761000 1.323958000 0.520951000

C -2.857895000 0.568005000 0.220785000

H -4.386839000 -2.462090000 -0.126519000

H -6.380486000 -1.097386000 0.396413000

H -6.127696000 1.343573000 0.821661000

H -3.889766000 2.392958000 0.702777000

C -1.565585000 1.325322000 0.163711000

C -1.247442000 2.045012000 -1.012166000

C -0.873909000 1.615285000 1.363492000

C -0.307455000 3.073948000 -0.948505000

C 0.069570000 2.643124000 1.375929000

C 0.338421000 3.408429000 0.242033000

H -0.079696000 3.635636000 -1.856013000

H 0.593043000 2.864358000 2.307557000

P -1.510368000 -1.798528000 -0.359624000

Pd 0.462938000 -0.506095000 -0.364351000

C -1.610384000 -3.149809000 0.885996000

C -1.907010000 -2.689530000 -1.917325000

C 1.313410000 4.544020000 0.300570000

H 1.687639000 4.806223000 -0.695827000

H 0.848444000 5.445145000 0.722174000

H 2.173069000 4.301403000 0.937125000

C -1.965972000 1.758190000 -2.296635000

H -3.013739000 2.085254000 -2.253538000

H -1.489625000 2.276546000 -3.135321000

H -1.977982000 0.683547000 -2.521446000

C -1.203047000 0.885783000 2.631470000

H -1.268068000 -0.198865000 2.478121000

H -0.444414000 1.074000000 3.398777000

H -2.173657000 1.206168000 3.034747000

H -0.815006000 -3.879152000 0.696666000

H -1.472930000 -2.733815000 1.890259000

H -2.576522000 -3.664708000 0.831562000

H -1.066759000 -3.349094000 -2.165204000

H -2.033288000 -1.965364000 -2.729366000

H -2.817362000 -3.292007000 -1.818196000

**TS_4-5B_**

E = -1746.19288372 a.u.

C 5.882736000 -0.715721000 0.580184000

C 4.845830000 0.168885000 0.307504000

C 3.640043000 -0.237772000 -0.297503000

C 3.482366000 -1.620047000 -0.569181000

C 4.540574000 -2.499081000 -0.311300000

C 5.735464000 -2.057831000 0.244306000

H 6.803205000 -0.356880000 1.036852000

H 4.993590000 1.224017000 0.538463000

H 4.418814000 -3.561182000 -0.524368000

H 6.542035000 -2.764631000 0.429946000

C 2.131502000 -2.190747000 -0.969210000

C 1.591776000 -3.003458000 0.215884000

H 1.506515000 -2.380511000 1.116989000

H 0.593008000 -3.399517000 -0.018487000

H 2.242808000 -3.853770000 0.455529000

C 2.226498000 -3.106658000 -2.193787000

H 1.223944000 -3.448526000 -2.486444000

H 2.666318000 -2.579111000 -3.050249000

H 2.831992000 -4.001294000 -1.998397000

C 1.145455000 -1.062676000 -1.270887000

H 1.287738000 -0.661692000 -2.290226000

H 0.114952000 -1.439057000 -1.204138000

C -1.432616000 -0.134581000 1.328296000

C -0.543991000 -0.432230000 2.371439000

C -0.931605000 -1.203980000 3.460059000

C -2.226937000 -1.707252000 3.514406000

C -3.116861000 -1.421647000 2.486246000

C -2.747811000 -0.633279000 1.390050000

H 0.479184000 -0.053527000 2.320685000

H -0.220892000 -1.415831000 4.255434000

H -2.544388000 -2.321158000 4.354351000

H -4.134818000 -1.809541000 2.521731000

C -3.780450000 -0.367482000 0.351934000

C -3.927103000 -1.254971000 -0.729743000

C -4.633449000 0.739191000 0.477983000

C -4.889911000 -0.985952000 -1.699542000

C -5.583047000 0.978533000 -0.519506000

C -5.722907000 0.132515000 -1.617477000

H -4.993643000 -1.668320000 -2.544941000

H -6.236139000 1.847919000 -0.429123000

P -0.703846000 0.970437000 0.036647000

Pd 1.561691000 0.587757000 -0.158461000

C -1.614157000 0.889663000 -1.552600000

C -1.111846000 2.663178000 0.615881000

C -6.752550000 0.391705000 -2.675051000

H -6.314497000 0.353312000 -3.679820000

H -7.546742000 -0.365779000 -2.648994000

H -7.224807000 1.371720000 -2.547043000

C -3.062120000 -2.474792000 -0.841338000

H -3.284116000 -3.196814000 -0.043873000

H -3.212166000 -2.980692000 -1.801034000

H -1.994553000 -2.232781000 -0.746442000

C -4.576585000 1.637937000 1.678526000

H -3.551572000 1.830483000 2.016769000

H -5.057465000 2.600844000 1.474012000

H -5.102792000 1.185538000 2.530843000

H -1.030410000 1.493342000 -2.258216000

H -1.672765000 -0.132774000 -1.935635000

H -2.618651000 1.314921000 -1.468168000

H -0.655887000 3.387136000 -0.068042000

H -0.709682000 2.820479000 1.621720000

H -2.197538000 2.812829000 0.621195000

C 3.108397000 2.976574000 -0.279846000

C 3.642506000 4.374125000 -0.176658000

H 3.127028000 4.942030000 0.600541000

H 4.712677000 4.330996000 0.056613000

H 3.543589000 4.886137000 -1.139175000

O 3.560978000 2.256623000 -1.234428000

H 3.338536000 0.978617000 -0.920219000

O 2.274310000 2.567979000 0.568985000

**IM5B**

E = -1746.24190784 a.u.

C 6.019734000 -1.288875000 -1.122210000

C 4.936373000 -1.841944000 -0.441767000

C 3.868657000 -1.040213000 -0.058510000

C 3.846704000 0.336465000 -0.334973000

C 4.937742000 0.871202000 -1.026204000

C 6.011988000 0.069899000 -1.413430000

H 6.860061000 -1.912082000 -1.420811000

H 4.922423000 -2.904752000 -0.205015000

H 4.967785000 1.930986000 -1.267444000

H 6.848998000 0.519424000 -1.944682000

C 2.646144000 1.179896000 0.112178000

C 2.862997000 2.667477000 -0.155090000

H 2.964205000 2.878891000 -1.228057000

H 2.004856000 3.237254000 0.218651000

H 3.762529000 3.040995000 0.352863000

C 2.439108000 0.975656000 1.617018000

H 1.578665000 1.557677000 1.968977000

H 2.257740000 -0.076909000 1.865580000

H 3.328133000 1.304194000 2.173389000

C 1.443531000 0.674318000 -0.702629000

H 1.617796000 0.879217000 -1.772401000

H 1.338441000 -0.412634000 -0.590427000

C -2.827042000 -0.627576000 -0.997531000

C -4.081774000 -0.250165000 -1.490343000

C -5.252135000 -0.788727000 -0.964832000

C -5.181883000 -1.718725000 0.064645000

C -3.940536000 -2.094714000 0.567179000

C -2.753987000 -1.555702000 0.064032000

H -4.160961000 0.472440000 -2.298750000

H -6.214546000 -0.479792000 -1.365940000

H -6.089954000 -2.150385000 0.479563000

H -3.877671000 -2.820898000 1.377414000

C -1.469927000 -1.996772000 0.682380000

C -0.976481000 -1.329372000 1.819676000

C -0.796461000 -3.124864000 0.181839000

C 0.221146000 -1.761449000 2.390286000

C 0.410303000 -3.512398000 0.769016000

C 0.937955000 -2.841207000 1.871775000

H 0.605716000 -1.237313000 3.267173000

H 0.942294000 -4.373869000 0.361626000

P -1.318386000 0.161614000 -1.706950000

Pd -0.351460000 1.538008000 -0.205233000

C -0.395258000 -1.138099000 -2.607589000

C -1.909912000 1.207201000 -3.088290000

C 2.222613000 -3.285679000 2.503841000

H 2.740571000 -2.452128000 2.993236000

H 2.044597000 -4.048863000 3.273390000

H 2.903818000 -3.727774000 1.766564000

C -1.735922000 -0.196578000 2.444987000

H -2.624870000 -0.563208000 2.977290000

H -1.113268000 0.338462000 3.171813000

H -2.085124000 0.528053000 1.698510000

C -1.377034000 -3.949093000 -0.929301000

H -1.893302000 -3.342345000 -1.680639000

H -0.600892000 -4.535503000 -1.433549000

H -2.118996000 -4.658423000 -0.536275000

H 0.376371000 -0.646404000 -3.209761000

H 0.084218000 -1.841742000 -1.921753000

H -1.082481000 -1.672190000 -3.272779000

H -1.018043000 1.626119000 -3.567988000

H -2.520235000 2.035586000 -2.714976000

H -2.473094000 0.625739000 -3.827286000

C -1.308517000 3.410835000 1.286288000

C -1.834859000 4.516167000 2.150508000

H -2.901148000 4.385433000 2.352103000

H -1.277928000 4.572916000 3.089903000

H -1.700744000 5.470425000 1.626736000

O -0.063682000 3.153924000 1.303344000

H 3.029464000 -1.498911000 0.465731000

O -2.094077000 2.768320000 0.529820000

**IM6B**

E = -2363.13259573 a.u.

C -2.337660000 6.369689000 0.338013000

C -3.204201000 5.437572000 -0.228830000

C -2.987455000 4.076249000 -0.046527000

C -1.905147000 3.602217000 0.709688000

C -1.044744000 4.553317000 1.269697000

C -1.256891000 5.918774000 1.087347000

H -2.505118000 7.435297000 0.196173000

H -4.056541000 5.771540000 -0.817737000

H -0.187499000 4.235571000 1.859416000

H -0.567960000 6.631686000 1.537599000

C -1.664312000 2.094029000 0.843695000

C -2.967931000 1.412122000 1.266808000

H -3.745598000 1.450921000 0.494581000

H -2.800444000 0.356368000 1.500039000

H -3.371211000 1.885649000 2.172625000

C -0.624467000 1.784877000 1.919623000

H -0.555745000 0.698181000 2.052992000

H 0.378139000 2.150342000 1.662406000

H -0.912575000 2.220012000 2.886792000

C -1.182673000 1.638598000 -0.553973000

H -2.033337000 1.649241000 -1.252469000

H -0.468015000 2.385659000 -0.919788000

C 2.489445000 1.833555000 -0.182886000

C 2.157626000 3.195743000 -0.171433000

C 2.621974000 4.046479000 0.825821000

C 3.425841000 3.540344000 1.840324000

C 3.782300000 2.196655000 1.825247000

C 3.334681000 1.323487000 0.827558000

H 1.521622000 3.607989000 -0.952722000

H 2.346886000 5.099124000 0.808100000

H 3.789438000 4.190523000 2.632783000

H 4.438615000 1.800015000 2.599538000

C 3.860337000 -0.074161000 0.856268000

C 5.124833000 -0.324353000 0.291409000

C 3.144385000 -1.122207000 1.461540000

C 5.625930000 -1.627014000 0.293365000

C 3.680964000 -2.409746000 1.443449000

C 4.915687000 -2.686020000 0.855716000

H 6.598570000 -1.818755000 -0.162333000

H 3.117064000 -3.222631000 1.905627000

P 1.651632000 0.775599000 -1.438760000

Pd -0.335559000 -0.253498000 -0.792737000

C 1.385115000 1.858845000 -2.894499000

C 2.876655000 -0.397896000 -2.128489000

C 5.458255000 -4.082428000 0.846195000

H 6.387170000 -4.149256000 0.269805000

H 5.670291000 -4.435536000 1.863593000

H 4.738079000 -4.787502000 0.411948000

C 5.931156000 0.777043000 -0.331665000

H 6.316404000 1.476243000 0.422630000

H 6.792064000 0.371599000 -0.873697000

H 5.338062000 1.377076000 -1.033829000

C 1.808991000 -0.890625000 2.099154000

H 1.038685000 -0.710952000 1.332999000

H 1.496299000 -1.767800000 2.678041000

H 1.812999000 -0.021572000 2.769547000

H 1.057665000 1.207099000 -3.713208000

H 0.610306000 2.611499000 -2.727519000

H 2.321039000 2.351956000 -3.181962000

H 2.384319000 -0.899437000 -2.969156000

H 3.162110000 -1.147632000 -1.389963000

H 3.761329000 0.144475000 -2.481051000

C 0.711915000 -2.774181000 -2.035608000

C 1.463043000 -4.092324000 -2.007732000

H 1.183935000 -4.696358000 -1.138167000

H 1.300490000 -4.663283000 -2.926271000

H 2.537259000 -3.878988000 -1.921771000

O 0.335288000 -2.302802000 -3.119881000

H -3.677873000 3.368646000 -0.504326000

O 0.579432000 -2.222186000 -0.885424000

C -2.409620000 -1.101244000 -1.271861000

C -2.119649000 -1.510161000 -0.142365000

C -3.072852000 -0.875630000 -2.575409000

C -4.387967000 -0.132542000 -2.321148000

H -4.202134000 0.880263000 -1.940717000

H -4.948368000 -0.046004000 -3.260971000

H -5.011229000 -0.668281000 -1.592769000

C -2.199198000 -0.104362000 -3.564597000

H -1.249604000 -0.630797000 -3.715334000

H -2.729677000 -0.034062000 -4.523421000

H -1.990224000 0.915266000 -3.218601000

C -3.358041000 -2.271168000 -3.150798000

H -2.418949000 -2.812021000 -3.319354000

H -3.991481000 -2.862193000 -2.477275000

H -3.879815000 -2.167014000 -4.110804000

C -1.960499000 -2.098366000 1.140612000

H -0.938381000 -2.238554000 1.495670000

C -3.013468000 -2.471428000 1.895992000

C -2.876697000 -3.095270000 3.232749000

O -3.881398000 -3.498792000 3.801655000

C -4.428924000 -2.259647000 1.416428000

O -5.030163000 -1.246184000 1.711313000

C -1.512232000 -3.203358000 3.843978000

H -0.848204000 -3.810658000 3.217104000

H -1.053470000 -2.211527000 3.942935000

H -1.596590000 -3.664647000 4.830506000

C -5.005218000 -3.355900000 0.581918000

H -4.350410000 -3.557461000 -0.276482000

H -5.036031000 -4.279964000 1.174413000

H -6.012099000 -3.100111000 0.241174000

**TS_6-7B_**

E = -2363.11956029 a.u.

C -3.413027000 6.231543000 -1.242209000

C -3.996768000 5.379033000 -0.307939000

C -3.696824000 4.021271000 -0.312529000

C -2.808750000 3.470248000 -1.248128000

C -2.225799000 4.343943000 -2.173292000

C -2.525369000 5.705114000 -2.173756000

H -3.648883000 7.293569000 -1.242736000

H -4.694250000 5.772916000 0.429193000

H -1.524054000 3.967484000 -2.914674000

H -2.059181000 6.355491000 -2.911744000

C -2.454894000 1.980198000 -1.187671000

C -1.800595000 1.508927000 -2.486905000

H -0.837682000 1.992389000 -2.683303000

H -1.616749000 0.427663000 -2.437277000

H -2.453733000 1.700312000 -3.350152000

C -3.740869000 1.161768000 -1.019349000

H -3.546229000 0.092753000 -1.165623000

H -4.198731000 1.276485000 -0.028935000

H -4.484653000 1.461144000 -1.770238000

C -1.501619000 1.836269000 0.023478000

H -0.772059000 2.656783000 -0.012056000

H -2.081478000 1.977698000 0.947784000

C 2.617022000 1.415456000 0.778601000

C 2.029109000 2.350255000 1.647526000

C 2.673295000 2.788521000 2.795541000

C 3.934795000 2.288208000 3.104700000

C 4.525185000 1.360870000 2.257892000

C 3.890979000 0.906580000 1.091979000

H 1.035092000 2.738472000 1.422631000

H 2.190047000 3.514744000 3.445272000

H 4.456188000 2.617959000 4.000465000

H 5.513000000 0.963183000 2.490416000

C 4.636397000 -0.086495000 0.267792000

C 5.463786000 0.350272000 -0.778351000

C 4.569061000 -1.454114000 0.590485000

C 6.160642000 -0.595055000 -1.534197000

C 5.283441000 -2.366986000 -0.186037000

C 6.075631000 -1.958598000 -1.260930000

H 6.793720000 -0.253391000 -2.354541000

H 5.228835000 -3.428922000 0.062615000

P 1.529336000 1.034565000 -0.674738000

Pd -0.427573000 0.064337000 0.216951000

C 1.546235000 2.655762000 -1.550862000

C 2.382624000 -0.017813000 -1.910205000

C 6.822532000 -2.966204000 -2.080666000

H 7.466891000 -2.482938000 -2.822807000

H 7.452686000 -3.606046000 -1.450346000

H 6.135551000 -3.632509000 -2.617938000

C 5.609622000 1.809911000 -1.088366000

H 6.061100000 2.355154000 -0.248638000

H 6.243818000 1.965189000 -1.967338000

H 4.641002000 2.289147000 -1.282142000

C 3.735750000 -1.934813000 1.740250000

H 2.663589000 -1.793248000 1.536592000

H 3.902979000 -3.002299000 1.923840000

H 3.970258000 -1.392373000 2.664875000

H 1.169799000 2.514021000 -2.569708000

H 0.925915000 3.399675000 -1.042272000

H 2.574885000 3.029680000 -1.608478000

H 1.652443000 -0.181164000 -2.712043000

H 2.663671000 -0.987852000 -1.491284000

H 3.264315000 0.483594000 -2.321801000

C 0.694074000 -2.558502000 -0.648252000

C 1.643058000 -3.737087000 -0.562260000

H 1.590242000 -4.213654000 0.422390000

H 1.433173000 -4.472462000 -1.343611000

H 2.672863000 -3.373968000 -0.691536000

O 0.009739000 -2.393539000 -1.673195000

H -4.165878000 3.377918000 0.430694000

O 0.709019000 -1.788997000 0.377933000

C -1.918145000 -0.684788000 1.519068000

C -2.591816000 -1.506593000 0.782031000

C -1.991323000 -0.304611000 2.971267000

C -3.332023000 0.387601000 3.245549000

H -4.176297000 -0.250653000 2.963267000

H -3.407486000 0.614212000 4.317295000

H -3.411026000 1.335297000 2.697562000

C -1.896832000 -1.602464000 3.788424000

H -0.966403000 -2.141269000 3.564122000

H -1.894475000 -1.356829000 4.858416000

H -2.740617000 -2.268080000 3.583692000

C -0.844424000 0.612104000 3.395307000

H 0.128930000 0.129384000 3.229800000

H -0.855618000 1.564212000 2.851847000

H -0.931614000 0.833149000 4.467673000

C -2.740930000 -2.219027000 -0.412206000

H -1.961473000 -2.086533000 -1.168804000

C -3.794681000 -3.080691000 -0.536842000

C -4.062138000 -3.880520000 -1.744436000

O -5.007695000 -4.654220000 -1.797827000

C -4.602237000 -3.066873000 0.672294000

O -4.172029000 -2.333879000 1.589466000

C -3.128736000 -3.688957000 -2.905939000

H -3.184148000 -2.654772000 -3.270829000

H -2.086269000 -3.866337000 -2.613987000

H -3.408139000 -4.370771000 -3.712395000

C -5.857298000 -3.834627000 0.850800000

H -6.574644000 -3.571374000 0.066359000

H -5.659885000 -4.905376000 0.734040000

H -6.282133000 -3.633945000 1.836756000

**IM7B**

E = -2363.14544585 a.u.

C 1.737075000 6.088406000 -0.148241000

C 1.524353000 5.530778000 1.112420000

C 0.608706000 4.499113000 1.276315000

C -0.126113000 3.988729000 0.194900000

C 0.096095000 4.564704000 -1.059573000

C 1.017312000 5.599703000 -1.231637000

H 2.455605000 6.894620000 -0.280334000

H 2.078564000 5.900646000 1.973164000

H -0.449141000 4.208412000 -1.930759000

H 1.166830000 6.023474000 -2.223159000

C -1.077639000 2.810675000 0.417808000

C -1.854423000 2.479528000 -0.857363000

H -1.227118000 2.072981000 -1.658949000

H -2.624890000 1.735720000 -0.632864000

H -2.371756000 3.368456000 -1.244900000

C -2.127831000 3.207649000 1.465306000

H -2.797891000 2.359797000 1.667134000

H -1.674493000 3.506364000 2.418563000

H -2.744374000 4.044686000 1.109098000

C -0.232050000 1.616198000 0.960711000

H 0.834169000 1.825073000 0.807673000

H -0.370569000 1.545113000 2.049397000

C 2.988700000 0.884657000 -0.643459000

C 3.052960000 2.283309000 -0.727389000

C 4.154236000 2.992208000 -0.258847000

C 5.227369000 2.309614000 0.301255000

C 5.172121000 0.925038000 0.406097000

C 4.062642000 0.197581000 -0.038943000

H 2.227982000 2.847823000 -1.161107000

H 4.165121000 4.078025000 -0.336513000

H 6.099301000 2.850125000 0.662883000

H 5.998740000 0.379080000 0.860508000

C 4.057692000 -1.273402000 0.193419000

C 4.769037000 -2.141786000 -0.650111000

C 3.365514000 -1.784503000 1.306396000

C 4.703911000 -3.516869000 -0.415627000

C 3.318784000 -3.164294000 1.504595000

C 3.967927000 -4.049720000 0.641798000

H 5.244012000 -4.191489000 -1.081890000

H 2.777974000 -3.556316000 2.368340000

P 1.449096000 0.055025000 -1.276562000

Pd -0.451460000 -0.328381000 0.201864000

C 1.025590000 1.115620000 -2.723523000

C 2.006646000 -1.435498000 -2.203183000

C 3.874547000 -5.530125000 0.851226000

H 4.695002000 -6.060236000 0.355175000

H 3.893225000 -5.788109000 1.916596000

H 2.935793000 -5.929666000 0.444618000

C 5.596246000 -1.622227000 -1.788316000

H 6.555901000 -1.220929000 -1.434566000

H 5.822087000 -2.419051000 -2.505330000

H 5.099247000 -0.807035000 -2.327913000

C 2.716593000 -0.859932000 2.291998000

H 1.822249000 -0.381037000 1.866310000

H 2.408160000 -1.402364000 3.192125000

H 3.398248000 -0.055317000 2.595903000

H 0.180887000 0.634326000 -3.228354000

H 0.727160000 2.126131000 -2.430591000

H 1.878021000 1.173420000 -3.411159000

H 1.195516000 -1.668918000 -2.900455000

H 2.168325000 -2.291569000 -1.545045000

H 2.914887000 -1.219205000 -2.775932000

C -0.946173000 -2.544674000 -1.708096000

C -0.917872000 -3.978218000 -2.198729000

H -1.491077000 -4.091398000 -3.122936000

H 0.122983000 -4.263534000 -2.400521000

H -1.299062000 -4.666296000 -1.437416000

O -1.283540000 -1.635222000 -2.488460000

H 0.472903000 4.068711000 2.268218000

O -0.565259000 -2.388461000 -0.495878000

C -2.092081000 -0.629410000 1.351633000

C -3.309913000 -0.595505000 0.677668000

C -2.038544000 -0.990862000 2.820817000

C -2.672376000 0.130291000 3.661226000

H -3.727939000 0.292941000 3.421927000

H -2.602870000 -0.144429000 4.722251000

H -2.134825000 1.077533000 3.525077000

C -2.779098000 -2.319242000 3.059258000

H -2.381491000 -3.109396000 2.408037000

H -2.620295000 -2.633575000 4.098696000

H -3.855625000 -2.240966000 2.888826000

C -0.599396000 -1.184677000 3.294154000

H -0.096555000 -1.975143000 2.720227000

H -0.016342000 -0.263166000 3.196156000

H -0.593748000 -1.478607000 4.352709000

C -3.576426000 -0.547613000 -0.689041000

H -2.803263000 -0.534592000 -1.456573000

C -4.962394000 -0.604871000 -0.868975000

C -5.714209000 -0.589535000 -2.135233000

O -6.934993000 -0.635449000 -2.143389000

C -5.514547000 -0.669240000 0.412268000

O -4.560824000 -0.683014000 1.328933000

C -4.896086000 -0.504068000 -3.387378000

H -4.312663000 0.426218000 -3.392410000

H -4.173706000 -1.328573000 -3.434454000

H -5.547345000 -0.530441000 -4.263756000

C -6.909191000 -0.701858000 0.873015000

H -7.502778000 -1.357867000 0.230930000

H -6.965139000 -1.026331000 1.914771000

H -7.352476000 0.299198000 0.791913000

**TS_7-8B_**

E = -2363.13214114 a.u.

C -0.181001000 6.127864000 -1.009579000

C -0.690096000 5.706181000 0.218251000

C -1.325320000 4.475416000 0.326229000

C -1.472034000 3.625324000 -0.781759000

C -0.969663000 4.072174000 -2.007435000

C -0.328090000 5.306733000 -2.120401000

H 0.320717000 7.089207000 -1.097079000

H -0.590798000 6.339739000 1.097606000

H -1.064214000 3.454708000 -2.897743000

H 0.057217000 5.622763000 -3.088002000

C -2.137994000 2.257581000 -0.597649000

C -2.195263000 1.479679000 -1.911461000

H -1.202718000 1.239321000 -2.309239000

H -2.732790000 0.536769000 -1.765334000

H -2.743661000 2.045884000 -2.676594000

C -3.590959000 2.480445000 -0.147647000

H -4.100963000 1.519181000 0.004987000

H -3.650270000 3.043799000 0.791966000

H -4.152222000 3.037309000 -0.909563000

C -1.357299000 1.530857000 0.535848000

H -0.389610000 2.006120000 0.745226000

H -1.912135000 1.695712000 1.470757000

C 2.639111000 1.303218000 -0.085160000

C 2.167627000 2.602100000 0.169331000

C 2.779757000 3.432413000 1.098551000

C 3.889672000 2.973702000 1.802462000

C 4.367862000 1.692406000 1.560006000

C 3.758471000 0.838373000 0.630263000

H 1.294746000 2.977200000 -0.367803000

H 2.387233000 4.433679000 1.266009000

H 4.381909000 3.610391000 2.534152000

H 5.240037000 1.326031000 2.101532000

C 4.354259000 -0.515999000 0.443364000

C 5.421436000 -0.684280000 -0.452931000

C 3.865070000 -1.615034000 1.171520000

C 5.963697000 -1.958820000 -0.632812000

C 4.432310000 -2.871660000 0.965234000

C 5.480154000 -3.065599000 0.062336000

H 6.782294000 -2.090144000 -1.342142000

H 4.047020000 -3.724923000 1.526910000

P 1.564487000 0.318693000 -1.227682000

Pd -0.362722000 -0.345061000 0.096091000

C 1.480894000 1.422389000 -2.704254000

C 2.522117000 -1.061849000 -1.978715000

C 6.060767000 -4.431327000 -0.145089000

H 6.480405000 -4.834445000 0.785345000

H 5.295024000 -5.143092000 -0.479097000

H 6.857965000 -4.419227000 -0.895899000

C 5.952281000 0.475228000 -1.242130000

H 6.421026000 1.229019000 -0.595731000

H 6.702536000 0.146983000 -1.969271000

H 5.152758000 0.993804000 -1.789402000

C 2.720512000 -1.452740000 2.124420000

H 1.791780000 -1.228763000 1.576845000

H 2.548723000 -2.370113000 2.698892000

H 2.891185000 -0.630912000 2.831839000

H 0.911226000 0.905252000 -3.485737000

H 0.982655000 2.370264000 -2.481490000

H 2.493411000 1.623301000 -3.075704000

H 1.891352000 -1.448869000 -2.789045000

H 2.698016000 -1.866313000 -1.261894000

H 3.470228000 -0.714652000 -2.403210000

C -0.055246000 -2.923157000 -1.236415000

C 0.524680000 -4.307929000 -1.442815000

H 0.159706000 -4.758938000 -2.369413000

H 1.619825000 -4.243062000 -1.481084000

H 0.274310000 -4.957868000 -0.597157000

O -0.773519000 -2.410033000 -2.110270000

H -1.704549000 4.160523000 1.298043000

O 0.286840000 -2.377650000 -0.125454000

C -1.887752000 -0.375171000 1.354136000

C -3.110587000 -0.853359000 0.800839000

C -1.696049000 -0.306366000 2.863168000

C -2.851392000 0.326036000 3.652325000

H -3.753458000 -0.289149000 3.650213000

H -2.536391000 0.455153000 4.695871000

H -3.115334000 1.318134000 3.260807000

C -1.553492000 -1.784063000 3.281175000

H -0.691060000 -2.255143000 2.791746000

H -1.399586000 -1.829739000 4.367080000

H -2.449870000 -2.369895000 3.043547000

C -0.409128000 0.419384000 3.264396000

H 0.455040000 0.065191000 2.690803000

H -0.488898000 1.505222000 3.129646000

H -0.211526000 0.237742000 4.328646000

C -3.330710000 -1.485795000 -0.401660000

H -2.551596000 -1.697290000 -1.134023000

C -4.700449000 -1.835735000 -0.461148000

C -5.392813000 -2.523959000 -1.564284000

O -6.574177000 -2.832492000 -1.495971000

C -5.260307000 -1.384283000 0.723900000

O -4.325758000 -0.812531000 1.487025000

C -4.564613000 -2.809304000 -2.782350000

H -4.192851000 -1.869685000 -3.212530000

H -3.680897000 -3.407060000 -2.526994000

H -5.164991000 -3.337458000 -3.526404000

C -6.632311000 -1.414858000 1.256327000

H -7.302513000 -1.856299000 0.517420000

H -6.673509000 -2.006902000 2.178920000

H -6.969751000 -0.401688000 1.504866000

**IM8B**

E = -2363.19118587 a.u.

C 2.602188000 4.608603000 0.026987000

C 1.932861000 4.643684000 1.247827000

C 0.668010000 4.076788000 1.374103000

C 0.031125000 3.466886000 0.285556000

C 0.713205000 3.453048000 -0.937243000

C 1.981583000 4.015340000 -1.066733000

H 3.592862000 5.047688000 -0.072322000

H 2.399434000 5.111072000 2.112928000

H 0.254687000 3.001206000 -1.814999000

H 2.481386000 3.987735000 -2.033489000

C -1.306209000 2.732354000 0.460468000

C -2.035416000 2.612696000 -0.877081000

H -1.561075000 1.892363000 -1.556500000

H -3.074058000 2.298998000 -0.736308000

H -2.056731000 3.590007000 -1.375691000

C -2.220081000 3.474474000 1.439480000

H -3.180117000 2.954278000 1.529490000

H -1.793848000 3.556632000 2.444929000

H -2.416038000 4.490154000 1.072059000

C -0.918646000 1.353464000 1.082656000

H 0.014954000 1.113222000 0.339686000

H -0.346728000 1.546715000 1.999129000

C 3.000088000 0.294883000 -1.773123000

C 3.292458000 1.182554000 -2.815415000

C 4.470702000 1.923421000 -2.825016000

C 5.381247000 1.788629000 -1.783592000

C 5.106140000 0.905930000 -0.745091000

C 3.927880000 0.155225000 -0.719621000

H 2.593635000 1.312745000 -3.638685000

H 4.670622000 2.606797000 -3.647585000

H 6.304358000 2.363910000 -1.779846000

H 5.816518000 0.786489000 0.072962000

C 3.700557000 -0.770727000 0.424620000

C 4.108724000 -2.111654000 0.345961000

C 3.129355000 -0.276023000 1.614448000

C 3.839224000 -2.967027000 1.419236000

C 2.904691000 -1.154055000 2.671677000

C 3.228269000 -2.510254000 2.584032000

H 4.135308000 -4.014740000 1.345821000

H 2.454404000 -0.773396000 3.590511000

P 1.407527000 -0.656591000 -1.776120000

Pd -0.240127000 -0.544682000 -0.011126000

C 0.513106000 -0.013964000 -3.248938000

C 1.869239000 -2.336137000 -2.383263000

C 2.931847000 -3.430338000 3.728251000

H 3.486718000 -3.141380000 4.630060000

H 1.866208000 -3.402326000 3.992053000

H 3.193293000 -4.467091000 3.490795000

C 4.893424000 -2.625957000 -0.825237000

H 5.961792000 -2.401872000 -0.695781000

H 4.800231000 -3.713720000 -0.918839000

H 4.593490000 -2.170958000 -1.774426000

C 2.781714000 1.175806000 1.754574000

H 2.138208000 1.524752000 0.934991000

H 2.263329000 1.366043000 2.702029000

H 3.678039000 1.810565000 1.737269000

H -0.429902000 -0.572196000 -3.287188000

H 0.292074000 1.053062000 -3.133962000

H 1.068546000 -0.180093000 -4.179652000

H 0.953642000 -2.787608000 -2.780382000

H 2.243887000 -2.959738000 -1.565999000

H 2.617800000 -2.269085000 -3.180954000

C -1.056140000 -3.162550000 -1.078791000

C -1.126291000 -4.672316000 -1.028411000

H -1.434957000 -5.024223000 -0.039153000

H -1.805832000 -5.059519000 -1.792183000

H -0.123494000 -5.075149000 -1.222230000

O -1.387722000 -2.555331000 -2.108287000

H 0.180638000 4.104060000 2.347064000

O -0.593492000 -2.623708000 -0.007305000

C -1.785897000 0.162737000 1.250937000

C -3.052228000 0.087633000 0.484481000

C -1.840153000 -0.494867000 2.654449000

C -2.476602000 0.469612000 3.664709000

H -3.493497000 0.757172000 3.375029000

H -2.531647000 -0.010738000 4.651424000

H -1.880315000 1.385292000 3.774638000

C -2.668096000 -1.780224000 2.602055000

H -2.266771000 -2.474780000 1.852952000

H -2.636649000 -2.275657000 3.581393000

H -3.722996000 -1.588260000 2.368291000

C -0.444199000 -0.863984000 3.159771000

H 0.037278000 -1.591956000 2.493124000

H 0.219422000 0.005390000 3.254394000

H -0.523326000 -1.315617000 4.158739000

C -3.442733000 -0.535635000 -0.654805000

H -2.806628000 -1.151144000 -1.286961000

C -4.831157000 -0.215166000 -0.855665000

C -5.690553000 -0.637649000 -1.968493000

O -6.871993000 -0.321819000 -2.043608000

C -5.198219000 0.585821000 0.198781000

O -4.137402000 0.767276000 1.014964000

C -5.040759000 -1.483185000 -3.027206000

H -4.184879000 -0.957719000 -3.469584000

H -4.650988000 -2.412919000 -2.594183000

H -5.767436000 -1.720562000 -3.807887000

C -6.449481000 1.258297000 0.594937000

H -6.290988000 2.338479000 0.705519000

H -7.213684000 1.081336000 -0.162931000

H -6.811019000 0.882113000 1.560300000

**TS_8B-PD_**

E = -2363.18451049 a.u.

C 2.668388000 4.593476000 0.209285000

C 2.020519000 4.469018000 1.435903000

C 0.763170000 3.879113000 1.509121000

C 0.110323000 3.404304000 0.363669000

C 0.771712000 3.547070000 -0.861360000

C 2.034700000 4.132590000 -0.938873000

H 3.652698000 5.053063000 0.149006000

H 2.498029000 4.828124000 2.345559000

H 0.303818000 3.200900000 -1.780325000

H 2.518027000 4.232794000 -1.909142000

C -1.244537000 2.691438000 0.481520000

C -1.951274000 2.620094000 -0.870827000

H -1.460180000 1.927571000 -1.566362000

H -2.990056000 2.295613000 -0.759046000

H -1.970232000 3.616496000 -1.329856000

C -2.159669000 3.466972000 1.446225000

H -3.141179000 2.984292000 1.508424000

H -1.756554000 3.524518000 2.463010000

H -2.301832000 4.492026000 1.080810000

C -0.975828000 1.322387000 1.137926000

H 0.297983000 1.056767000 -0.013972000

H -0.271767000 1.441452000 1.967457000

C 2.981356000 0.321615000 -1.760450000

C 3.269996000 1.249783000 -2.767783000

C 4.452901000 1.983363000 -2.753804000

C 5.368534000 1.800304000 -1.724782000

C 5.094684000 0.877914000 -0.720733000

C 3.913012000 0.132959000 -0.717858000

H 2.567620000 1.416373000 -3.581099000

H 4.651418000 2.699102000 -3.548597000

H 6.295198000 2.369391000 -1.703780000

H 5.809170000 0.721943000 0.087359000

C 3.685950000 -0.839834000 0.387502000

C 4.064856000 -2.184064000 0.242937000

C 3.142006000 -0.389482000 1.606935000

C 3.779202000 -3.084191000 1.274937000

C 2.899900000 -1.311947000 2.621521000

C 3.182177000 -2.670708000 2.462903000

H 4.049094000 -4.133917000 1.148022000

H 2.468906000 -0.964894000 3.562692000

P 1.382111000 -0.607507000 -1.799284000

Pd -0.191683000 -0.422805000 -0.045341000

C 0.516149000 0.018466000 -3.291110000

C 1.803130000 -2.310774000 -2.355498000

C 2.856445000 -3.638134000 3.558995000

H 3.395204000 -3.393461000 4.483349000

H 1.785705000 -3.610332000 3.802070000

H 3.113576000 -4.666092000 3.281964000

C 4.834948000 -2.659048000 -0.954364000

H 5.910054000 -2.483555000 -0.807067000

H 4.701443000 -3.735238000 -1.112266000

H 4.558178000 -2.138310000 -1.876376000

C 2.849829000 1.063844000 1.826178000

H 2.230660000 1.486269000 1.023309000

H 2.328418000 1.221648000 2.777996000

H 3.772556000 1.659939000 1.853308000

H -0.428144000 -0.536999000 -3.335046000

H 0.298548000 1.088106000 -3.206885000

H 1.092811000 -0.172962000 -4.203835000

H 0.876614000 -2.749763000 -2.741019000

H 2.169529000 -2.924575000 -1.528166000

H 2.550666000 -2.270468000 -3.155732000

C -1.088051000 -3.082574000 -1.051385000

C -1.207631000 -4.588037000 -0.957595000

H -1.549617000 -4.897995000 0.035324000

H -1.881608000 -4.980017000 -1.723939000

H -0.214102000 -5.029539000 -1.111933000

O -1.411750000 -2.498351000 -2.099331000

H 0.293281000 3.777635000 2.486320000

O -0.600176000 -2.523966000 -0.005610000

C -1.838610000 0.206051000 1.288732000

C -3.088031000 0.124254000 0.495709000

C -1.865809000 -0.549641000 2.644829000

C -2.511142000 0.363087000 3.697019000

H -3.520951000 0.673804000 3.404869000

H -2.587119000 -0.166581000 4.656772000

H -1.910184000 1.267960000 3.860548000

C -2.689308000 -1.833407000 2.531040000

H -2.288268000 -2.490629000 1.749506000

H -2.656366000 -2.372494000 3.486983000

H -3.745161000 -1.631339000 2.308877000

C -0.467549000 -0.927683000 3.132584000

H 0.028609000 -1.614429000 2.434135000

H 0.180806000 -0.054087000 3.276967000

H -0.544663000 -1.431308000 4.106489000

C -3.459917000 -0.497620000 -0.649593000

H -2.811731000 -1.105663000 -1.277005000

C -4.848656000 -0.185816000 -0.863700000

C -5.694301000 -0.609092000 -1.986948000

O -6.877562000 -0.302472000 -2.071288000

C -5.233130000 0.606604000 0.191135000

O -4.181803000 0.793809000 1.018522000

C -5.028435000 -1.444455000 -3.043631000

H -4.165987000 -0.915301000 -3.468231000

H -4.645187000 -2.377658000 -2.612211000

H -5.743583000 -1.675959000 -3.836645000

C -6.494886000 1.263798000 0.579307000

H -6.349110000 2.344443000 0.700434000

H -7.249115000 1.084928000 -0.187824000

H -6.862578000 0.875930000 1.537708000

**IM9**

E = -2363.18620363 a.u.

C 2.624865000 4.582813000 0.387373000

C 1.978490000 4.403149000 1.607770000

C 0.720538000 3.810902000 1.655561000

C 0.065701000 3.388423000 0.491240000

C 0.724717000 3.589823000 -0.727264000

C 1.988479000 4.176374000 -0.779903000

H 3.609334000 5.044196000 0.347382000

H 2.457566000 4.720216000 2.532158000

H 0.257673000 3.282166000 -1.660441000

H 2.471810000 4.316855000 -1.745299000

C -1.292841000 2.679455000 0.579425000

C -1.990972000 2.647743000 -0.779526000

H -1.491256000 1.985475000 -1.497217000

H -3.028586000 2.314419000 -0.684781000

H -2.015251000 3.660232000 -1.202049000

C -2.213384000 3.453990000 1.545324000

H -3.197073000 2.972794000 1.593665000

H -1.816838000 3.500537000 2.565391000

H -2.348280000 4.483408000 1.189134000

C -1.064985000 1.309945000 1.236294000

H 0.449532000 1.091254000 -0.295784000

H -0.338129000 1.391638000 2.049513000

C 2.980713000 0.392834000 -1.751428000

C 3.268301000 1.364626000 -2.716453000

C 4.448486000 2.101091000 -2.663395000

C 5.359042000 1.876585000 -1.638266000

C 5.085550000 0.908814000 -0.677104000

C 3.907216000 0.160278000 -0.713101000

H 2.568948000 1.564504000 -3.524829000

H 4.648002000 2.852286000 -3.424412000

H 6.282653000 2.448720000 -1.587181000

H 5.795862000 0.721505000 0.127983000

C 3.667384000 -0.859802000 0.346430000

C 4.051935000 -2.195978000 0.149960000

C 3.085845000 -0.467900000 1.567866000

C 3.731456000 -3.144792000 1.126307000

C 2.802792000 -1.439674000 2.524778000

C 3.086218000 -2.789619000 2.308451000

H 4.005201000 -4.187557000 0.957396000

H 2.338446000 -1.137137000 3.465288000

P 1.378148000 -0.521902000 -1.823413000

Pd -0.128774000 -0.319296000 -0.053176000

C 0.513433000 0.138722000 -3.297687000

C 1.781340000 -2.223225000 -2.386820000

C 2.711850000 -3.811929000 3.337303000

H 3.194352000 -3.607626000 4.301631000

H 1.628633000 -3.806401000 3.520354000

H 2.996015000 -4.822802000 3.026103000

C 4.851243000 -2.610247000 -1.050726000

H 5.912546000 -2.363108000 -0.907498000

H 4.787822000 -3.691623000 -1.215645000

H 4.536165000 -2.104709000 -1.969525000

C 2.798455000 0.974874000 1.852828000

H 2.203401000 1.440630000 1.055919000

H 2.252965000 1.088207000 2.797461000

H 3.725310000 1.559964000 1.933371000

H -0.437467000 -0.403537000 -3.363391000

H 0.304060000 1.207429000 -3.185698000

H 1.091194000 -0.034995000 -4.213007000

H 0.837423000 -2.678537000 -2.704792000

H 2.211598000 -2.818858000 -1.577535000

H 2.480850000 -2.173306000 -3.229098000

C -0.951938000 -3.080092000 -0.878454000

C -0.992860000 -4.584375000 -0.714687000

H -1.245323000 -4.869484000 0.311783000

H -1.699259000 -5.038408000 -1.414895000

H 0.006723000 -4.985345000 -0.929742000

O -1.312265000 -2.568371000 -1.953260000

H 0.250585000 3.668449000 2.627793000

O -0.488517000 -2.444975000 0.132182000

C -1.900862000 0.198279000 1.322863000

C -3.107133000 0.101619000 0.468652000

C -1.943972000 -0.632080000 2.632408000

C -2.659822000 0.219118000 3.692436000

H -3.664770000 0.515326000 3.367999000

H -2.763570000 -0.353837000 4.624326000

H -2.093352000 1.131980000 3.920155000

C -2.731624000 -1.929134000 2.441484000

H -2.285701000 -2.548206000 1.655004000

H -2.723568000 -2.500353000 3.379100000

H -3.782791000 -1.740697000 2.186388000

C -0.553327000 -0.983187000 3.157661000

H 0.001131000 -1.606130000 2.445119000

H 0.049027000 -0.093731000 3.381537000

H -0.649632000 -1.544393000 4.097678000

C -3.407816000 -0.525094000 -0.693794000

H -2.720945000 -1.132369000 -1.279496000

C -4.785872000 -0.227330000 -0.982438000

C -5.562716000 -0.651718000 -2.154245000

O -6.743410000 -0.359001000 -2.301212000

C -5.236151000 0.558919000 0.050749000

O -4.232920000 0.757105000 0.935042000

C -4.827350000 -1.469296000 -3.178132000

H -3.940926000 -0.932118000 -3.538679000

H -4.468939000 -2.406186000 -2.733455000

H -5.488016000 -1.694849000 -4.018638000

C -6.527693000 1.197593000 0.365404000

H -6.410895000 2.282890000 0.475787000

H -7.236584000 0.990049000 -0.437775000

H -6.935791000 0.816259000 1.309849000

**TS_9-10_**

E = -1358.21915701 a.u.

H 1.751021000 -2.061744000 0.162084000

C -2.473644000 -0.602625000 -0.029875000

C -3.789632000 -1.069214000 -0.117866000

C -4.845048000 -0.189823000 -0.324612000

C -4.589419000 1.173454000 -0.448296000

C -3.286203000 1.645079000 -0.355742000

C -2.211165000 0.772387000 -0.143221000

H -3.997059000 -2.134654000 -0.022786000

H -5.862918000 -0.567178000 -0.391462000

H -5.406768000 1.871584000 -0.614887000

H -3.084979000 2.712388000 -0.446323000

C -0.851162000 1.383467000 -0.029175000

C -0.090738000 1.645247000 -1.188584000

C -0.419422000 1.883582000 1.220325000

C 1.080092000 2.404897000 -1.076435000

C 0.754396000 2.631156000 1.284243000

C 1.514746000 2.909818000 0.145010000

H 1.661325000 2.606105000 -1.977684000

H 1.081559000 3.016502000 2.251427000

P -1.075985000 -1.777644000 0.210424000

Pd 0.913710000 -0.726340000 -0.055988000

C -1.440015000 -2.561300000 1.831633000

C -1.438082000 -3.142657000 -0.961847000

C 2.789353000 3.689398000 0.245883000

H 3.033337000 4.186909000 -0.699420000

H 2.736578000 4.451184000 1.032117000

H 3.632941000 3.030290000 0.492203000

C -0.560376000 1.193704000 -2.540953000

H 0.261659000 1.212498000 -3.264872000

H -0.972913000 0.177706000 -2.519638000

H -1.353867000 1.849685000 -2.925419000

C -1.217038000 1.617482000 2.460765000

H -1.351656000 0.539299000 2.626331000

H -0.722933000 2.035976000 3.343565000

H -2.223432000 2.051948000 2.398747000

H -0.714951000 -3.364138000 2.004976000

H -1.339162000 -1.819115000 2.630585000

H -2.453459000 -2.979473000 1.845131000

H -0.650902000 -3.897946000 -0.860706000

H -1.420743000 -2.758120000 -1.987267000

H -2.407766000 -3.611643000 -0.760416000

C 3.716785000 -1.276978000 -0.068505000

C 5.207510000 -1.113235000 -0.181297000

H 5.537327000 -0.262550000 0.423320000

H 5.738124000 -2.016184000 0.127627000

H 5.463457000 -0.886607000 -1.222421000

O 3.248656000 -2.420334000 0.183438000

O 3.010054000 -0.238344000 -0.252740000

**IM10**

E = -1358.22879118 a.u.

H -0.582902000 -2.626035000 0.703480000

C 2.546270000 -0.023235000 -0.171887000

C 3.923739000 -0.007037000 -0.408261000

C 4.562130000 1.177521000 -0.751160000

C 3.820105000 2.352101000 -0.856254000

C 2.450808000 2.337128000 -0.620827000

C 1.789960000 1.150565000 -0.277088000

H 4.500468000 -0.927869000 -0.325763000

H 5.633578000 1.186120000 -0.936551000

H 4.311374000 3.285176000 -1.123286000

H 1.872990000 3.257623000 -0.702504000

C 0.311236000 1.233177000 -0.035285000

C -0.154178000 1.668955000 1.238485000

C -0.575436000 1.256143000 -1.141324000

C -1.467503000 2.100307000 1.371079000

C -1.887403000 1.702358000 -0.955689000

C -2.347253000 2.131570000 0.283240000

H -1.816924000 2.433091000 2.349663000

H -2.562836000 1.710527000 -1.812456000

P 1.681475000 -1.569350000 0.281083000

Pd -0.515000000 -1.116489000 0.350652000

C 2.212854000 -2.808360000 -0.953424000

C 2.480510000 -2.142690000 1.823035000

C -3.761129000 2.582338000 0.471830000

H -3.815170000 3.496178000 1.075695000

H -4.256699000 2.773318000 -0.486357000

H -4.343511000 1.816247000 1.001799000

C 0.778538000 1.720434000 2.409480000

H 0.232097000 1.930463000 3.334618000

H 1.322379000 0.776112000 2.540868000

H 1.536565000 2.505272000 2.282723000

C -0.097843000 0.899237000 -2.516866000

H 0.514759000 -0.010700000 -2.520396000

H -0.944455000 0.740121000 -3.192562000

H 0.522294000 1.701764000 -2.939408000

H 1.767349000 -3.773935000 -0.691910000

H 1.862571000 -2.512514000 -1.947481000

H 3.304756000 -2.902997000 -0.958774000

H 2.054410000 -3.113717000 2.097471000

H 2.287212000 -1.429303000 2.630706000

H 3.561397000 -2.248352000 1.675523000

C -3.244977000 -1.251082000 -0.538476000

C -4.733166000 -0.966113000 -0.480013000

H -4.911266000 0.052730000 -0.849351000

H -5.283575000 -1.652595000 -1.130399000

H -5.124057000 -1.025168000 0.540204000

O -2.747168000 -1.701238000 -1.576504000

O -2.605148000 -0.951600000 0.538334000

**3a**

E = -1004.92768663 a.u.

C -5.645370000 -1.150564000 -0.896734000

C -5.398009000 -0.368199000 0.228299000

C -4.104027000 -0.236287000 0.719924000

C -3.020186000 -0.882228000 0.109594000

C -3.286511000 -1.656111000 -1.023863000

C -4.581605000 -1.790667000 -1.521200000

H -6.656888000 -1.256743000 -1.282873000

H -6.217550000 0.143123000 0.729767000

H -2.478625000 -2.169919000 -1.538677000

H -4.754856000 -2.402370000 -2.404481000

C -1.596210000 -0.696604000 0.659046000

C -0.666163000 -1.781773000 0.114100000

H -0.471067000 -1.675531000 -0.958743000

H 0.297081000 -1.780411000 0.632905000

H -1.117050000 -2.768045000 0.283161000

C -1.606955000 -0.828642000 2.193403000

H -0.581649000 -0.752519000 2.575114000

H -2.197670000 -0.044881000 2.680079000

H -2.018655000 -1.801762000 2.492757000

C -1.188049000 0.730581000 0.322536000

H -2.033580000 1.417331000 0.379188000

H -3.940535000 0.388333000 1.596595000

C 0.001799000 1.274072000 0.021100000

C 1.244541000 0.482218000 0.022960000

C 0.184912000 2.770141000 -0.269956000

C 1.242128000 3.338450000 0.683127000

H 2.217837000 2.853505000 0.550680000

H 1.379060000 4.411991000 0.497496000

H 0.940784000 3.211328000 1.731448000

C 0.661198000 2.957352000 -1.714599000

H -0.055848000 2.526029000 -2.425778000

H 0.760091000 4.027471000 -1.940765000

H 1.639105000 2.494255000 -1.890341000

C -1.102353000 3.567472000 -0.089789000

H -1.888513000 3.245046000 -0.784570000

H -1.494218000 3.493888000 0.932884000

H -0.902587000 4.627401000 -0.291560000

C 2.106503000 0.038323000 -0.924781000

H 1.998734000 0.176754000 -1.993915000

C 3.146205000 -0.683985000 -0.242111000

C 4.303051000 -1.364449000 -0.838393000

O 5.141197000 -1.948196000 -0.162082000

C 2.842904000 -0.611865000 1.097142000

O 1.703872000 0.094079000 1.264135000

C 4.407905000 -1.309112000 -2.335896000

H 3.520286000 -1.756406000 -2.800330000

H 4.455104000 -0.268474000 -2.679828000

H 5.301695000 -1.843762000 -2.666109000

C 3.485846000 -1.118435000 2.323923000

H 2.788729000 -1.737795000 2.902013000

H 4.361372000 -1.712525000 2.058275000

H 3.799536000 -0.290511000 2.972196000

**TS_10-11_**

E = -1358.21916130 a.u.

H 1.746632000 -2.065623000 0.140901000

C -2.474700000 -0.601515000 -0.024195000

C -3.792145000 -1.065781000 -0.102992000

C -4.848260000 -0.183545000 -0.293313000

C -4.592036000 1.180281000 -0.409529000

C -3.287354000 1.649479000 -0.326415000

C -2.211448000 0.773813000 -0.130840000

H -4.000355000 -2.131538000 -0.013264000

H -5.867262000 -0.559044000 -0.352929000

H -5.409969000 1.880741000 -0.562935000

H -3.085564000 2.717164000 -0.411033000

C -0.849755000 1.382824000 -0.026366000

C -0.097955000 1.646003000 -1.191085000

C -0.408580000 1.880898000 1.220670000

C 1.074046000 2.405029000 -1.086421000

C 0.765004000 2.629411000 1.276750000

C 1.516832000 2.909578000 0.132273000

H 1.648372000 2.607821000 -1.991754000

H 1.099116000 3.013992000 2.241876000

P -1.076247000 -1.779056000 0.198140000

Pd 0.912988000 -0.727852000 -0.071466000

C -1.428193000 -2.568484000 1.819198000

C -1.449135000 -3.139105000 -0.976336000

C 2.790613000 3.691559000 0.224506000

H 3.028643000 4.186945000 -0.723420000

H 2.740245000 4.455457000 1.008855000

H 3.636683000 3.034884000 0.468504000

C -0.578940000 1.198112000 -2.540662000

H 0.235951000 1.222564000 -3.272424000

H -0.988085000 0.180721000 -2.519589000

H -1.378353000 1.852992000 -2.914617000

C -1.195661000 1.610595000 2.466946000

H -1.323823000 0.531548000 2.632387000

H -0.696659000 2.030494000 3.346302000

H -2.204520000 2.040280000 2.412883000

H -0.701240000 -3.371097000 1.985501000

H -1.322680000 -1.828312000 2.619484000

H -2.441168000 -2.987600000 1.838489000

H -0.660982000 -3.894701000 -0.885590000

H -1.441846000 -2.750808000 -2.000421000

H -2.416704000 -3.608910000 -0.767016000

C 3.716139000 -1.280157000 -0.066838000

C 5.209321000 -1.107148000 -0.115893000

H 5.522972000 -0.468852000 0.717713000

H 5.728751000 -2.065208000 -0.047776000

H 5.497059000 -0.594761000 -1.039366000

O 3.245513000 -2.425838000 0.168801000

O 3.011271000 -0.240808000 -0.255321000

**IM11**

E = -1358.24006035 a.u.

H 2.822347000 -2.273957000 0.111119000

C -2.406107000 -0.823048000 -0.025347000

C -3.629824000 -1.505253000 -0.054143000

C -4.838157000 -0.827245000 -0.143707000

C -4.840009000 0.562873000 -0.208967000

C -3.635553000 1.252105000 -0.176172000

C -2.406799000 0.582449000 -0.081110000

H -3.644690000 -2.593325000 -0.002318000

H -5.773346000 -1.382535000 -0.163249000

H -5.777751000 1.109427000 -0.282491000

H -3.631027000 2.341066000 -0.221771000

C -1.184506000 1.438240000 -0.029439000

C -0.550138000 1.838124000 -1.220568000

C -0.745872000 1.946684000 1.204475000

C 0.517860000 2.732780000 -1.153673000

C 0.330028000 2.837697000 1.230265000

C 0.974247000 3.244447000 0.063069000

H 1.003251000 3.045950000 -2.080033000

H 0.664584000 3.233742000 2.190526000

P -0.844817000 -1.819579000 0.100438000

Pd 1.144797000 -0.772008000 -0.029886000

C -1.155407000 -2.796073000 1.635958000

C -1.109887000 -3.115058000 -1.188376000

C 2.134465000 4.192167000 0.104062000

H 2.025532000 4.993864000 -0.636557000

H 2.243664000 4.652797000 1.091967000

H 3.076702000 3.676875000 -0.125375000

C -1.017310000 1.317504000 -2.546947000

H -0.397884000 1.704652000 -3.362918000

H -0.974355000 0.219938000 -2.583390000

H -2.058740000 1.598288000 -2.752310000

C -1.422984000 1.550789000 2.482767000

H -1.414237000 0.461419000 2.626850000

H -0.929629000 2.007846000 3.347051000

H -2.477227000 1.857076000 2.496042000

H -0.339735000 -3.517748000 1.757217000

H -1.157950000 -2.128505000 2.504065000

H -2.108259000 -3.336938000 1.589241000

H -0.292339000 -3.841148000 -1.114200000

H -1.072961000 -2.657195000 -2.182592000

H -2.062874000 -3.642626000 -1.063742000

C 4.107492000 -0.928221000 -0.018214000

C 5.561543000 -0.631820000 -0.045002000

H 5.731072000 0.445001000 -0.013630000

H 6.062754000 -1.119903000 0.796039000

H 5.994803000 -1.041261000 -0.964507000

O 3.817369000 -2.209376000 0.118100000

O 3.232803000 -0.068321000 -0.126406000

**IM5A′**

E = -2134.13628807 a.u.

C -4.198121000 -2.504855000 -2.125047000

C -3.105023000 -2.071465000 -1.369282000

C -2.968926000 -0.731698000 -0.983054000

C -3.975191000 0.175433000 -1.382758000

C -5.069673000 -0.259633000 -2.131897000

C -5.182559000 -1.598323000 -2.505929000

H -4.278476000 -3.552379000 -2.414019000

H -2.351326000 -2.806496000 -1.078483000

H -5.843757000 0.448901000 -2.431022000

H -6.036318000 -1.929934000 -3.094745000

C -3.800400000 1.596081000 -0.902257000

C -4.591149000 1.786384000 0.397142000

H -4.247879000 1.092039000 1.176491000

H -4.470720000 2.810665000 0.779624000

H -5.663928000 1.610227000 0.236704000

C -4.274912000 2.638816000 -1.915506000

H -4.023020000 3.649017000 -1.563798000

H -3.794980000 2.493481000 -2.892723000

H -5.363017000 2.609981000 -2.066039000

C -2.302154000 1.783159000 -0.628868000

H -1.759084000 1.960925000 -1.572962000

H -2.131918000 2.658963000 0.015290000

C 0.708857000 2.975542000 0.632104000

C -0.086333000 4.111322000 0.850791000

C 0.172146000 5.317866000 0.210451000

C 1.240919000 5.414335000 -0.672558000

C 2.046594000 4.303214000 -0.888079000

C 1.800589000 3.079821000 -0.253602000

H -0.928418000 4.063388000 1.537484000

H -0.462489000 6.178990000 0.407380000

H 1.453639000 6.350568000 -1.183866000

H 2.900166000 4.371151000 -1.562445000

C 2.763913000 1.972390000 -0.512040000

C 2.511375000 1.005181000 -1.498565000

C 3.969465000 1.938843000 0.213864000

C 3.453579000 -0.003149000 -1.712530000

C 4.873246000 0.902249000 -0.014930000

C 4.628059000 -0.087126000 -0.966778000

H 3.272377000 -0.735535000 -2.501702000

H 5.796524000 0.868250000 0.565659000

P 0.153070000 1.389810000 1.410588000

Pd -1.427195000 0.071805000 0.131769000

C 1.643472000 0.539579000 2.077316000

C -0.621997000 1.971082000 2.979923000

C 5.580534000 -1.224174000 -1.163320000

H 5.601933000 -1.557501000 -2.207552000

H 6.600035000 -0.955254000 -0.864421000

H 5.272710000 -2.089338000 -0.560555000

C 1.247923000 1.023207000 -2.306155000

H 0.979190000 2.036937000 -2.628984000

H 1.342175000 0.395439000 -3.199683000

H 0.394192000 0.639252000 -1.719534000

C 4.286791000 2.991575000 1.234569000

H 3.454335000 3.151992000 1.932110000

H 5.171763000 2.718095000 1.819278000

H 4.488174000 3.964252000 0.765928000

H 1.281736000 -0.219743000 2.781376000

H 2.204723000 0.029486000 1.289139000

H 2.295158000 1.235777000 2.617624000

H -0.754448000 1.095450000 3.625608000

H -1.610213000 2.408260000 2.800036000

H 0.016433000 2.699225000 3.493958000

C -0.991746000 -1.902477000 1.368409000

C -0.286517000 -1.994140000 0.361584000

C -1.704788000 -2.212580000 2.628249000

C -2.228971000 -3.649322000 2.496020000

H -1.408106000 -4.361434000 2.347417000

H -2.763037000 -3.927319000 3.413790000

H -2.924647000 -3.741126000 1.652677000

C -0.703721000 -2.137851000 3.786136000

H -0.347739000 -1.111922000 3.942776000

H -1.190119000 -2.472255000 4.711284000

H 0.166004000 -2.781501000 3.600530000

C -2.876917000 -1.267576000 2.886640000

H -2.545890000 -0.222857000 2.936549000

H -3.637943000 -1.350822000 2.100909000

H -3.344242000 -1.527393000 3.845294000

C 0.529420000 -2.298643000 -0.758698000

H 0.125443000 -2.054770000 -1.743324000

C 1.727875000 -2.907529000 -0.643660000

C 2.538732000 -3.344868000 -1.802873000

O 3.603052000 -3.914361000 -1.596500000

C 2.311600000 -3.225562000 0.710817000

O 2.997591000 -2.411792000 1.297136000

C 2.013143000 -3.135319000 -3.191583000

H 1.193749000 -3.840068000 -3.382876000

H 1.614776000 -2.126377000 -3.346220000

H 2.815201000 -3.326666000 -3.908736000

C 1.977023000 -4.574791000 1.258694000

H 0.887031000 -4.707121000 1.282186000

H 2.369152000 -5.348161000 0.585677000

H 2.397923000 -4.705692000 2.259181000

**TS_5-6A′_**

E = -2134.11670943 a.u.

C 5.707496000 -0.921160000 -0.335452000

C 4.404161000 -0.482620000 -0.082962000

C 3.288545000 -1.259460000 -0.424539000

C 3.521578000 -2.510435000 -1.040102000

C 4.823454000 -2.950255000 -1.285453000

C 5.917412000 -2.158079000 -0.936368000

H 6.556605000 -0.295974000 -0.060391000

H 4.265975000 0.492611000 0.390280000

H 4.992398000 -3.919889000 -1.756900000

H 6.929381000 -2.506805000 -1.136590000

C 2.287660000 -3.325431000 -1.356615000

C 1.999850000 -4.264192000 -0.178987000

H 1.866482000 -3.702363000 0.755896000

H 1.083689000 -4.846683000 -0.358572000

H 2.825330000 -4.974262000 -0.028596000

C 2.435531000 -4.173525000 -2.621177000

H 1.478912000 -4.657359000 -2.863840000

H 2.728473000 -3.558529000 -3.483045000

H 3.182465000 -4.972226000 -2.508855000

C 1.137353000 -2.321801000 -1.525340000

H 1.203484000 -1.852525000 -2.522793000

H 0.164263000 -2.838335000 -1.475740000

C -2.122288000 -1.557391000 0.567502000

C -1.401819000 -2.598379000 1.168590000

C -2.032799000 -3.626228000 1.861625000

C -3.417486000 -3.624011000 1.970499000

C -4.149104000 -2.596710000 1.383823000

C -3.529627000 -1.559261000 0.679290000

H -0.314890000 -2.597368000 1.080219000

H -1.441326000 -4.420303000 2.312391000

H -3.930816000 -4.417915000 2.508573000

H -5.236040000 -2.587923000 1.465553000

C -4.408494000 -0.512844000 0.085521000

C -4.917094000 -0.676287000 -1.212632000

C -4.775975000 0.610767000 0.849303000

C -5.720080000 0.328749000 -1.758946000

C -5.584715000 1.587718000 0.271522000

C -6.055319000 1.472625000 -1.037995000

H -6.098373000 0.207840000 -2.775197000

H -5.854434000 2.466496000 0.860117000

P -1.059315000 -0.319664000 -0.320956000

Pd 1.303519000 -0.743588000 -0.169887000

C -1.581922000 -0.327391000 -2.086527000

C -1.609498000 1.371115000 0.159493000

C -6.900098000 2.553827000 -1.640029000

H -7.268786000 2.271018000 -2.631721000

H -7.767456000 2.782813000 -1.008375000

H -6.334187000 3.488238000 -1.747156000

C -4.648499000 -1.922130000 -2.004329000

H -5.293363000 -2.746250000 -1.668711000

H -4.850831000 -1.761938000 -3.069147000

H -3.615113000 -2.272235000 -1.902265000

C -4.324154000 0.764249000 2.271200000

H -3.271206000 0.489304000 2.406741000

H -4.455095000 1.795649000 2.615994000

H -4.904781000 0.116925000 2.942999000

C 1.669697000 0.767132000 1.329942000

C 1.762203000 1.866832000 0.647032000

C 1.719065000 0.469488000 2.801347000

C 1.828690000 -1.026994000 3.089075000

H 2.752184000 -1.448799000 2.671833000

H 1.844397000 -1.191079000 4.174956000

H 0.976678000 -1.581001000 2.675219000

C 2.924351000 1.179048000 3.431024000

H 2.859050000 2.265183000 3.318981000

H 2.968813000 0.936828000 4.501039000

H 3.861377000 0.838276000 2.970885000

C 0.416621000 0.997264000 3.420298000

H 0.303491000 2.075540000 3.261919000

H -0.458587000 0.486007000 2.995485000

H 0.426758000 0.802645000 4.500819000

C 1.780048000 2.507449000 -0.595772000

H 1.714430000 1.882343000 -1.485884000

C 1.875013000 3.874537000 -0.633219000

C 1.885633000 4.687755000 -1.860931000

O 1.965578000 5.907306000 -1.808693000

C 1.938560000 4.397730000 0.719347000

O 1.919586000 3.531202000 1.622690000

C 1.788354000 3.964314000 -3.173421000

H 2.625285000 3.267072000 -3.300252000

H 0.863954000 3.375930000 -3.228366000

H 1.800800000 4.693612000 -3.986492000

C 2.011112000 5.836539000 1.068765000

H 2.887874000 6.295682000 0.600542000

H 1.141085000 6.365539000 0.665230000

H 2.054744000 5.953171000 2.153908000

H -0.916074000 0.370806000 -2.610824000

H -1.452144000 -1.321276000 -2.525968000

H -2.617531000 0.007476000 -2.213557000

H -0.954910000 2.077452000 -0.366553000

H -2.643644000 1.569550000 -0.138996000

H -1.492749000 1.533653000 1.236000000

**IM6A′**

E = -2134.14020520 a.u.

C 5.785975000 -0.674846000 -0.165669000

C 4.455737000 -0.305517000 0.054251000

C 3.387914000 -1.132565000 -0.324395000

C 3.701724000 -2.364497000 -0.944352000

C 5.031043000 -2.734461000 -1.156297000

C 6.074977000 -1.893460000 -0.770178000

H 6.593274000 -0.008990000 0.138152000

H 4.255279000 0.658303000 0.527585000

H 5.260145000 -3.688666000 -1.633747000

H 7.108165000 -2.189053000 -0.945984000

C 2.522875000 -3.240099000 -1.309071000

C 2.234106000 -4.184541000 -0.135864000

H 2.033469000 -3.622692000 0.787177000

H 1.355918000 -4.813213000 -0.347197000

H 3.086581000 -4.851854000 0.055823000

C 2.770707000 -4.089386000 -2.556565000

H 1.849396000 -4.619382000 -2.837099000

H 3.070375000 -3.466591000 -3.410749000

H 3.548881000 -4.852058000 -2.408114000

C 1.334771000 -2.291052000 -1.527599000

H 1.429316000 -1.824557000 -2.525785000

H 0.391841000 -2.865640000 -1.539788000

C -2.073779000 -1.686824000 0.493247000

C -1.327082000 -2.722987000 1.065854000

C -1.939114000 -3.775102000 1.739141000

C -3.323660000 -3.797559000 1.854902000

C -4.075953000 -2.770032000 1.296213000

C -3.476869000 -1.707160000 0.610701000

H -0.240330000 -2.696108000 0.976322000

H -1.332913000 -4.568606000 2.170885000

H -3.820692000 -4.610992000 2.378921000

H -5.162284000 -2.780383000 1.385294000

C -4.368698000 -0.655051000 0.049654000

C -4.875040000 -0.781759000 -1.252694000

C -4.746537000 0.439163000 0.850608000

C -5.682992000 0.236179000 -1.767975000

C -5.563474000 1.426868000 0.305114000

C -6.028671000 1.352351000 -1.009832000

H -6.060193000 0.145092000 -2.787783000

H -5.843360000 2.282728000 0.921958000

P -1.029808000 -0.398425000 -0.403585000

Pd 1.377749000 -0.710906000 -0.129069000

C -1.483649000 -0.508650000 -2.215244000

C -1.699024000 1.310516000 -0.044250000

C -6.881609000 2.446042000 -1.576915000

H -7.250609000 2.191210000 -2.576038000

H -7.748640000 2.649850000 -0.936339000

H -6.321713000 3.386761000 -1.657193000

C -4.608016000 -2.005649000 -2.078746000

H -5.261095000 -2.833786000 -1.769703000

H -4.803527000 -1.814562000 -3.139748000

H -3.578297000 -2.367074000 -1.981199000

C -4.290399000 0.546316000 2.274932000

H -3.206554000 0.406822000 2.376579000

H -4.550169000 1.522348000 2.697976000

H -4.759480000 -0.223907000 2.902049000

C 1.560411000 0.817237000 1.233674000

C 1.625270000 2.110618000 0.705721000

C 1.523634000 0.598944000 2.730370000

C 1.633138000 -0.886971000 3.078819000

H 2.585562000 -1.308701000 2.732668000

H 1.582579000 -1.019788000 4.168321000

H 0.817833000 -1.468008000 2.628999000

C 2.679173000 1.326659000 3.435660000

H 2.605264000 2.414153000 3.356366000

H 2.672959000 1.062493000 4.501330000

H 3.647766000 1.013486000 3.023486000

C 0.172473000 1.108068000 3.264067000

H 0.033168000 2.181728000 3.099170000

H -0.661658000 0.570649000 2.791176000

H 0.120905000 0.918294000 4.344330000

C 1.721942000 2.523152000 -0.618540000

H 1.795757000 1.837247000 -1.457227000

C 1.697588000 3.924205000 -0.654703000

C 1.751147000 4.824638000 -1.818079000

O 1.719669000 6.037175000 -1.668991000

C 1.580652000 4.334958000 0.674254000

O 1.547945000 3.287902000 1.483177000

C 1.839208000 4.177456000 -3.166347000

H 2.751916000 3.572705000 -3.238235000

H 0.993099000 3.496079000 -3.321221000

H 1.844270000 4.940718000 -3.947548000

C 1.488954000 5.673686000 1.276108000

H 2.394182000 6.251535000 1.058053000

H 0.653715000 6.230317000 0.838644000

H 1.356589000 5.599576000 2.358137000

H -0.808514000 0.171229000 -2.749069000

H -1.330760000 -1.524827000 -2.588887000

H -2.519141000 -0.193451000 -2.381360000

H -1.030937000 2.016381000 -0.552394000

H -2.712458000 1.432409000 -0.437844000

H -1.678258000 1.524891000 1.028454000

**TS_6-7A′_**

E = -2134.10530373 a.u.

C 4.669273000 2.531686000 -0.432750000

C 3.382457000 2.063138000 -0.185107000

C 3.011852000 0.708566000 -0.269314000

C 4.001305000 -0.193108000 -0.823997000

C 5.294499000 0.307029000 -1.022181000

C 5.652210000 1.631638000 -0.799911000

H 4.889583000 3.589441000 -0.305180000

H 2.661951000 2.799890000 0.158128000

H 6.058793000 -0.362870000 -1.406300000

H 6.679606000 1.952263000 -0.959052000

C 3.715155000 -1.608700000 -1.390332000

C 3.771290000 -2.693774000 -0.304506000

H 2.970569000 -2.593119000 0.436642000

H 3.663822000 -3.682398000 -0.773501000

H 4.735865000 -2.676844000 0.222226000

C 4.746322000 -2.019922000 -2.455232000

H 4.395694000 -2.941605000 -2.936448000

H 4.861244000 -1.257019000 -3.235576000

H 5.737829000 -2.238055000 -2.035429000

C 2.333994000 -1.617463000 -2.072130000

H 2.323004000 -0.955327000 -2.953518000

H 2.089557000 -2.636523000 -2.400593000

C -1.523118000 -2.549949000 0.631211000

C -0.880955000 -3.487633000 1.455676000

C -1.456237000 -3.932114000 2.637492000

C -2.698787000 -3.436760000 3.023692000

C -3.338168000 -2.499109000 2.223992000

C -2.770490000 -2.033421000 1.030432000

H 0.098983000 -3.872095000 1.169405000

H -0.935912000 -4.662216000 3.253186000

H -3.164881000 -3.775376000 3.946207000

H -4.306094000 -2.097365000 2.523990000

C -3.511504000 -0.980883000 0.279473000

C -4.464728000 -1.325090000 -0.694853000

C -3.273438000 0.368163000 0.583327000

C -5.105312000 -0.309293000 -1.403935000

C -3.930819000 1.357963000 -0.151182000

C -4.838059000 1.040204000 -1.160083000

H -5.830136000 -0.578619000 -2.174356000

H -3.730627000 2.406696000 0.080582000

P -0.527960000 -2.087319000 -0.862021000

Pd 1.274753000 -0.748395000 -0.521348000

C -0.240417000 -3.742798000 -1.615054000

C -1.575525000 -1.300887000 -2.145972000

C -5.511877000 2.115389000 -1.957650000

H -5.303903000 2.006877000 -3.029895000

H -6.602719000 2.079437000 -1.842929000

H -5.173125000 3.111627000 -1.649898000

C -4.779550000 -2.762054000 -0.985618000

H -5.190145000 -3.270277000 -0.103152000

H -5.512442000 -2.850222000 -1.794417000

H -3.884832000 -3.328744000 -1.278392000

C -2.322753000 0.749717000 1.675162000

H -1.305572000 0.400988000 1.454609000

H -2.290485000 1.838223000 1.803033000

H -2.608171000 0.305482000 2.638035000

C 1.540838000 0.658468000 0.912167000

C 0.702751000 1.816116000 0.609383000

C 2.055358000 0.471644000 2.351985000

C 2.875420000 -0.808490000 2.490492000

H 3.770898000 -0.795385000 1.857175000

H 3.209589000 -0.918232000 3.531168000

H 2.281035000 -1.695531000 2.234779000

C 2.920435000 1.620379000 2.894528000

H 2.427043000 2.593536000 2.814930000

H 3.138647000 1.441118000 3.956287000

H 3.882369000 1.678804000 2.369382000

C 0.835271000 0.302746000 3.275636000

H 0.223408000 1.207287000 3.339319000

H 0.195283000 -0.527363000 2.944910000

H 1.185637000 0.068240000 4.290036000

C 0.010244000 2.176695000 -0.517455000

H -0.000151000 1.616210000 -1.447179000

C -0.707482000 3.378354000 -0.229806000

C -1.658543000 4.080711000 -1.102094000

O -2.260076000 5.086594000 -0.743837000

C -0.408112000 3.694230000 1.077525000

O 0.454998000 2.786004000 1.574438000

C -1.884851000 3.478082000 -2.459738000

H -0.939810000 3.371623000 -3.006223000

H -2.307233000 2.468573000 -2.353119000

H -2.573509000 4.101449000 -3.035675000

C -0.861980000 4.750916000 2.000628000

H -0.015537000 5.334999000 2.382674000

H -1.551454000 5.416174000 1.478186000

H -1.373236000 4.314145000 2.868983000

H 0.081120000 -3.597368000 -2.651706000

H 0.542494000 -4.290384000 -1.080841000

H -1.168183000 -4.326136000 -1.606378000

H -0.915211000 -1.165970000 -3.011977000

H -2.419965000 -1.935926000 -2.434175000

H -1.935112000 -0.319244000 -1.823859000

**IM7A′**

E = -2134.19667262 a.u.

C 5.660120000 0.660804000 1.056030000

C 4.459026000 1.053206000 0.483626000

C 3.510449000 0.131975000 0.004402000

C 3.762739000 -1.254273000 0.160856000

C 5.000530000 -1.619914000 0.714589000

C 5.942232000 -0.696892000 1.149635000

H 6.369750000 1.405895000 1.409802000

H 4.239927000 2.115848000 0.380931000

H 5.234046000 -2.676505000 0.832697000

H 6.882980000 -1.042151000 1.573962000

C 2.775139000 -2.408850000 -0.124233000

C 2.473961000 -3.066660000 1.233902000

H 2.059651000 -2.335353000 1.940488000

H 1.741363000 -3.877205000 1.109866000

H 3.370875000 -3.500865000 1.692363000

C 3.441691000 -3.466236000 -1.023376000

H 2.733185000 -4.282944000 -1.215960000

H 3.735017000 -3.041547000 -1.992117000

H 4.336612000 -3.911664000 -0.569819000

C 1.436623000 -2.032147000 -0.778348000

H 1.551329000 -1.884382000 -1.859924000

H 0.737826000 -2.873108000 -0.648397000

C -2.312170000 -2.370973000 0.567929000

C -2.130413000 -3.733759000 0.839193000

C -2.913169000 -4.706706000 0.225859000

C -3.902202000 -4.330957000 -0.675121000

C -4.091033000 -2.982906000 -0.957825000

C -3.304509000 -1.992360000 -0.361956000

H -1.367936000 -4.053707000 1.546036000

H -2.748639000 -5.756311000 0.459308000

H -4.523839000 -5.081808000 -1.157653000

H -4.860230000 -2.675818000 -1.666344000

C -3.547986000 -0.573035000 -0.746151000

C -2.768283000 0.014733000 -1.755714000

C -4.574753000 0.164618000 -0.128934000

C -2.960399000 1.364638000 -2.064657000

C -4.746993000 1.503993000 -0.475066000

C -3.939046000 2.127810000 -1.427909000

H -2.352371000 1.819580000 -2.849619000

H -5.537170000 2.079081000 0.010981000

P -1.162139000 -1.152122000 1.362775000

Pd 0.669225000 -0.304607000 0.120539000

C -2.239005000 0.134090000 2.136535000

C -0.642600000 -2.015296000 2.905882000

C -4.166330000 3.564645000 -1.787898000

H -3.430067000 3.920856000 -2.517247000

H -5.162863000 3.708789000 -2.225559000

H -4.114295000 4.215597000 -0.905978000

C -1.771563000 -0.800623000 -2.522938000

H -1.393327000 -0.241950000 -3.385526000

H -0.909597000 -1.079384000 -1.895286000

H -2.219060000 -1.732498000 -2.891775000

C -5.487987000 -0.465503000 0.880139000

H -6.020827000 0.297812000 1.457532000

H -6.245795000 -1.094677000 0.393221000

H -4.950383000 -1.113521000 1.582074000

C 2.339946000 0.697727000 -0.754665000

C 1.581732000 1.722509000 -0.062754000

C 2.619837000 0.912120000 -2.277062000

C 3.565588000 -0.148999000 -2.848252000

H 3.158367000 -1.161976000 -2.769751000

H 3.721131000 0.057699000 -3.915611000

H 4.550504000 -0.136886000 -2.364684000

C 1.343559000 0.876830000 -3.120063000

H 0.569855000 1.556037000 -2.747059000

H 1.576281000 1.168073000 -4.154022000

H 0.925940000 -0.135805000 -3.150406000

C 3.326521000 2.262878000 -2.483815000

H 2.700326000 3.114400000 -2.200778000

H 4.260331000 2.316395000 -1.909538000

H 3.588706000 2.381799000 -3.544047000

C 1.156120000 1.764305000 1.270687000

H 1.640289000 1.259725000 2.100880000

C 0.204703000 2.846803000 1.370361000

C -0.473014000 3.320536000 2.583110000

O -1.376904000 4.146758000 2.550114000

C 0.068796000 3.355972000 0.107399000

O 0.888539000 2.699962000 -0.752534000

C 0.026834000 2.759824000 3.885371000

H 0.087470000 1.664356000 3.862560000

H 1.045961000 3.124008000 4.072214000

H -0.623250000 3.079105000 4.703476000

C -0.739045000 4.439135000 -0.478329000

H -0.096243000 5.222616000 -0.899480000

H -1.360698000 4.054861000 -1.298500000

H -1.382278000 4.873054000 0.289079000

H -1.622551000 0.642847000 2.887431000

H -2.588492000 0.873285000 1.409974000

H -3.094910000 -0.322425000 2.646944000

H -0.061369000 -1.290384000 3.489406000

H -1.512735000 -2.333172000 3.492647000

H 0.001962000 -2.874061000 2.701510000

**IM8B-cis**

E = -2363.17028520 a.u.

C -5.699526000 4.038827000 0.796096000

C -5.541258000 2.970911000 -0.083178000

C -4.268632000 2.532976000 -0.433115000

C -3.121577000 3.133816000 0.099663000

C -3.296733000 4.224201000 0.957335000

C -4.569665000 4.669033000 1.306251000

H -6.694203000 4.378739000 1.076328000

H -6.414138000 2.473767000 -0.502356000

H -2.432951000 4.728220000 1.384422000

H -4.674215000 5.511468000 1.987103000

C -1.728417000 2.647303000 -0.299214000

C -0.620307000 3.227165000 0.574517000

H -0.813888000 3.117668000 1.645601000

H 0.335073000 2.735303000 0.340641000

H -0.491225000 4.297209000 0.370083000

C -1.469111000 3.146823000 -1.733204000

H -0.458470000 2.870642000 -2.070270000

H -2.194406000 2.735661000 -2.446420000

H -1.544699000 4.240274000 -1.766659000

C -1.695265000 1.089088000 -0.381754000

H -2.300596000 0.808400000 -1.252556000

H -0.611406000 0.992676000 -0.929030000

C 2.879638000 1.219395000 -1.616207000

C 2.344705000 2.178958000 -2.487902000

C 2.817917000 3.485675000 -2.509689000

C 3.849506000 3.859117000 -1.654494000

C 4.393164000 2.916655000 -0.790408000

C 3.921754000 1.599444000 -0.746430000

H 1.535917000 1.907553000 -3.164830000

H 2.381624000 4.206292000 -3.198098000

H 4.230513000 4.877778000 -1.660744000

H 5.205234000 3.196737000 -0.119566000

C 4.559893000 0.660449000 0.218956000

C 5.748064000 -0.001032000 -0.132203000

C 3.986145000 0.452413000 1.485862000

C 6.316196000 -0.900802000 0.772029000

C 4.580645000 -0.457960000 2.359307000

C 5.743635000 -1.151015000 2.017614000

H 7.230643000 -1.425719000 0.491431000

H 4.129529000 -0.619876000 3.340214000

P 2.010692000 -0.412755000 -1.547728000

Pd 0.050140000 -0.396665000 -0.157902000

C 1.488411000 -0.701870000 -3.288105000

C 3.256590000 -1.749884000 -1.352743000

C 6.364570000 -2.116743000 2.980359000

H 6.760585000 -1.598802000 3.863472000

H 5.630595000 -2.845619000 3.345815000

H 7.190850000 -2.668126000 2.519129000

C 6.399038000 0.226858000 -1.464197000

H 6.778223000 1.252733000 -1.561551000

H 7.244661000 -0.453832000 -1.608122000

H 5.697786000 0.074217000 -2.295378000

C 2.752305000 1.193914000 1.902728000

H 1.877401000 0.887297000 1.308340000

H 2.517055000 1.001089000 2.955285000

H 2.862064000 2.278767000 1.771570000

H 1.116346000 -1.731687000 -3.333811000

H 0.665239000 -0.038793000 -3.571432000

H 2.322022000 -0.584331000 -3.990234000

H 2.731957000 -2.680101000 -1.597488000

H 3.616622000 -1.814253000 -0.323242000

H 4.100611000 -1.611877000 -2.037814000

C 0.801075000 -3.151908000 0.378825000

C 1.475551000 -4.238075000 1.187420000

H 1.095897000 -4.258954000 2.213733000

H 1.343932000 -5.217923000 0.721041000

H 2.550236000 -4.019257000 1.243674000

O 0.460306000 -3.358691000 -0.794705000

H -4.177883000 1.694776000 -1.120741000

O 0.666514000 -2.038806000 1.011089000

C -1.857688000 0.002729000 0.642857000

C -2.782208000 -1.037921000 0.116972000

C -1.897794000 0.123612000 2.193819000

C -2.981739000 1.105111000 2.657811000

H -3.949999000 0.909201000 2.180141000

H -3.116723000 1.006620000 3.743913000

H -2.710399000 2.146060000 2.453438000

C -2.223136000 -1.250184000 2.798699000

H -1.506587000 -2.010526000 2.463709000

H -2.156735000 -1.180509000 3.892638000

H -3.237414000 -1.592312000 2.555570000

C -0.555915000 0.540762000 2.798377000

H 0.188410000 -0.253163000 2.669888000

H -0.154795000 1.459937000 2.364742000

H -0.683161000 0.713510000 3.876627000

C -2.638573000 -2.281977000 -0.402114000

H -1.690789000 -2.790065000 -0.559401000

C -3.961986000 -2.771486000 -0.691382000

C -4.324526000 -4.073279000 -1.262594000

O -5.487831000 -4.406973000 -1.456172000

C -4.829498000 -1.770370000 -0.326328000

O -4.129606000 -0.721587000 0.157018000

C -3.183448000 -4.991302000 -1.600966000

H -2.512683000 -4.519482000 -2.330150000

H -2.580953000 -5.204867000 -0.708964000

H -3.568894000 -5.927079000 -2.013102000

C -6.295264000 -1.615153000 -0.375315000

H -6.572411000 -0.728365000 -0.959638000

H -6.738376000 -2.502803000 -0.830318000

H -6.712151000 -1.483541000 0.631185000

**TS_8B-9-cis_**

E = -2363.16422593 a.u.

C -4.723229000 4.772942000 1.220263000

C -4.949754000 3.807818000 0.240743000

C -3.883268000 3.110472000 -0.312448000

C -2.565248000 3.350123000 0.101182000

C -2.352543000 4.326211000 1.076860000

C -3.420896000 5.029910000 1.632454000

H -5.556374000 5.319354000 1.656928000

H -5.963519000 3.595198000 -0.092833000

H -1.345667000 4.546066000 1.424273000

H -3.228114000 5.783043000 2.393924000

C -1.421708000 2.561475000 -0.540916000

C -0.047076000 2.965513000 -0.007746000

H 0.095557000 2.776003000 1.057602000

H 0.737142000 2.429510000 -0.556387000

H 0.113357000 4.036854000 -0.184929000

C -1.404356000 2.916089000 -2.039524000

H -0.605313000 2.370611000 -2.561309000

H -2.354953000 2.683099000 -2.533161000

H -1.210980000 3.988314000 -2.166397000

C -1.761280000 1.051168000 -0.448550000

H -2.290748000 0.765356000 -1.362951000

H -0.396305000 0.510037000 -1.397392000

C 2.761583000 0.755424000 -1.823309000

C 2.203453000 1.532171000 -2.849385000

C 2.668708000 2.810437000 -3.128374000

C 3.709781000 3.341717000 -2.373471000

C 4.275613000 2.580216000 -1.359109000

C 3.819415000 1.288827000 -1.062582000

H 1.371859000 1.142086000 -3.434840000

H 2.212147000 3.388893000 -3.928542000

H 4.081323000 4.344139000 -2.573642000

H 5.095871000 2.985627000 -0.766914000

C 4.498768000 0.551480000 0.041121000

C 5.681119000 -0.158660000 -0.225081000

C 3.979073000 0.590883000 1.347098000

C 6.302567000 -0.855573000 0.812872000

C 4.626834000 -0.120771000 2.356232000

C 5.786771000 -0.857175000 2.107691000

H 7.214552000 -1.416059000 0.601620000

H 4.219174000 -0.091933000 3.368407000

P 1.877878000 -0.824742000 -1.456694000

Pd -0.073043000 -0.429246000 -0.193695000

C 1.513200000 -1.530620000 -3.112640000

C 3.053716000 -2.077023000 -0.816715000

C 6.446755000 -1.631839000 3.206926000

H 7.448535000 -1.967478000 2.918041000

H 6.536369000 -1.033489000 4.121611000

H 5.863331000 -2.523837000 3.471032000

C 6.263426000 -0.196096000 -1.606283000

H 6.580389000 0.799869000 -1.942445000

H 7.138140000 -0.853685000 -1.647191000

H 5.535164000 -0.555492000 -2.346211000

C 2.744153000 1.380572000 1.660347000

H 1.868179000 0.992943000 1.117138000

H 2.515523000 1.343559000 2.731450000

H 2.853152000 2.435441000 1.373615000

H 1.108568000 -2.536805000 -2.953783000

H 0.758908000 -0.943223000 -3.642933000

H 2.426298000 -1.598614000 -3.715535000

H 2.511217000 -3.028462000 -0.842676000

H 3.347165000 -1.867461000 0.214070000

H 3.938976000 -2.143827000 -1.458753000

C 0.620825000 -2.972324000 1.124943000

C 1.212411000 -3.813797000 2.236315000

H 0.665424000 -3.642370000 3.170970000

H 1.183962000 -4.878114000 1.989095000

H 2.252229000 -3.513462000 2.416085000

O 0.217030000 -3.505715000 0.079656000

H -4.082765000 2.350613000 -1.066903000

O 0.599738000 -1.713458000 1.378786000

C -2.068514000 0.129660000 0.602917000

C -2.956357000 -0.953581000 0.090002000

C -2.167536000 0.339393000 2.136357000

C -3.486998000 1.048309000 2.480759000

H -4.354301000 0.530190000 2.055042000

H -3.610584000 1.063232000 3.572486000

H -3.498528000 2.084242000 2.128212000

C -2.187222000 -1.019688000 2.849363000

H -1.290387000 -1.604991000 2.617550000

H -2.218187000 -0.850915000 3.933718000

H -3.072933000 -1.612004000 2.589430000

C -1.022053000 1.155704000 2.726109000

H -0.043609000 0.759945000 2.426351000

H -1.087056000 2.211321000 2.444566000

H -1.078715000 1.119507000 3.822241000

C -2.798302000 -2.265799000 -0.213804000

H -1.868270000 -2.821022000 -0.111565000

C -4.069867000 -2.741775000 -0.692785000

C -4.401220000 -4.095102000 -1.154217000

O -5.524952000 -4.404312000 -1.532994000

C -4.924417000 -1.666170000 -0.644280000

O -4.263163000 -0.585193000 -0.179168000

C -3.280841000 -5.096612000 -1.144027000

H -2.459100000 -4.758145000 -1.787884000

H -2.867075000 -5.206708000 -0.133873000

H -3.645722000 -6.063957000 -1.497876000

C -6.347573000 -1.474937000 -0.981557000

H -6.470054000 -0.703048000 -1.751515000

H -6.760313000 -2.416647000 -1.347489000

H -6.917865000 -1.151260000 -0.101870000

**IM9-cis**

E = -2363.16605201 a.u.

C -4.609864000 4.956250000 1.194181000

C -4.892542000 4.006832000 0.213535000

C -3.868588000 3.250110000 -0.341934000

C -2.538219000 3.414708000 0.069353000

C -2.268792000 4.375224000 1.045932000

C -3.294599000 5.137661000 1.605027000

H -5.409819000 5.549584000 1.631654000

H -5.917163000 3.854064000 -0.119712000

H -1.249852000 4.535573000 1.390969000

H -3.057726000 5.877359000 2.367222000

C -1.442039000 2.565679000 -0.579973000

C -0.043030000 2.914241000 -0.069221000

H 0.099717000 2.746906000 0.999904000

H 0.701749000 2.315248000 -0.605958000

H 0.171681000 3.970808000 -0.277240000

C -1.432205000 2.911075000 -2.080971000

H -0.659741000 2.334850000 -2.607849000

H -2.395821000 2.704512000 -2.560881000

H -1.208931000 3.976225000 -2.217603000

C -1.841459000 1.076371000 -0.472343000

H -2.271337000 0.753063000 -1.424023000

H -0.169285000 0.317134000 -1.720786000

C 2.833707000 0.686955000 -1.825771000

C 2.338000000 1.429377000 -2.907393000

C 2.842059000 2.684977000 -3.217056000

C 3.856693000 3.228838000 -2.434831000

C 4.363201000 2.498726000 -1.368175000

C 3.871704000 1.227071000 -1.043403000

H 1.526961000 1.029567000 -3.514550000

H 2.438057000 3.236677000 -4.062778000

H 4.255725000 4.215839000 -2.657297000

H 5.165955000 2.911379000 -0.757517000

C 4.502382000 0.514745000 0.104329000

C 5.673166000 -0.231680000 -0.106538000

C 3.944311000 0.605441000 1.391635000

C 6.246976000 -0.910332000 0.970148000

C 4.545368000 -0.089214000 2.440930000

C 5.693778000 -0.859617000 2.248533000

H 7.150789000 -1.498206000 0.803207000

H 4.108667000 -0.021731000 3.439099000

P 1.916534000 -0.871840000 -1.452367000

Pd -0.071132000 -0.394699000 -0.347314000

C 1.684186000 -1.674521000 -3.084132000

C 3.024925000 -2.081768000 -0.640418000

C 6.302227000 -1.619474000 3.387119000

H 7.310722000 -1.970426000 3.143235000

H 6.363410000 -1.005636000 4.293821000

H 5.699652000 -2.500883000 3.643831000

C 6.291452000 -0.332285000 -1.469358000

H 6.626971000 0.645006000 -1.840234000

H 7.159444000 -0.999752000 -1.458806000

H 5.579565000 -0.718037000 -2.212783000

C 2.712591000 1.422879000 1.638368000

H 1.846088000 1.015371000 1.094635000

H 2.458650000 1.440857000 2.704271000

H 2.838044000 2.460506000 1.300401000

H 1.209605000 -2.646099000 -2.905287000

H 1.028849000 -1.088924000 -3.733586000

H 2.654514000 -1.827564000 -3.571339000

H 2.488560000 -3.035892000 -0.662922000

H 3.234377000 -1.814343000 0.397196000

H 3.959287000 -2.168636000 -1.205090000

C 0.520688000 -2.847097000 1.191396000

C 1.048575000 -3.630900000 2.375295000

H 0.466640000 -3.399867000 3.275251000

H 1.017252000 -4.707213000 2.187268000

H 2.084490000 -3.332775000 2.581004000

O 0.193707000 -3.435381000 0.147072000

H -4.110379000 2.504370000 -1.099022000

O 0.471856000 -1.579287000 1.382897000

C -2.151437000 0.170951000 0.550850000

C -2.992748000 -0.951534000 0.046328000

C -2.238258000 0.367879000 2.086057000

C -3.525487000 1.140991000 2.419213000

H -4.413105000 0.669851000 1.980120000

H -3.659866000 1.153995000 3.509561000

H -3.480421000 2.178224000 2.074396000

C -2.335663000 -0.987037000 2.799064000

H -1.468914000 -1.616945000 2.576786000

H -2.366113000 -0.814377000 3.882686000

H -3.249067000 -1.533737000 2.532879000

C -1.056039000 1.124686000 2.681647000

H -0.103234000 0.661967000 2.398778000

H -1.055596000 2.178624000 2.387048000

H -1.130335000 1.105115000 3.777206000

C -2.786351000 -2.253822000 -0.266762000

H -1.829863000 -2.768894000 -0.200172000

C -4.059679000 -2.792640000 -0.668223000

C -4.348243000 -4.164361000 -1.104220000

O -5.478991000 -4.540066000 -1.390593000

C -4.962354000 -1.761228000 -0.567477000

O -4.327956000 -0.646943000 -0.143453000

C -3.172400000 -5.096250000 -1.187115000

H -2.423560000 -4.706645000 -1.888522000

H -2.676980000 -5.184060000 -0.212168000

H -3.504268000 -6.083020000 -1.519006000

C -6.407994000 -1.636005000 -0.831400000

H -6.602268000 -0.882107000 -1.604580000

H -6.798944000 -2.599059000 -1.163438000

H -6.945685000 -1.322117000 0.071929000

**A-r**

E = -4560.50829020 a.u.

C 2.694944000 -1.544221000 -0.089361000

C 3.676652000 -2.549081000 -0.134969000

C 5.020962000 -2.275306000 -0.345122000

C 5.426620000 -0.958083000 -0.521243000

C 4.478478000 0.053226000 -0.466745000

C 3.114444000 -0.202734000 -0.246666000

H 3.384678000 -3.587402000 -0.012792000

H 5.741870000 -3.089125000 -0.377944000

H 6.472203000 -0.716556000 -0.699372000

H 4.790829000 1.089065000 -0.598530000

C 2.265441000 1.034272000 -0.198287000

C 2.174853000 1.766084000 1.007321000

C 1.755132000 1.591317000 -1.386788000

C 1.559601000 3.019401000 0.999349000

C 1.148257000 2.849938000 -1.344240000

C 1.049290000 3.588586000 -0.169017000

H 1.509061000 3.584827000 1.931086000

H 0.761308000 3.284076000 -2.268032000

P 0.906261000 -2.064022000 0.060764000

Pd -0.812210000 -0.439663000 0.040694000

C 0.676890000 -3.349487000 -1.368311000

C 0.794692000 -2.966533000 1.755456000

C 0.440174000 4.974978000 -0.177435000

H 0.288296000 5.250116000 -1.232804000

C 2.822738000 1.277888000 2.290516000

H 3.056694000 0.211148000 2.165301000

C 1.909270000 0.909156000 -2.731467000

H 2.329773000 -0.091590000 -2.560351000

C -1.768208000 1.455129000 2.331684000

C -1.751641000 1.481158000 0.927878000

C -2.681988000 0.709275000 0.188062000

C -3.634495000 -0.139225000 0.842645000

C -3.584139000 -0.124573000 2.239534000

C -2.696556000 0.664500000 2.979747000

H -1.075140000 2.086373000 2.885246000

H -1.127289000 2.211125000 0.414917000

H -4.280633000 -0.745095000 2.794539000

H -2.743876000 0.644168000 4.066138000

Br -2.891272000 1.320290000 -1.649390000

C -4.717566000 -0.967733000 0.134719000

C -5.831202000 -0.032295000 -0.357231000

H -5.476420000 0.693433000 -1.095706000

H -6.632359000 -0.621698000 -0.822621000

H -6.266752000 0.523782000 0.483415000

C -5.369085000 -1.976352000 1.085446000

H -6.076834000 -2.591189000 0.515916000

H -4.629990000 -2.651215000 1.537691000

H -5.938043000 -1.495337000 1.889893000

C -4.151835000 -1.787790000 -1.030083000

H -3.670622000 -1.175006000 -1.796947000

H -3.418224000 -2.518912000 -0.664237000

H -4.967815000 -2.342615000 -1.512508000

C -0.574844000 -3.641392000 1.837367000

H -0.730961000 -4.006791000 2.863027000

H -1.387406000 -2.936497000 1.606137000

H -0.665117000 -4.504569000 1.166328000

C 0.852793000 -1.866622000 2.814171000

H 0.752651000 -2.316847000 3.812949000

H 1.810067000 -1.329346000 2.788782000

H 0.044930000 -1.133775000 2.682092000

C 1.886089000 -3.980408000 2.085885000

H 1.656830000 -4.427884000 3.064303000

H 1.958920000 -4.800333000 1.364656000

H 2.870830000 -3.507419000 2.175060000

C -0.823017000 -3.401009000 -1.670300000

H -0.997853000 -4.099631000 -2.502323000

H -1.411207000 -3.753739000 -0.814629000

H -1.209213000 -2.416620000 -1.969084000

C 1.381671000 -2.805580000 -2.608391000

H 1.122133000 -3.437909000 -3.469844000

H 1.059485000 -1.783784000 -2.843237000

H 2.473927000 -2.809074000 -2.509674000

C 1.153588000 -4.777627000 -1.116195000

H 0.631860000 -5.257803000 -0.280687000

H 0.934561000 -5.373839000 -2.014617000

H 2.232297000 -4.853243000 -0.945531000

C -0.923569000 5.006901000 0.508092000

H -0.847211000 4.688114000 1.557430000

H -1.338229000 6.022996000 0.499464000

H -1.644841000 4.349182000 0.005731000

C 1.372099000 6.010816000 0.445255000

H 0.959398000 7.020585000 0.327565000

H 1.504432000 5.836309000 1.521296000

H 2.364400000 5.993825000 -0.021487000

C 0.572989000 0.737237000 -3.449093000

H 0.139214000 1.705373000 -3.733201000

H 0.703206000 0.155563000 -4.371503000

H -0.162687000 0.225300000 -2.810620000

C 2.901230000 1.668534000 -3.610800000

H 2.542023000 2.683464000 -3.828036000

H 3.881788000 1.758176000 -3.125558000

H 3.044470000 1.154377000 -4.569873000

C 4.143682000 2.009043000 2.530972000

H 4.632101000 1.639809000 3.441991000

H 4.841367000 1.873452000 1.695074000

H 3.975249000 3.087502000 2.655641000

C 1.920662000 1.412233000 3.512822000

H 2.387417000 0.929695000 4.381381000

H 1.749580000 2.463158000 3.781883000

H 0.942572000 0.942792000 3.351042000

**TS1-r**

E = -4560.49598207 a.u.

C 3.080831000 -0.578521000 2.667751000

C 2.027387000 -1.313811000 2.137251000

C 2.001995000 -1.640014000 0.768373000

C 3.074479000 -1.314917000 -0.101190000

C 4.127844000 -0.608164000 0.495779000

C 4.147362000 -0.245477000 1.841115000

H 3.079918000 -0.308033000 3.721871000

H 1.228694000 -1.672245000 2.784913000

H 4.982670000 -0.326248000 -0.115887000

H 5.007911000 0.288767000 2.238682000

Br 1.000656000 -3.626420000 0.560468000

C 3.190438000 -1.711410000 -1.579922000

C 3.908232000 -0.621704000 -2.385047000

H 3.380598000 0.339159000 -2.303246000

H 3.925179000 -0.906025000 -3.444853000

H 4.950231000 -0.470052000 -2.081636000

C 4.007480000 -3.006879000 -1.668686000

H 4.107201000 -3.323121000 -2.716052000

H 3.520882000 -3.819199000 -1.114039000

H 5.016604000 -2.869370000 -1.258898000

C 1.836297000 -1.926094000 -2.260876000

H 1.975046000 -1.990314000 -3.348942000

H 1.161259000 -1.082418000 -2.051819000

H 1.339434000 -2.844331000 -1.936371000

C -2.899867000 1.029805000 -0.224418000

C -4.279991000 1.272626000 -0.335795000

C -4.810760000 2.553254000 -0.411176000

C -3.955948000 3.647938000 -0.372571000

C -2.590418000 3.433466000 -0.258443000

C -2.033051000 2.145533000 -0.185336000

H -4.971265000 0.436060000 -0.362581000

H -5.886557000 2.689475000 -0.495319000

H -4.346542000 4.661641000 -0.426794000

H -1.915796000 4.288505000 -0.220340000

C -0.537769000 2.140308000 -0.051846000

C 0.045962000 2.225707000 1.233520000

C 0.270417000 2.319520000 -1.193222000

C 1.415737000 2.454917000 1.340640000

C 1.642435000 2.549001000 -1.033096000

C 2.234908000 2.635147000 0.223711000

H 1.863550000 2.537267000 2.333290000

H 2.249972000 2.708354000 -1.923789000

P -2.289934000 -0.729686000 -0.089886000

Pd 0.065927000 -1.175631000 0.262096000

C -2.838059000 -1.545857000 -1.742420000

C -3.303973000 -1.493217000 1.369123000

C 3.685450000 3.018195000 0.435390000

H 4.096038000 2.317000000 1.181737000

C -0.797524000 2.221846000 2.494143000

H -1.783199000 1.809753000 2.241382000

C -0.319423000 2.399170000 -2.589747000

H -1.340965000 1.996813000 -2.547953000

C -2.578178000 -3.048047000 -1.628216000

H -3.297992000 -3.548988000 -0.969032000

H -2.677189000 -3.503856000 -2.624418000

H -1.564635000 -3.262152000 -1.260002000

C -1.912648000 -0.973592000 -2.814362000

H -2.151353000 -1.437178000 -3.782878000

H -2.044399000 0.110116000 -2.927447000

H -0.856929000 -1.176974000 -2.596514000

C -4.273355000 -1.311189000 -2.205576000

H -4.425901000 -1.869867000 -3.140951000

H -5.028003000 -1.660107000 -1.494021000

H -4.467270000 -0.255968000 -2.429544000

C -4.742193000 -1.915023000 1.080311000

H -4.804803000 -2.700345000 0.319192000

H -5.168772000 -2.329299000 2.006002000

H -5.389055000 -1.083656000 0.781498000

C -3.308742000 -0.480579000 2.511753000

H -3.776640000 -0.944259000 3.392388000

H -2.290727000 -0.187921000 2.798299000

H -3.879401000 0.424965000 2.272505000

C -2.537376000 -2.727934000 1.847526000

H -1.505986000 -2.481920000 2.133727000

H -3.049129000 -3.143788000 2.728223000

H -2.490561000 -3.516646000 1.088537000

C -1.009597000 3.655444000 2.982048000

H -0.050541000 4.124306000 3.242708000

H -1.489159000 4.277423000 2.215707000

H -1.644627000 3.672764000 3.877296000

C -0.217581000 1.367742000 3.616065000

H 0.721577000 1.783188000 4.005387000

H -0.921297000 1.322196000 4.457331000

H -0.017858000 0.340137000 3.283489000

C 3.764868000 4.427767000 1.025231000

H 4.804806000 4.703040000 1.242064000

H 3.362961000 5.162489000 0.313366000

H 3.191533000 4.515445000 1.955691000

C 4.547668000 2.936861000 -0.814283000

H 4.227684000 3.669657000 -1.568004000

H 5.592331000 3.161576000 -0.567530000

H 4.517582000 1.944014000 -1.279817000

C -0.420347000 3.856358000 -3.043120000

H 0.575429000 4.317124000 -3.097426000

H -0.871667000 3.921801000 -4.041642000

H -1.030621000 4.458182000 -2.358546000

C 0.455937000 1.591922000 -3.626564000

H -0.099085000 1.563396000 -4.573236000

H 1.434985000 2.040749000 -3.840899000

H 0.623648000 0.557350000 -3.302255000

**IM1-r**

E = -4560.52098156 a.u.

C -4.025903000 -1.415596000 -2.347901000

C -2.775586000 -0.973263000 -1.931677000

C -2.282183000 -1.228847000 -0.646443000

C -3.017160000 -2.049719000 0.233027000

C -4.286579000 -2.466616000 -0.216066000

C -4.802180000 -2.156263000 -1.465526000

H -4.379272000 -1.179738000 -3.350354000

H -2.170418000 -0.408704000 -2.637898000

H -4.887415000 -3.096065000 0.437965000

H -5.788955000 -2.514091000 -1.752523000

Br 0.259532000 -2.686857000 -1.161346000

C -2.570942000 -2.652814000 1.581994000

C -3.657140000 -2.479642000 2.654822000

H -4.601266000 -2.973186000 2.400365000

H -3.311649000 -2.921507000 3.598904000

H -3.872316000 -1.419904000 2.845420000

C -1.283584000 -2.075176000 2.161294000

H -1.088867000 -2.553039000 3.132106000

H -0.417924000 -2.266321000 1.516446000

H -1.358428000 -0.995705000 2.339602000

C -2.332447000 -4.154678000 1.359594000

H -2.013183000 -4.629273000 2.298276000

H -3.239932000 -4.667724000 1.017383000

H -1.544065000 -4.309605000 0.610581000

C 0.456985000 2.827824000 0.349036000

C 0.407286000 4.197828000 0.656234000

C 1.552973000 4.972385000 0.770225000

C 2.795834000 4.387588000 0.557934000

C 2.866152000 3.030146000 0.283915000

C 1.719309000 2.222551000 0.201793000

H -0.551159000 4.683202000 0.799397000

H 1.469458000 6.029427000 1.011567000

H 3.706675000 4.978789000 0.619568000

H 3.839119000 2.557469000 0.150198000

C 2.014686000 0.757781000 0.052521000

C 2.473382000 0.256668000 -1.193550000

C 2.286332000 0.013908000 1.238025000

C 3.123851000 -0.974202000 -1.232810000

C 2.898733000 -1.231291000 1.132285000

C 3.323945000 -1.744011000 -0.092554000

H 3.468247000 -1.364625000 -2.191845000

H 3.092607000 -1.797580000 2.043322000

P -1.120183000 1.877063000 0.084639000

Pd -0.437697000 -0.415277000 -0.364202000

C -2.194866000 2.197710000 1.648350000

C -1.914247000 2.771642000 -1.430560000

C 4.038180000 -3.072006000 -0.187963000

H 3.966116000 -3.398620000 -1.237387000

C 2.338812000 1.047488000 -2.478602000

H 1.749222000 1.947810000 -2.258667000

C 2.096386000 0.616497000 2.618783000

H 1.404438000 1.464231000 2.532324000

C -1.120552000 2.323622000 -2.655227000

H -1.586644000 2.742157000 -3.558702000

H -0.089948000 2.697743000 -2.615332000

H -1.084944000 1.235153000 -2.765640000

C -1.868862000 4.299808000 -1.410666000

H -0.846307000 4.688882000 -1.456688000

H -2.390250000 4.652892000 -2.312316000

H -2.376838000 4.746269000 -0.550229000

C -3.374762000 2.342353000 -1.565794000

H -3.511536000 1.257704000 -1.524648000

H -4.009489000 2.803120000 -0.800238000

H -3.747434000 2.685187000 -2.541946000

C -2.826675000 3.581701000 1.786453000

H -3.522816000 3.814017000 0.973468000

H -3.410199000 3.587360000 2.718759000

H -2.096995000 4.392457000 1.871232000

C -1.300409000 1.943271000 2.860040000

H -0.817773000 0.959040000 2.816459000

H -0.520831000 2.707503000 2.969276000

H -1.918501000 1.965956000 3.768918000

C -3.323836000 1.167932000 1.654715000

H -3.878421000 1.263449000 2.599408000

H -4.039065000 1.313642000 0.836756000

H -2.937855000 0.149261000 1.585901000

C 3.704849000 1.508174000 -2.984193000

H 4.346692000 0.649745000 -3.222920000

H 3.595332000 2.104570000 -3.898924000

H 4.228095000 2.121374000 -2.240434000

C 1.610694000 0.260345000 -3.565041000

H 2.214035000 -0.588447000 -3.913962000

H 0.660117000 -0.147881000 -3.198091000

H 1.407240000 0.902145000 -4.432445000

C 5.517498000 -2.896960000 0.152304000

H 6.059164000 -3.846379000 0.053898000

H 5.996519000 -2.161649000 -0.506251000

H 5.636680000 -2.547168000 1.187293000

C 3.406984000 -4.151323000 0.682961000

H 3.878964000 -5.122996000 0.490860000

H 3.537587000 -3.936038000 1.752032000

H 2.332065000 -4.239825000 0.479651000

C 3.433168000 1.160790000 3.129930000

H 3.861172000 1.908710000 2.452320000

H 3.305252000 1.630415000 4.113763000

H 4.164749000 0.348513000 3.238389000

C 1.525644000 -0.352404000 3.648003000

H 2.241808000 -1.144968000 3.901263000

H 1.294839000 0.183476000 4.577652000

H 0.605100000 -0.833033000 3.299540000

**IM2-r**

E = -2217.60128513 a.u.

C 1.425806000 2.346067000 2.890959000

C 1.600642000 1.239195000 2.065695000

C 2.145761000 1.327888000 0.776767000

C 2.538847000 2.599305000 0.286997000

C 2.337640000 3.699083000 1.145047000

C 1.796663000 3.597616000 2.418485000

H 1.003027000 2.219598000 3.886667000

H 1.295160000 0.273151000 2.456422000

H 2.641741000 4.686904000 0.803886000

H 1.674154000 4.488877000 3.030760000

C 3.244241000 2.945345000 -1.042148000

C 2.522469000 4.109867000 -1.737934000

H 2.541091000 5.042594000 -1.164174000

H 3.002672000 4.317989000 -2.703417000

H 1.471243000 3.857763000 -1.937209000

C 3.308492000 1.822068000 -2.069887000

H 3.864143000 2.179399000 -2.948061000

H 3.829413000 0.932510000 -1.695023000

H 2.311647000 1.533843000 -2.415105000

C 4.688604000 3.357183000 -0.723724000

H 5.209626000 3.652551000 -1.645154000

H 4.735427000 4.204601000 -0.028743000

H 5.238421000 2.517780000 -0.276867000

C -0.494928000 -1.247357000 1.161632000

C 0.309194000 -1.743426000 2.207841000

C -0.161223000 -1.984144000 3.489106000

C -1.486614000 -1.695601000 3.783899000

C -2.282715000 -1.145314000 2.792329000

C -1.830065000 -0.899317000 1.482711000

H 1.365318000 -1.924274000 2.014439000

H 0.512710000 -2.376955000 4.246782000

H -1.892473000 -1.869928000 4.777592000

H -3.313058000 -0.877852000 3.023970000

C -2.861276000 -0.232040000 0.623923000

C -3.882670000 -0.988691000 0.018756000

C -2.916173000 1.181150000 0.577445000

C -4.873261000 -0.328409000 -0.714676000

C -3.922817000 1.792564000 -0.168228000

C -4.899407000 1.055929000 -0.840166000

H -5.655752000 -0.912471000 -1.200951000

H -3.954103000 2.881055000 -0.219581000

P 0.551740000 -1.294330000 -0.406782000

Pd 2.711682000 -0.439707000 -0.010837000

C 0.685471000 -3.213938000 -0.682503000

C -0.165761000 -0.482608000 -1.987547000

C -5.961466000 1.744162000 -1.667300000

H -6.619940000 0.959312000 -2.070192000

C -3.972904000 -2.494864000 0.176329000

H -3.009820000 -2.850130000 0.566318000

C -1.952685000 2.043235000 1.377705000

H -1.027166000 1.469839000 1.535460000

C 5.116703000 -1.308963000 -0.221545000

O 4.172038000 -2.047488000 -0.632416000

O 4.874244000 -0.165983000 0.280157000

C 6.528297000 -1.800858000 -0.293753000

H 7.238289000 -0.969976000 -0.291522000

H 6.731261000 -2.426651000 0.583548000

H 6.674579000 -2.419331000 -1.183675000

C 1.273670000 -3.507681000 -2.062227000

H 0.589051000 -3.254635000 -2.876593000

H 2.234728000 -3.006361000 -2.225149000

H 1.454404000 -4.589551000 -2.127229000

C 1.610062000 -3.865423000 0.349847000

H 1.771148000 -4.907201000 0.038534000

H 2.591071000 -3.378409000 0.401089000

H 1.169890000 -3.905112000 1.350098000

C -0.687949000 -3.869822000 -0.574471000

H -1.104911000 -3.779324000 0.435816000

H -1.410242000 -3.465004000 -1.291667000

H -0.576705000 -4.942806000 -0.788689000

C -1.464177000 -1.076659000 -2.519293000

H -2.278120000 -1.024022000 -1.791170000

H -1.769482000 -0.490322000 -3.398855000

H -1.346170000 -2.114907000 -2.849147000

C 0.892902000 -0.568279000 -3.099052000

H 0.724540000 0.263147000 -3.797253000

H 1.921844000 -0.489961000 -2.730704000

H 0.809916000 -1.490932000 -3.677076000

C -0.393022000 0.985899000 -1.641711000

H -1.195433000 1.097004000 -0.911162000

H 0.509075000 1.464156000 -1.244337000

H -0.696079000 1.525060000 -2.551363000

C -5.047923000 -2.855865000 1.202259000

H -5.097340000 -3.943034000 1.345009000

H -4.856730000 -2.396331000 2.179789000

H -6.037067000 -2.518543000 0.863461000

C -4.247611000 -3.232457000 -1.131773000

H -4.139110000 -4.314475000 -0.982621000

H -5.271156000 -3.058816000 -1.488683000

H -3.560534000 -2.932730000 -1.932774000

C -6.813354000 2.686811000 -0.822217000

H -7.602286000 3.147483000 -1.429949000

H -7.293101000 2.160411000 0.011783000

H -6.204772000 3.498280000 -0.401581000

C -5.343165000 2.491813000 -2.846296000

H -4.701865000 3.313424000 -2.500064000

H -4.728498000 1.828767000 -3.467864000

H -6.122304000 2.927577000 -3.484259000

C -1.568374000 3.358626000 0.705551000

H -2.414533000 4.057389000 0.664846000

H -0.777287000 3.850176000 1.286800000

H -1.193946000 3.223360000 -0.316600000

C -2.550654000 2.343897000 2.753557000

H -2.762130000 1.429685000 3.320912000

H -1.865315000 2.961384000 3.349356000

H -3.493513000 2.898293000 2.647108000

**TS_2-3­_-r**

E = -2217.56495287 a.u.

C 1.077005000 3.748871000 0.046675000

C 1.152177000 2.396395000 -0.285834000

C 2.368963000 1.707441000 -0.287351000

C 3.549014000 2.439250000 -0.033344000

C 3.460637000 3.789462000 0.321701000

C 2.234437000 4.444551000 0.376967000

H 0.107956000 4.249705000 0.055007000

H 0.230811000 1.874890000 -0.531679000

H 4.371024000 4.346205000 0.544947000

H 2.188621000 5.495722000 0.655381000

C 4.886844000 1.741898000 -0.169674000

C 4.673474000 0.556356000 -1.115422000

H 4.241649000 0.886657000 -2.073228000

H 5.645072000 0.128100000 -1.416050000

H 4.611823000 -0.673756000 -0.477918000

C 5.357315000 1.251911000 1.204222000

H 6.304093000 0.699624000 1.117794000

H 5.523201000 2.098798000 1.883879000

H 4.616436000 0.585250000 1.667478000

C -0.406761000 0.062448000 1.291827000

C 0.606026000 0.429568000 2.199744000

C 0.368714000 1.123469000 3.376254000

C -0.931029000 1.496323000 3.687713000

C -1.943999000 1.188314000 2.793329000

C -1.730563000 0.495395000 1.588108000

H 1.638001000 0.180575000 1.958804000

H 1.198811000 1.373372000 4.032984000

H -1.155926000 2.039108000 4.602972000

H -2.961805000 1.509043000 3.013116000

C -2.966242000 0.395544000 0.736400000

C -3.927764000 -0.615574000 0.949573000

C -3.253133000 1.423679000 -0.192125000

C -5.042126000 -0.681228000 0.111087000

C -4.367859000 1.302306000 -1.023878000

C -5.255998000 0.235339000 -0.914932000

H -5.770441000 -1.481991000 0.251899000

H -4.553895000 2.076626000 -1.767528000

P 0.409676000 -1.031923000 -0.027608000

Pd 2.614575000 -0.259926000 -0.680068000

C 0.576599000 -2.701659000 0.948901000

C -0.595656000 -1.299296000 -1.630046000

C -6.387246000 0.021668000 -1.893608000

H -7.188425000 -0.511105000 -1.357303000

C -3.833827000 -1.597814000 2.099725000

H -2.803467000 -1.580404000 2.473994000

C -2.457849000 2.718322000 -0.228575000

H -1.469505000 2.526587000 0.209195000

C 4.252363000 -2.670175000 -0.689042000

O 5.009107000 -1.853471000 -0.074353000

O 3.183815000 -2.317855000 -1.256360000

C 4.627137000 -4.120197000 -0.690633000

H 4.249535000 -4.621379000 -1.585564000

H 5.708317000 -4.251855000 -0.605285000

H 4.157267000 -4.592338000 0.182900000

C 0.790176000 -3.893337000 0.014326000

H 1.648568000 -3.758919000 -0.648595000

H 0.979839000 -4.776428000 0.641741000

H -0.096387000 -4.119031000 -0.587781000

C 1.777274000 -2.609830000 1.893666000

H 1.915398000 -3.590081000 2.372410000

H 2.710246000 -2.355780000 1.376518000

H 1.623388000 -1.880472000 2.696704000

C -0.668976000 -2.972502000 1.783876000

H -0.555897000 -3.948419000 2.278818000

H -0.813767000 -2.220402000 2.568344000

H -1.571220000 -3.020904000 1.165382000

C 0.298254000 -2.017138000 -2.645822000

H 0.491681000 -3.064016000 -2.398192000

H -0.221570000 -1.995641000 -3.614357000

H 1.263914000 -1.511333000 -2.767957000

C -1.901822000 -2.067599000 -1.477008000

H -2.608862000 -1.559834000 -0.816223000

H -2.372445000 -2.150549000 -2.468370000

H -1.750579000 -3.087137000 -1.105322000

C -0.876325000 0.089539000 -2.195686000

H -1.430519000 0.709835000 -1.491312000

H 0.049240000 0.611210000 -2.473178000

H -1.493351000 -0.011461000 -3.101487000

C -4.743840000 -1.149565000 3.244860000

H -5.792944000 -1.123754000 2.919963000

H -4.672994000 -1.846711000 4.089812000

H -4.481209000 -0.148940000 3.609144000

C -4.176659000 -3.033337000 1.707643000

H -3.881280000 -3.725264000 2.506892000

H -5.255717000 -3.163038000 1.552185000

H -3.672294000 -3.347714000 0.785200000

C -6.966702000 1.308914000 -2.463576000

H -6.244147000 1.824162000 -3.110385000

H -7.848027000 1.089489000 -3.078192000

H -7.270866000 2.007848000 -1.674919000

C -5.896979000 -0.885349000 -3.023677000

H -5.062555000 -0.408600000 -3.558585000

H -5.538259000 -1.848625000 -2.638786000

H -6.695458000 -1.084181000 -3.749945000

C -2.259329000 3.316870000 -1.618611000

H -3.195341000 3.727008000 -2.019163000

H -1.546161000 4.149314000 -1.565965000

H -1.872653000 2.594277000 -2.346851000

C -3.148821000 3.755543000 0.661159000

H -4.152906000 3.981748000 0.276731000

H -3.258403000 3.407506000 1.695126000

H -2.576610000 4.692795000 0.678453000

C 5.960274000 2.662960000 -0.753363000

H 5.642200000 3.083711000 -1.716701000

H 6.212305000 3.496411000 -0.084837000

H 6.884485000 2.094094000 -0.922436000

**IM3-r**

E = -2217.58743538 a.u.

C 1.284793000 3.836515000 0.442710000

C 1.310093000 2.486286000 0.086103000

C 2.514461000 1.806185000 -0.119207000

C 3.714906000 2.542143000 0.000344000

C 3.688313000 3.888843000 0.364535000

C 2.476496000 4.538533000 0.593604000

H 0.327882000 4.335494000 0.603857000

H 0.367269000 1.955717000 -0.026960000

H 4.623637000 4.441751000 0.462998000

H 2.464318000 5.589549000 0.876971000

C 4.977400000 1.766495000 -0.276955000

C 4.530055000 0.615993000 -1.182778000

H 4.356337000 0.991619000 -2.204849000

H 5.296851000 -0.173513000 -1.255935000

H 4.419992000 -1.342050000 0.510177000

C 5.547301000 1.234533000 1.041975000

H 6.428251000 0.601197000 0.860728000

H 5.857606000 2.060649000 1.696805000

H 4.801410000 0.644489000 1.594233000

C -0.492495000 -0.151014000 1.354366000

C 0.495776000 0.075175000 2.333890000

C 0.230382000 0.603960000 3.589030000

C -1.072360000 0.959315000 3.907643000

C -2.057781000 0.807650000 2.944140000

C -1.815495000 0.276756000 1.664653000

H 1.532181000 -0.162150000 2.092038000

H 1.040368000 0.741822000 4.301728000

H -1.319437000 1.373302000 4.882545000

H -3.074543000 1.126344000 3.172253000

C -3.014476000 0.330306000 0.758912000

C -4.014814000 -0.664865000 0.797486000

C -3.227748000 1.479849000 -0.037457000

C -5.091087000 -0.581724000 -0.087147000

C -4.308447000 1.508532000 -0.921301000

C -5.231759000 0.468193000 -0.990670000

H -5.848563000 -1.367405000 -0.080111000

H -4.435918000 2.380903000 -1.560309000

P 0.365224000 -1.087404000 -0.059310000

Pd 2.686088000 -0.145626000 -0.621487000

C 0.396158000 -2.877831000 0.688205000

C -0.588590000 -1.110770000 -1.716518000

C -6.330912000 0.417269000 -2.027263000

H -7.174799000 -0.129611000 -1.577578000

C -4.008845000 -1.784139000 1.819120000

H -2.997347000 -1.854150000 2.234918000

C -2.385156000 2.735717000 0.124278000

H -1.412570000 2.444089000 0.542789000

C 4.193506000 -2.808517000 -0.625517000

O 4.779539000 -2.257468000 0.426761000

O 3.348963000 -2.215699000 -1.292770000

C 4.604082000 -4.212039000 -0.876156000

H 4.310832000 -4.521975000 -1.880183000

H 5.679445000 -4.341146000 -0.729331000

H 4.090204000 -4.848709000 -0.143696000

C 0.710151000 -3.922125000 -0.383513000

H 1.607448000 -3.689273000 -0.964615000

H 0.879721000 -4.885436000 0.119581000

H -0.124691000 -4.071672000 -1.076228000

C 1.486453000 -2.952320000 1.758302000

H 1.531735000 -3.983746000 2.136983000

H 2.482309000 -2.698963000 1.376217000

H 1.278055000 -2.303997000 2.616577000

C -0.927389000 -3.245823000 1.343216000

H -0.882383000 -4.290548000 1.686130000

H -1.130189000 -2.620679000 2.220595000

H -1.768352000 -3.162764000 0.646990000

C 0.344677000 -1.694218000 -2.783757000

H 0.504713000 -2.770645000 -2.680079000

H -0.116178000 -1.521599000 -3.767392000

H 1.325340000 -1.199461000 -2.782560000

C -1.914548000 -1.862526000 -1.750978000

H -2.646304000 -1.455411000 -1.047083000

H -2.342556000 -1.770616000 -2.761381000

H -1.796315000 -2.933564000 -1.549494000

C -0.813542000 0.352477000 -2.088021000

H -1.364047000 0.889694000 -1.315772000

H 0.135799000 0.874591000 -2.267867000

H -1.407777000 0.403892000 -3.013438000

C -4.953092000 -1.438835000 2.971978000

H -5.984104000 -1.327348000 2.609381000

H -4.948065000 -2.234935000 3.727691000

H -4.670113000 -0.501467000 3.466753000

C -4.386840000 -3.145672000 1.238670000

H -4.135339000 -3.944434000 1.948146000

H -5.465428000 -3.215755000 1.045217000

H -3.870374000 -3.358118000 0.293946000

C -6.833459000 1.780938000 -2.478678000

H -6.070393000 2.321375000 -3.054295000

H -7.705516000 1.664553000 -3.133246000

H -7.128123000 2.412854000 -1.632059000

C -5.837282000 -0.389359000 -3.229713000

H -4.957521000 0.096139000 -3.677366000

H -5.543801000 -1.406140000 -2.939350000

H -6.611858000 -0.465825000 -4.003473000

C -2.135005000 3.513647000 -1.165166000

H -3.053159000 3.985706000 -1.538797000

H -1.417244000 4.322350000 -0.977098000

H -1.729258000 2.888167000 -1.969312000

C -3.056575000 3.660560000 1.142944000

H -4.044800000 3.977626000 0.781851000

H -3.196641000 3.170997000 2.114195000

H -2.452189000 4.563100000 1.302644000

C 6.057412000 2.600016000 -0.968201000

H 5.674443000 3.063836000 -1.886999000

H 6.445914000 3.400095000 -0.322729000

H 6.909133000 1.961726000 -1.241859000

**IM1b-r**

E = -2002.59715200 a.u.

C -4.906967000 -2.626347000 1.216443000

C -3.811607000 -1.777731000 1.064056000

C -3.796601000 -0.759351000 0.106956000

C -4.940403000 -0.530562000 -0.699592000

C -6.009141000 -1.427956000 -0.547215000

C -5.999859000 -2.467474000 0.377804000

H -4.884785000 -3.411455000 1.969878000

H -2.947573000 -1.932392000 1.705255000

H -6.898362000 -1.301705000 -1.159379000

H -6.857258000 -3.133424000 0.449397000

Br -1.437954000 2.843034000 1.229727000

C -5.104670000 0.701079000 -1.598093000

C -6.444425000 0.726594000 -2.334090000

H -7.299413000 0.756142000 -1.647327000

H -6.493132000 1.629627000 -2.955412000

H -6.564398000 -0.137877000 -2.999703000

C -4.002745000 0.779158000 -2.651889000

H -4.152789000 1.657346000 -3.294148000

H -3.016508000 0.872246000 -2.181447000

H -4.000117000 -0.114090000 -3.291742000

C -5.052696000 1.950749000 -0.711110000

H -5.194960000 2.860200000 -1.311023000

H -5.830762000 1.922597000 0.062834000

H -4.079015000 2.061868000 -0.197423000

C 0.556784000 -0.440092000 1.335590000

C -0.304367000 -0.544564000 2.446837000

C 0.130830000 -0.484484000 3.760245000

C 1.480429000 -0.283218000 4.014882000

C 2.344682000 -0.145039000 2.941563000

C 1.930586000 -0.220507000 1.598245000

H -1.371938000 -0.671613000 2.278554000

H -0.587443000 -0.580414000 4.571087000

H 1.857456000 -0.219983000 5.032938000

H 3.400520000 0.042673000 3.131781000

C 3.052219000 0.013804000 0.634167000

C 3.348311000 1.335716000 0.232549000

C 3.920335000 -1.038541000 0.276413000

C 4.460831000 1.559523000 -0.579496000

C 5.006945000 -0.764479000 -0.557158000

C 5.288785000 0.523354000 -1.004872000

H 4.690916000 2.573756000 -0.907614000

H 5.658176000 -1.584612000 -0.858793000

P -0.454024000 -0.738277000 -0.236366000

Pd -2.357073000 0.615560000 0.300606000

C 0.179727000 -0.016310000 -1.915643000

C -0.476794000 -2.677862000 -0.362227000

C 6.403517000 0.796664000 -1.987835000

H 6.610199000 1.877600000 -1.952994000

C 2.502136000 2.515661000 0.674400000

H 1.479318000 2.152181000 0.858700000

C 3.761942000 -2.447731000 0.821649000

H 2.712408000 -2.581724000 1.124974000

C 4.110840000 -3.539879000 -0.189526000

H 5.196595000 -3.630399000 -0.323618000

H 3.751891000 -4.512334000 0.170502000

H 3.670427000 -3.362938000 -1.178902000

C 4.628321000 -2.636112000 2.068959000

H 4.535592000 -3.660157000 2.453123000

H 5.686109000 -2.461818000 1.828884000

H 4.349780000 -1.947833000 2.874662000

C 7.695472000 0.055945000 -1.666332000

H 8.494320000 0.364329000 -2.351796000

H 8.033822000 0.253640000 -0.642105000

H 7.576504000 -1.029822000 -1.777093000

C 5.924953000 0.456927000 -3.399518000

H 5.691919000 -0.614366000 -3.478788000

H 5.016060000 1.014913000 -3.659549000

H 6.694192000 0.690232000 -4.146721000

C 2.420890000 3.631810000 -0.363767000

H 1.625626000 4.336192000 -0.088883000

H 3.355730000 4.205852000 -0.415076000

H 2.206839000 3.256581000 -1.372319000

C 3.022915000 3.103044000 1.987149000

H 2.401632000 3.954088000 2.297661000

H 3.020120000 2.370310000 2.801795000

H 4.052617000 3.467254000 1.863037000

C -0.070299000 1.489556000 -1.881771000

H 0.414518000 1.971419000 -1.027162000

H 0.340382000 1.925243000 -2.804423000

H -1.136616000 1.737620000 -1.836223000

C 1.647361000 -0.224756000 -2.291220000

H 1.749460000 0.073880000 -3.344815000

H 2.307876000 0.416433000 -1.706746000

H 1.995921000 -1.255698000 -2.212058000

C -0.683393000 -0.632818000 -3.020793000

H -0.364127000 -1.646400000 -3.284125000

H -1.750769000 -0.651018000 -2.782651000

H -0.561929000 -0.011956000 -3.919621000

C 0.783769000 -3.153698000 -1.080452000

H 1.683133000 -2.696490000 -0.662190000

H 0.870497000 -4.240761000 -0.936805000

H 0.759203000 -2.970649000 -2.159227000

C -0.455441000 -3.305065000 1.032377000

H 0.492869000 -3.130092000 1.554756000

H -1.271409000 -2.969772000 1.678458000

H -0.564310000 -4.390908000 0.903663000

C -1.703584000 -3.201243000 -1.109065000

H -1.543271000 -4.272493000 -1.297519000

H -2.620562000 -3.104706000 -0.521033000

H -1.864587000 -2.724705000 -2.080005000

**IM2-b-r**

E = -5177.38695748 a.u.

C 2.749140000 4.306548000 0.776301000

C 2.160022000 3.048517000 0.712944000

C 2.437140000 2.114704000 -0.299814000

C 3.360290000 2.479069000 -1.308608000

C 3.959605000 3.750620000 -1.206359000

C 3.672521000 4.658793000 -0.198697000

H 2.490336000 4.988782000 1.584333000

H 1.452075000 2.795621000 1.500083000

H 4.690052000 4.042021000 -1.958157000

H 4.165442000 5.628732000 -0.181280000

Br 0.645997000 -1.647015000 1.727506000

C 3.825356000 1.625502000 -2.501880000

C 4.032769000 2.478688000 -3.762437000

H 4.904826000 3.139611000 -3.707800000

H 4.193648000 1.814497000 -4.622021000

H 3.148290000 3.094457000 -3.976919000

C 2.805202000 0.566666000 -2.889411000

H 3.235354000 -0.129275000 -3.623982000

H 2.461749000 -0.009621000 -2.028164000

H 1.921188000 1.031852000 -3.344751000

C 5.162744000 0.965984000 -2.145512000

H 5.534224000 0.375864000 -2.995794000

H 5.922298000 1.722793000 -1.906612000

H 5.078433000 0.296331000 -1.282082000

C -1.827365000 0.927984000 1.105297000

C -1.006194000 1.465815000 2.112618000

C -1.468619000 1.875386000 3.353178000

C -2.818346000 1.739406000 3.643783000

C -3.639419000 1.138844000 2.702195000

C -3.186883000 0.698064000 1.445350000

H 0.057260000 1.566796000 1.915797000

H -0.771662000 2.291969000 4.077746000

H -3.224867000 2.062683000 4.599338000

H -4.686086000 0.962939000 2.946396000

C -4.213147000 -0.088619000 0.686913000

C -4.215490000 -1.498194000 0.818089000

C -5.260420000 0.544305000 -0.018410000

C -5.154021000 -2.238064000 0.096418000

C -6.189493000 -0.242750000 -0.702766000

C -6.131191000 -1.634235000 -0.691479000

H -5.141963000 -3.326090000 0.161751000

H -6.980262000 0.250607000 -1.268537000

P -0.817887000 0.859331000 -0.502820000

Pd 1.518269000 0.335621000 0.272156000

C -1.385277000 -0.493783000 -1.776446000

C -1.172685000 2.624156000 -1.231189000

C -7.090195000 -2.467803000 -1.510858000

H -6.899582000 -3.522869000 -1.260039000

C -3.313328000 -2.217959000 1.811698000

H -2.364160000 -1.666326000 1.896547000

C -5.455562000 2.051015000 -0.019484000

H -4.501473000 2.521037000 0.261065000

C 3.576841000 -0.149232000 1.213550000

C 3.409100000 -0.915943000 0.256551000

C 4.186276000 0.439681000 2.427140000

C 3.419792000 -1.969758000 -0.695594000

H 2.539325000 -2.077786000 -1.327373000

C 4.438397000 -2.846611000 -0.821304000

C 4.417416000 -3.939361000 -1.821772000

O 5.383019000 -4.685429000 -1.911170000

C 5.639287000 -2.750589000 0.080878000

O 6.387327000 -1.791858000 0.025354000

C 3.216955000 -4.088840000 -2.709819000

H 2.315983000 -4.289394000 -2.117218000

H 3.032702000 -3.172728000 -3.284686000

H 3.386755000 -4.918260000 -3.399769000

C 5.786976000 -3.846536000 1.085663000

H 4.906402000 -3.842450000 1.744403000

H 5.806857000 -4.820551000 0.585668000

H 6.693822000 -3.705000000 1.679498000

C 4.972845000 -0.706991000 3.083795000

H 4.300865000 -1.519583000 3.387604000

H 5.727425000 -1.111211000 2.397193000

H 5.482639000 -0.327943000 3.978861000

C 5.159137000 1.550919000 2.019055000

H 4.633738000 2.394706000 1.556459000

H 5.679335000 1.917567000 2.913377000

H 5.911992000 1.173925000 1.314448000

C 3.143199000 0.962237000 3.413559000

H 3.645445000 1.242634000 4.348165000

H 2.631433000 1.850119000 3.027088000

H 2.391143000 0.194248000 3.632901000

C -5.884410000 2.610335000 -1.376390000

H -6.938649000 2.387773000 -1.585874000

H -5.781875000 3.702645000 -1.382674000

H -5.291221000 2.209005000 -2.207636000

C -6.493831000 2.454260000 1.030017000

H -6.645850000 3.541351000 1.023362000

H -7.461513000 1.980755000 0.814693000

H -6.197576000 2.161788000 2.043830000

C -8.549492000 -2.163730000 -1.187440000

H -9.216254000 -2.819494000 -1.761212000

H -8.765656000 -2.308590000 -0.122045000

H -8.809242000 -1.128356000 -1.444731000

C -6.818866000 -2.283001000 -3.002681000

H -7.020246000 -1.248344000 -3.312087000

H -5.774000000 -2.508661000 -3.251178000

H -7.460288000 -2.939476000 -3.604459000

C -2.974922000 -3.660628000 1.442710000

H -2.190725000 -4.032073000 2.113582000

H -3.841315000 -4.324862000 1.561858000

H -2.609283000 -3.769611000 0.415235000

C -3.983819000 -2.226374000 3.189459000

H -3.351209000 -2.745268000 3.921377000

H -4.177840000 -1.217524000 3.569334000

H -4.944682000 -2.757758000 3.141270000

C -1.314530000 -1.840437000 -1.069950000

H -1.968085000 -1.871626000 -0.199099000

H -1.645733000 -2.616701000 -1.775531000

H -0.305263000 -2.087809000 -0.729401000

C -2.782923000 -0.341107000 -2.383833000

H -3.045148000 -1.302681000 -2.849039000

H -3.558950000 -0.110404000 -1.653597000

H -2.812627000 0.410699000 -3.176260000

C -0.386480000 -0.530953000 -2.929339000

H -0.349508000 0.404716000 -3.498425000

H 0.623586000 -0.786305000 -2.593074000

H -0.710491000 -1.319761000 -3.623624000

C -2.535689000 2.684600000 -1.912733000

H -3.296971000 2.134289000 -1.357328000

H -2.857102000 3.735043000 -1.971952000

H -2.501343000 2.299337000 -2.936125000

C -1.183747000 3.653977000 -0.100475000

H -2.050078000 3.535642000 0.561979000

H -0.274682000 3.642432000 0.507984000

H -1.255973000 4.649390000 -0.561324000

C -0.108242000 3.031478000 -2.248417000

H -0.423496000 3.982594000 -2.701957000

H 0.873480000 3.188116000 -1.792037000

H 0.001526000 2.307996000 -3.064793000

**TS_2-3­_-b-r**

E = -5177.38245025 a.u.

C -2.702872000 -4.448039000 0.106269000

C -2.133560000 -3.191840000 0.278678000

C -2.475518000 -2.060665000 -0.485892000

C -3.446186000 -2.237667000 -1.505242000

C -4.031056000 -3.512833000 -1.640819000

C -3.682058000 -4.607452000 -0.864670000

H -2.383148000 -5.281866000 0.729543000

H -1.374569000 -3.102927000 1.052848000

H -4.799955000 -3.657632000 -2.396402000

H -4.166213000 -5.568909000 -1.024934000

Br -0.563511000 1.309307000 2.063534000

C -3.970491000 -1.167475000 -2.482417000

C -4.270384000 -1.759924000 -3.868905000

H -5.153644000 -2.408334000 -3.885569000

H -4.468257000 -0.942583000 -4.575157000

H -3.415659000 -2.335089000 -4.251284000

C -2.949797000 -0.065006000 -2.724280000

H -3.402114000 0.760079000 -3.292880000

H -2.539914000 0.331771000 -1.791769000

H -2.106264000 -0.453560000 -3.310275000

C -5.277880000 -0.582934000 -1.931847000

H -5.685973000 0.167574000 -2.624847000

H -6.034635000 -1.369494000 -1.805989000

H -5.135356000 -0.104913000 -0.956718000

C 1.870277000 -1.139303000 0.922774000

C 1.077396000 -1.866039000 1.829811000

C 1.568354000 -2.481041000 2.970212000

C 2.919667000 -2.367623000 3.264203000

C 3.712283000 -1.588013000 2.436392000

C 3.229077000 -0.943444000 1.282521000

H 0.013598000 -1.954225000 1.629025000

H 0.893143000 -3.038697000 3.616484000

H 3.348783000 -2.845615000 4.141963000

H 4.758183000 -1.432334000 2.697472000

C 4.224631000 -0.017785000 0.653382000

C 4.199533000 1.354024000 1.010822000

C 5.267399000 -0.504251000 -0.161365000

C 5.117110000 2.216761000 0.414187000

C 6.177920000 0.401570000 -0.715488000

C 6.098181000 1.767805000 -0.470436000

H 5.089729000 3.280530000 0.654185000

H 6.967464000 0.022506000 -1.363515000

P 0.817152000 -0.803845000 -0.622154000

Pd -1.487980000 -0.418435000 0.361357000

C 1.366882000 0.724305000 -1.690323000

C 1.143713000 -2.426727000 -1.639688000

C 6.992857000 2.773810000 -1.159036000

H 7.132681000 3.614346000 -0.461029000

C 3.289497000 1.884517000 2.108800000

H 2.345848000 1.317064000 2.102290000

C 5.466902000 -1.984015000 -0.439451000

H 4.520482000 -2.502090000 -0.222022000

C -3.319955000 0.030473000 1.329674000

C -3.700246000 1.049995000 0.630713000

C -3.901540000 -0.681893000 2.521435000

C -3.458087000 2.110486000 -0.246214000

H -2.461807000 2.193029000 -0.675190000

C -4.445489000 3.012810000 -0.525931000

C -4.273423000 4.141935000 -1.457786000

O -5.208272000 4.877780000 -1.739092000

C -5.668637000 2.670648000 0.186665000

O -5.573133000 1.710408000 0.977571000

C -2.906468000 4.343555000 -2.049300000

H -2.148636000 4.466875000 -1.265705000

H -2.611660000 3.474247000 -2.652357000

H -2.917411000 5.231265000 -2.685658000

C -6.962656000 3.371772000 0.005137000

H -6.847970000 4.435751000 0.237538000

H -7.275553000 3.321176000 -1.043363000

H -7.721882000 2.922532000 0.648963000

C -4.366023000 0.374819000 3.534815000

H -3.522458000 1.002809000 3.848076000

H -5.146481000 1.019793000 3.122825000

H -4.762130000 -0.133051000 4.423766000

C -5.100441000 -1.512513000 2.042704000

H -4.797717000 -2.264515000 1.302398000

H -5.533912000 -2.038792000 2.903528000

H -5.876043000 -0.876167000 1.601224000

C -2.890186000 -1.587586000 3.221090000

H -3.341576000 -1.983455000 4.140274000

H -2.602801000 -2.440326000 2.599085000

H -1.985312000 -1.029443000 3.492668000

C 5.858741000 -2.283779000 -1.886969000

H 6.906072000 -2.018444000 -2.080773000

H 5.760475000 -3.357756000 -2.089676000

H 5.241391000 -1.742836000 -2.614989000

C 6.535390000 -2.562550000 0.490270000

H 6.689066000 -3.630013000 0.286034000

H 7.495793000 -2.051977000 0.334942000

H 6.269490000 -2.456924000 1.547926000

C 8.364931000 2.232778000 -1.532866000

H 8.998886000 3.039935000 -1.919105000

H 8.878006000 1.782564000 -0.674286000

H 8.297867000 1.472557000 -2.322627000

C 6.279023000 3.314053000 -2.398714000

H 6.086707000 2.500848000 -3.113216000

H 5.313007000 3.766428000 -2.140610000

H 6.886204000 4.074449000 -2.906328000

C 2.936606000 3.364712000 1.984533000

H 2.139671000 3.609587000 2.697188000

H 3.793525000 4.007971000 2.226124000

H 2.584072000 3.640455000 0.983702000

C 3.959521000 1.669692000 3.469610000

H 3.316968000 2.043106000 4.277743000

H 4.172352000 0.613908000 3.670177000

H 4.910050000 2.219742000 3.514979000

C 1.179487000 1.955779000 -0.812432000

H 1.784033000 1.908058000 0.094347000

H 1.485375000 2.843898000 -1.385375000

H 0.139626000 2.088919000 -0.497958000

C 2.787916000 0.748183000 -2.260961000

H 2.986735000 1.774926000 -2.602387000

H 3.561157000 0.491590000 -1.537829000

H 2.899731000 0.100960000 -3.134036000

C 0.404866000 0.857184000 -2.868996000

H 0.488724000 0.037014000 -3.591931000

H -0.636683000 0.946509000 -2.546579000

H 0.660119000 1.783312000 -3.405149000

C 2.456278000 -2.349597000 -2.410270000

H 3.261910000 -1.928513000 -1.804913000

H 2.755940000 -3.366654000 -2.704508000

H 2.360311000 -1.762645000 -3.329334000

C 1.239775000 -3.627092000 -0.695083000

H 2.145822000 -3.601637000 -0.076241000

H 0.372511000 -3.730096000 -0.035031000

H 1.289622000 -4.534013000 -1.315042000

C 0.011830000 -2.690064000 -2.633041000

H 0.310766000 -3.532919000 -3.273358000

H -0.924208000 -2.963803000 -2.137108000

H -0.186559000 -1.838533000 -3.293504000

**IM3-b-r**

E = -5177.41346359 a.u.

C -2.685967000 -4.526302000 0.384965000

C -2.166751000 -3.242544000 0.513132000

C -2.524428000 -2.159596000 -0.313622000

C -3.466273000 -2.425062000 -1.342450000

C -4.003503000 -3.723712000 -1.434304000

C -3.634423000 -4.767871000 -0.598613000

H -2.351126000 -5.318438000 1.053355000

H -1.430491000 -3.095003000 1.301270000

H -4.747942000 -3.932286000 -2.199328000

H -4.080091000 -5.752993000 -0.723622000

Br -0.660773000 1.453888000 1.953777000

C -4.003193000 -1.406892000 -2.367644000

C -4.358605000 -2.065667000 -3.709281000

H -5.249665000 -2.702225000 -3.663908000

H -4.569814000 -1.281886000 -4.448957000

H -3.523703000 -2.669437000 -4.091322000

C -2.968725000 -0.339800000 -2.701244000

H -3.424831000 0.468226000 -3.291325000

H -2.517889000 0.087998000 -1.802159000

H -2.153895000 -0.772033000 -3.298320000

C -5.283880000 -0.769348000 -1.813554000

H -5.695100000 -0.041660000 -2.528582000

H -6.051219000 -1.534654000 -1.632196000

H -5.111119000 -0.250014000 -0.865161000

C 1.881773000 -1.091597000 0.961676000

C 1.087859000 -1.764303000 1.910080000

C 1.564611000 -2.257206000 3.114593000

C 2.901490000 -2.069252000 3.434336000

C 3.694638000 -1.347138000 2.556293000

C 3.224927000 -0.826361000 1.336149000

H 0.033029000 -1.905443000 1.689660000

H 0.887941000 -2.776133000 3.791041000

H 3.318366000 -2.449524000 4.364254000

H 4.730347000 -1.141823000 2.824808000

C 4.226545000 0.051276000 0.649007000

C 4.216888000 1.441666000 0.921832000

C 5.267886000 -0.497785000 -0.125547000

C 5.159516000 2.253410000 0.292575000

C 6.201574000 0.358566000 -0.717902000

C 6.146709000 1.738003000 -0.547430000

H 5.148570000 3.329128000 0.474285000

H 6.992211000 -0.071876000 -1.331527000

P 0.825467000 -0.880411000 -0.605400000

Pd -1.531937000 -0.431172000 0.386269000

C 1.374281000 0.568480000 -1.764602000

C 1.143531000 -2.562141000 -1.526029000

C 7.079124000 2.685598000 -1.268315000

H 7.221393000 3.560165000 -0.613908000

C 3.293757000 2.052139000 1.967123000

H 2.361118000 1.468838000 2.004524000

C 5.441613000 -1.995287000 -0.309073000

H 4.477244000 -2.478232000 -0.090817000

C -3.231221000 0.215248000 1.233163000

C -3.847530000 1.283563000 0.565498000

C -3.816250000 -0.338049000 2.514457000

C -3.404848000 2.089332000 -0.474942000

H -2.428841000 2.009478000 -0.938387000

C -4.411215000 3.016778000 -0.774971000

C -4.411230000 4.079073000 -1.794544000

O -5.395245000 4.784362000 -1.957769000

C -5.463948000 2.730218000 0.093656000

O -5.134469000 1.734282000 0.904156000

C -3.156993000 4.237736000 -2.598525000

H -2.309621000 4.468283000 -1.940167000

H -2.910234000 3.302252000 -3.117181000

H -3.280586000 5.039564000 -3.329885000

C -6.802420000 3.320215000 0.240265000

H -6.722266000 4.384210000 0.489163000

H -7.349698000 3.261640000 -0.706419000

H -7.364726000 2.806302000 1.023362000

C -4.229853000 0.770236000 3.497802000

H -3.378818000 1.430744000 3.706524000

H -5.064394000 1.376416000 3.140509000

H -4.537173000 0.305433000 4.443246000

C -5.049263000 -1.176145000 2.116092000

H -4.780009000 -1.982452000 1.421198000

H -5.462657000 -1.633170000 3.024814000

H -5.833129000 -0.565346000 1.655693000

C -2.832683000 -1.242444000 3.259032000

H -3.305316000 -1.600676000 4.183210000

H -2.550845000 -2.119127000 2.670343000

H -1.923027000 -0.690601000 3.526809000

C 5.867244000 -2.389778000 -1.723445000

H 6.926504000 -2.163192000 -1.901042000

H 5.744250000 -3.470922000 -1.865676000

H 5.285472000 -1.878845000 -2.501108000

C 6.465656000 -2.541390000 0.687659000

H 6.610948000 -3.619392000 0.540522000

H 7.438477000 -2.050959000 0.544901000

H 6.159315000 -2.381282000 1.727698000

C 8.447778000 2.095774000 -1.577272000

H 9.111571000 2.869158000 -1.982114000

H 8.925853000 1.675412000 -0.684072000

H 8.383531000 1.301420000 -2.332880000

C 6.407221000 3.171224000 -2.553097000

H 6.190899000 2.320459000 -3.215282000

H 5.457059000 3.678399000 -2.343375000

H 7.052831000 3.870737000 -3.099347000

C 2.915133000 3.508528000 1.707300000

H 2.098190000 3.797998000 2.379714000

H 3.754069000 4.188168000 1.908310000

H 2.578250000 3.688299000 0.679815000

C 3.953342000 1.968214000 3.347101000

H 3.297367000 2.402512000 4.112863000

H 4.176696000 0.937027000 3.641961000

H 4.896505000 2.532574000 3.354161000

C 1.231042000 1.843932000 -0.943691000

H 1.896402000 1.837781000 -0.079237000

H 1.497814000 2.703327000 -1.577158000

H 0.213516000 1.991965000 -0.568778000

C 2.781878000 0.535479000 -2.364018000

H 3.022521000 1.550028000 -2.715937000

H 3.553382000 0.247965000 -1.650461000

H 2.851879000 -0.123388000 -3.233499000

C 0.383910000 0.657700000 -2.924680000

H 0.432004000 -0.207383000 -3.596510000

H -0.649072000 0.787469000 -2.583576000

H 0.646411000 1.543620000 -3.521965000

C 2.449730000 -2.565676000 -2.308493000

H 3.275301000 -2.151455000 -1.725920000

H 2.709580000 -3.603845000 -2.565438000

H 2.368590000 -2.010455000 -3.248725000

C 1.215295000 -3.699701000 -0.506226000

H 2.118992000 -3.646847000 0.114551000

H 0.341385000 -3.742742000 0.152273000

H 1.252712000 -4.647365000 -1.063022000

C -0.001268000 -2.860893000 -2.494912000

H 0.252367000 -3.775928000 -3.050574000

H -0.952701000 -3.033760000 -1.982048000

H -0.151887000 -2.068018000 -3.236759000

**TS_3-4­_-b-r**

E = -5177.39681276 a.u.

C -2.922991000 -4.200318000 0.392487000

C -2.474943000 -2.905874000 0.606153000

C -2.830543000 -1.799280000 -0.197637000

C -3.646816000 -2.068936000 -1.340233000

C -4.167428000 -3.368472000 -1.471510000

C -3.830975000 -4.421696000 -0.634251000

H -2.584919000 -5.010357000 1.035817000

H -1.784961000 -2.750115000 1.427920000

H -4.847448000 -3.578670000 -2.293315000

H -4.258465000 -5.407966000 -0.803057000

Br -0.535000000 1.569001000 1.976634000

C -3.997828000 -1.110781000 -2.494948000

C -4.037479000 -1.844600000 -3.848330000

H -4.898409000 -2.512295000 -3.962149000

H -4.111605000 -1.095740000 -4.648437000

H -3.123444000 -2.428348000 -4.021467000

C -2.944234000 -0.019194000 -2.676963000

H -3.351734000 0.812230000 -3.269350000

H -2.563898000 0.373250000 -1.733468000

H -2.080437000 -0.422237000 -3.220242000

C -5.403038000 -0.538037000 -2.268931000

H -5.640782000 0.212771000 -3.035277000

H -6.153045000 -1.336906000 -2.337988000

H -5.519487000 -0.080574000 -1.285719000

C 1.957146000 -1.067434000 0.949970000

C 1.153727000 -1.721001000 1.905025000

C 1.641364000 -2.279438000 3.076200000

C 2.998975000 -2.188830000 3.349508000

C 3.805762000 -1.487996000 2.466619000

C 3.325277000 -0.893954000 1.284695000

H 0.085192000 -1.799659000 1.712588000

H 0.958876000 -2.777489000 3.762418000

H 3.423588000 -2.626779000 4.250156000

H 4.861021000 -1.356862000 2.704103000

C 4.343527000 -0.034852000 0.596903000

C 4.402642000 1.342289000 0.925753000

C 5.342324000 -0.593082000 -0.225071000

C 5.362740000 2.139197000 0.303533000

C 6.295458000 0.246541000 -0.810471000

C 6.305624000 1.618603000 -0.582938000

H 5.401583000 3.205801000 0.528936000

H 7.049026000 -0.194687000 -1.462424000

P 0.870993000 -0.750495000 -0.575275000

Pd -1.413006000 -0.246297000 0.418467000

C 1.495994000 0.658892000 -1.736957000

C 1.007088000 -2.452458000 -1.498933000

C 7.265554000 2.552395000 -1.285680000

H 7.410229000 3.422369000 -0.625832000

C 3.530920000 1.946519000 2.019336000

H 2.574589000 1.401890000 2.052229000

C 5.459628000 -2.087113000 -0.464462000

H 4.503928000 -2.552420000 -0.181670000

C -3.289819000 -0.134344000 1.075376000

C -4.043222000 0.902398000 0.431963000

C -3.828894000 -0.604920000 2.437244000

C -3.630709000 1.984378000 -0.309253000

H -2.605581000 2.186590000 -0.596386000

C -4.760881000 2.795371000 -0.577359000

C -4.795339000 4.041423000 -1.362014000

O -5.825822000 4.681446000 -1.514587000

C -5.834283000 2.152957000 0.014299000

O -5.418212000 1.044228000 0.635538000

C -3.495228000 4.471953000 -1.974392000

H -2.741343000 4.644467000 -1.195857000

H -3.100574000 3.685014000 -2.630813000

H -3.641340000 5.389558000 -2.548514000

C -7.280246000 2.425030000 0.041987000

H -7.485424000 3.373399000 -0.455615000

H -7.824734000 1.621791000 -0.471367000

H -7.648428000 2.462319000 1.073872000

C -4.112712000 0.678919000 3.256388000

H -3.197019000 1.277073000 3.349570000

H -4.899429000 1.304548000 2.828137000

H -4.435623000 0.385290000 4.262801000

C -5.116662000 -1.431386000 2.303513000

H -4.927870000 -2.367959000 1.763597000

H -5.474237000 -1.691816000 3.308934000

H -5.917078000 -0.891124000 1.790078000

C -2.832585000 -1.402813000 3.288852000

H -3.163249000 -1.365571000 4.334289000

H -2.789098000 -2.460403000 3.020598000

H -1.825236000 -0.971391000 3.240043000

C 5.760098000 -2.445642000 -1.919537000

H 6.802961000 -2.222783000 -2.179951000

H 5.611376000 -3.520695000 -2.082789000

H 5.120205000 -1.904762000 -2.627624000

C 6.541792000 -2.686074000 0.435391000

H 6.632806000 -3.766515000 0.264995000

H 7.518026000 -2.229837000 0.221317000

H 6.327554000 -2.529197000 1.498845000

C 8.630466000 1.939745000 -1.568390000

H 9.314274000 2.701239000 -1.962635000

H 9.084942000 1.513950000 -0.665578000

H 8.567871000 1.144721000 -2.323281000

C 6.630641000 3.054623000 -2.582369000

H 6.439595000 2.214557000 -3.265338000

H 5.672798000 3.555911000 -2.394020000

H 7.290811000 3.764777000 -3.096601000

C 3.207371000 3.427234000 1.834051000

H 2.440619000 3.724611000 2.559565000

H 4.085147000 4.061472000 2.017083000

H 2.825080000 3.662200000 0.833939000

C 4.224091000 1.774564000 3.373933000

H 3.604051000 2.188961000 4.179457000

H 4.425358000 0.723950000 3.610376000

H 5.183460000 2.310797000 3.379775000

C 1.571651000 1.915941000 -0.878211000

H 2.359153000 1.832108000 -0.130067000

H 1.815815000 2.769737000 -1.528505000

H 0.634404000 2.130223000 -0.353761000

C 2.839131000 0.472212000 -2.446366000

H 3.201762000 1.461434000 -2.764849000

H 3.610140000 0.034427000 -1.808738000

H 2.749440000 -0.136388000 -3.351127000

C 0.425186000 0.906750000 -2.799671000

H 0.310089000 0.071712000 -3.500192000

H -0.552723000 1.137182000 -2.358157000

H 0.733626000 1.780968000 -3.392133000

C 2.307562000 -2.618029000 -2.273352000

H 3.174255000 -2.339743000 -1.668829000

H 2.426556000 -3.674347000 -2.558692000

H 2.322167000 -2.032097000 -3.197436000

C 0.926335000 -3.598569000 -0.489352000

H 1.817188000 -3.661501000 0.147352000

H 0.038984000 -3.547053000 0.151733000

H 0.862058000 -4.538812000 -1.056025000

C -0.168325000 -2.597438000 -2.463898000

H -0.079114000 -3.565501000 -2.978989000

H -1.133724000 -2.578806000 -1.945610000

H -0.176890000 -1.821442000 -3.237013000

**IM4-b-r**

E = -5177.44123632 a.u.

C 2.464778000 4.036374000 0.710665000

C 2.604118000 2.695231000 1.035537000

C 3.171048000 1.699440000 0.186100000

C 3.445600000 2.121672000 -1.195955000

C 3.343682000 3.492680000 -1.441593000

C 2.897901000 4.448740000 -0.530009000

H 2.052032000 4.731629000 1.437477000

H 2.351592000 2.416185000 2.045226000

H 3.607692000 3.867609000 -2.422524000

H 2.867166000 5.495840000 -0.822487000

Br 0.982943000 -2.102545000 0.673941000

C 3.848567000 1.280717000 -2.442426000

C 3.602592000 2.061595000 -3.750019000

H 4.324334000 2.869727000 -3.918738000

H 3.722738000 1.358994000 -4.583967000

H 2.585892000 2.473368000 -3.807260000

C 3.007286000 0.009834000 -2.622307000

H 3.524806000 -0.676839000 -3.307310000

H 2.798310000 -0.532685000 -1.699303000

H 2.047261000 0.267881000 -3.081799000

C 5.352868000 0.956723000 -2.454753000

H 5.656445000 0.721010000 -3.483133000

H 5.949528000 1.816477000 -2.122080000

H 5.614900000 0.094483000 -1.845362000

C -2.111074000 0.547012000 1.270746000

C -1.256771000 0.820416000 2.355982000

C -1.675461000 0.876227000 3.677480000

C -3.012944000 0.650526000 3.967339000

C -3.870261000 0.320370000 2.927944000

C -3.461584000 0.228403000 1.585996000

H -0.205142000 1.007807000 2.147445000

H -0.955692000 1.094536000 4.464423000

H -3.384561000 0.700394000 4.988279000

H -4.909614000 0.084353000 3.154786000

C -4.514504000 -0.343053000 0.682160000

C -4.567798000 -1.750489000 0.518425000

C -5.548449000 0.449100000 0.145471000

C -5.540425000 -2.294816000 -0.317369000

C -6.498365000 -0.143474000 -0.694399000

C -6.488470000 -1.506328000 -0.969111000

H -5.568015000 -3.373481000 -0.476975000

H -7.272599000 0.484343000 -1.134629000

P -1.106858000 0.844330000 -0.311787000

Pd 1.328174000 0.352837000 0.221927000

C -1.738354000 -0.089708000 -1.862776000

C -1.310712000 2.747913000 -0.572682000

C -7.419651000 -2.139764000 -1.976720000

H -7.571782000 -3.185793000 -1.667070000

C -3.647198000 -2.686316000 1.289811000

H -2.709150000 -2.151276000 1.498950000

C -5.739596000 1.906633000 0.519938000

H -4.807648000 2.268216000 0.975706000

C 3.365310000 0.375956000 0.840801000

C 4.380966000 -0.524463000 0.236797000

C 3.493644000 0.238344000 2.434630000

C 4.413695000 -1.786752000 -0.253262000

H 3.539537000 -2.399217000 -0.437543000

C 5.794312000 -2.147333000 -0.416690000

C 6.332808000 -3.411994000 -0.934547000

O 7.535796000 -3.619284000 -1.038440000

C 6.525333000 -1.060152000 0.003489000

O 5.684602000 -0.082632000 0.401885000

C 5.323690000 -4.451839000 -1.331098000

H 4.701959000 -4.729408000 -0.470609000

H 4.644959000 -4.059984000 -2.098853000

H 5.834653000 -5.339328000 -1.711652000

C 7.964967000 -0.741608000 0.046028000

H 8.542629000 -1.619188000 -0.247973000

H 8.197243000 0.084398000 -0.639991000

H 8.268921000 -0.425230000 1.051334000

C 3.977472000 -1.180509000 2.791358000

H 3.298895000 -1.942392000 2.387472000

H 4.995312000 -1.391073000 2.445571000

H 3.987887000 -1.277966000 3.884356000

C 4.548259000 1.214891000 2.984301000

H 4.243696000 2.264920000 2.918884000

H 4.723249000 0.991875000 4.045585000

H 5.504555000 1.109093000 2.461145000

C 2.196502000 0.383628000 3.255628000

H 2.444254000 0.212656000 4.312130000

H 1.706889000 1.361022000 3.218209000

H 1.463483000 -0.374628000 2.958364000

C -6.081733000 2.802452000 -0.668590000

H -7.111643000 2.637379000 -1.010511000

H -6.005029000 3.859397000 -0.383278000

H -5.417132000 2.632924000 -1.524293000

C -6.833037000 2.032371000 1.582879000

H -6.975656000 3.083295000 1.865816000

H -7.791686000 1.656083000 1.200504000

H -6.591042000 1.467730000 2.491145000

C -8.781383000 -1.467479000 -2.075331000

H -9.442697000 -2.042811000 -2.734233000

H -9.269903000 -1.383019000 -1.096894000

H -8.701830000 -0.458095000 -2.500538000

C -6.733403000 -2.154236000 -3.344052000

H -6.534883000 -1.126670000 -3.681123000

H -5.771975000 -2.682606000 -3.305905000

H -7.362314000 -2.642578000 -4.099373000

C -3.295370000 -3.978748000 0.557310000

H -2.501157000 -4.505549000 1.100663000

H -4.153682000 -4.661938000 0.508781000

H -2.943568000 -3.808517000 -0.466625000

C -4.292490000 -3.048979000 2.629754000

H -3.635144000 -3.709014000 3.210398000

H -4.507802000 -2.165253000 3.240596000

H -5.239285000 -3.581171000 2.462267000

C -1.964573000 -1.547746000 -1.480422000

H -2.902049000 -1.666483000 -0.938877000

H -2.044875000 -2.141574000 -2.404004000

H -1.145148000 -1.958730000 -0.878947000

C -3.000070000 0.444890000 -2.533642000

H -3.333146000 -0.296077000 -3.276928000

H -3.828190000 0.595314000 -1.829977000

H -2.818735000 1.379580000 -3.075802000

C -0.586748000 -0.072213000 -2.867914000

H -0.269766000 0.934910000 -3.162158000

H 0.279671000 -0.610550000 -2.466694000

H -0.914505000 -0.592745000 -3.779737000

C -2.720547000 3.211927000 -0.907070000

H -3.444992000 2.817943000 -0.190222000

H -2.758696000 4.309797000 -0.841240000

H -3.038826000 2.938275000 -1.915706000

C -0.917475000 3.446211000 0.729857000

H -1.690227000 3.329243000 1.499516000

H 0.034406000 3.092438000 1.138363000

H -0.813188000 4.522206000 0.528089000

C -0.358962000 3.174054000 -1.689401000

H -0.298330000 4.272262000 -1.709584000

H 0.658299000 2.782943000 -1.548178000

H -0.719134000 2.854286000 -2.674493000

**IM5-b-r**

E = -2834.52414053 a.u.

C -2.343464000 -4.061406000 0.865307000

C -2.542173000 -2.727667000 1.175423000

C -3.125280000 -1.757486000 0.303365000

C -3.381822000 -2.210748000 -1.071573000

C -3.211847000 -3.579478000 -1.307260000

C -2.729286000 -4.502014000 -0.384979000

H -1.919121000 -4.735085000 1.605372000

H -2.311569000 -2.429464000 2.185362000

H -3.447065000 -3.971731000 -2.288808000

H -2.639402000 -5.548274000 -0.668445000

C -3.838214000 -1.400595000 -2.317842000

C -3.623310000 -2.188669000 -3.625813000

H -4.324745000 -3.021773000 -3.754643000

H -3.799312000 -1.498250000 -4.460155000

H -2.598348000 -2.569258000 -3.726144000

C -3.025225000 -0.115451000 -2.532710000

H -3.581075000 0.567583000 -3.192224000

H -2.774572000 0.437222000 -1.626693000

H -2.081184000 -0.358158000 -3.035835000

C -5.351619000 -1.130550000 -2.278317000

H -5.681338000 -0.796090000 -3.270941000

H -5.901427000 -2.051500000 -2.041833000

H -5.648996000 -0.363907000 -1.566795000

C 2.055369000 -0.742411000 1.166530000

C 1.197972000 -1.195296000 2.187473000

C 1.605976000 -1.454604000 3.487851000

C 2.937064000 -1.259662000 3.822658000

C 3.799374000 -0.766321000 2.854318000

C 3.402382000 -0.473287000 1.538122000

H 0.151639000 -1.364667000 1.939085000

H 0.883841000 -1.806971000 4.222048000

H 3.301750000 -1.462844000 4.826971000

H 4.835473000 -0.565848000 3.124222000

C 4.471019000 0.199644000 0.728170000

C 4.563416000 1.613273000 0.761200000

C 5.480684000 -0.541348000 0.080428000

C 5.553878000 2.240028000 0.005610000

C 6.454427000 0.134704000 -0.662057000

C 6.485520000 1.522783000 -0.743726000

H 5.611823000 3.329276000 -0.004226000

H 7.214110000 -0.445830000 -1.184632000

P 1.058556000 -0.769972000 -0.449105000

Pd -1.350826000 -0.348342000 0.211879000

C 1.654227000 0.477914000 -1.771446000

C 1.296797000 -2.581047000 -1.065237000

C 7.446302000 2.260905000 -1.647199000

H 7.590840000 3.263810000 -1.215690000

C 3.679689000 2.460175000 1.667823000

H 2.718495000 1.939462000 1.798225000

C 5.617178000 -2.044526000 0.242403000

H 4.647910000 -2.442251000 0.576826000

C -3.343270000 -0.418229000 0.920634000

C -4.389779000 0.447864000 0.322008000

C -3.398587000 -0.207007000 2.506658000

C -4.431059000 1.666849000 -0.265212000

H -3.561132000 2.225511000 -0.591922000

C -5.811591000 2.050700000 -0.353838000

C -6.352501000 3.283196000 -0.939243000

O -7.550613000 3.539109000 -0.943651000

C -6.533760000 1.022407000 0.205256000

O -5.687594000 0.054963000 0.617893000

C -5.348694000 4.223859000 -1.544974000

H -4.612874000 4.540343000 -0.794594000

H -4.787848000 3.722087000 -2.344381000

H -5.857964000 5.101525000 -1.950132000

C -7.973085000 0.758229000 0.389966000

H -8.546754000 1.629141000 0.069033000

H -8.287448000 -0.114015000 -0.198538000

H -8.199789000 0.540118000 1.440859000

C -3.855144000 1.233134000 2.814813000

H -3.207002000 1.973918000 2.329684000

H -4.892678000 1.433636000 2.527332000

H -3.793562000 1.392092000 3.898762000

C -4.429492000 -1.147641000 3.154339000

H -4.130236000 -2.201005000 3.126971000

H -4.554596000 -0.872352000 4.210536000

H -5.408054000 -1.064503000 2.669894000

C -2.062091000 -0.319651000 3.265183000

H -2.259236000 -0.114898000 4.326473000

H -1.572513000 -1.296102000 3.234756000

H -1.351126000 0.431754000 2.906026000

C 6.032873000 -2.768425000 -1.037566000

H 7.094831000 -2.602901000 -1.260655000

H 5.894686000 -3.850795000 -0.921614000

H 5.458559000 -2.446260000 -1.914872000

C 6.632938000 -2.358324000 1.343564000

H 6.731707000 -3.443022000 1.479456000

H 7.622801000 -1.962381000 1.078917000

H 6.344646000 -1.922965000 2.307425000

C 8.810454000 1.598497000 -1.779763000

H 9.486628000 2.236632000 -2.361580000

H 9.275041000 1.415745000 -0.803206000

H 8.744312000 0.638137000 -2.307967000

C 6.801566000 2.433768000 -3.023284000

H 6.613140000 1.452499000 -3.482558000

H 5.840805000 2.959145000 -2.951721000

H 7.452632000 3.003986000 -3.698208000

C 3.382896000 3.864543000 1.145614000

H 2.608300000 4.329728000 1.767969000

H 4.267703000 4.512341000 1.203137000

H 3.032440000 3.874050000 0.107442000

C 4.337395000 2.592244000 3.044616000

H 3.719691000 3.211327000 3.707857000

H 4.487913000 1.622601000 3.531497000

H 5.318771000 3.077511000 2.950192000

C 1.823797000 1.821422000 -1.062563000

H 2.842825000 1.919508000 -0.690937000

H 1.651260000 2.636915000 -1.780431000

H 1.136565000 1.957537000 -0.221995000

C 2.941870000 0.173654000 -2.533336000

H 3.233281000 1.085901000 -3.077443000

H 3.778615000 -0.094780000 -1.876983000

H 2.809753000 -0.613208000 -3.283687000

C 0.507794000 0.616736000 -2.776181000

H 0.265026000 -0.324279000 -3.283017000

H -0.391734000 1.020079000 -2.295751000

H 0.822312000 1.331308000 -3.551557000

C 2.680164000 -2.876080000 -1.627110000

H 3.461402000 -2.473152000 -0.980168000

H 2.825211000 -3.965538000 -1.684333000

H 2.823556000 -2.479216000 -2.635740000

C 1.076697000 -3.518953000 0.121791000

H 1.916971000 -3.488917000 0.827142000

H 0.156878000 -3.305490000 0.677359000

H 1.000787000 -4.547785000 -0.259654000

C 0.238675000 -2.863805000 -2.129179000

H 0.255030000 -3.935650000 -2.377950000

H -0.773295000 -2.614817000 -1.784573000

H 0.432258000 -2.315289000 -3.059343000

C -1.101516000 2.642460000 0.214201000

C -0.744457000 3.964174000 0.866086000

H 0.042095000 4.452703000 0.277058000

H -1.617400000 4.627525000 0.848957000

H -0.399937000 3.845665000 1.897287000

O -0.898854000 1.611831000 0.953266000

O -1.532699000 2.639262000 -0.946687000

**TS_5-6_-b-r-1**

E = -2834.46291221 a.u.

C 2.654801000 3.690770000 2.080319000

C 2.564218000 2.307714000 2.059457000

C 2.965723000 1.516456000 0.960014000

C 3.367578000 2.214557000 -0.234654000

C 3.532591000 3.599988000 -0.142184000

C 3.193639000 4.343893000 0.982970000

H 2.339824000 4.240626000 2.964588000

H 2.188097000 1.825238000 2.950629000

H 3.897801000 4.145699000 -1.007061000

H 3.324229000 5.423722000 0.979117000

C 3.581171000 1.586210000 -1.632021000

C 3.248141000 2.611104000 -2.738126000

H 3.988889000 3.416168000 -2.812208000

H 3.258381000 2.091314000 -3.704596000

H 2.258068000 3.065222000 -2.608153000

C 2.693234000 0.343356000 -1.959128000

H 3.353203000 -0.377556000 -2.470341000

H 1.945631000 0.617466000 -2.714112000

C 5.075012000 1.268952000 -1.850045000

H 5.256951000 1.094197000 -2.918959000

H 5.700969000 2.117766000 -1.542802000

H 5.416298000 0.380084000 -1.320908000

C -1.912779000 1.223297000 0.632092000

C -1.021989000 1.979818000 1.417875000

C -1.380856000 2.617984000 2.596304000

C -2.683244000 2.494250000 3.057702000

C -3.554152000 1.670664000 2.359486000

C -3.204154000 1.003948000 1.172053000

H 0.010631000 2.080443000 1.089035000

H -0.638165000 3.198102000 3.141709000

H -3.008444000 2.989350000 3.969710000

H -4.548599000 1.487651000 2.765430000

C -4.238262000 0.013756000 0.735241000

C -4.103751000 -1.324341000 1.173557000

C -5.414941000 0.423849000 0.077851000

C -5.141818000 -2.221139000 0.918500000

C -6.410703000 -0.520632000 -0.181405000

C -6.296293000 -1.845456000 0.233895000

H -5.049262000 -3.255723000 1.249519000

H -7.312885000 -0.205483000 -0.707899000

P -1.034122000 0.843263000 -1.000899000

Pd 1.250457000 0.109840000 -0.147220000

C -1.915902000 -0.369129000 -2.207892000

C -1.091357000 2.627020000 -1.781182000

C -7.395488000 -2.847533000 -0.038622000

C -2.850859000 -1.803948000 1.893625000

H -2.005455000 -1.222060000 1.497022000

C -5.659967000 1.873444000 -0.302777000

H -4.692471000 2.393437000 -0.280542000

C 2.955385000 0.019015000 1.063007000

C 4.224955000 -0.590928000 0.588865000

C 2.500508000 -0.711978000 2.396240000

C 4.582585000 -1.718293000 -0.075878000

H 3.897338000 -2.397360000 -0.568389000

C 6.014715000 -1.808078000 -0.024330000

C 6.865630000 -2.842429000 -0.625524000

O 8.084343000 -2.846297000 -0.499653000

C 6.441125000 -0.707648000 0.683565000

O 5.373333000 0.023682000 1.061414000

C 6.158350000 -3.911237000 -1.409598000

H 5.462667000 -4.461262000 -0.763451000

H 5.559870000 -3.468266000 -2.215589000

H 6.887512000 -4.606181000 -1.832992000

C 7.766895000 -0.177301000 1.052018000

H 8.541693000 -0.845411000 0.672741000

H 7.913239000 0.824376000 0.626871000

H 7.871701000 -0.088613000 2.140395000

C 2.650635000 -2.237018000 2.262390000

H 2.150593000 -2.618268000 1.367873000

H 3.700138000 -2.550367000 2.252095000

H 2.181658000 -2.707299000 3.137016000

C 3.406763000 -0.335352000 3.584774000

H 3.316460000 0.698197000 3.928388000

H 3.151632000 -0.982918000 4.434882000

H 4.462011000 -0.514137000 3.346638000

C 1.022776000 -0.475324000 2.759827000

H 0.809832000 -0.995048000 3.704567000

H 0.744403000 0.571596000 2.902472000

H 0.346390000 -0.907865000 2.004657000

C 1.124383000 -2.819576000 -1.199236000

C 0.635480000 -4.131976000 -1.759990000

H 0.100854000 -3.945018000 -2.700019000

H 1.479825000 -4.788278000 -1.996623000

H -0.039362000 -4.634081000 -1.061192000

O 0.634677000 -2.378943000 -0.139899000

O 2.018100000 -2.218265000 -1.892020000

H 2.242516000 -0.902877000 -1.499723000

H -7.056708000 -3.816996000 0.358706000

C -2.205681000 -1.682623000 -1.483844000

H -3.099714000 -1.606336000 -0.863799000

H -2.407496000 -2.456402000 -2.240192000

H -1.364243000 -2.024458000 -0.871383000

C -0.893576000 -0.688772000 -3.306836000

H 0.031856000 -1.108597000 -2.893003000

H -1.335703000 -1.446778000 -3.969709000

H -0.629317000 0.167211000 -3.932269000

C -3.215843000 0.123388000 -2.829936000

H -3.625739000 -0.679586000 -3.461157000

H -3.964296000 0.344061000 -2.061339000

H -3.095194000 1.004763000 -3.468642000

C -2.466614000 3.278965000 -1.667727000

H -3.251653000 2.715432000 -2.183231000

H -2.765602000 3.417328000 -0.620531000

H -2.421698000 4.277409000 -2.127769000

C -0.671210000 2.571452000 -3.250002000

H -1.399791000 2.062334000 -3.887218000

H -0.585584000 3.601456000 -3.624594000

H 0.308308000 2.095196000 -3.385682000

C -0.081946000 3.528891000 -1.063952000

H 0.055700000 4.442473000 -1.660437000

H -0.425146000 3.847301000 -0.074201000

H 0.896665000 3.047125000 -0.950782000

C -2.922545000 -1.539038000 3.398156000

H -3.781393000 -2.062299000 3.841728000

H -2.013891000 -1.908182000 3.892428000

H -3.020354000 -0.471963000 3.629324000

C -2.530641000 -3.277848000 1.663416000

H -3.222044000 -3.935853000 2.206603000

H -2.563093000 -3.554464000 0.602412000

H -1.520669000 -3.494404000 2.035265000

C -6.576223000 2.545516000 0.721361000

H -7.548653000 2.035932000 0.761496000

H -6.150708000 2.529785000 1.731740000

H -6.759810000 3.593048000 0.449762000

C -6.252657000 2.053751000 -1.698402000

H -7.293404000 1.706970000 -1.746095000

H -6.257596000 3.118098000 -1.965715000

H -5.688290000 1.517074000 -2.470771000

C -8.685689000 -2.468498000 0.683988000

H -9.458429000 -3.232528000 0.530641000

H -8.525944000 -2.361159000 1.763766000

H -9.083395000 -1.516000000 0.308367000

C -7.645463000 -3.012018000 -1.534970000

H -8.015562000 -2.077803000 -1.978136000

H -6.729740000 -3.294804000 -2.068479000

H -8.399298000 -3.787290000 -1.721743000

**IM6-b-r-1**

E = -2834.52327991 a.u.

C -4.016783000 -3.894260000 1.951776000

C -3.478985000 -2.614080000 2.018430000

C -3.334662000 -1.778379000 0.895677000

C -3.721478000 -2.319082000 -0.369178000

C -4.315698000 -3.585431000 -0.396543000

C -4.467190000 -4.379841000 0.734912000

H -4.103111000 -4.489355000 2.858974000

H -3.188743000 -2.251636000 2.996715000

H -4.663872000 -3.985716000 -1.345774000

H -4.919644000 -5.366044000 0.653744000

C -3.550409000 -1.603132000 -1.724538000

C -3.114145000 -2.595528000 -2.815747000

H -3.838463000 -3.397607000 -2.992116000

H -3.004479000 -2.057014000 -3.766386000

H -2.146903000 -3.057211000 -2.580323000

C -2.520505000 -0.459047000 -1.736811000

H -3.011065000 0.515839000 -1.659798000

H -1.939102000 -0.484479000 -2.668985000

C -4.915699000 -1.035075000 -2.163785000

H -4.820415000 -0.541378000 -3.140534000

H -5.660250000 -1.836336000 -2.265892000

H -5.308486000 -0.299078000 -1.452187000

C 1.919159000 -1.604264000 0.456930000

C 1.102342000 -2.597180000 1.039895000

C 1.430961000 -3.283318000 2.199112000

C 2.607873000 -2.963006000 2.860334000

C 3.397904000 -1.944361000 2.350189000

C 3.087770000 -1.240056000 1.172702000

H 0.144799000 -2.827385000 0.574906000

H 0.754863000 -4.042791000 2.586232000

H 2.899547000 -3.479257000 3.771997000

H 4.303625000 -1.657755000 2.884129000

C 4.050554000 -0.127211000 0.891925000

C 3.816902000 1.145813000 1.463144000

C 5.268094000 -0.382609000 0.231001000

C 4.767017000 2.149056000 1.273663000

C 6.187455000 0.656847000 0.065787000

C 5.948363000 1.933553000 0.564644000

H 4.590667000 3.141617000 1.689692000

H 7.119207000 0.455697000 -0.463127000

P 1.089220000 -1.091148000 -1.170167000

Pd -1.133905000 -0.536558000 -0.222714000

C 1.811665000 0.441641000 -2.070014000

C 1.395850000 -2.672893000 -2.242151000

C 6.893411000 3.085369000 0.307969000

H 6.716538000 3.832985000 1.097053000

C 2.580593000 1.439761000 2.301863000

H 1.772208000 0.772146000 1.960457000

C 5.639690000 -1.773543000 -0.246714000

H 4.721537000 -2.375266000 -0.265328000

C -2.851003000 -0.345990000 1.020308000

C -3.955179000 0.583901000 0.669156000

C -2.196160000 0.153273000 2.374657000

C -4.040909000 1.855590000 0.197253000

H -3.214034000 2.467702000 -0.136124000

C -5.422714000 2.240076000 0.212759000

C -5.982105000 3.515574000 -0.243590000

O -7.175476000 3.782741000 -0.165105000

C -6.110837000 1.154372000 0.699387000

O -5.241103000 0.161984000 0.979564000

C -4.996233000 4.487910000 -0.833125000

H -4.215912000 4.739867000 -0.103189000

H -4.490565000 4.034831000 -1.696032000

H -5.510926000 5.399103000 -1.147274000

C -7.540477000 0.869906000 0.926425000

H -8.132581000 1.742021000 0.642301000

H -7.864685000 0.004190000 0.334373000

H -7.736109000 0.634183000 1.980085000

C -1.600057000 1.558228000 2.191956000

H -0.921045000 1.614906000 1.330185000

H -2.369265000 2.324799000 2.053472000

H -1.030107000 1.827396000 3.094237000

C -3.219480000 0.293693000 3.515428000

H -3.747344000 -0.629492000 3.772254000

H -2.706736000 0.641767000 4.423302000

H -3.979592000 1.045349000 3.264548000

C -1.015390000 -0.722528000 2.811578000

H -0.638815000 -0.377026000 3.785232000

H -1.242051000 -1.787885000 2.905806000

H -0.177289000 -0.628187000 2.103286000

C 6.247660000 -1.808955000 -1.646134000

H 7.253660000 -1.369976000 -1.664202000

H 6.345973000 -2.848341000 -1.985040000

H 5.635797000 -1.272798000 -2.381750000

C 6.600674000 -2.428306000 0.746297000

H 6.853057000 -3.448197000 0.428671000

H 7.537065000 -1.857488000 0.812939000

H 6.172400000 -2.485087000 1.754358000

C 8.364460000 2.692916000 0.347144000

H 8.998418000 3.584585000 0.270968000

H 8.625500000 2.170984000 1.275792000

H 8.629766000 2.037918000 -0.493321000

C 6.550682000 3.733253000 -1.033588000

H 6.700544000 3.016673000 -1.853491000

H 5.504042000 4.061703000 -1.064928000

H 7.188665000 4.604816000 -1.227843000

C 2.073794000 2.875540000 2.186742000

H 1.118994000 2.972879000 2.719344000

H 2.767567000 3.588660000 2.650962000

H 1.907338000 3.191312000 1.149826000

C 2.847522000 1.131456000 3.776155000

H 1.955619000 1.347650000 4.379591000

H 3.120969000 0.082910000 3.940607000

H 3.667507000 1.757795000 4.154201000

C 1.713910000 1.589421000 -1.070483000

H 2.475165000 1.501255000 -0.296292000

H 1.882007000 2.544663000 -1.591246000

H 0.728630000 1.644029000 -0.586876000

C 3.238642000 0.347156000 -2.591309000

H 3.517647000 1.318427000 -3.028083000

H 3.956286000 0.130846000 -1.793029000

H 3.345217000 -0.404358000 -3.382485000

C 0.881531000 0.781019000 -3.243696000

H 1.027898000 0.134434000 -4.111605000

H -0.178073000 0.756353000 -2.968573000

H 1.108342000 1.807718000 -3.565671000

C 2.856883000 -3.110077000 -2.229074000

H 3.179263000 -3.402218000 -1.221842000

H 2.969393000 -3.992562000 -2.876582000

H 3.536864000 -2.337484000 -2.604215000

C 0.555872000 -3.843559000 -1.722751000

H 0.963748000 -4.277471000 -0.804467000

H -0.492135000 -3.574469000 -1.537700000

H 0.567157000 -4.637655000 -2.482355000

C 0.958179000 -2.393310000 -3.679596000

H 0.973851000 -3.338901000 -4.239094000

H -0.062459000 -1.991062000 -3.742408000

H 1.639911000 -1.709350000 -4.193630000

C -1.064901000 3.770506000 -1.290352000

C -1.092415000 4.927634000 -2.232727000

H -0.294365000 4.801095000 -2.974415000

H -2.038800000 4.965736000 -2.780730000

H -0.933025000 5.860360000 -1.689537000

O -0.708957000 3.811951000 -0.132529000

O -1.469503000 2.637671000 -1.888249000

H -1.347175000 1.884566000 -1.267603000

**TS_5-6_-b-r-2**

E = -2834.46662690 a.u.

C 2.711096000 3.750013000 1.933868000

C 2.628207000 2.371937000 1.876121000

C 3.131642000 1.592977000 0.804953000

C 3.569210000 2.315555000 -0.366342000

C 3.726583000 3.702578000 -0.226051000

C 3.336491000 4.423627000 0.893103000

H 2.318651000 4.281537000 2.798044000

H 2.162261000 1.865126000 2.708854000

H 4.143003000 4.265790000 -1.055466000

H 3.482300000 5.500905000 0.924255000

C 3.796860000 1.773429000 -1.803890000

C 3.418122000 2.849166000 -2.844333000

H 4.135633000 3.674994000 -2.892407000

H 3.412843000 2.387259000 -3.839419000

H 2.420922000 3.271676000 -2.665825000

C 2.909074000 0.569902000 -2.143129000

H 2.916869000 0.377560000 -3.225096000

H 3.210033000 -0.359767000 -1.665396000

H 1.847074000 0.809819000 -1.910883000

C 5.272866000 1.435061000 -2.060803000

H 5.419308000 1.209814000 -3.125945000

H 5.912447000 2.292036000 -1.811689000

H 5.621535000 0.569297000 -1.493492000

C -2.006182000 1.148145000 0.794234000

C -1.136270000 1.914228000 1.594802000

C -1.529110000 2.588142000 2.741511000

C -2.850419000 2.495996000 3.154133000

C -3.717634000 1.696869000 2.424108000

C -3.339545000 1.000933000 1.261606000

H -0.092639000 1.987110000 1.299414000

H -0.800596000 3.171627000 3.301769000

H -3.200670000 3.014429000 4.043671000

H -4.745478000 1.581280000 2.767221000

C -4.427819000 0.114273000 0.732269000

C -4.507773000 -1.221271000 1.190326000

C -5.465466000 0.636248000 -0.067083000

C -5.559260000 -2.024804000 0.746361000

C -6.492630000 -0.210444000 -0.490749000

C -6.546153000 -1.549016000 -0.114215000

H -5.615167000 -3.063479000 1.073411000

H -7.275249000 0.194198000 -1.133258000

P -1.030441000 0.663680000 -0.759357000

Pd 1.300374000 0.035552000 -0.004053000

C -1.829294000 -0.655569000 -1.891198000

C -1.005943000 2.370738000 -1.693685000

C -7.601250000 -2.484898000 -0.658355000

H -7.593904000 -3.386792000 -0.026854000

C -3.505484000 -1.793457000 2.180590000

H -2.551093000 -1.261041000 2.044581000

C -5.541022000 2.104652000 -0.436338000

H -4.558928000 2.551013000 -0.237844000

C 3.171331000 0.104976000 0.964734000

C 4.401588000 -0.517745000 0.416377000

C 2.722421000 -0.625096000 2.299078000

C 4.720082000 -1.622138000 -0.302970000

H 3.995471000 -2.269680000 -0.784467000

C 6.149007000 -1.762385000 -0.254293000

C 6.968419000 -2.801727000 -0.889859000

O 8.182954000 -2.864118000 -0.739538000

C 6.611141000 -0.712261000 0.505670000

O 5.568193000 0.037950000 0.917074000

C 6.235783000 -3.798932000 -1.741846000

H 5.491882000 -4.341497000 -1.145207000

H 5.687857000 -3.290255000 -2.544677000

H 6.943825000 -4.509828000 -2.174652000

C 7.954031000 -0.244029000 0.896699000

H 8.705961000 -0.920803000 0.487387000

H 8.134590000 0.770395000 0.518230000

H 8.060050000 -0.208976000 1.988009000

C 3.209866000 -2.082833000 2.295676000

H 2.960600000 -2.615228000 1.374428000

H 4.294358000 -2.146911000 2.456253000

H 2.722797000 -2.605851000 3.128895000

C 3.246930000 -0.052614000 3.627667000

H 2.680656000 0.794250000 4.022941000

H 3.201456000 -0.834053000 4.397566000

H 4.298445000 0.255386000 3.541925000

C 1.199033000 -0.660331000 2.154256000

H 0.755627000 -1.427692000 2.809898000

H 0.694127000 0.275474000 2.415730000

H 0.820329000 -1.574521000 1.198531000

C -5.879804000 2.351345000 -1.904836000

H -6.927536000 2.109265000 -2.126322000

H -5.736337000 3.411438000 -2.150993000

H -5.252059000 1.759853000 -2.583232000

C -6.560618000 2.820228000 0.450772000

H -6.603645000 3.889441000 0.206112000

H -7.565710000 2.402237000 0.302791000

H -6.315465000 2.727360000 1.515617000

C -9.006569000 -1.896153000 -0.625941000

H -9.741323000 -2.640898000 -0.955784000

H -9.287713000 -1.566673000 0.381581000

H -9.097298000 -1.032857000 -1.298375000

C -7.228514000 -2.906154000 -2.079526000

H -7.217838000 -2.035541000 -2.750118000

H -6.231641000 -3.363769000 -2.113367000

H -7.949764000 -3.629648000 -2.480716000

C -3.232106000 -3.284689000 2.002402000

H -2.379889000 -3.581695000 2.625752000

H -4.087375000 -3.895411000 2.319954000

H -2.998350000 -3.551819000 0.964494000

C -3.981900000 -1.544892000 3.613017000

H -3.265430000 -1.955638000 4.336036000

H -4.106077000 -0.477577000 3.828547000

H -4.950147000 -2.036370000 3.782330000

C -2.143654000 -1.870966000 -1.024678000

H -3.056156000 -1.715326000 -0.449134000

H -2.318861000 -2.737849000 -1.680037000

H -1.329281000 -2.127550000 -0.337076000

C -3.096408000 -0.250008000 -2.633717000

H -3.469825000 -1.124219000 -3.188855000

H -3.888236000 0.057785000 -1.941979000

H -2.937117000 0.551404000 -3.362967000

C -0.742052000 -1.084767000 -2.885131000

H -0.437629000 -0.292945000 -3.574202000

H 0.149571000 -1.463131000 -2.366587000

H -1.148442000 -1.905744000 -3.494004000

C -2.371372000 3.046939000 -1.689769000

H -2.688384000 3.304489000 -0.671845000

H -2.306547000 3.986625000 -2.258427000

H -3.148011000 2.432086000 -2.156908000

C -0.008230000 3.311031000 -1.011693000

H -0.358479000 3.658768000 -0.034123000

H 0.981091000 2.851929000 -0.885495000

H 0.112518000 4.205052000 -1.640933000

C -0.543957000 2.192109000 -3.141192000

H -0.432548000 3.190468000 -3.588413000

H 0.429064000 1.692766000 -3.221050000

H -1.265255000 1.648182000 -3.758794000

C 0.943136000 -3.207263000 -0.333519000

C 0.408369000 -4.509310000 -0.889353000

H 0.043251000 -4.349518000 -1.910875000

H 1.222062000 -5.241507000 -0.952370000

H -0.395631000 -4.922821000 -0.273416000

O 0.481022000 -2.849292000 0.806986000

O 1.776524000 -2.568280000 -1.002956000

**IM6-b-r-2**

E = -2834.50875947 a.u.

C -3.374867000 -4.272350000 1.015993000

C -3.151551000 -2.930156000 1.263811000

C -3.514157000 -1.873553000 0.391101000

C -3.976487000 -2.281092000 -0.915620000

C -4.243475000 -3.648278000 -1.105203000

C -3.986001000 -4.641881000 -0.172237000

H -3.074848000 -5.012931000 1.754863000

H -2.644623000 -2.687942000 2.186492000

H -4.663249000 -3.967030000 -2.053715000

H -4.221917000 -5.679918000 -0.395854000

C -4.178590000 -1.395858000 -2.172646000

C -4.141130000 -2.220335000 -3.475367000

H -5.028600000 -2.846638000 -3.621507000

H -4.108074000 -1.521631000 -4.321484000

H -3.249869000 -2.858487000 -3.537678000

C -3.027621000 -0.395066000 -2.364144000

H -3.305998000 0.379322000 -3.093743000

H -2.718644000 0.127950000 -1.453396000

H -2.149211000 -0.917347000 -2.764290000

C -5.564767000 -0.735472000 -2.172756000

H -5.686473000 -0.116554000 -3.072591000

H -6.338177000 -1.515134000 -2.198630000

H -5.766151000 -0.109491000 -1.305582000

C 2.164586000 -1.299157000 1.026238000

C 1.445967000 -2.087993000 1.946396000

C 1.986909000 -2.596997000 3.117975000

C 3.305676000 -2.303600000 3.431640000

C 4.023271000 -1.484138000 2.574161000

C 3.494998000 -0.955283000 1.381859000

H 0.405484000 -2.312516000 1.732820000

H 1.372666000 -3.209043000 3.774937000

H 3.767164000 -2.685766000 4.339232000

H 5.048384000 -1.218264000 2.830721000

C 4.447330000 -0.021581000 0.695666000

C 4.448164000 1.345628000 1.056269000

C 5.459514000 -0.518382000 -0.149410000

C 5.403091000 2.190987000 0.490828000

C 6.383563000 0.370970000 -0.703516000

C 6.365051000 1.730726000 -0.406570000

H 5.400047000 3.250597000 0.749475000

H 7.146487000 -0.017744000 -1.378684000

P 1.026786000 -1.013916000 -0.460224000

Pd -1.298204000 -0.451912000 0.227428000

C 1.618518000 0.276997000 -1.742858000

C 1.035318000 -2.774609000 -1.283976000

C 7.313711000 2.705604000 -1.066185000

H 7.315828000 3.619910000 -0.452636000

C 3.457646000 1.914113000 2.060696000

H 2.552778000 1.286491000 2.036622000

C 5.624723000 -2.001939000 -0.418142000

H 4.691375000 -2.500419000 -0.127966000

C -3.301334000 -0.457085000 0.887437000

C -4.248395000 0.572997000 0.418042000

C -2.945037000 -0.167361000 2.410477000

C -4.176535000 1.771172000 -0.213071000

H -3.291166000 2.183971000 -0.686688000

C -5.471285000 2.387753000 -0.119810000

C -5.876678000 3.690103000 -0.658519000

O -7.011018000 4.135409000 -0.526711000

C -6.266033000 1.503447000 0.571871000

O -5.541643000 0.416292000 0.909111000

C -4.815565000 4.461686000 -1.392383000

H -3.942363000 4.630118000 -0.749592000

H -4.461523000 3.891827000 -2.260976000

H -5.216772000 5.421721000 -1.726220000

C -7.689058000 1.502910000 0.962839000

H -8.149577000 2.442004000 0.649378000

H -8.221928000 0.667517000 0.490313000

H -7.800515000 1.387536000 2.048002000

C -3.134289000 1.316976000 2.754563000

H -2.623715000 1.990024000 2.060549000

H -4.192387000 1.605221000 2.791972000

H -2.711081000 1.490772000 3.752792000

C -3.735060000 -0.904023000 3.500697000

H -3.591690000 -1.984204000 3.550589000

H -3.456124000 -0.502400000 4.484319000

H -4.810076000 -0.721463000 3.364679000

C -1.454371000 -0.425552000 2.302911000

H -0.803770000 0.371447000 2.686989000

H -1.112901000 -1.405864000 2.649875000

C 5.899517000 -2.341481000 -1.880449000

H 6.902344000 -2.022291000 -2.193459000

H 5.850684000 -3.428222000 -2.027246000

H 5.175486000 -1.878055000 -2.562199000

C 6.742218000 -2.568512000 0.459101000

H 6.859537000 -3.646806000 0.291281000

H 7.701685000 -2.087064000 0.225036000

H 6.543066000 -2.412316000 1.526224000

C 8.745572000 2.193162000 -1.155038000

H 9.407738000 2.972554000 -1.551696000

H 9.131062000 1.889154000 -0.174287000

H 8.824540000 1.330058000 -1.829216000

C 6.787567000 3.075268000 -2.452755000

H 6.757694000 2.188980000 -3.102154000

H 5.769598000 3.481792000 -2.398671000

H 7.429919000 3.824273000 -2.933110000

C 3.033254000 3.351482000 1.764566000

H 2.176949000 3.625470000 2.393271000

H 3.837156000 4.064210000 1.991279000

H 2.745806000 3.499011000 0.716328000

C 4.039663000 1.850362000 3.474145000

H 3.331042000 2.262753000 4.203911000

H 4.279119000 0.824806000 3.777513000

H 4.963480000 2.442878000 3.530801000

C 1.899063000 1.570640000 -0.983526000

H 2.891755000 1.558799000 -0.534530000

H 1.863586000 2.416690000 -1.686167000

H 1.161954000 1.758434000 -0.194738000

C 2.842602000 -0.083913000 -2.574694000

H 3.106492000 0.778790000 -3.205536000

H 3.711084000 -0.303500000 -1.944093000

H 2.670778000 -0.934292000 -3.244506000

C 0.424127000 0.562991000 -2.662508000

H 0.130955000 -0.283049000 -3.288938000

H -0.450845000 0.902197000 -2.090983000

H 0.708397000 1.384138000 -3.336431000

C 2.438646000 -3.356766000 -1.394965000

H 2.894934000 -3.507478000 -0.408893000

H 2.378718000 -4.341497000 -1.881854000

H 3.103550000 -2.729742000 -1.999993000

C 0.167819000 -3.731991000 -0.463446000

H 0.605759000 -3.985985000 0.507267000

H -0.841726000 -3.329747000 -0.298079000

H 0.068045000 -4.673214000 -1.023269000

C 0.405718000 -2.709245000 -2.674783000

H 0.343393000 -3.732842000 -3.070454000

H -0.617851000 -2.312207000 -2.646894000

H 0.993874000 -2.125976000 -3.389131000

C -0.795802000 3.564882000 -0.247215000

C -0.180937000 4.906717000 -0.027834000

H 0.904012000 4.815283000 -0.168142000

H -0.568093000 5.622120000 -0.755746000

H -0.353332000 5.264149000 0.991033000

O -1.324145000 3.199838000 -1.278596000

O -0.669725000 2.773347000 0.826535000

H -0.991952000 1.866170000 0.594114000

**IM5B-r**

E = -2217.60366813 a.u.

C -5.926444000 1.451375000 -2.226665000

C -5.190863000 2.095447000 -1.234902000

C -4.402133000 1.358357000 -0.358990000

C -4.320983000 -0.039062000 -0.439857000

C -5.058720000 -0.667883000 -1.450049000

C -5.854810000 0.066149000 -2.327643000

H -6.548065000 2.023480000 -2.912106000

H -5.229008000 3.179524000 -1.137853000

H -5.026710000 -1.749723000 -1.559231000

H -6.422544000 -0.454446000 -3.096726000

C -3.386175000 -0.811550000 0.500682000

C -3.711958000 -2.303982000 0.515396000

H -3.535411000 -2.775470000 -0.460414000

H -3.073905000 -2.810755000 1.248157000

H -4.760291000 -2.481097000 0.792989000

C -3.503147000 -0.267859000 1.926808000

H -2.903530000 -0.877136000 2.609937000

H -3.148628000 0.768202000 2.008035000

H -4.547452000 -0.293261000 2.267511000

C -1.990521000 -0.572166000 -0.098110000

H -2.027505000 -0.845176000 -1.157921000

H -1.720617000 0.493466000 -0.025973000

C 2.574154000 -0.707386000 -0.707661000

C 3.665043000 -1.480867000 -1.134046000

C 4.973848000 -1.238918000 -0.735469000

C 5.236006000 -0.191961000 0.133993000

C 4.186205000 0.628868000 0.520756000

C 2.863210000 0.428535000 0.101276000

H 3.505016000 -2.305372000 -1.815236000

H 5.773928000 -1.875866000 -1.105178000

H 6.246476000 0.010585000 0.481145000

H 4.395956000 1.500724000 1.138305000

C 1.935725000 1.570024000 0.389157000

C 1.133023000 1.636574000 1.549991000

C 1.965176000 2.668905000 -0.499755000

C 0.236610000 2.697585000 1.687319000

C 1.068772000 3.722220000 -0.301797000

C 0.160180000 3.729397000 0.750559000

H -0.418233000 2.724886000 2.557941000

H 1.061400000 4.556889000 -1.003506000

P 0.857883000 -1.343923000 -1.172218000

Pd -0.412611000 -1.528439000 0.777565000

C 0.349815000 -0.405545000 -2.788397000

C 1.035736000 -3.233384000 -1.618865000

C -0.896121000 4.804695000 0.848003000

H -0.674219000 5.551696000 0.070172000

C 1.329669000 0.665563000 2.698805000

H 1.568686000 -0.322405000 2.277897000

C 2.977970000 2.779556000 -1.630148000

H 3.345745000 1.770754000 -1.868587000

C -0.056557000 -2.750654000 3.042210000

C 0.072124000 -3.453559000 4.360076000

H 1.086778000 -3.356860000 4.756543000

H -0.651759000 -3.065219000 5.082232000

H -0.135367000 -4.520937000 4.216603000

O -1.196728000 -2.313460000 2.688057000

H -3.835710000 1.889834000 0.402821000

O 0.949180000 -2.623630000 2.288969000

C 1.593594000 -0.208467000 -3.655280000

H 2.062286000 -1.155252000 -3.945938000

H 1.295349000 0.307638000 -4.579246000

H 2.351936000 0.409249000 -3.160399000

C -0.714976000 -1.134772000 -3.612325000

H -1.634887000 -1.333328000 -3.049600000

H -0.989530000 -0.469793000 -4.444070000

H -0.371465000 -2.071322000 -4.058937000

C -0.237316000 0.962135000 -2.432423000

H -1.276626000 0.890811000 -2.096856000

H 0.332137000 1.491296000 -1.665730000

H -0.239581000 1.581555000 -3.340652000

C -0.390122000 -3.805973000 -1.596084000

H -1.077792000 -3.298550000 -2.278814000

H -0.339492000 -4.859702000 -1.905120000

H -0.819993000 -3.771926000 -0.586581000

C 1.652471000 -3.585468000 -2.976930000

H 1.739637000 -4.680497000 -3.021349000

H 1.030159000 -3.286806000 -3.824336000

H 2.656553000 -3.185487000 -3.144708000

C 1.812221000 -3.971295000 -0.521967000

H 1.469368000 -3.708769000 0.484355000

H 1.642466000 -5.048158000 -0.665359000

H 2.893213000 -3.811709000 -0.566137000

C 2.427679000 3.386383000 -2.918160000

H 2.225722000 4.459824000 -2.810178000

H 3.168267000 3.281349000 -3.721028000

H 1.503692000 2.900235000 -3.249253000

C 4.177697000 3.609585000 -1.164900000

H 4.932417000 3.677532000 -1.959057000

H 3.861409000 4.631880000 -0.915249000

H 4.659282000 3.184026000 -0.276956000

C 0.116285000 0.508139000 3.607846000

H 0.301765000 -0.294471000 4.334417000

H -0.072003000 1.421390000 4.188460000

H -0.795577000 0.251189000 3.055456000

C 2.509831000 1.127165000 3.559480000

H 2.657861000 0.441162000 4.403557000

H 3.449322000 1.174603000 2.999506000

H 2.311919000 2.127644000 3.969658000

C -0.904043000 5.517277000 2.195788000

H 0.071281000 5.965068000 2.421474000

H -1.653810000 6.318089000 2.202895000

H -1.154839000 4.829017000 3.013395000

C -2.268284000 4.204120000 0.543704000

H -2.263418000 3.664053000 -0.413607000

H -2.556514000 3.490893000 1.329922000

H -3.042528000 4.980893000 0.499018000

**IM6B-r**

E = -2834.48804673 a.u.

C -4.037944000 6.286327000 0.932294000

C -3.571710000 5.844509000 -0.302550000

C -3.523643000 4.484035000 -0.590603000

C -3.943459000 3.523430000 0.340601000

C -4.399770000 3.987989000 1.580828000

C -4.450439000 5.348395000 1.873330000

H -4.077366000 7.349603000 1.159011000

H -3.241391000 6.562790000 -1.050759000

H -4.727801000 3.280864000 2.339978000

H -4.816817000 5.674809000 2.844991000

C -3.821720000 2.019047000 0.046367000

C -4.058489000 1.747465000 -1.439945000

H -3.301155000 2.198564000 -2.089613000

H -4.048187000 0.669226000 -1.633872000

H -5.039182000 2.129863000 -1.755646000

C -4.888371000 1.248527000 0.830279000

H -4.968497000 0.220864000 0.466685000

H -4.672887000 1.198832000 1.905644000

H -5.874476000 1.716103000 0.704103000

C -2.373492000 1.691326000 0.488243000

H -1.737450000 2.461332000 0.047309000

H -2.316213000 1.812074000 1.582422000

C 1.702824000 -0.016984000 -1.505209000

C 0.804788000 -0.359856000 -2.533016000

C 1.182261000 -1.028869000 -3.688256000

C 2.505985000 -1.412995000 -3.842866000

C 3.398144000 -1.151360000 -2.813464000

C 3.043665000 -0.472363000 -1.634349000

H -0.249443000 -0.120106000 -2.408502000

H 0.436871000 -1.258197000 -4.446295000

H 2.839407000 -1.935690000 -4.736518000

H 4.428712000 -1.492455000 -2.904848000

C 4.172202000 -0.401664000 -0.645245000

C 4.302518000 -1.417865000 0.332960000

C 5.206182000 0.545856000 -0.790520000

C 5.371146000 -1.366629000 1.227534000

C 6.264226000 0.548282000 0.123839000

C 6.351529000 -0.376305000 1.157454000

H 5.452493000 -2.135005000 1.996686000

H 7.051351000 1.297062000 0.021635000

P 0.762510000 1.106150000 -0.309645000

Pd -1.320519000 -0.089457000 0.185504000

C 1.578701000 1.405089000 1.395403000

C 0.750151000 2.763128000 -1.325219000

C 7.475404000 -0.308872000 2.165997000

H 8.162519000 0.482793000 1.829783000

C 3.372381000 -2.620924000 0.364755000

H 2.395632000 -2.319351000 -0.041581000

C 5.256011000 1.532606000 -1.940707000

H 4.268466000 1.546906000 -2.415918000

C -0.428239000 -2.710126000 -0.871207000

C 0.380938000 -3.989434000 -0.938170000

H 0.695152000 -4.326837000 0.055809000

H -0.180249000 -4.781538000 -1.443306000

H 1.286011000 -3.790269000 -1.530291000

O -1.261395000 -2.478714000 -1.761225000

H -3.145165000 4.171479000 -1.562482000

O -0.136239000 -1.931486000 0.105376000

C -2.739419000 -1.323398000 1.436272000

C -3.139881000 -1.429791000 0.269685000

C -2.549060000 -1.591117000 2.881092000

C -1.679960000 -2.851083000 3.000167000

H -0.701107000 -2.698912000 2.527443000

H -1.532294000 -3.091078000 4.061228000

H -2.163376000 -3.709371000 2.515455000

C -1.884475000 -0.437343000 3.626862000

H -2.443539000 0.500149000 3.512051000

H -1.847382000 -0.680045000 4.696518000

H -0.856489000 -0.276698000 3.281137000

C -3.939108000 -1.852191000 3.479103000

H -4.573165000 -0.958486000 3.406836000

H -4.445174000 -2.682856000 2.970945000

H -3.833275000 -2.108956000 4.541094000

C -3.835784000 -1.752991000 -0.927140000

H -3.412032000 -1.390306000 -1.859604000

C -4.971444000 -2.483936000 -0.925114000

C -5.707727000 -2.829847000 -2.158811000

O -6.702696000 -3.539477000 -2.078532000

C -5.517087000 -3.051050000 0.357682000

O -4.936972000 -3.963518000 0.917098000

C -5.208295000 -2.310627000 -3.474273000

H -5.186963000 -1.214082000 -3.480642000

H -4.186539000 -2.658399000 -3.668665000

H -5.866530000 -2.663815000 -4.271271000

C -6.739698000 -2.397691000 0.915219000

H -6.485302000 -1.368496000 1.209109000

H -7.522682000 -2.326021000 0.152490000

H -7.105912000 -2.945046000 1.788100000

C 2.899906000 2.166400000 1.382481000

H 2.795968000 3.191032000 1.008368000

H 3.267158000 2.234568000 2.417534000

H 3.671121000 1.658129000 0.797193000

C 1.789509000 0.030260000 2.020939000

H 0.897120000 -0.605353000 1.954032000

H 2.614691000 -0.492635000 1.538556000

H 2.049567000 0.159362000 3.082213000

C 0.580137000 2.160924000 2.277498000

H -0.367483000 1.622606000 2.376412000

H 1.021354000 2.243083000 3.281049000

H 0.367157000 3.175672000 1.932226000

C 0.426161000 3.988825000 -0.464946000

H 1.209138000 4.213019000 0.266193000

H 0.361392000 4.856518000 -1.136941000

H -0.531509000 3.924905000 0.061511000

C -0.295591000 2.665086000 -2.435112000

H -0.379481000 3.646028000 -2.924457000

H -0.013792000 1.943266000 -3.208375000

H -1.286642000 2.394190000 -2.055529000

C 2.111511000 2.994146000 -1.971052000

H 2.095349000 3.960761000 -2.495675000

H 2.916483000 3.032583000 -1.230161000

H 2.347956000 2.221248000 -2.711172000

C 5.592574000 2.957997000 -1.505637000

H 6.653635000 3.060655000 -1.243437000

H 5.395021000 3.659959000 -2.326051000

H 5.006443000 3.281871000 -0.636431000

C 6.263317000 1.069537000 -2.994661000

H 6.292251000 1.773276000 -3.836599000

H 7.275618000 1.015016000 -2.571418000

H 6.014545000 0.078112000 -3.392385000

C 8.263686000 -1.612407000 2.246522000

H 8.654623000 -1.910135000 1.266049000

H 9.113095000 -1.506370000 2.932811000

H 7.640802000 -2.434973000 2.622414000

C 6.942858000 0.085161000 3.541875000

H 6.255901000 -0.678562000 3.931445000

H 7.762506000 0.193280000 4.263468000

H 6.396435000 1.035882000 3.504177000

C 3.123545000 -3.204124000 1.753234000

H 2.326427000 -3.957644000 1.696025000

H 4.011501000 -3.715323000 2.148801000

H 2.816552000 -2.448894000 2.487040000

C 3.953970000 -3.718761000 -0.530894000

H 4.045193000 -3.396757000 -1.575231000

H 4.955450000 -4.004910000 -0.179844000

H 3.325978000 -4.618895000 -0.507988000

**TS_6-7B­_-r**

E = -2834.47795485 a.u.

C -4.251646000 6.194449000 -1.183768000

C -4.561206000 5.491489000 -0.021046000

C -4.402650000 4.111822000 0.026267000

C -3.933415000 3.387199000 -1.080702000

C -3.630027000 4.110264000 -2.239410000

C -3.787035000 5.495275000 -2.291286000

H -4.373428000 7.274752000 -1.223920000

H -4.928700000 6.021121000 0.856090000

H -3.264723000 3.597927000 -3.126646000

H -3.542863000 6.027278000 -3.209115000

C -3.718553000 1.869949000 -0.960365000

C -3.389420000 1.249820000 -2.314233000

H -2.454217000 1.637151000 -2.735877000

H -3.279738000 0.163344000 -2.209168000

H -4.192093000 1.430814000 -3.042611000

C -5.026360000 1.243708000 -0.467293000

H -4.971859000 0.150032000 -0.452557000

H -5.299520000 1.572035000 0.542177000

H -5.849710000 1.525952000 -1.138118000

C -2.522607000 1.743687000 0.017727000

H -1.777933000 2.452544000 -0.345789000

H -2.839712000 2.100227000 1.011381000

C 1.885443000 0.628260000 1.432582000

C 1.122455000 0.833143000 2.598010000

C 1.636148000 0.752318000 3.883561000

C 2.975986000 0.433243000 4.050856000

C 3.737412000 0.158205000 2.925414000

C 3.236905000 0.222273000 1.611862000

H 0.063752000 1.060778000 2.488357000

H 0.987743000 0.935249000 4.738100000

H 3.420901000 0.369314000 5.041216000

H 4.777243000 -0.142405000 3.051045000

C 4.226852000 -0.260727000 0.588576000

C 5.261756000 0.573097000 0.117644000

C 4.230451000 -1.631037000 0.230327000

C 6.181698000 0.069924000 -0.806805000

C 5.170071000 -2.084803000 -0.694885000

C 6.136509000 -1.247038000 -1.249168000

H 6.957694000 0.734530000 -1.186062000

H 5.162779000 -3.131974000 -0.999107000

P 0.777351000 1.127151000 -0.022034000

Pd -1.390591000 0.042955000 0.478647000

C 0.909532000 3.066297000 0.103969000

C 1.385915000 0.565829000 -1.746074000

C 7.053014000 -1.768698000 -2.332076000

H 7.141318000 -2.856908000 -2.187334000

C 5.459064000 1.989631000 0.618592000

H 4.544732000 2.288894000 1.143307000

C 3.290839000 -2.636028000 0.880406000

H 2.372701000 -2.106734000 1.178672000

C -0.496550000 -2.854819000 0.632804000

C -0.366269000 -4.077677000 1.522270000

H -0.115341000 -4.970917000 0.941800000

H 0.365065000 -3.928767000 2.324424000

H -1.343308000 -4.250852000 1.996257000

O -0.781985000 -3.010205000 -0.566728000

H -4.645677000 3.587312000 0.949705000

O -0.349748000 -1.735414000 1.238872000

C -3.070186000 -0.884098000 1.329427000

C -3.512086000 -1.632426000 0.373596000

C -3.442177000 -0.722897000 2.778649000

C -4.870064000 -0.175695000 2.891584000

H -5.588550000 -0.799537000 2.350084000

H -5.156173000 -0.152952000 3.951407000

H -4.935234000 0.850547000 2.508554000

C -3.367125000 -2.116276000 3.422987000

H -2.357617000 -2.536061000 3.325056000

H -3.597116000 -2.031325000 4.493350000

H -4.080436000 -2.809350000 2.964884000

C -2.494563000 0.209057000 3.531004000

H -1.467088000 -0.173680000 3.512826000

H -2.498074000 1.223386000 3.111114000

H -2.813007000 0.278960000 4.579706000

C -3.439751000 -2.154862000 -0.922287000

H -2.585404000 -1.869018000 -1.528773000

C -4.413404000 -3.014876000 -1.350767000

C -4.435314000 -3.625758000 -2.690018000

O -5.356538000 -4.347722000 -3.044898000

C -5.403128000 -3.220649000 -0.306478000

O -5.162147000 -2.645276000 0.776434000

C -3.274626000 -3.325024000 -3.595965000

H -3.229220000 -2.251666000 -3.821853000

H -2.324637000 -3.596678000 -3.119223000

H -3.389089000 -3.881912000 -4.528552000

C -6.631669000 -4.035814000 -0.465141000

H -6.365504000 -5.069883000 -0.709015000

H -7.219807000 -4.006135000 0.454799000

H -7.224267000 -3.668043000 -1.309206000

C 2.355896000 3.499215000 0.317369000

H 2.736139000 3.166662000 1.290272000

H 3.024407000 3.135062000 -0.467519000

H 2.401642000 4.598219000 0.306729000

C 0.108035000 3.586443000 1.301217000

H 0.193232000 4.682563000 1.309160000

H -0.958079000 3.342281000 1.260115000

H 0.510235000 3.230429000 2.255407000

C 0.394654000 3.753131000 -1.164023000

H -0.635419000 3.489794000 -1.429991000

H 0.410440000 4.838168000 -0.987817000

H 1.033508000 3.564099000 -2.032687000

C 0.251290000 0.815898000 -2.742231000

H 0.001591000 1.872193000 -2.880496000

H 0.568347000 0.423973000 -3.719621000

H -0.657072000 0.275026000 -2.447674000

C 1.577645000 -0.943924000 -1.674311000

H 2.413310000 -1.199928000 -1.024810000

H 0.680390000 -1.472615000 -1.326660000

H 1.821886000 -1.310931000 -2.683161000

C 2.669075000 1.213791000 -2.255738000

H 3.511047000 1.063961000 -1.571957000

H 2.937769000 0.739242000 -3.211791000

H 2.559305000 2.287061000 -2.447845000

C 6.405823000 -1.530675000 -3.696582000

H 6.257273000 -0.454644000 -3.866204000

H 7.036108000 -1.917696000 -4.507435000

H 5.424824000 -2.017495000 -3.765950000

C 8.453120000 -1.170558000 -2.298518000

H 8.918998000 -1.270963000 -1.310648000

H 9.098339000 -1.673044000 -3.029192000

H 8.443399000 -0.104188000 -2.560195000

C 6.604477000 2.038398000 1.630969000

H 6.733726000 3.056509000 2.020571000

H 6.426228000 1.371902000 2.483446000

H 7.552177000 1.736904000 1.164178000

C 5.716982000 2.997288000 -0.499510000

H 5.639012000 4.021649000 -0.112934000

H 6.725419000 2.886990000 -0.919410000

H 5.002792000 2.890856000 -1.325868000

C 2.886870000 -3.795835000 -0.025110000

H 3.736648000 -4.454231000 -0.247151000

H 2.137023000 -4.414269000 0.482775000

H 2.456933000 -3.465072000 -0.978270000

C 3.941683000 -3.203675000 2.144283000

H 4.871314000 -3.731928000 1.890051000

H 4.188022000 -2.421677000 2.871635000

H 3.270882000 -3.921869000 2.633916000

**IM7B-r**

E = -2834.50474201 a.u.

C -4.399090000 6.257438000 -1.124876000

C -4.764051000 5.515099000 -0.003519000

C -4.578471000 4.138028000 0.016111000

C -4.027160000 3.454190000 -1.078529000

C -3.666777000 4.217298000 -2.194894000

C -3.849754000 5.599664000 -2.218833000

H -4.543137000 7.335616000 -1.143423000

H -5.197779000 6.011416000 0.862846000

H -3.234448000 3.738933000 -3.070938000

H -3.560623000 6.162198000 -3.104880000

C -3.781509000 1.940127000 -0.989092000

C -3.414581000 1.361882000 -2.351910000

H -2.467764000 1.760285000 -2.736180000

H -3.306046000 0.273415000 -2.278074000

H -4.196508000 1.564670000 -3.097069000

C -5.073138000 1.260664000 -0.530203000

H -4.966645000 0.170196000 -0.526260000

H -5.378516000 1.565250000 0.477478000

H -5.896967000 1.510573000 -1.213331000

C -2.610012000 1.801329000 0.012227000

H -1.847402000 2.507109000 -0.326359000

H -2.955741000 2.161981000 0.994425000

C 1.870875000 0.658162000 1.432468000

C 1.096617000 0.846063000 2.593704000

C 1.597465000 0.748859000 3.883381000

C 2.936088000 0.429318000 4.060041000

C 3.708269000 0.167872000 2.938456000

C 3.219108000 0.247497000 1.621524000

H 0.039463000 1.076889000 2.476477000

H 0.940193000 0.919793000 4.733685000

H 3.371506000 0.353586000 5.053836000

H 4.746522000 -0.135741000 3.070254000

C 4.212839000 -0.233424000 0.601590000

C 5.252655000 0.598026000 0.137503000

C 4.211460000 -1.602486000 0.239939000

C 6.178295000 0.091673000 -0.779294000

C 5.158892000 -2.060721000 -0.675251000

C 6.133832000 -1.226406000 -1.219660000

H 6.958671000 0.753807000 -1.154294000

H 5.148197000 -3.107592000 -0.980891000

P 0.769978000 1.175158000 -0.021883000

Pd -1.477158000 0.087639000 0.470121000

C 0.905939000 3.111733000 0.138587000

C 1.395450000 0.647186000 -1.750514000

C 7.059784000 -1.749895000 -2.293672000

H 7.118668000 -2.842480000 -2.168426000

C 5.446843000 2.013803000 0.641857000

H 4.525203000 2.314522000 1.152598000

C 3.251435000 -2.600667000 0.868881000

H 2.335057000 -2.062507000 1.157044000

C -0.495655000 -2.828913000 0.596372000

C -0.377063000 -4.051603000 1.490122000

H -0.075554000 -4.936778000 0.921498000

H 0.310978000 -3.884670000 2.326391000

H -1.369727000 -4.251891000 1.919286000

O -0.690015000 -2.986881000 -0.618395000

H -4.864578000 3.584137000 0.909318000

O -0.437281000 -1.709904000 1.222212000

C -3.069864000 -0.896950000 1.175235000

C -3.648172000 -1.842606000 0.339549000

C -3.457908000 -0.800557000 2.637494000

C -4.935510000 -0.389134000 2.758173000

H -5.615778000 -1.079701000 2.249990000

H -5.206536000 -0.365219000 3.821846000

H -5.095234000 0.617912000 2.353097000

C -3.227153000 -2.158567000 3.325090000

H -2.177758000 -2.462457000 3.222880000

H -3.448174000 -2.055884000 4.395654000

H -3.863830000 -2.951602000 2.922498000

C -2.624378000 0.234542000 3.395799000

H -1.560783000 -0.032971000 3.391087000

H -2.732309000 1.242121000 2.974975000

H -2.957997000 0.271271000 4.441757000

C -3.375624000 -2.147322000 -0.991858000

H -2.622111000 -1.642188000 -1.582889000

C -4.219192000 -3.188448000 -1.389270000

C -4.305489000 -3.854378000 -2.698630000

O -5.110156000 -4.753530000 -2.890618000

C -5.002402000 -3.492215000 -0.273307000

O -4.676147000 -2.720914000 0.750537000

C -3.361441000 -3.369689000 -3.755835000

H -3.516906000 -2.299944000 -3.945830000

H -2.322921000 -3.486439000 -3.420174000

H -3.513733000 -3.931732000 -4.679946000

C -6.073477000 -4.478983000 -0.078794000

H -5.675665000 -5.493250000 -0.199008000

H -6.515708000 -4.377858000 0.914979000

H -6.845421000 -4.357004000 -0.845364000

C 2.345043000 3.558085000 0.368140000

H 2.723918000 3.211161000 1.336795000

H 3.022806000 3.216101000 -0.418746000

H 2.379899000 4.657718000 0.379110000

C 0.089181000 3.601553000 1.338386000

H 0.134008000 4.700162000 1.352378000

H -0.967116000 3.316806000 1.297185000

H 0.504614000 3.254878000 2.290357000

C 0.385615000 3.812866000 -1.118732000

H -0.645158000 3.547603000 -1.381505000

H 0.397996000 4.896227000 -0.931536000

H 1.020723000 3.634570000 -1.992211000

C 0.256332000 0.905154000 -2.741119000

H 0.005951000 1.962689000 -2.868373000

H 0.567924000 0.522486000 -3.724098000

H -0.652288000 0.363527000 -2.446936000

C 1.595276000 -0.862964000 -1.703261000

H 2.448133000 -1.123011000 -1.077161000

H 0.709527000 -1.404367000 -1.344098000

H 1.818965000 -1.216395000 -2.721922000

C 2.675677000 1.305018000 -2.253156000

H 3.517338000 1.143886000 -1.571221000

H 2.947088000 0.846459000 -3.216393000

H 2.566766000 2.381646000 -2.427284000

C 6.448198000 -1.470676000 -3.666824000

H 6.346527000 -0.387873000 -3.826984000

H 7.078089000 -1.872677000 -4.470703000

H 5.450134000 -1.917242000 -3.761187000

C 8.472561000 -1.186198000 -2.219414000

H 8.916399000 -1.318677000 -1.225222000

H 9.120836000 -1.686862000 -2.948682000

H 8.491806000 -0.114270000 -2.456721000

C 6.577591000 2.058551000 1.670845000

H 6.704131000 3.075835000 2.063530000

H 6.385502000 1.391649000 2.520066000

H 7.531005000 1.754929000 1.217138000

C 5.721976000 3.023820000 -0.469565000

H 5.652016000 4.046672000 -0.077421000

H 6.731592000 2.906123000 -0.884639000

H 5.010714000 2.929994000 -1.299960000

C 2.847184000 -3.748829000 -0.052047000

H 3.677985000 -4.446334000 -0.220546000

H 2.038374000 -4.324641000 0.414216000

H 2.488598000 -3.406828000 -1.030195000

C 3.873631000 -3.185894000 2.139047000

H 4.807109000 -3.713544000 1.898033000

H 4.105814000 -2.413933000 2.881642000

H 3.191058000 -3.909020000 2.604741000
