# Supplementary material for: Marine furanocembranoids-inspired macrocycles enabled by Pd-catalyzed unactivated C(sp3)-H olefination mediated by donor/donor carbenes
Source: Nat Commun. 2021 Feb 26;12:1304. doi: 10.1038/s41467-021-21484-x (PMC7910576; doi:10.1038/s41467-021-21484-x)
Supplement: Supplementary file 6 — Reporting Summary [file 41467_2021_21484_MOESM6_ESM.pdf]

nature research

Corresponding author(s): Weibo Yang

Last updated by author(s): Jan 11, 2021

Reporting Summary

Nature Research wishes to improve the reproducibility of the work that we publish. This form provides structure for consistency and transparency in reporting. For further information on Nature Research policies, see our Editorial Policies and the Editorial Policy Checklist.

Statistics

For all statistical analyses, confirm that the following items are present in the figure legend, table legend, main text, or Methods section.

n/a	Confirmed	
x		The exact sample size (*n*) for each experimental group/condition, given as a discrete number and unit of measurement
x		A statement on whether measurements were taken from distinct samples or whether the same sample was measured repeatedly
x		The statistical test(s) used AND whether they are one- or two-sided<br>*Only common tests should be described solely by name; describe more complex techniques in the Methods section.*
x		A description of all covariates tested
x		A description of any assumptions or corrections, such as tests of normality and adjustment for multiple comparisons
	x	A full description of the statistical parameters including central tendency (e.g. means) or other basic estimates (e.g. regression coefficient) AND variation (e.g. standard deviation) or associated estimates of uncertainty (e.g. confidence intervals)
x		For null hypothesis testing, the test statistic (e.g. *F*, *t*, *r*) with confidence intervals, effect sizes, degrees of freedom and *P* value noted<br>*Give P values as exact values whenever suitable.*
x		For Bayesian analysis, information on the choice of priors and Markov chain Monte Carlo settings
x		For hierarchical and complex designs, identification of the appropriate level for tests and full reporting of outcomes
x		Estimates of effect sizes (e.g. Cohen's *d*, Pearson's *r*), indicating how they were calculated

Our web collection on statistics for biologists contains articles on many of the points above.

Software and code

Policy information about availability of computer code

Data collection	Gaussian 09, Revision D.01
Data analysis	GaussView 5.0

For manuscripts utilizing custom algorithms or software that are central to the research but not yet described in published literature, software must be made available to editors and reviewers. We strongly encourage code deposition in a community repository (e.g. GitHub). See the Nature Research guidelines for submitting code & software for further information.

Data

Policy information about availability of data

All manuscripts must include a data availability statement. This statement should provide the following information, where applicable:

- Accession codes, unique identifiers, or web links for publicly available datasets
- A list of figures that have associated raw data
- A description of any restrictions on data availability

The authors confirm that the data supporting the findings of this study are available within the article and its supplementary materials.

Field-specific reporting

Life sciences study design

All studies must disclose on these points even when the disclosure is negative.

Sample size	All of the bioactivity tests were conducted in triplicate or duplicate for each assay.
Data exclusions	No data were excluded from the analyses.
Replication	All of the bioactivity tests were conducted in triplicate or duplicate for each assay, and independent experiment was repeated at least twice.
Randomization	Randomization is not relevant to this study. Identical cellular system condition with same stimulation was subjected to the indicated compounds, and cytokines and signaling proteins in respective cultured wells were determined.
Blinding	Blinding was not relevant to this study. Sample grouping was not applied in bioactivity assays.

Reporting for specific materials, systems and methods

We require information from authors about some types of materials, experimental systems and methods used in many studies. Here, indicate whether each material, system or method listed is relevant to your study. If you are not sure if a list item applies to your research, read the appropriate section before selecting a response.

Materials & experimental systems

n/a	Involved in the study
☐	☒ Antibodies
☐	☒ Eukaryotic cell lines
☒	☐ Palaeontology and archaeology
☒	☐ Animals and other organisms
☒	☐ Human research participants
☒	☐ Clinical data
☒	☐ Dual use research of concern

Methods

n/a	Involved in the study
☒	☐ ChIP-seq
☒	☐ Flow cytometry
☒	☐ MRI-based neuroimaging

Antibodies

Antibodies used	Anti-IKKa antidody: Cell Signaling Technology. catalog number: 61294; clone name: D3W6N; lot number: 1.<br>Anti-p-IKKa antidody: Cell Signaling Technology. catalog number: 2697; clone name: 16A6; lot number: 19.<br>Anti-IKB-α antidody: Cell Signaling Technology. catalog number: 4812; clone name: 44D4; lot number: 12.<br>Anti-NF-κb antidody: Cell Signaling Technology. catalog number: 8242; clone name: D14E12; lot number: 9.<br>Anti-p-NF-κb antidody: Cell Signaling Technology. catalog number: 3033; clone name: 93H1; lot number: 16.<br>HRP-conjugated Monoclonal Mouse Anti-glyceraldehyde-3-phosphate Dehydrogease (GAPDH): KangChen Biotech. catalog number: KC-5G5; lot number: 1907.
Validation	Anti-IKKa antidody: species: human, mouse, rat, application: WB, IP.<br>Anti-p-IKKa antidody: species: human, mouse, rat, monkey; application: WB, IP,IHC,FC.<br>Anti-IKB-α antidody: species: human, mouse, rat, monkey, Mink ; application: WB, IP.<br>Anti-NF-κb antidody: species: human, mouse, rat, monkey, Mink, Dog; application: WB, IP,IHC,FC,ChIP.<br>Anti-p-NF-κb antidody: species: human, mouse, rat, monkey, Pig, Dog; application: WB, IP,IHC,FC.<br>HRP-conjugated Monoclonal Mouse Anti-glyceraldehyde-3-phosphate Dehydrogease (GAPDH): species: mouse.

Eukaryotic cell lines

Policy information about cell lines

Cell line source(s)	The RAW264.7 cell line was purchased from the American Type Culture Collection (ATCC; Manassas, VA, USA)
Authentication	Cells have been authenticated by morphology, karyotyping, and PCR based approaches to confirm the identity.
Mycoplasma contamination	All cells used were negative for mycoplasma contamination.
Commonly misidentified lines (See ICLAC register)	No commonly misidentified cell lines were used.
